# In-depth mass spectrometric mapping of the human vitreous proteome

**DOI:** 10.1186/1477-5956-11-22

**Published:** 2013-05-20

**Authors:** Sebastian Aretz, Tim U Krohne, Kerstin Kammerer, Uwe Warnken, Agnes Hotz-Wagenblatt, Marion Bergmann, Boris V Stanzel, Tore Kempf, Frank G Holz, Martina Schnölzer, Jürgen Kopitz

**Affiliations:** 1 Institute of Pathology, University of Heidelberg, Im Neuenheimer Feld 220, Heidelberg, D-69120, Germany; 2 University Eye Hospital, University of Bonn, Bonn, Germany; 3 Functional Proteome Analysis, German Cancer Research Center (DKFZ), Heidelberg, Germany

**Keywords:** Human eye, Vitreous proteome, Eye disease, Prefractionation strategy

## Abstract

Mapping of proteins involved in normal eye functions is a prerequisite to identify pathological changes during eye disease processes. We therefore analysed the proteome of human vitreous by applying in-depth proteomic screening technologies. For ethical reasons human vitreous samples were obtained by vitrectomy from “surrogate normal patients” with epiretinal gliosis that is considered to constitute only negligible pathological vitreoretinal changes. We applied different protein prefractionation strategies including liquid phase isoelectric focussing, 1D SDS gel electrophoresis and a combination of both and compared the number of identified proteins obtained by the respective method. Liquid phase isoelectric focussing followed by SDS gel electrophoresis increased the number of identified proteins by a factor of five compared to the analysis of crude unseparated human vitreous. Depending on the prefractionation method proteins were subjected to trypsin digestion either in-gel or in solution and the resulting peptides were analysed on a UPLC system coupled online to an LTQ Orbitrap XL mass spectrometer. The obtained mass spectra were searched against the SwissProt database using the Mascot search engine. Bioinformatics tools were used to annotate known biological functions to the detected proteins. Following this strategy we examined the vitreous proteomes of three individuals and identified 1111 unique proteins. Besides structural, transport and binding proteins, we detected 261 proteins with known enzymatic activity, 51 proteases, 35 protease inhibitors, 35 members of complement and coagulation cascades, 15 peptide hormones, 5 growth factors, 11 cytokines, 47 receptors, 30 proteins of visual perception, 91 proteins involved in apoptosis regulation and 265 proteins with signalling activity. This highly complex mixture strikingly differs from the human plasma proteome. Thus human vitreous fluid seems to be a unique body fluid. 262 unique proteins were detected which are present in all three patient samples indicating that these might represent the constitutive protein pattern of human vitreous. The presented catalogue of human vitreous proteins will enhance our understanding of physiological processes in the eye and provides the groundwork for future studies on pathological vitreous proteome changes.

## Background

The human vitreous humor is a clear aqueous solution which fills the posterior compartment of the eye, located between the lens and the retina. It occupies about 80% of the volume of the eyeball and comprises 99% water but has a gel-like structure at birth due to a network of collagen fibrils and large molecules of hyaluronic acid. With aging the vitreous undergoes a process of progressive liquefaction so that at the age of 80–90 years more than half of the vitreous is liquid [1]. Vitreous humor contains several low molecular weight solutes including inorganic salts, sugars and ascorbic acid. The total concentration of protein in human vitreous is approximately 1200 μg/ml, of which collagen accounts for 180 μg/ml [2]. Soluble proteins in vitreous humor are thought to originate from plasma by filtration through fenestrated capillaries of the ciliary body stroma via the iris root [3,4]. However, protein secretion or shedding from posterior chamber tissues, including photoreceptors and the retinal pigment epithelium, may have a significant impact on the pattern of soluble proteins found in the vitreous. Since, as in all other human tissues, a major function of soluble extracellular proteins is maintenance of homeostasis in adjacent tissues, vitreous fluid contains soluble proteins that are necessary to sustain normal ocular morphology and functions. Such important physiological role requires tight control of the qualitative and quantitative composition of the vitreous proteome. Consequently the vitreous in the healthy eye should contain a complex but defined protein mixture. Studies investigating the vitreous proteome provide valuable information for the understanding of ocular biochemistry. Conversely, if the protein composition of the vitreous is central to the health of the human eye, alterations in vitreous protein expression will be on one hand indicative of ocular disease, on the other hand may be actively involved in the pathogenic process. Indeed, a number of vitreous fluid proteins have already been linked to the aetiology of ocular and vitreoretinal disorders [5,6]. However, we are still far from a detailed understanding of the complex biochemical processes mediated by vitreous proteins, and consequently extensive analysis of the role of vitreous proteome changes in the development of eye diseases is not yet feasible.

Towards a better understanding of vitreous protein composition and functions a number of proteomic analyses have already been conducted. In recent studies only 38 resp. 121 individual proteins were identified in human vitreous humor [7,8]. By applying different proteomic methods including 2-DE/MALDI-MS, nanoLC-MALDI-MS/MS, and nanoLC-ESI-MS/MS a total of 346 individual proteins were identified in vitreous of a non-diabetic control group [9]. In a recent study 355 proteins were identified by nano-LC-ESI-MS/MS and 206 proteins by antibody-based protein arrays in human aqueous humor whose composition may be similar to the vitreous body [10]. Analysis of aqueous humor collected during routine cataract surgery from otherwise healthy eyes identified 198 unique proteins [11]. However, considering the complexity of other body fluids like blood plasma or cerebrospinal fluid containing thousands of different proteins, it is likely to assume that only the most abundant proteins in the vitreous have been detected up to now. Indeed in the present study applying state-of-the art proteomics technologies we were able to detect more than 1000 different proteins in human vitreous.

## Methods

### Sample collection

Following approval by the ethics review board of the University Hospital Bonn, vitreous material was sampled from patients undergoing elective pars plana vitrectomy for epiretinal gliosis at the Bonn University Eye Hospital. Written consent from the patients was obtained for use of the samples in the present study. Patients with previous vitreoretinal surgery, intravitreal drug injections, or additional vitreoretinal diseases were excluded. During vitrectomy, complete vitreous material diluted in surgical irrigation fluid (balanced salt solution; PuriSol, Carl Zeiss Meditec, Jena, Germany) was collected. Samples were stored frozen at −80°C immediately after surgery until analysis. Contamination with blood during surgery was excluded by haemoglobin ELISA (Bethyl Laboratories; Montgomery, TX, USA). Only samples without detectable haemoglobin were used in this study. The test has a detection limit of 0.27 ng/ml.

### Sample processing and fractionation

Before further processing, protein content of the vitreous samples was determined by the Lowry procedure [12]. Protein was precipitated from aliquots of vitreous samples (representing 50 μg of protein for SDS polyacrylamide gel electrophoresis (SDS PAGE) and 1mg for liquid phase isoelectric focussing (liquid phase IEF)) by adding an equal volume of 10% trichloroacetic acid (TCA). After centrifugation (15 min, 15000 g, 4°C) and washing with 1 ml ice-cold acetone the protein pellet was dried for ~10 min *in vacuo* and resolubilized for SDS PAGE or liquid phase IEF.

For SDS-PAGE vitreous protein precipitate was dissolved in NuPAGE LDS sample buffer and separated on a NuPAGE Novex 4-12% Bis Tris gel (Invitrogen, Carlsbad, CA, USA) following the manufacturer’s instructions. The gel was Coomassie-stained according to the procedure of Kang *et al*. [13] and each lane was cut into 26 pieces. Liquid phase IEF was conducted in a ZOOM® IEF Fractionator (Invitrogen). To this end the protein precipitate (1 mg) was dissolved in 900 μl 1.1× IEF Denaturant (7.7 M urea, 2.2 M thiourea, 4.4% CHAPS). Then 10 μl 100× protease inhibitor (90 mM AEBSF, 430 mM EDTA, 8.5 mM Bestatin, 1.4 mM Pepstatin A, 1.1 mM E-64, 1.0 mM Leupeptin) and 10 μl 2 M DTT were added. The mixture was sonicated (10 rounds / 10 s / 50% power), and 10 μl 1 M Tris base were added, followed by an incubation time of 30 min at room temperature (RT). 5.2 μl of dimethylacetamide were added and incubated for further 30 min at RT. Finally 10 μl of ZOOM carrier ampholytes (pH3-10) and 10 μl 2 M DTT were added to the sample solution. The whole mixture was filled up to a final volume of 3.5 ml with IEF denaturant and loaded to the ZOOM IEF fractionator. A trace of bromophenol blue visualized direction of the sample movement. Settings for separation were: current 2 mA, power 2W, voltage 100V for 20 min / 200V for 80 min / 600V for 80 min. Separation yielded 5 fractions of about 650 μl representing pH ranges of 3,0 - 4,6 (fraction 1), 4,6 - 5,4 (fraction 2), 5,4 – 6,2 (fraction 3), 6,2 – 7,0 (fraction 4), 7,0 – 10 (fraction 5). To obtain similar amounts of protein for further separation by SDS PAGE protein was precipitated from 400 μl of fraction 1, 100 μl of fraction 2, 70 μl of fraction 3, 90 μl of fraction 4 and 300 μl of fraction 5 by TCA/acetone as described above.

### Preparation of samples for protein identification

#### In-solution digestion

Proteins in the individual IEF fractions were reduced, alkylated and digested in solution with trypsin. Briefly, after TCA precipitation, the dried pellet was resuspended in 20 μl 40 mM NH_4_HCO_3_. 2 μl DTT (10 mM DTT in NH_4_HCO_3_) was added to the solution and proteins were reduced at 45°C for 1 h. Free cysteine residues were alkylated with 1 μl iodoacetamide (55 mM in 40 mM NH_4_HCO_3_) for 30 min at 25°C in the dark, followed by the addition of 2.5 μl DTT. The mixture was incubated at 37°C for 15 min in a thermomixer (600 rpm). Afterwards, trypsin (1/100 w/w of precipitated protein amount) was added and proteins were digested at 37°C overnight.

#### In-gel digestion

Proteins in the individual gel slices were reduced, alkylated and in-gel digested with trypsin [14]. Briefly, after incubation with 150 μl water at 37°C for 5 min, water was removed (washing step) and gel pieces were shrunk by dehydration with 150 μl water/acetonitrile 50:50 (v/v) at 37°C for 5 min in a thermo mixer (600 rpm). The solution was removed and the proteins were reduced with 100 μl 10 mM DTT in 40 mM NH_4_HCO_3_ for 1 h at 56°C. The solution was removed and gel pieces were incubated with 150 μl water for 5 min at 37°C. After removing the solution from the gel plugs proteins were alkylated with 100 μl 55 mM iodoacetamide in 40 mM NH_4_HCO_3_ for 30 min at 25°C in the dark, followed by three alternating washing steps each with 150 μl of water and water/acetonitrile 50:50 (v/v) for 8 min at 37°C. Gel pieces were then dehydrated with 100 μl acetonitrile for 1 min at RT, dried for 15 min and subsequently rehydrated with porcine trypsin (sequencing grade, Promega, Mannheim, Germany) with a minimal volume sufficient to cover the gel pieces after rehydration (100 ng trypsin in 40 mM NH_4_HCO_3_). Samples were incubated overnight at 37°C.

#### Extraction

After overnight digestion the supernatant was collected in PCR tubes while gel pieces were subjected to four further extraction steps. Gel pieces were sonicated for 5 min in acetonitrile/0.1% aqueous TFA 50:50 (v/v). Following centrifugation the supernatant was collected and gel pieces were sonicated for 5 min in acetonitrile. After collecting the supernatant gel pieces were sonicated for 5 min in 0.1% TFA followed by another extraction step with acetonitrile. The combined solutions were dried in a speed-vac at 37°C for 2 h. Peptides were redissolved in 5 μl 0.1% TFA by sonication for 5 min and subsequently analysed by nanoLC ESI-MS/MS.

#### ESI-MS/MS analysis and database search

Tryptic peptide mixtures were separated using a nanoAcquity UPLC system. A C18 trap column (180 μm × 20 mm) with a particle size of 5 μm was used (Waters GmbH, Eschborn, Germany). Liquid chromatography separation was performed on a BEH130 C18 main-column (100 μm × 100 mm) with a particle size of 1.7 μm (Waters GmbH, Eschborn, Germany) at a flow rate of 0.4 μl / min. Gel slices 1–7, 12–17 and 22–26 were separated by a 1 h gradient whereas slices 8–11 and 18–21 were fractionated by a 2 h gradient. The 1 h gradient was set as follows: from 0 to 4% B in 1 min, from 4 to 40% B in 39 min, from 40 to 60% B in 5 min, from 60 to 85% B in 0.1 min, 6 min at 85% B, from 85 to 0% B in 0.1 min, and 9 min at 0% B. The 2 h gradient started from 0 to 4% B in 1 min, from 4% to 30% in 79 min. The next step was set from 30 to 45% B within 10 min followed by an increase in 10 min of solvent B from 45 to 90% and for a further 10 min at 90% solvent B. After this step the concentration was stepped down to 0% solvent B and continued for 15 min. Solvent A contained 98.9% water, 1% acetonitrile and 0.1% formic acid, solvent B contained 99.9% acetonitrile and 0.1% formic acid. The nanoUPLC system was coupled online to an LTQ Orbitrap XL mass spectrometer (Thermo Scientific, Bremen, Germany).Data were acquired by scan cycles of one FTMS scan with a resolution of 60000 at m/z 400 and a range from 300 to 2000 m/z in parallel with six MS/MS scans in the ion trap of the most abundant precursor ions.

The mgf-files were used for database searches with the MASCOT search engine (Matrix Science, London, UK; version 2.2) against SwissProt database (http://www.expasy.ch/sprot/sprot-top.html Rel. 2011_05). Taxonomy was set to human. The peptide mass tolerance for database searches was 10 ppm and fragment mass tolerance 0.4 Da. Carbamidomethylation of C was set as fixed modification. Variable modifications included oxidation of M and deamidation of N and Q. One missed cleavage site in case of incomplete trypsin hydrolysis was allowed. Ion score cut off was set to 20. Proteins were considered as identified if two unique peptides were detected and at least one peptide had an individual ion score exceeding the MASCOT identity threshold of 26. Identification under the applied search parameters refers to False Discovery Rate (FDR) < 3,5% and a match probability of p<0.05, where p is the probability that the observed match is a random event. We indicated proteins for which only one peptide matched the threshold of 26 by an asterisk in all tables provided in the results section as well as in the supplementary material. Each gel slice was analyzed separately and MS/MS data were merged prior to protein database search.

### Data analysis

Web-based tools for classification and functional annotation of all proteins by their GO number were used. First, we used DAVID Bioinformatics Resources (the Database for Annotation, Visualization and Integration Discovery [15,16]. The SwissProt ID list was pasted and Uniprot IDentifier was chosen to establish a gene list. The function “Functional annotation table” using GO terms (Cellular Component, Molecular Function, Biological Process, Protein Class) were chosen. Second, the classification System PANTHER (Protein ANalysis THrough Evolutionary Relationships) [17,18], Batch ID upload, was used creating a similar annotation table with the GO terms (CC, MF, BP,PC). Search procedure was again performed by using SwissProt identification numbers.

The GO terms which were in common in both tables were used for annotation of the proteins. “Secreted proteins” were annotated according to DAVID’s SP-PIR-Keywords.

Proteins which were still unknown and could not be analyzed were run through the DomainSweep pipeline which identifies the domain architecture within a protein sequence [19]. Domain hits are listed as ‘significant’ by DomainSweep

i. if two or more hits belong to the same INTERPRO family. The task compares all true positive hits of the different protein family databases grouping together those hits, which are members of the same INTERPRO family/domain.

ii. if the motif shows the same order as described in PRINTS or BLOCKS. Both databases characterize a protein family with a group of highly conserved motifs/segments in a well-defined order. The task compares the order of the identified true positive hits with the order described in the corresponding PRINTS or BLOCKS entry. Only hits in correct order are accepted.

All other hits above the trusted thresholds are listed as ‘putative’. They are significant within the domain database searched.

## Results

**Figure 1:**
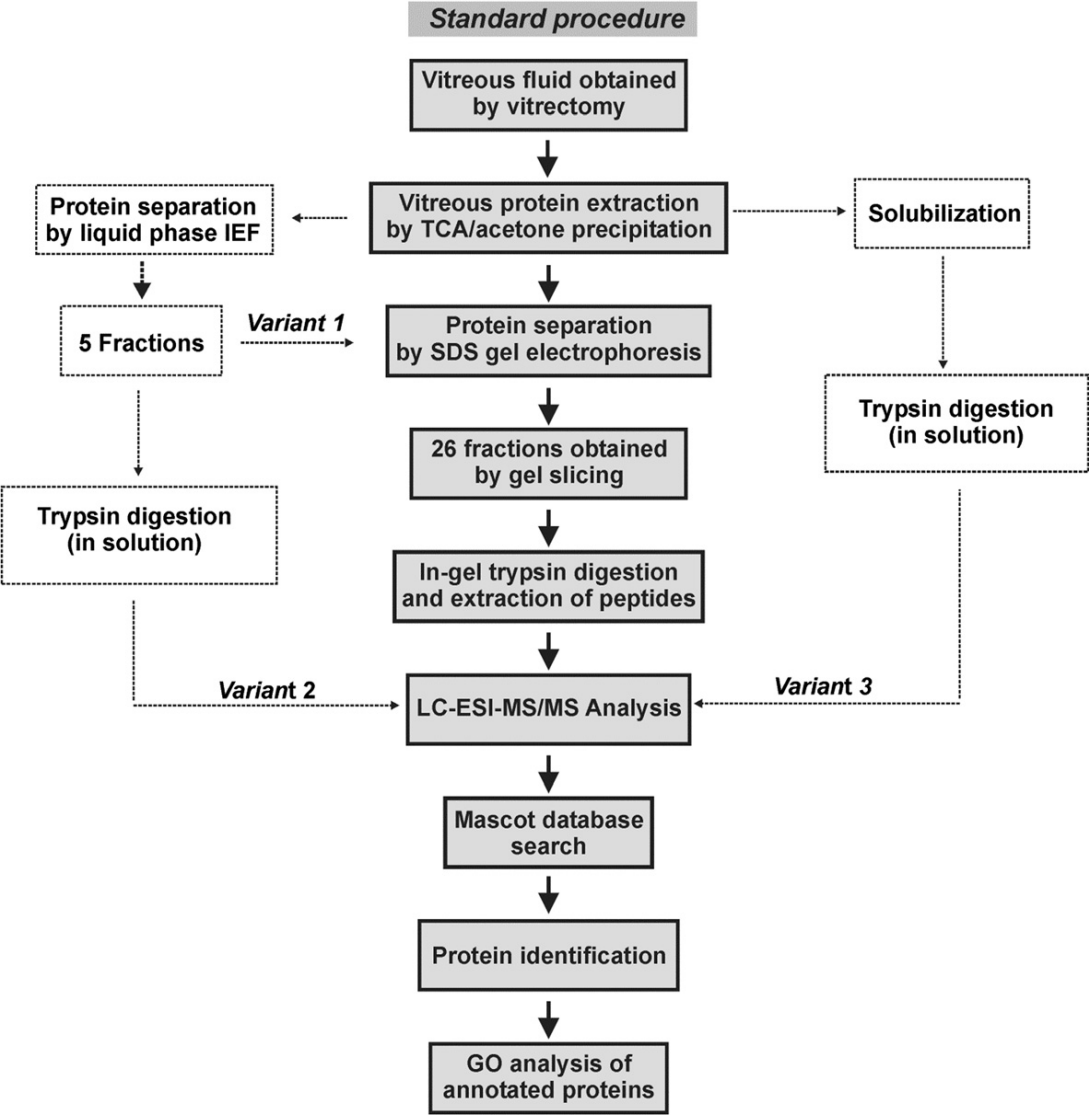
**Experimental work flow for vitreous humor proteome analysis.** The grey coloured boxes depict the work flow of the standard procedure for proteomic analysis of vitreous humor. After precipitation vitreous proteins were separated by 1D SDS gel electrophoresis. Following Coomassie staining lanes were cut into 26 slices of the same size. Proteins in each individual slice were subjected to in-gel digestion with trypsin. MS/MS data obtained by nanoLC-ESI-MS/MS analysis were searched with the MASCOT search engine against SwissProt database. In order to test alternative experimental strategies different variants of the standard procedure were applied. Variant 1: Liquid phase IEF was used as additional prefractionation step before SDS-gel electrophoresis. Variant 2: Samples obtained from liquid phase IEF were directly applied for nanoLC-ESI-MS/MS analysis. Variant 3: Vitreous proteins obtained after TCA/acetone precipitation was directly subjected to nanoLC-ESI-MS/MS analysis without any prefractionation.

**Figure 2:**
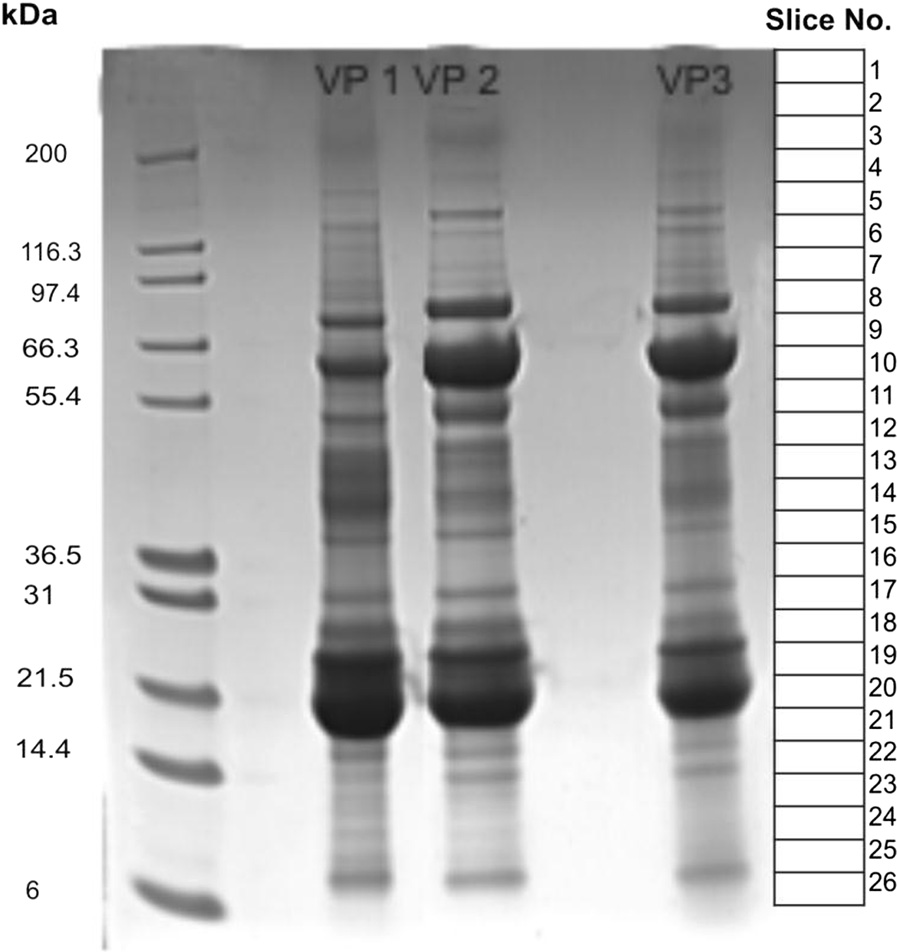
**SDS gel electrophoresis of vitreous protein samples.** Vitreous protein samples obtained from three individuals were separated on a NuPAGE^TM^ 4–12% Bis-Tris mini gel using MES running buffer, Coomassie stained, cut into 26 slices and analyzed according to the standard procedure depicted in Figure 1.

**Figure 3:**
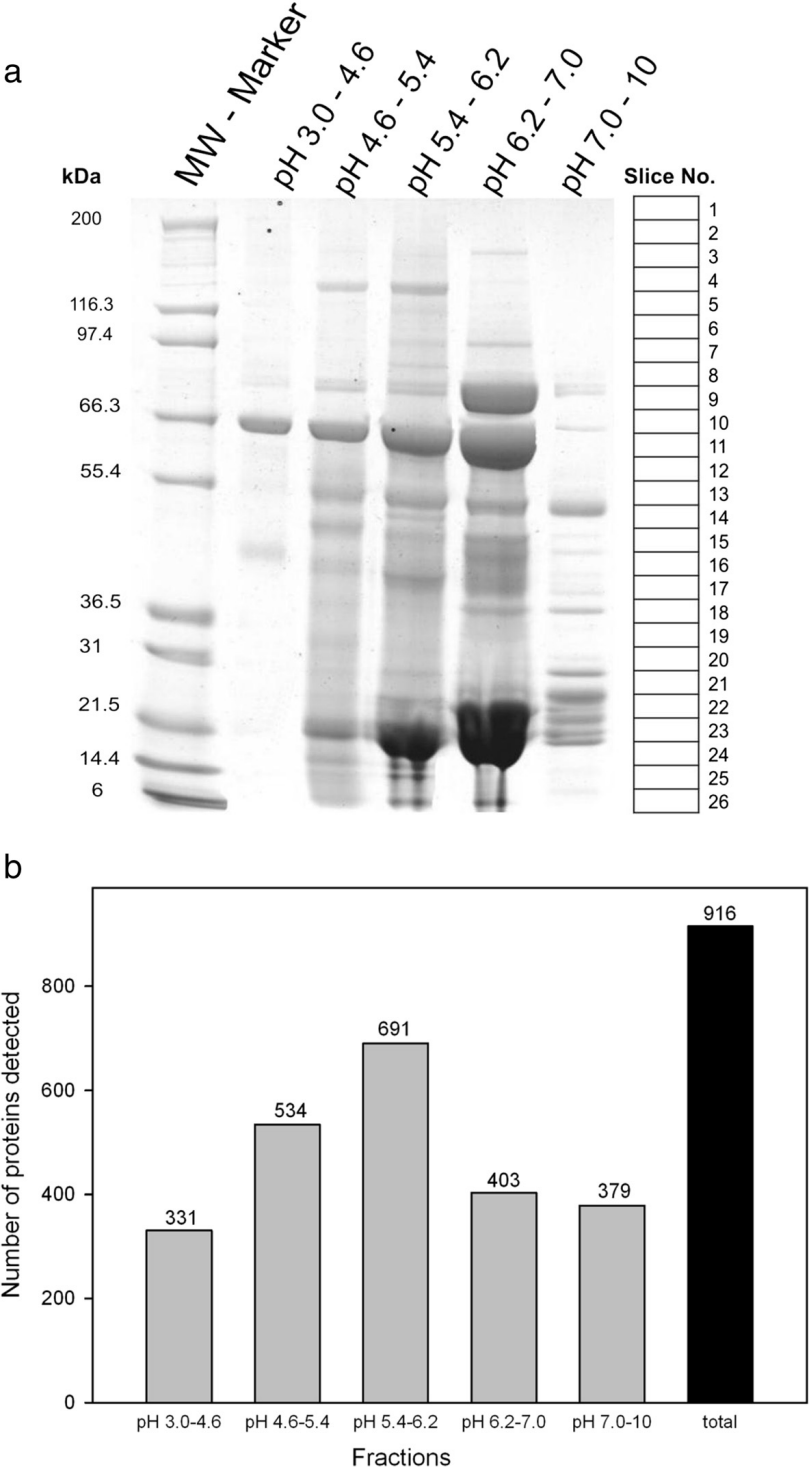
**Combined liquid phase IEF and SDS gel electrophoresis prefractionation (Variant 1). a:** Vitreous protein sample VP2 was prefractionated by liquid phase IEF in a Zoom Fractionator^TM^ (Invitrogen) yielding 5 fractions representing the indicated IP ranges. Each individual fraction was separated by SDS gel electrophoresis as described in the legend to Figure 2. The complete work flow is outlined as variant 1 in Figure 1. **b:** Overview of the total number of identified proteins in the different pH fractions obtained by variant 1. Details on the proteins are given in Additional file 1: Table S1.

**Figure 4:**
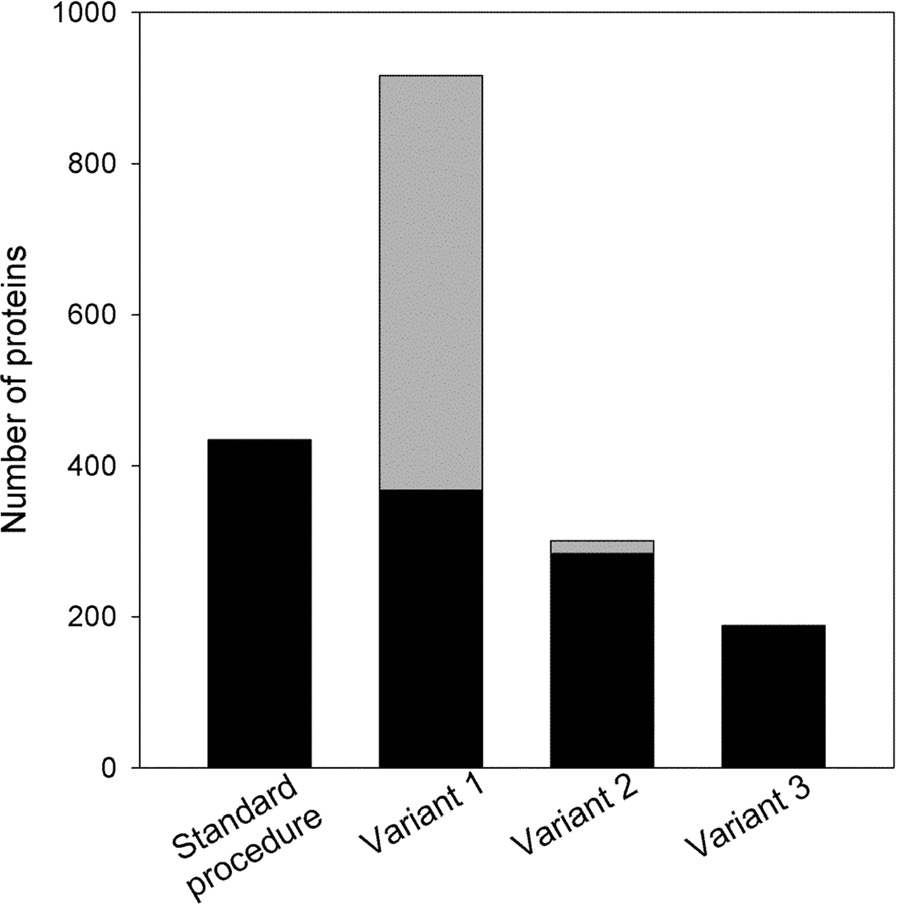
**Comparison of different prefractionation strategies.** Vitreous protein sample VP2 was analyzed with four different prefractionation strategies according to the work flow outlined in Figure 1. The bars represent the total number of proteins detected by each individual method. The black part of the bars represents proteins detectable with the standard procedure. The grey part indicates proteins that were detected with the variant method but were not found applying the standard procedure.

Complete vitreous material of three individuals (2 males, 1 female; age range 68–83 years) was collected. The three individuals were not related to each other. All were phakic at the time of operation. None was suffering from diabetes, dyslipidemia, or obesity (defined as BMI>30). Individual 1 and 2 received medical treatment for hypertension, individuals 2 and 3 for hypothyroidism. In addition, individual 1 was suffering from coronary heart disease, and individual 3 from benign prostate hyperplasia. No significant other medical conditions were present. The proteome of each individual vitreous sample (VP1, VP2, VP3) was analyzed according to the work flow (standard procedure) outlined in Figure 1. The result of protein prefractionation by SDS gel electrophoresis (standard procedure) is shown in Figure 2. 463 unique proteins were detected in VP1, 434 in VP2, and 372 in VP3. All detected proteins are listed in Additional file 1: Table S1 together with their accession number, molecular weight, isoelectric point (pI), Mascot score, number of unique peptides and sequence coverage. 242 of these proteins were found in all of the three samples, 137 in two samples, whereas 265 proteins were detected in only one of the three samples. In order to test whether additional prefractionation of the samples by liquid phase isolelectric focussing prior to SDS gel electrophoresis (variant 1 in Figure 1) may enable digging deeper into the vitreous proteome, sample VP2 was subjected to such prefractionation procedure which reduces sample complexity by resolving the proteome into 5 fractions according to the proteins’ isoelectric points. The total number of identified proteins in vitreous sample VP2 increased to 916 by liquid IEF combined with SDS PAGE, but 66 proteins detectable by the standard procedure were lost by applying such additional prefractionation (Figure 3). On the other hand, when liquid phase IEF was applied without subsequent SDS-gel electrophoresis (variant 2 in Figure 1) only 284 proteins were detectable in VP2, however among these were 17 additional proteins not detected in the standard procedure. Likewise direct analysis of the vitreous proteome without any prefraction (variant 3 in Figure 1) revealed only 186 proteins, but 2 new proteins (ABHEB and NPC2) were detected. A comparison of the different prefractionation methods is shown in Figure 4. Adding the proteins that were obviously lost during prefractionation and the low abundance proteins which became only detectable after liquid phase IEF prefractionation, the list of vitreous proteins grew to 1111 distinct protein species (Additional file 1: Table S1). Among the proteins detectable by prefractionation variant 1, 20 proteins emerged in VP2 that were also found in VP1 and VP3 by the standard procedure. Thus these proteins were added to compilation of proteins detectable in all samples yielding a total number of 262 unique proteins detectable in all individuals (Additional file 2: Table S2).

**Figure 5:**
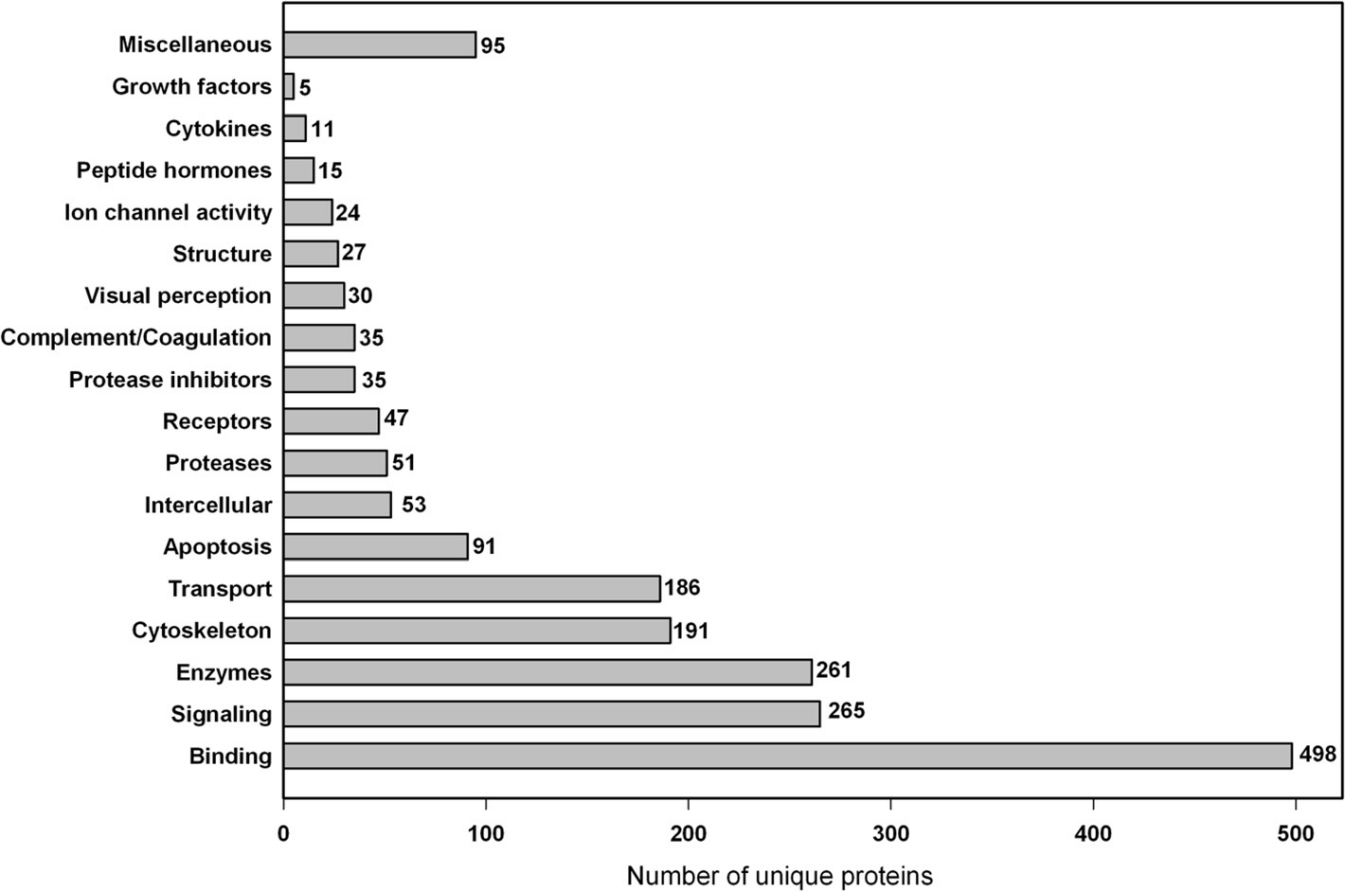
**Annotation of protein function.** Functional annotation of each individual protein was achieved by Web based tools as described in Materials and Methods. Details are given in Additional file 3: Table S3.

**Figure 6:**
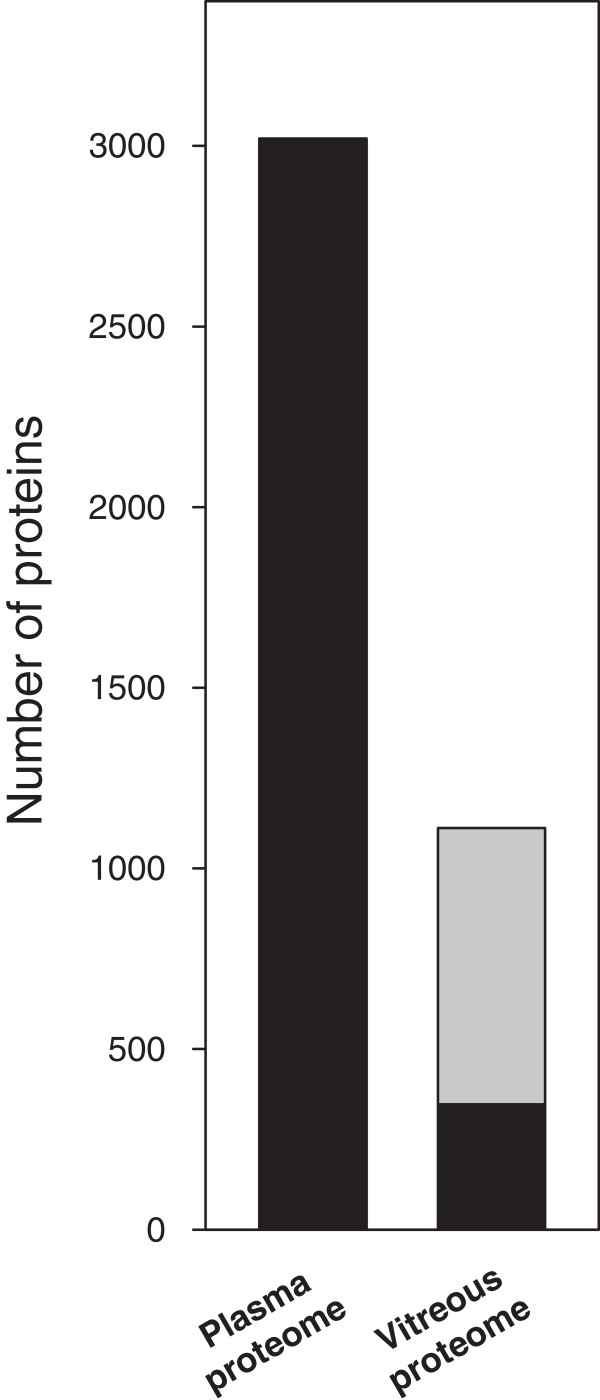
**Comparison of vitreous proteome with plasma proteome.** The list of vitreous proteins obtained in our study is compared to a map of plasma proteins from the human proteome project. The black part of the bar representing the vitreous proteome indicates proteins that are also found in the plasma proteome map. The grey part indicates proteins not listed for the plasma proteome.

Annotation of protein functions was achieved by the DAVID Bioinformatics tool using the SwissProt entries. 1105 proteins were found classified in the database, 6 are designated as uncharacterized. The result is outlined in Figure 5. Details are given in Additional file 3: Table S3. A total number of 210 proteins where annotated as secreted proteins. In order to detect specific differences between the plasma and the vitreous proteome our results were compared to a comprehensive catalogue of the plasma proteome (http://www.hupo.org), where 3020 plasma proteins were identified based on two or more peptides. Figure 6 summarizes the comparison between this map of the human plasma proteome and the vitreous proteins detected in our study. 764 proteins of the complete list of 1111 individual proteins are not listed in the HUPO plasma proteome catalogue. In Additional file 2: Table S2 and Additional file 3: Table S3 all vitreous proteins also catalogued as plasma compounds are indicated.

## Discussion

Ethical feasibility largely confines vitreous material sampling for research purposes to individuals that receive vitreous removal surgery (vitrectomy) for medical reasons, i.e. as a treatment for vitreoretinal diseases such as diabetic retinopathy. As vitrectomies are not performed in healthy persons, most previous studies on the vitreous proteome employed control groups of “surrogate normal patients” with diseases such as epiretinal gliosis, vitreomacular traction syndrome, and idiopathic macular hole that are considered to constitute only comparatively minor pathological vitreoretinal changes [9,20,21]. In our study, we examined vitreous material from three patients with epiretinal gliosis (also: epiretinal membrane, macular pucker), a disease in which retinal cells form a thin membrane on the inner retinal surface that causes visual distortions and decline [22]. Our study thus presents a catalogue of the vitreous proteome in the absence of neurodegenerative, ischemic, and vasoproliferative retinal changes associated with many other retinal diseases and provides a baseline for future comparative proteomic studies of such pathologies.

Our results clearly indicate that the protein composition of the human vitreous humor is much more complex than might be expected from previous proteome analyses. A very similar protein pattern can be expected for aqueous humor filling the anterior eye chamber between the cornea and lens, since the gel-like vitreous humor consists of 99% fluid and there is a continuous exchange between the aqueous humor and the vitreous humor located in the posterior part of the eye behind the lens. Such exchange is also reflected by the detection of the lens-specific crystallins in the vitreous humor. 262 unique proteins were detected which are present in all three patient samples indicating that these might represent the constitutive protein pattern of human vitreous. However, there also seems to exist a striking heterogeneity between the individual samples, as 236 proteins were only detectable in one sample and 196 proteins were found in two of the three specimen. Such heterogeneity may be a common feature of human biological fluids as exemplified by human plasma which also shows broad interindividual differences, in particular with regard to lower abundance proteins [23]. This should be kept in mind when the vitreous proteome of patients with eye disorders is screened for specific disease markers. In particular, some of the proteins that were not identified in at least two of the three subjects may not represent “normal” vitreous proteins. In general it is difficult to distinguish between the proteins that are actively secreted to the human vitreous and proteins introduced by low-level tissue leakage. The annotation as secreted protein given in Additional file 2: Table S2 and Additional file 3: Table S3 may serve as a rough guideline. Various keratins were detected in the samples. However, in particular the keratins 1, 2, 5, 9, 10 and 14 are known contaminants that may occur during sample preparation [24]. Since collection of the clinical samples in a keratin free environment was not feasible, these keratins might have arrived in the samples through contamination rather than through natural abundance in vitreous humor.

Although we applied a highly sensitive state-of-the-art methodology to map the vitreous proteome, only further prefractionation of the samples enabled detection of very low abundance proteins. Thus applying liquid phase isolelectric focussing as prefractionation strategy in combination with 1D SDS PAGE facilitated a striking increase in the dynamic range of detection, i.e. the number of proteins detected in sample VP2 increased from 434 for the gel separated sample to 916. Consequently liquid phase IEF might also be applied for efficient prefractionation prior to detection by other methods, including Western blotting or ELISA techniques, in studies on very low abundance proteins. On the other hand our results clearly indicate that during prefractionation either by SDS-gel electrophoresis or liquid phase IEF some proteins will be lost. Thus a combination of different prefractionation strategies is necessary to cover the complete vitreous proteome.

Comparison of the map of vitreous proteins obtained in our study with the HUPO plasma proteome indicates that the vitreous proteome strikingly differs from the plasma proteome. Thus considering the complete list of all 1111 proteins detected in the vitreous samples, only about 27% are listed also as plasma proteins. Relating to the 262 “constitutive” vitreous proteins only about 50% are known plasma proteins. Consequently the aqueous phase of human vitreous appears to be a discrete and unique body fluid with only partial overlap to other extracellular fluid body compartments. This is contradictory to the assumption that most protein species of the vitreous originate from plasma. The detection of crystallins may indicate that protein release from the lens could also contribute to the vitreous proteome. However, recent studies indicate that crystallins are also synthesized in the retina. Crystallins may have several metabolic and regulatory functions. Their synthesis is dramatically up regulated by a large range of diseases, including diabetic retinopathy, age-related macular degeneration, uveitis, trauma and ischemia. Further investigations on the origin as well as on normal and pathological functions of crystallins in the vitreous may help to understand the aetiology of the above mentioned disorders [25]. The detection of dermcidin, an antimicrobial peptide commonly considered to be a primitive mechanism of immunity, in human vitreous is another interesting finding [26]. Recently this peptide was also identified in tear fluid [27]. Galectin-1 is an example of a protein with potential adhesion/growth regulatory functions in the eye [28], but has not been annotated as a plasma protein. Lengsin is an eye lens-specific member of the glutamine synthetase superfamily [29]. Opticin is a classical vitreous protein, where it is associated with humor collagen fibrils [30]. Some of the detected proteins such as retinoschisin and retbindin are specifically secreted by photoreceptor cells [31,32] which indicates that cells of all retinal layers including the outer retina contribute proteins to the vitreous proteome. A previous analysis of the secretome of cultured human RPE indicates that RPE cells secrete a variety of proteins (73 individual proteins) including extracellular matrix proteins, cell adhesion proteins, complement factors, proteases, protease inhibitors, enzymes and growth factors, that may be involved in normal functions of the eye as well as in the pathogenesis of eye disorders [33]. Therefore a comprehensive in-depth analysis of the RPE secretome and comparison to the vitreous proteome will help to assess the contribution of the RPE to the vitreous proteome composition and function.

## Conclusions

Human vitreous appears to be a discrete and unique body fluid with only partial overlap to the plasma proteome. Our findings suggest that vitreous proteome analysis may prove useful in examining all retinal diseases, even those primarily affecting the outer retina such as age-related macular degeneration and most hereditary retinal dystrophies. Altogether this study provides the most comprehensive human vitreous proteome coverage and list reported to date. The protein set has immediate utility for investigators interested in using vitreous samples to study eye disorders, including widespread diseases like age-related macular degeneration or diabetic retinopathies.

In addition a comparison of different prefractionation methods for comprehensive proteome analysis of a largely uncharacterized human body fluid is given with the example of human vitreous, indicating that human vitreous contains a variety of low abundance proteins that were only detectable by employing efficient prefractionation methods like liquid phase isoelectric focussing.

## Competing interests

The authors declare that they have no competing interests.

## Authors’ contributions

SA was involved in all steps of the proteomic analyses. TUK and BVS collected, processed and characterized the clinical samples. KK and UW participated in mass spectrometric analysis. AHW was involved in the bioinformatic analysis of the data. MB and TK participated in the sample preparation for mass spectrometric analysis. FGH, MS, JK designed and coordinated the study, and drafted the manuscript. All authors read and approved the manuscript.

## Supplementary Material

Additional file 1: Table 1 Proteins found in any of the three samples. All vitreous humor proteins detected in our study are compiled in alphabetical order. A protein was considered as identified if two peptides were detected with ion score cut off set to 20 and at least one peptide had an individual ion score exceeding the MASCOT identity threshold above 26. Mascot scores for all identified proteins in relation to the sample and analytic procedure are given. If no score is given the protein was not detected in this patient sample or by this work-up procedure. Click here for file

Additional file 2: Table S2 Proteins found in vitreous protein samples from all three patients. Click here for file

Additional file 3: Table S3 Functional annotation of vitreous proteins. Classification and functional annotation was conducted as described in Materials and Methods. P: proteins catalogued or annotated as plasma proteins; S: predicted as secreted proteins. The Roman numerals indicate the following annotations; I: Enzymes, II: Proteases, III: Protease Inhibitors, IV: Complement and coagulation system, V: Growths factors, VI: Cytokines, VII: Peptide hormones, VIII: Transport, IX: Receptors, X: Structure, XI: Visual perception, XII: Ion channel activity, XIII: Binding, XIV: Apoptosis, XV: Cytoskeleton, XVI: Signaling, XVII: Intercellular, XVIII: Miscellaneous Click here for file
