# Supplementary material for: In-depth mass spectrometric mapping of the human vitreous proteome
Source: Proteome Sci. 2013 May 20;11:22. doi: 10.1186/1477-5956-11-22 (PMC3689628; doi:10.1186/1477-5956-11-22)
Supplement: Additional file 1: Table 1 — Proteins found in any of the three samples. All vitreous humor proteins detected in our study are compiled in alphabetical order. A protein was considered as identified if two peptides were detected with ion score cut off set to 20 and at least one peptide had an individual ion score exceeding the MASCOT identity threshold above 26. Mascot scores for all identified proteins in relation to the sample and analytic procedure are given. If no score is given the protein was not detected in this patient sample or by this work-up procedure. [file 1477-5956-11-22-S1.doc]

**Supplementary table 1: Proteins found in any of the three samples**

**All vitreous humor proteins detected in our study are compiled in alphabetical order. A protein was considered as identified if two peptides were detected with ion score cut off set to 20 and at least one peptide had an individual ion score exceeding the MASCOT identity threshold above 26. Mascot scores for all identified proteins in relation to the sample and analytic procedure are given. If no score is given the protein was not detected in this patient sample or by this work-up procedure.**

|  | | | | **Standard Procedure** | | | | | | | | | **VP2** | | | | | | | | |
| --- | --- | --- | --- | --- | --- | --- | --- | --- | --- | --- | --- | --- | --- | --- | --- | --- | --- | --- | --- | --- | --- |
|  |  |  |  | **VP1** | | | **VP2** | | | **VP3** | | | **Variant 1** | | | **Variant 2** | | | **Variant 3** | | |
| **Protein Name** | **ACC Number** | **Mass**  **[Da]** | **pI** | **Score** | **Seq.Cov** | **No.**  **pep** | **Score** | **Seq.**  **Cov** | **No.**  **pep** | **Score** | **Seq.**  **Cov** | **No.**  **pep** | **Score** | **Seq.Cov** | **No.**  **pep** | **Score** | **Seq.**  **Cov** | **No.**  **pep** | **Score** | **Seq.**  **Cov** | **No.**  **pep** |
| 14-3-3 protein beta/alpha | 1433B_HUMAN | 28179 | 4.76 | 602.18 | 43.9 | 11 | 437 | 35.8 | 9 | 563.27 | 41.5 | 10 | 809.34 | 54.5 | 13 | 483.52 | 38.6 | 10 |  |  |  |
| 14-3-3 protein epsilon | 1433E_HUMAN | 29326 | 4.63 | 881.36 | 57.6 | 15 | 892.27 | 60.8 | 18 | 812.49 | 57.3 | 14 | 1272.99 | 68.6 | 20 | 1009.4 | 52.2 | 16 | 461.41 | 31.4 | 7 |
| 14-3-3 protein eta | 1433F_HUMAN | 28372 | 4.76 | 403.04 | 28.9 | 8 | 488.52 | 35 | 10 | 425.39 | 38.6 | 9 | 919.22 | 58.1 | 15 | 533.27 | 40.7 | 10 |  |  |  |
| 14-3-3 protein gamma | 1433G_HUMAN | 28456 | 4.80 | 707.1 | 53.4 | 13 | 647.03 | 57.1 | 13 | 707.51 | 50.2 | 11 | 973.32 | 74.1 | 18 | 850.38 | 60.7 | 14 | 193.13 | 15.4 | 4 |
| 14-3-3 protein sigma | 1433S_HUMAN | 27871 | 4.69 |  |  |  | 420.02 | 27.4 | 7 |  |  |  | 324.89 | 26.2 | 7 |  |  |  |  |  |  |
| 14-3-3 protein theta | 1433T_HUMAN | 28032 | 4.68 | 415.8 | 29 | 9 | 400.48 | 29.4 | 8 | 364.51 | 27.8 | 8 | 694.06 | 53.9 | 13 |  |  |  |  |  |  |
| 14-3-3 protein zeta/delta | 1433Z_HUMAN | 27899 | 4.74 | 819.28 | 62.9 | 15 | 739.37 | 59.6 | 14 | 830.12 | 59.6 | 15 | 1162.84 | 65.3 | 19 | 744.66 | 56.3 | 12 | 345.33 | 30.2 | 7 |
| 1-phosphatidylinositol 4.5-bisphosphate phosphodiesterase eta-1* | PLCH1_HUMAN | 191214 | 7.87 |  |  |  |  |  |  |  |  |  | 58.42 | 1.1 | 2 |  |  |  |  |  |  |
| 26S protease regulatory subunit 6A | PRS6A_HUMAN | 49458 | 5.13 |  |  |  |  |  |  |  |  |  | 182.45 | 10.7 | 3 |  |  |  |  |  |  |
| 26S proteasome non-ATPase regulatory subunit 11* | PSD11_HUMAN | 47719 | 6.08 |  |  |  |  |  |  |  |  |  | 74.49 | 6.9 | 2 |  |  |  |  |  |  |
| 26S proteasome non-ATPase regulatory subunit 13 | PSD13_HUMAN | 43203 | 5.53 |  |  |  |  |  |  |  |  |  | 64.17 | 9.8 | 3 |  |  |  |  |  |  |
| 26S proteasome non-ATPase regulatory subunit 2 | PSMD2_HUMAN | 100877 | 5.09 | 107.26 | 3 | 2 | 57.67 | 1.9 | 2 |  |  |  | 172.37 | 8.6 | 5 |  |  |  |  |  |  |
| 3-hydroxyanthranilate 3.4-dioxygenase | 3HAO_HUMAN | 32707 | 5.62 |  |  |  |  |  |  |  |  |  | 273.42 | 26.2 | 6 |  |  |  |  |  |  |
| 40S ribosomal protein SA* | RSSA_HUMAN | 32947 | 4.79 |  |  |  |  |  |  |  |  |  | 63.69 | 7.1 | 2 |  |  |  |  |  |  |
| 4-hydroxyphenylpyruvate dioxygenase | HPPD_HUMAN | 45077 | 6.52 | 742.1 | 49.1 | 15 | 424.02 | 33.1 | 11 | 399.32 | 23.7 | 9 | 765.83 | 49.4 | 16 | 117.87 | 6.6 | 2 | 126.17 | 9.2 | 3 |
| 4-trimethylaminobutyraldehyde dehydrogenase | AL9A1_HUMAN | 54679 | 5.70 | 260.22 | 14.6 | 7 | 211.17 | 11.1 | 5 | 157.1 | 8.7 | 4 | 459.05 | 20.4 | 10 | 52.73 | 3.8 | 2 | 63.07 | 4.9 | 2 |
| 6-phosphofructokinase type C | K6PP_HUMAN | 86454 | 7.50 |  |  |  |  |  |  |  |  |  | 129.35 | 4.7 | 4 |  |  |  | 89.45 | 3.3 | 2 |
| 6-phosphofructokinase. liver type | K6PL_HUMAN | 85762 | 7.26 | 811.17 | 25.4 | 17 | 469.44 | 15 | 11 | 219.08 | 8.1 | 5 | 553.31 | 16.3 | 12 | 259.43 | 7.3 | 5 | 300.66 | 12.1 | 7 |
| 6-phosphofructokinase. muscle type | K6PF_HUMAN | 85984 | 8.23 | 127.35 | 4.9 | 3 | 189.48 | 6.7 | 5 |  |  |  |  |  |  |  |  |  |  |  |  |
| 6-phosphogluconate dehydrogenase. decarboxylating | 6PGD_HUMAN | 53619 | 6.80 | 826.94 | 35.4 | 15 | 366.55 | 14.3 | 6 | 327.88 | 14.9 | 5 | 953.77 | 36.4 | 16 |  |  |  |  |  |  |
| 6-phosphogluconolactonase | 6PGL_HUMAN | 27815 | 5.70 | 153.83 | 28.7 | 4 | 191.65 | 29.5 | 5 |  |  |  | 332.69 | 39.5 | 6 |  |  |  |  |  |  |
| 72 kDa type IV collagenase | MMP2_HUMAN | 74918 | 5.26 | 186.29 | 6.8 | 4 |  |  |  | 102.12 | 4.7 | 2 | 379.31 | 16.5 | 9 |  |  |  |  |  |  |
| A disintegrin and metalloproteinase with thrombospondin motifs 5* | ATS5_HUMAN | 104048 | 9.14 |  |  |  |  |  |  |  |  |  | 48.35 | 1.5 | 2 |  |  |  |  |  |  |
| Abhydrolase domain-containing protein 14B | ABHEB_HUMAN | 22446 | 5.94 |  |  |  |  |  |  |  |  |  |  |  |  |  |  |  | 59.59 | 15.2 | 2 |
| Abnormal spindle-like microcephaly-associated protein | ASPM_HUMAN | 413189 | 10.45 | 91.11 | 0.7 | 3 | 64.5 | 0.4 | 2 |  |  |  | 61.98 | 0.3 | 2 |  |  |  |  |  |  |
| Acetyl-CoA acetyltransferase. cytosolic | THIC_HUMAN | 41838 | 6.47 | 114.53 | 15.4 | 3 | 130.15 | 11.6 | 3 | 131.31 | 14.1 | 4 | 98.15 | 8.8 | 2 |  |  |  |  |  |  |
| Acetyl-CoA carboxylase 1 | ACACA_HUMAN | 267095 | 5.96 |  |  |  |  |  |  |  |  |  | 66.36 | 1 | 3 |  |  |  |  |  |  |
| Acid ceramidase | ASAH1_HUMAN | 45087 | 7.52 |  |  |  |  |  |  |  |  |  | 228.61 | 18.2 | 6 |  |  |  |  |  |  |
| Actin. alpha cardiac muscle 1 | ACTC_HUMAN | 42334 | 5.23 | 713.57 | 31.8 | 13 | 735.02 | 31.8 | 13 | 634.09 | 35 | 12 | 891.8 | 56.5 | 18 | 633.89 | 33.2 | 12 | 673.92 | 45.6 | 15 |
| Actin. cytoplasmic 1 | ACTB_HUMAN | 42052 | 5.29 | 1414.25 | 67.7 | 22 | 1298.11 | 68.8 | 23 | 1209.6 | 67.5 | 19 | 1389.81 | 81.6 | 23 | 1154.92 | 67.5 | 19 | 1104.39 | 81.3 | 22 |
| Actin-related protein 2 | ARP2_HUMAN | 45017 | 6.30 |  |  |  |  |  |  |  |  |  | 90.95 | 6.3 | 3 |  |  |  |  |  |  |
| Actin-related protein 2/3 complex subunit 4 | ARPC4_HUMAN | 19768 | 8.53 |  |  |  | 83.28 | 11.3 | 2 |  |  |  |  |  |  | 58.01 | 17.3 | 2 |  |  |  |
| Actin-related protein 3 | ARP3_HUMAN | 47797 | 5.62 |  |  |  |  |  |  |  |  |  | 160.24 | 11.7 | 4 |  |  |  |  |  |  |
| Actin-related protein 3B | ARP3B_HUMAN | 48090 | 5.62 | 101.39 | 4.8 | 2 |  |  |  |  |  |  |  |  |  |  |  |  |  |  |  |
| Acylamino-acid-releasing enzyme | ACPH_HUMAN | 82142 | 5.30 | 242.76 | 8.1 | 5 | 130.23 | 4.9 | 3 |  |  |  | 800.97 | 26.4 | 15 | 160.4 | 4.6 | 3 | 80.41 | 2.9 | 2 |
| Acylphosphatase-2 | ACYP2_HUMAN | 11190 | 9.52 |  |  |  |  |  |  |  |  |  | 124.05 | 30.3 | 3 |  |  |  |  |  |  |
| Adenosylhomocysteinase | SAHH_HUMAN | 48255 | 5.92 | 705.83 | 35.9 | 14 | 537.44 | 29.6 | 11 | 832.16 | 41.4 | 16 | 740.15 | 36.8 | 14 | 175.62 | 8.6 | 3 | 154.64 | 10.4 | 4 |
| Adenylosuccinate lyase | PUR8_HUMAN | 55595 | 6.69 | 137.41 | 8.5 | 3 | 80.9 | 6.2 | 2 | 74.26 | 5.8 | 2 | 193.26 | 11.6 | 5 |  |  |  |  |  |  |
| Adenylyl cyclase-associated protein 1* | CAP1_HUMAN | 52325 | 8.24 |  |  |  |  |  |  |  |  |  | 51.34 | 4.2 | 2 |  |  |  |  |  |  |
| Adenylyl cyclase-associated protein 2* | CAP2_HUMAN | 53076 | 5.95 |  |  |  |  |  |  |  |  |  | 58.8 | 4 | 2 |  |  |  |  |  |  |
| ADP-ribosylation factor 1 | ARF1_HUMAN | 20741 | 6.32 | 153.14 | 21.5 | 4 | 159.02 | 27.1 | 4 |  |  |  |  |  |  |  |  |  |  |  |  |
| ADP-ribosylation factor-like protein 3 | ARL3_HUMAN | 20614 | 6.74 | 107.8 | 20.9 | 2 |  |  |  |  |  |  |  |  |  |  |  |  |  |  |  |
| ADP-sugar pyrophosphatase | NUDT5_HUMAN | 24597 | 4.87 |  |  |  |  |  |  |  |  |  | 367.48 | 31.1 | 7 | 199.22 | 19.2 | 4 |  |  |  |
| Afamin | AFAM_HUMAN | 70963 | 5.64 | 1030.59 | 41.7 | 24 | 1046.83 | 39.9 | 23 | 647.21 | 29.4 | 16 | 1411.84 | 43.2 | 28 | 188.32 | 7.7 | 5 | 168.21 | 8.3 | 5 |
| Aflatoxin B1 aldehyde reductase member 2 | ARK72_HUMAN | 40020 | 6.70 |  |  |  |  |  |  |  |  |  | 114.31 | 7.5 | 2 |  |  |  |  |  |  |
| Agrin | AGRIN_HUMAN | 222861 | 6.02 | 449.31 | 7.5 | 12 |  |  |  | 110.79 | 1.3 | 4 | 248.68 | 4.2 | 8 |  |  |  |  |  |  |
| Alanine--tRNA ligase. cytoplasmic | SYAC_HUMAN | 107484 | 5.34 |  |  |  |  |  |  |  |  |  | 158.6 | 5.3 | 5 |  |  |  |  |  |  |
| Alcohol dehydrogenase [NADP(+)] | AK1A1_HUMAN | 36892 | 6.32 |  |  |  |  |  |  |  |  |  | 241.19 | 15.7 | 4 |  |  |  |  |  |  |
| Aldose 1-epimerase | GALM_HUMAN | 37970 | 6.19 |  |  |  |  |  |  | 120.12 | 6.7 | 2 | 401.19 | 23.7 | 7 |  |  |  |  |  |  |
| Aldose reductase | ALDR_HUMAN | 36230 | 6.51 | 735.76 | 73.7 | 18 | 609.77 | 57.3 | 15 | 95.72 | 5.4 | 2 | 574.57 | 51.9 | 14 | 227.23 | 22.2 | 6 | 382.2 | 38.9 | 10 |
| Alpha-1-acid glycoprotein 1 | A1AG1_HUMAN | 23725 | 4.93 |  |  |  | 416.38 | 44.8 | 8 | 469.68 | 41.3 | 8 | 635.53 | 49.3 | 10 | 726.8 | 46.8 | 12 | 55.35 | 11.9 | 2 |
| Alpha-1-acid glycoprotein 2 | A1AG2_HUMAN | 23873 | 5.04 |  |  |  | 289.03 | 39.3 | 6 | 287.66 | 26.4 | 5 | 610.92 | 48.3 | 10 | 676.12 | 46.8 | 11 | 57.82 | 9.5 | 2 |
| Alpha-1-antichymotrypsin | AACT_HUMAN | 47792 | 5.33 | 921.15 | 45.9 | 18 | 876.71 | 43.3 | 16 | 1210.57 | 50.4 | 20 | 1476.02 | 55.3 | 22 | 1571.85 | 56 | 25 | 850.65 | 45.2 | 18 |
| Alpha-1-antitrypsin | A1AT_HUMAN | 46878 | 5.37 | 1934.83 | 67.7 | 32 | 2023.89 | 67.2 | 31 | 2060.95 | 71.3 | 32 | 2246.93 | 71.3 | 34 | 1666.24 | 67.2 | 28 | 1689.72 | 65.6 | 29 |
| Alpha-1B-glycoprotein | A1BG_HUMAN | 54790 | 5.56 | 872.95 | 57.2 | 16 | 797.53 | 44.2 | 13 | 874.25 | 45.5 | 14 | 890.57 | 51.5 | 14 | 419.22 | 29.5 | 10 | 607.91 | 31.3 | 10 |
| Alpha-2-antiplasmin | A2AP_HUMAN | 54873 | 5.87 | 355.6 | 24.4 | 8 | 197.09 | 15.9 | 4 | 191.77 | 11.4 | 4 | 394.12 | 30.8 | 10 | 222.11 | 21.6 | 6 | 94.29 | 13.6 | 3 |
| Alpha-2-HS-glycoprotein | FETUA_HUMAN | 40098 | 5.44 | 473.05 | 37.6 | 9 | 541.19 | 44.1 | 10 | 444.35 | 32.7 | 8 | 721.12 | 45.8 | 12 | 562.87 | 28.3 | 9 | 408.44 | 35.4 | 9 |
| Alpha-2-macroglobulin | A2MG_HUMAN | 164613 | 6.04 | 3505.44 | 53.8 | 63 | 4132.5 | 54.3 | 65 | 3482.08 | 52.6 | 58 | 3403.23 | 46.9 | 55 | 741.8 | 13.6 | 14 | 1363.26 | 28.2 | 31 |
| Alpha-actinin-1 | ACTN1_HUMAN | 103563 | 5.25 | 1033.63 | 29.4 | 21 | 323.23 | 8.4 | 6 | 52.85 | 3 | 2 | 551.59 | 16.8 | 13 |  |  |  | 80.06 | 2.8 | 2 |
| Alpha-actinin-4 | ACTN4_HUMAN | 105245 | 5.28 | 541.52 | 11.2 | 10 |  |  |  |  |  |  | 392.64 | 9.7 | 8 |  |  |  |  |  |  |
| Alpha-centractin | ACTZ_HUMAN | 42701 | 6.20 | 71.36 | 6.1 | 2 |  |  |  |  |  |  | 158.88 | 8.8 | 2 |  |  |  |  |  |  |
| Alpha-crystallin A chain | CRYAA_HUMAN | 20011 | 5.77 | 1326.95 | 94.2 | 20 | 1423.93 | 92.5 | 20 | 1246.4 | 94.2 | 20 | 1414.33 | 97.1 | 22 | 1332.94 | 94.2 | 20 | 1302.79 | 94.8 | 22 |
| Alpha-crystallin B chain | CRYAB_HUMAN | 20146 | 6.77 | 1143.53 | 96 | 19 | 1454.38 | 97.1 | 23 | 1247.83 | 94.9 | 21 | 1379.74 | 97.1 | 24 | 1285.59 | 94.9 | 20 | 1051.17 | 95.4 | 18 |
| Alpha-enolase | ENOA_HUMAN | 47481 | 7.01 | 1904.56 | 77.9 | 31 | 1634.84 | 62.7 | 26 | 1584.75 | 67.1 | 24 | 2015.02 | 74 | 31 | 1152.62 | 53 | 19 | 1017.96 | 61.5 | 20 |
| Alpha-mannosidase 2C1 | MA2C1_HUMAN | 116959 | 6.10 |  |  |  |  |  |  |  |  |  | 312.16 | 7 | 7 |  |  |  |  |  |  |
| Alpha-soluble NSF attachment protein | SNAA_HUMAN | 33667 | 5.23 |  |  |  |  |  |  |  |  |  | 480.83 | 42.4 | 10 | 156.79 | 11.5 | 3 |  |  |  |
| Amyloid beta A4 protein | A4_HUMAN | 87914 | 4.74 | 605.15 | 19.6 | 14 | 608.23 | 20.1 | 13 | 514.15 | 15.6 | 10 | 362.39 | 15.1 | 10 | 614.99 | 19.7 | 12 | 122.09 | 3.1 | 3 |
| Amyloid-like protein 1 | APLP1_HUMAN | 72815 | 5.55 |  |  |  | 242.09 | 12 | 6 | 197.18 | 10 | 4 | 487.37 | 21.5 | 12 | 391.11 | 17.4 | 9 |  |  |  |
| Amyloid-like protein 2 | APLP2_HUMAN | 87927 | 4.74 | 962.96 | 32.9 | 21 | 1168.13 | 30.8 | 20 | 884.8 | 26.1 | 17 | 831.17 | 24.8 | 17 | 881.62 | 27.4 | 17 | 532.65 | 17.6 | 11 |
| Angiotensinogen | ANGT_HUMAN | 53406 | 5.87 | 599.35 | 26 | 10 | 418.94 | 21.6 | 7 | 767.59 | 39.4 | 12 | 1028 | 39.4 | 13 | 576.3 | 30.3 | 9 | 465.07 | 26.8 | 9 |
| Ankyrin repeat and SOCS box protein 2 | ASB2_HUMAN | 65898 | 7.55 |  |  |  |  |  |  |  |  |  | 87.36 | 4.6 | 3 |  |  |  |  |  |  |
| Ankyrin repeat domain-containing protein 17 | ANR17_HUMAN | 275970 | 6.07 |  |  |  |  |  |  |  |  |  | 58.24 | 0.5 | 2 |  |  |  |  |  |  |
| Ankyrin repeat domain-containing protein 20A1* | A20A1_HUMAN | 94902 | 7.97 |  |  |  |  |  |  |  |  |  | 47.24 | 1.9 | 2 |  |  |  |  |  |  |
| Ankyrin repeat domain-containing protein 30B | AN30B_HUMAN | 169256 | 5.88 |  |  |  |  |  |  |  |  |  | 72.45 | 1.4 | 3 |  |  |  |  |  |  |
| Ankyrin repeat domain-containing protein 35 | ANR35_HUMAN | 110811 | 5.76 |  |  |  |  |  |  |  |  |  | 67.36 | 3.4 | 3 |  |  |  |  |  |  |
| Ankyrin-1 | ANK1_HUMAN | 207334 | 5.65 |  |  |  |  |  |  |  |  |  | 57.95 | 0.7 | 2 | 61.44 | 0.9 | 2 |  |  |  |
| Ankyrin-2 | ANK2_HUMAN | 432499 | 5.02 | 330.95 | 2.1 | 8 | 218.93 | 1.2 | 5 | 67.78 | 0.5 | 2 | 52.86 | 0.4 | 2 |  |  |  |  |  |  |
| Ankyrin-3 | ANK3_HUMAN | 482394 | 6.07 | 100.84 | 0.7 | 3 |  |  |  |  |  |  |  |  |  |  |  |  |  |  |  |
| Annexin A1 | ANXA1_HUMAN | 38918 | 6.57 |  |  |  | 306.67 | 16.2 | 5 |  |  |  | 748.26 | 42.2 | 13 |  |  |  |  |  |  |
| Annexin A2 | ANXA2_HUMAN | 38808 | 7.58 |  |  |  | 172.87 | 15 | 4 | 178.34 | 12.7 | 4 | 618.95 | 44.8 | 13 |  |  |  |  |  |  |
| Annexin A3 | ANXA3_HUMAN | 36524 | 5.63 |  |  |  |  |  |  |  |  |  | 468.62 | 34.4 | 10 |  |  |  |  |  |  |
| Annexin A5 | ANXA5_HUMAN | 35971 | 4.94 |  |  |  | 97.65 | 5.6 | 2 | 250.72 | 12.5 | 3 | 631.5 | 40 | 12 | 85.5 | 7.2 | 2 |  |  |  |
| Antigen KI-67 | KI67_HUMAN | 360698 | 9.49 |  |  |  |  |  |  |  |  |  | 65.4 | 0.8 | 3 |  |  |  |  |  |  |
| Antileukoproteinase | SLPI_HUMAN | 15228 | 9.11 |  |  |  |  |  |  |  |  |  | 201.19 | 39.4 | 6 |  |  |  |  |  |  |
| Antithrombin-III | ANT3_HUMAN | 53025 | 6.32 | 1074.7 | 49.1 | 22 | 718.79 | 40.3 | 15 | 1013.66 | 50.4 | 21 | 1163.54 | 48.7 | 22 | 722.47 | 33.2 | 11 | 561.14 | 31.7 | 12 |
| Apolipoprotein A-I | APOA1_HUMAN | 30759 | 5.57 | 1209.49 | 68.9 | 22 | 1020.74 | 62.5 | 18 | 1171.54 | 71.2 | 23 | 863.23 | 50.6 | 14 | 821.86 | 59.6 | 16 | 1014.41 | 62.9 | 18 |
| Apolipoprotein A-II | APOA2_HUMAN | 11282 | 6.26 | 203.84 | 41 | 4 | 135.13 | 21 | 3 | 105.47 | 19 | 2 |  |  |  | 116.19 | 41 | 3 | 223.41 | 41 | 4 |
| Apolipoprotein A-IV | APOA4_HUMAN | 45371 | 5.29 | 926.41 | 47.5 | 19 | 839.41 | 47 | 16 | 335.24 | 22.2 | 7 | 1196.05 | 57.1 | 23 | 234.53 | 18.2 | 7 | 417.82 | 30.1 | 9 |
| Apolipoprotein B-100 | APOB_HUMAN | 516651 | 6.58 | 111.14 | 0.9 | 4 |  |  |  |  |  |  |  |  |  |  |  |  |  |  |  |
| Apolipoprotein D | APOD_HUMAN | 21547 | 5.07 | 74.47 | 11.6 | 2 |  |  |  |  |  |  | 150.42 | 21.2 | 4 |  |  |  |  |  |  |
| Apolipoprotein E | APOE_HUMAN | 36246 | 5.65 | 1047.79 | 55.8 | 18 | 753.23 | 40.1 | 12 |  |  |  | 1327.17 | 74.4 | 23 | 489.92 | 37.9 | 11 | 596.46 | 44.2 | 11 |
| Arf-GAP with Rho-GAP domain. ANK repeat and PH domain-containing protein 2 | ARAP2_HUMAN | 195098 | 7.12 |  |  |  |  |  |  |  |  |  | 71.61 | 1.2 | 3 |  |  |  |  |  |  |
| Arginase-1 | ARGI1_HUMAN | 34884 | 6.72 |  |  |  | 187.96 | 12.7 | 3 | 81.59 | 6.8 | 2 | 182.92 | 13 | 4 |  |  |  |  |  |  |
| Aspartate aminotransferase. cytoplasmic | AATC_HUMAN | 46447 | 6.52 |  |  |  |  |  |  |  |  |  | 114.08 | 10.4 | 3 |  |  |  |  |  |  |
| Aspartate aminotransferase. mitochondrial | AATM_HUMAN | 47886 | 9.15 |  |  |  |  |  |  |  |  |  | 72.52 | 3.3 | 2 |  |  |  |  |  |  |
| Astrocytic phosphoprotein PEA-15 | PEA15_HUMAN | 15088 | 4.94 |  |  |  |  |  |  |  |  |  | 210.08 | 30 | 4 |  |  |  |  |  |  |
| Ataxin-2 | ATX2_HUMAN | 140823 | 9.60 | 60.55 | 1.2 | 2 |  |  |  |  |  |  |  |  |  |  |  |  |  |  |  |
| ATPase family AAA domain-containing protein 3A* | ATD3A_HUMAN | 71610 | 9.08 |  |  |  |  |  |  |  |  |  | 55.32 | 2.4 | 2 |  |  |  |  |  |  |
| ATP-binding cassette sub-family A member 12 | ABCAC_HUMAN | 295387 | 7.89 |  |  |  |  |  |  | 55.52 | 0.5 | 2 | 56.84 | 0.5 | 2 |  |  |  |  |  |  |
| ATP-binding cassette sub-family A member 13 | ABCAD_HUMAN | 580524 | 6.01 |  |  |  |  |  |  |  |  |  | 66.45 | 0.5 | 3 |  |  |  |  |  |  |
| ATP-binding cassette sub-family B member 9 | ABCB9_HUMAN | 84877 | 8.00 |  |  |  |  |  |  |  |  |  | 74.74 | 4 | 3 |  |  |  |  |  |  |
| ATP-binding cassette sub-family C member 11* | ABCCB_HUMAN | 155855 | 8.40 | 61.15 | 1.3 | 2 |  |  |  |  |  |  |  |  |  |  |  |  |  |  |  |
| ATP-binding cassette sub-family C member 8 | ABCC8_HUMAN | 178476 | 7.99 | 50.66 | 0.9 | 2 |  |  |  |  |  |  | 52.14 | 0.9 | 2 |  |  |  |  |  |  |
| ATP-binding cassette sub-family F member 1 | ABCF1_HUMAN | 96323 | 6.40 |  |  |  | 73.72 | 3.1 | 3 |  |  |  | 59.09 | 2.1 | 2 |  |  |  |  |  |  |
| ATP-binding cassette sub-family F member 3 | ABCF3_HUMAN | 80094 | 5.96 |  |  |  |  |  |  |  |  |  | 57.52 | 2.1 | 2 |  |  |  |  |  |  |
| ATP-dendent (S)-NAD(P)H-hydrate dehydratase | CARKD_HUMAN | 36895 | 8.20 |  |  |  | 333.29 | 28.2 | 7 | 273.74 | 21.3 | 5 | 521.18 | 40.1 | 10 |  |  |  |  |  |  |
| Attractin | ATRN_HUMAN | 163450 | 7.25 | 462.45 | 8 | 10 |  |  |  |  |  |  | 208.94 | 3.5 | 5 |  |  |  |  |  |  |
| Azurocidin | CAP7_HUMAN | 27325 | 9.75 |  |  |  |  |  |  |  |  |  | 104.94 | 8 | 2 |  |  |  |  |  |  |
| Baculoviral IAP repeat-containing protein 6 | BIRC6_HUMAN | 536192 | 5.67 | 54.29 | 0.3 | 2 |  |  |  |  |  |  | 70.58 | 0.5 | 3 |  |  |  |  |  |  |
| BAI1-associated protein 3 | BAIP3_HUMAN | 133129 | 5.95 | 53.72 | 1.2 | 2 |  |  |  |  |  |  |  |  |  |  |  |  |  |  |  |
| Band 4.1-like protein 3 | E41L3_HUMAN | 121458 | 5.09 |  |  |  |  |  |  |  |  |  |  |  |  | 68.09 | 1.3 | 2 |  |  |  |
| Basement membrane-specific heparan sulfate proteoglycan core protein | PGBM_HUMAN | 479253 | 6.06 | 760.41 | 5 | 19 |  |  |  | 723.73 | 5.2 | 20 |  |  |  |  |  |  |  |  |  |
| Bax inhibitor 1* | BI1_HUMAN | 26805 | 8.79 |  |  |  |  |  |  |  |  |  | 50.09 | 3.8 | 2 |  |  |  |  |  |  |
| Beta-1.4-galactosyltransferase 1 | B4GT1_HUMAN | 44291 | 8.88 |  |  |  |  |  |  |  |  |  | 69.78 | 4.8 | 2 |  |  |  |  |  |  |
| Beta-2-glycoprotein 1 | APOH_HUMAN | 39584 | 8.35 | 685.82 | 47.5 | 11 | 317.26 | 23.8 | 7 | 434.32 | 39.4 | 9 | 467.81 | 31.9 | 10 | 172.4 | 11.9 | 4 | 221.45 | 24.6 | 6 |
| Beta-2-microglobulin | B2MG_HUMAN | 13820 | 6.06 | 170.12 | 40.3 | 4 | 214.25 | 40.3 | 4 | 166.8 | 40.3 | 4 | 145.58 | 40.3 | 4 |  |  |  | 103.34 | 35.3 | 3 |
| Beta-actin-like protein 2 | ACTBL_HUMAN | 42318 | 5.39 | 402.82 | 21 | 9 | 397.46 | 18.1 | 8 | 296.82 | 17.6 | 6 | 424.64 | 25.5 | 9 |  |  |  |  |  |  |
| Beta-Ala-His dipeptidase | CNDP1_HUMAN | 56784 | 5.14 |  |  |  |  |  |  |  |  |  | 778.62 | 43.6 | 16 | 134.2 | 7.3 | 4 |  |  |  |
| Beta-crystallin A2 | CRBA2_HUMAN | 22424 | 5.95 | 333.3 | 45.2 | 6 | 474.07 | 56.9 | 7 | 435.94 | 56.9 | 7 | 360.94 | 39.1 | 5 | 316.23 | 39.1 | 5 | 324.31 | 38.6 | 5 |
| Beta-crystallin A3 | CRBA1_HUMAN | 25419 | 5.81 | 1373.4 | 92.6 | 19 | 1388.26 | 85.6 | 19 | 1308.68 | 91.2 | 18 | 1294.73 | 91.2 | 18 | 1238.35 | 83.7 | 17 | 1272.97 | 87.4 | 19 |
| Beta-crystallin A4 | CRBA4_HUMAN | 22645 | 5.83 | 938.92 | 86.7 | 11 | 977.17 | 86.7 | 11 | 795.59 | 80.1 | 9 | 782.05 | 59.7 | 8 | 917.62 | 80.1 | 10 | 870.71 | 80.1 | 11 |
| Beta-crystallin B1 | CRBB1_HUMAN | 28063 | 8.60 | 1626.69 | 90.5 | 20 | 1926.03 | 93.7 | 25 | 1759.95 | 90.5 | 24 | 1868.15 | 89.7 | 24 | 1724.29 | 89.7 | 22 | 1550.99 | 87.7 | 20 |
| Beta-crystallin B2 | CRBB2_HUMAN | 23479 | 6.51 | 1455.67 | 93.7 | 22 | 1566.45 | 93.7 | 23 | 1587.14 | 94.1 | 25 | 1409.09 | 91.2 | 21 | 1503.58 | 85.4 | 22 | 1188.17 | 82.9 | 19 |
| Beta-crystallin B3 | CRBB3_HUMAN | 24351 | 6.25 | 728.65 | 61.6 | 12 | 704.23 | 72 | 12 | 752.26 | 67.3 | 11 | 853.44 | 75.8 | 13 | 557.98 | 61.1 | 10 | 768.58 | 72 | 12 |
| Beta-crystallin S | CRBS_HUMAN | 21392 | 6.44 | 939.08 | 85.4 | 14 | 1063.7 | 87.1 | 16 | 957.22 | 83.7 | 15 | 1019.49 | 85.4 | 16 | 872.99 | 82 | 13 | 966.65 | 84.3 | 15 |
| Beta-enolase | ENOB_HUMAN | 47244 | 7.59 |  |  |  |  |  |  |  |  |  | 528.19 | 23.7 | 9 |  |  |  |  |  |  |
| Beta-hexosaminidase subunit alpha | HEXA_HUMAN | 61120 | 5.04 |  |  |  |  |  |  |  |  |  | 514.34 | 18.5 | 9 | 76.96 | 9.6 | 3 |  |  |  |
| Beta-hexosaminidase subunit beta | HEXB_HUMAN | 63527 | 6.29 |  |  |  |  |  |  |  |  |  | 67.96 | 4.1 | 3 |  |  |  |  |  |  |
| Biotinidase | BTD_HUMAN | 62006 | 5.82 | 231.21 | 12.7 | 5 | 166.91 | 8.7 | 4 | 262.18 | 9.8 | 4 | 617.33 | 25.8 | 10 | 571.41 | 24.5 | 9 | 90.21 | 4.1 | 2 |
| Bisphosphoglycerate mutase | PMGE_HUMAN | 30158 | 6.10 | 734.62 | 54.8 | 13 | 482.38 | 35.5 | 8 | 656.53 | 54.4 | 11 | 716.49 | 64.1 | 13 | 411.25 | 36.7 | 8 | 364.11 | 40.9 | 8 |
| Bleomycin hydrolase | BLMH_HUMAN | 53155 | 5.88 | 64.68 | 4 | 2 | 59.79 | 4 | 2 |  |  |  | 124.6 | 6.6 | 3 |  |  |  |  |  |  |
| BPI fold-containing family A member 1 | BPIA1_HUMAN | 26810 | 5.41 |  |  |  |  |  |  |  |  |  | 492.05 | 34.4 | 6 |  |  |  |  |  |  |
| BPI fold-containing family B member 1 | BPIB1_HUMAN | 52580 | 6.72 |  |  |  | 209.31 | 13.2 | 5 |  |  |  | 1032.59 | 48.8 | 19 |  |  |  |  |  |  |
| BPI fold-containing family B member 2 | BPIB2_HUMAN | 49256 | 8.82 |  |  |  |  |  |  |  |  |  | 246.41 | 19.2 | 6 |  |  |  |  |  |  |
| Brain acid soluble protein 1 | BASP1_HUMAN | 22680 | 4.65 | 573.76 | 68.7 | 9 |  |  |  |  |  |  | 478.62 | 56.4 | 8 | 443.27 | 69.2 | 8 | 285.09 | 41 | 5 |
| Brain-specific angiogenesis inhibitor 1 | BAI1_HUMAN | 176900 | 8.28 |  |  |  |  |  |  |  |  |  | 63.52 | 1.9 | 3 |  |  |  |  |  |  |
| Brefeldin A-inhibited guanine nucleotide-exchange protein 2 | BIG2_HUMAN | 204418 | 5.93 | 56.58 | 0.8 | 2 | 76.7 | 1.3 | 3 |  |  |  | 60.22 | 1 | 2 |  |  |  |  |  |  |
| Brevican core protein | PGCB_HUMAN | 100539 | 4.58 |  |  |  |  |  |  |  |  |  | 80.32 | 2.3 | 3 | 84.14 | 2.3 | 3 |  |  |  |
| Bromodomain adjacent to zinc finger domain protein 2B | BAZ2B_HUMAN | 242078 | 6.13 | 77.58 | 1 | 3 |  |  |  |  |  |  |  |  |  |  |  |  |  |  |  |
| Bromodomain-containing protein 4* | BRD4_HUMAN | 152580 | 9.23 |  |  |  |  |  |  |  |  |  | 64.02 | 1.4 | 2 |  |  |  |  |  |  |
| C3 and PZP-like alpha-2-macroglobulin domain-containing protein 8 | CPMD8_HUMAN | 208624 | 6.00 | 207.6 | 3.4 | 5 |  |  |  |  |  |  | 267.46 | 3.7 | 5 |  |  |  |  |  |  |
| Cadherin-2 | CADH2_HUMAN | 100203 | 4.65 |  |  |  |  |  |  | 354.36 | 8.8 | 8 | 205.54 | 8.3 | 6 | 142.42 | 7 | 3 |  |  |  |
| Cadherin-6 | CADH6_HUMAN | 88539 | 4.77 |  |  |  |  |  |  |  |  |  | 145.56 | 5.8 | 4 |  |  |  |  |  |  |
| Cadherin-related family member 1 | CDHR1_HUMAN | 93765 | 5.34 |  |  |  |  |  |  |  |  |  | 111.04 | 4.1 | 3 | 136.63 | 5.2 | 3 |  |  |  |
| Calcium-dependent secretion activator 2 | CAPS2_HUMAN | 148895 | 5.84 |  |  |  |  |  |  |  |  |  | 75.04 | 1.1 | 2 |  |  |  |  |  |  |
| Calcium-regulated heat stable protein 1 | CHSP1_HUMAN | 16110 | 8.42 |  |  |  |  |  |  | 118.06 | 18.4 | 2 |  |  |  | 89.76 | 12.9 | 2 | 100.87 | 18.4 | 2 |
| Calcyphosin-2* | CAYP2_HUMAN | 64480 | 8.87 |  |  |  |  |  |  |  |  |  | 49.5 | 2.2 | 2 |  |  |  |  |  |  |
| Caldesmon | CALD1_HUMAN | 93232 | 5.62 | 118.62 | 4.8 | 5 |  |  |  |  |  |  |  |  |  |  |  |  |  |  |  |
| Calmodulin-like protein 5 | CALL5_HUMAN | 15883 | 4.35 |  |  |  | 214.27 | 49.3 | 5 | 72.99 | 21.2 | 2 |  |  |  |  |  |  |  |  |  |
| Calreticulin | CALR_HUMAN | 48283 | 4.29 |  |  |  |  |  |  |  |  |  |  |  |  | 126.65 | 6.2 | 2 |  |  |  |
| Calsyntenin-1 | CSTN1_HUMAN | 110978 | 4.82 | 862.13 | 18.8 | 19 | 1571.51 | 33.4 | 28 | 575.03 | 14.9 | 12 | 1265.91 | 29.6 | 25 | 233.24 | 8.8 | 8 | 173.49 | 5 | 5 |
| Calsyntenin-2* | CSTN2_HUMAN | 108249 | 5.21 |  |  |  |  |  |  |  |  |  | 51.31 | 1.7 | 2 |  |  |  |  |  |  |
| cAMP-dependent protein kinase type I-alpha regulatory subunit | KAP0_HUMAN | 43183 | 5.27 |  |  |  |  |  |  |  |  |  | 233.89 | 15.2 | 5 |  |  |  |  |  |  |
| CAP-Gly domain-containing linker protein 1 | CLIP1_HUMAN | 162888 | 5.29 |  |  |  |  |  |  |  |  |  | 68.77 | 1 | 3 |  |  |  |  |  |  |
| CAP-Gly domain-containing linker protein 2 | CLIP2_HUMAN | 116223 | 6.29 |  |  |  |  |  |  |  |  |  | 114.82 | 4.1 | 5 |  |  |  |  |  |  |
| Carbonic anhydrase 1 | CAH1_HUMAN | 28909 | 6.59 | 78.76 | 10.7 | 2 |  |  |  |  |  |  |  |  |  |  |  |  |  |  |  |
| Carbonic anhydrase 14* | CAH14_HUMAN | 37872 | 5.90 |  |  |  |  |  |  |  |  |  | 58.88 | 5 | 2 |  |  |  |  |  |  |
| Carbonic anhydrase 2 | CAH2_HUMAN | 29285 | 6.87 | 744.11 | 59.6 | 14 | 511.89 | 44.6 | 9 | 403.85 | 35.8 | 7 | 554.05 | 45.8 | 10 | 234.59 | 18.5 | 4 | 186.01 | 23.8 | 5 |
| Carbonic anhydrase-related protein 10 | CAH10_HUMAN | 37824 | 8.22 |  |  |  |  |  |  |  |  |  | 112.45 | 6.4 | 2 |  |  |  |  |  |  |
| Carbonyl reductase [NADPH] 1 | CBR1_HUMAN | 30641 | 8.56 | 549.17 | 46.2 | 10 | 1250 | 75.1 | 18 | 1207.39 | 77.3 | 18 | 1287.8 | 78.7 | 19 | 838.3 | 54.2 | 11 | 871.78 | 67.9 | 14 |
| Carboxypeptidase B2 | CBPB2_HUMAN | 48964 | 7.62 |  |  |  |  |  |  |  |  |  | 58.08 | 3.3 | 2 |  |  |  |  |  |  |
| Carboxypeptidase E | CBPE_HUMAN | 53516 | 5.03 |  |  |  | 62.81 | 4.2 | 2 | 201.88 | 11.3 | 4 | 529.65 | 30.7 | 10 | 192.28 | 10.9 | 4 |  |  |  |
| Carboxypeptidase N subunit 2 | CPN2_HUMAN | 61373 | 5.64 |  |  |  |  |  |  |  |  |  | 110.62 | 7 | 4 |  |  |  |  |  |  |
| Cardiomyopathy-associated protein 5 | CMYA5_HUMAN | 450760 | 4.73 |  |  |  | 56.38 | 0.5 | 2 |  |  |  | 71.41 | 0.7 | 3 |  |  |  |  |  |  |
| Cartilage acidic protein 1 | CRAC1_HUMAN | 72174 | 4.95 |  |  |  | 118.41 | 3.8 | 3 |  |  |  | 319.43 | 14.7 | 9 |  |  |  |  |  |  |
| Caspase-14 | CASPE_HUMAN | 27947 | 5.45 | 88.42 | 9.1 | 2 | 204.83 | 16.9 | 4 | 248.77 | 22.3 | 5 | 643.69 | 40.5 | 12 |  |  |  |  |  |  |
| Caspase-8* | CASP8_HUMAN | 56097 | 5.00 |  |  |  |  |  |  |  |  |  | 83.83 | 3.3 | 2 |  |  |  |  |  |  |
| Catalase | CATA_HUMAN | 59947 | 6.90 |  |  |  |  |  |  | 103.64 | 5.5 | 3 | 196.85 | 9.3 | 5 |  |  |  |  |  |  |
| Catenin alpha-1* | CTNA1_HUMAN | 100693 | 5.95 |  |  |  | 61.84 | 1.9 | 2 |  |  |  |  |  |  |  |  |  |  |  |  |
| Catenin alpha-2 | CTNA2_HUMAN | 106045 | 5.52 |  |  |  | 60.53 | 2.1 | 2 |  |  |  | 78.24 | 2.7 | 3 |  |  |  |  |  |  |
| Catenin beta-1 | CTNB1_HUMAN | 86069 | 5.53 | 106.04 | 3.5 | 3 | 126.6 | 4.5 | 4 | 135.75 | 4.9 | 4 | 163.47 | 5.5 | 5 |  |  |  |  |  |  |
| Cathepsin D | CATD_HUMAN | 45037 | 6.11 | 580.58 | 32.3 | 10 | 818.21 | 36.2 | 13 | 641.39 | 32.3 | 10 | 899.73 | 40.8 | 15 | 326.62 | 23.3 | 7 | 625.98 | 35.4 | 10 |
| Cathepsin L1 | CATL1_HUMAN | 37996 | 5.31 |  |  |  |  |  |  |  |  |  | 195.78 | 15 | 4 |  |  |  |  |  |  |
| Cathepsin Z | CATZ_HUMAN | 34530 | 6.70 |  |  |  |  |  |  |  |  |  | 175.96 | 9.6 | 3 |  |  |  |  |  |  |
| Caveolin-1 | CAV1_HUMAN | 20630 | 5.66 |  |  |  | 148.76 | 30.3 | 4 | 188.82 | 33.1 | 4 | 214.45 | 36 | 5 | 71.55 | 22.5 | 2 | 80.42 | 16.3 | 2 |
| Cell adhesion molecule 1 | CADM1_HUMAN | 48935 | 4.94 |  |  |  |  |  |  |  |  |  |  |  |  | 106.21 | 11.5 | 2 |  |  |  |
| Cell adhesion molecule 2* | CADM2_HUMAN | 47980 | 5.17 |  |  |  |  |  |  |  |  |  |  |  |  | 60.73 | 10.1 | 2 |  |  |  |
| Cell adhesion molecule-related/down-regulated by oncogenes | CDON_HUMAN | 140656 | 6.04 |  |  |  |  |  |  |  |  |  | 61.03 | 1.5 | 2 |  |  |  |  |  |  |
| Cell division cycle 5-like protein | CDC5L_HUMAN | 92422 | 8.23 |  |  |  |  |  |  |  |  |  | 64.98 | 3.6 | 3 |  |  |  |  |  |  |
| Centlein | CNTLN_HUMAN | 162131 | 8.28 | 51.28 | 1.1 | 2 | 55.45 | 0.9 | 2 | 70.09 | 1.9 | 3 | 68.14 | 1.8 | 3 |  |  |  |  |  |  |
| Centriolin | CNTRL_HUMAN | 269860 | 5.45 |  |  |  |  |  |  |  |  |  | 156.33 | 1.4 | 6 |  |  |  |  |  |  |
| Centromere protein F | CENPF_HUMAN | 370844 | 5.03 | 59.38 | 0.4 | 2 |  |  |  |  |  |  | 107.26 | 1 | 4 |  |  |  |  |  |  |
| Centromere-associated protein E | CENPE_HUMAN | 317588 | 5.51 |  |  |  |  |  |  |  |  |  | 115.2 | 1.5 | 5 |  |  |  |  |  |  |
| Centrosomal protein of 164 kDa | CE164_HUMAN | 164727 | 5.27 |  |  |  |  |  |  |  |  |  | 69.96 | 1.5 | 3 |  |  |  |  |  |  |
| Centrosomal protein of 290 kDa | CE290_HUMAN | 290892 | 5.75 | 48.8 | 0.5 | 2 | 99.01 | 1.5 | 4 | 84.72 | 0.8 | 3 | 181.75 | 2.1 | 8 |  |  |  |  |  |  |
| Centrosome and spindle pole-associated protein 1* | CSPP1_HUMAN | 145718 | 6.37 |  |  |  |  |  |  |  |  |  | 55.45 | 1.3 | 2 |  |  |  |  |  |  |
| Centrosome-associated protein 350 | CE350_HUMAN | 352312 | 5.95 |  |  |  |  |  |  |  |  |  | 82.31 | 1.1 | 4 |  |  |  |  |  |  |
| Ceramide kinase* | CERK1_HUMAN | 61194 | 8.55 |  |  |  |  |  |  |  |  |  | 51.66 | 2.6 | 2 |  |  |  |  |  |  |
| Cerebellin-1 | CBLN1_HUMAN | 21198 | 6.73 |  |  |  |  |  |  |  |  |  | 84.67 | 14.5 | 3 |  |  |  |  |  |  |
| Ceruloplasmin | CERU_HUMAN | 122983 | 5.44 | 2628.94 | 51.2 | 44 | 2875.84 | 58.5 | 52 | 2176.33 | 42.7 | 39 | 2825.76 | 46.9 | 46 | 1054.48 | 27.1 | 20 | 1270.46 | 37.7 | 26 |
| Chitinase-3-like protein 1 | CH3L1_HUMAN | 42998 | 8.69 | 160 | 11.5 | 4 | 69.91 | 5 | 2 | 271.01 | 15.1 | 5 | 546.67 | 33.9 | 10 |  |  |  |  |  |  |
| Chloride intracellular channel protein 1 | CLIC1_HUMAN | 27248 | 5.09 |  |  |  |  |  |  |  |  |  | 262.05 | 27 | 5 |  |  |  |  |  |  |
| Chloride intracellular channel protein 5 | CLIC5_HUMAN | 46816 | 4.71 |  |  |  |  |  |  |  |  |  | 400.65 | 23.7 | 8 |  |  |  |  |  |  |
| Chondroitin sulfate N-acetylgalactosaminyltransferase 1 | CGAT1_HUMAN | 61769 | 8.63 |  |  |  |  |  |  |  |  |  | 67.48 | 3.9 | 3 |  |  |  |  |  |  |
| Chondroitin sulfate synthase 1 | CHSS1_HUMAN | 92353 | 9.31 |  |  |  |  |  |  |  |  |  | 99.8 | 4.2 | 4 |  |  |  |  |  |  |
| Chromogranin-A | CMGA_HUMAN | 50829 | 4.58 |  |  |  |  |  |  |  |  |  |  |  |  | 154.73 | 9.4 | 3 |  |  |  |
| Cingulin | CING_HUMAN | 136532 | 5.47 |  |  |  |  |  |  |  |  |  | 101.19 | 2.3 | 4 |  |  |  |  |  |  |
| Cingulin-like protein 1 | CGNL1_HUMAN | 149559 | 5.51 |  |  |  |  |  |  |  |  |  | 61.87 | 1.8 | 3 |  |  |  |  |  |  |
| C-Jun-amino-terminal kinase-interacting protein 4 | JIP4_HUMAN | 146913 | 5.05 |  |  |  |  |  |  |  |  |  | 100.14 | 2.6 | 4 |  |  |  |  |  |  |
| Clathrin heavy chain 1 | CLH1_HUMAN | 193260 | 5.48 | 1432.93 | 23.7 | 30 | 477.83 | 6.7 | 11 | 512.59 | 7 | 10 | 150 | 2.6 | 5 |  |  |  |  |  |  |
| Clusterin | CLUS_HUMAN | 53031 | 5.89 | 589.85 | 29.4 | 11 | 902.18 | 33.6 | 16 | 720.91 | 27.8 | 13 | 1397.11 | 42.5 | 23 | 1516.88 | 41.4 | 23 | 872.32 | 34.3 | 15 |
| Clusterin-associated protein 1* | CLUA1_HUMAN | 48209 | 4.69 |  |  |  |  |  |  |  |  |  | 60.6 | 3.6 | 2 |  |  |  |  |  |  |
| Coactosin-like protein | COTL1_HUMAN | 16049 | 5.54 | 348.5 | 63.4 | 9 | 474.12 | 71.8 | 10 | 505.23 | 80.3 | 11 | 545.98 | 71.8 | 12 | 246.27 | 32.4 | 5 | 219.84 | 50 | 6 |
| Coagulation factor V | FA5_HUMAN | 252686 | 5.69 | 122.76 | 2.1 | 4 |  |  |  |  |  |  |  |  |  |  |  |  |  |  |  |
| Coagulation factor XII | FA12_HUMAN | 70029 | 8.04 | 194.93 | 12.2 | 7 | 164.65 | 8.5 | 4 | 84.07 | 2.4 | 2 | 140.19 | 8.5 | 4 |  |  |  |  |  |  |
| Cofilin-1 | COF1_HUMAN | 18719 | 8.22 | 103.07 | 23.5 | 2 |  |  |  |  |  |  |  |  |  |  |  |  |  |  |  |
| Cofilin-2* | COF2_HUMAN | 18839 | 7.66 |  |  |  |  |  |  |  |  |  | 65.1 | 11.4 | 2 |  |  |  |  |  |  |
| Coiled-coil and C2 domain-containing protein 2A* | C2D2A_HUMAN | 186754 | 6.30 |  |  |  |  |  |  |  |  |  | 54.85 | 1.3 | 2 |  |  |  |  |  |  |
| Coiled-coil domain-containing protein 110 | CC110_HUMAN | 97235 | 5.89 |  |  |  |  |  |  |  |  |  | 70.45 | 2.6 | 3 |  |  |  |  |  |  |
| Coiled-coil domain-containing protein 121* | CC121_HUMAN | 33155 | 9.84 |  |  |  |  |  |  |  |  |  | 52.27 | 6.1 | 2 |  |  |  |  |  |  |
| Coiled-coil domain-containing protein 138 | CC138_HUMAN | 76912 | 8.75 |  |  |  |  |  |  |  |  |  | 74.12 | 3 | 3 |  |  |  |  |  |  |
| Coiled-coil domain-containing protein 144A | C144A_HUMAN | 166448 | 5.27 |  |  |  |  |  |  |  |  |  | 68.05 | 1.6 | 3 |  |  |  |  |  |  |
| Coiled-coil domain-containing protein 147 | CC147_HUMAN | 103525 | 8.45 |  |  |  |  |  |  |  |  |  | 68.49 | 2.6 | 3 |  |  |  |  |  |  |
| Coiled-coil domain-containing protein 148* | CC148_HUMAN | 71601 | 9.00 |  |  |  |  |  |  |  |  |  | 59.85 | 1.4 | 2 |  |  |  |  |  |  |
| Coiled-coil domain-containing protein 30 | CCD30_HUMAN | 91905 | 5.57 |  |  |  |  |  |  |  |  |  | 68.05 | 3.3 | 3 |  |  |  |  |  |  |
| Coiled-coil domain-containing protein 87 | CCD87_HUMAN | 96741 | 8.74 |  |  |  |  |  |  |  |  |  | 74.72 | 2.5 | 3 |  |  |  |  |  |  |
| Coiled-coil domain-containing protein 88B | CC88B_HUMAN | 165166 | 5.10 |  |  |  |  |  |  |  |  |  | 65.02 | 1.6 | 3 |  |  |  |  |  |  |
| Collagen alpha-1(II) chain | CO2A1_HUMAN | 142782 | 6.58 | 152.35 | 2.1 | 3 | 370.47 | 4.4 | 6 |  |  |  | 620.82 | 7.7 | 11 | 358.25 | 4.1 | 6 |  |  |  |
| Collagen alpha-1(IX) chain | CO9A1_HUMAN | 92440 | 8.94 |  |  |  |  |  |  |  |  |  |  |  |  | 105.49 | 2.4 | 2 |  |  |  |
| Collagen alpha-1(VI) chain | CO6A1_HUMAN | 109602 | 5.26 | 126.48 | 2.7 | 2 |  |  |  | 271.39 | 7.8 | 6 | 194.17 | 6.1 | 5 |  |  |  |  |  |  |
| Collagen alpha-1(XII) chain* | COCA1_HUMAN | 334138 | 5.38 |  |  |  |  |  |  | 52.78 | 0.5 | 2 |  |  |  |  |  |  |  |  |  |
| Collagen alpha-1(XVIII) chain | COIA1_HUMAN | 179389 | 5.67 | 230.31 | 4 | 4 | 171.57 | 2.7 | 4 | 200.74 | 2.5 | 4 |  |  |  |  |  |  |  |  |  |
| Collagen alpha-2(IX) chain | CO9A2_HUMAN | 65319 | 9.24 | 150.18 | 3.9 | 2 | 178.1 | 3.9 | 2 |  |  |  | 142.01 | 3.9 | 2 | 200.53 | 3.9 | 2 |  |  |  |
| COMM domain-containing protein 4* | COMD4_HUMAN | 22092 | 6.89 |  |  |  |  |  |  |  |  |  | 48.91 | 7 | 2 |  |  |  |  |  |  |
| Complement C1q subcomponent subunit B | C1QB_HUMAN | 26933 | 8.83 |  |  |  |  |  |  |  |  |  | 247.87 | 22.9 | 6 |  |  |  |  |  |  |
| Complement C1q subcomponent subunit C | C1QC_HUMAN | 25985 | 8.61 |  |  |  | 82.39 | 8.6 | 2 |  |  |  | 182.66 | 21.2 | 4 | 165.83 | 17.6 | 3 |  |  |  |
| Complement C1q tumor necrosis factor-related protein 3 | C1QT3_HUMAN | 27262 | 6.05 |  |  |  | 118.93 | 9.8 | 2 |  |  |  | 157.87 | 15 | 3 |  |  |  |  |  |  |
| Complement C1r subcomponent | C1R_HUMAN | 81606 | 5.82 | 116.57 | 4 | 3 | 85.09 | 2.4 | 2 |  |  |  | 332.36 | 13.6 | 7 |  |  |  |  |  |  |
| Complement C1r subcomponent-like protein* | C1RL_HUMAN | 54206 | 6.75 |  |  |  |  |  |  |  |  |  | 72.24 | 4.7 | 2 |  |  |  |  |  |  |
| Complement C1s subcomponent | C1S_HUMAN | 78174 | 4.86 |  |  |  | 104.46 | 4.7 | 4 | 222.01 | 7 | 5 | 460.89 | 19.3 | 11 |  |  |  |  |  |  |
| Complement C2 | CO2_HUMAN | 84583 | 7.24 | 814.28 | 27.9 | 18 | 304.68 | 12.1 | 9 |  |  |  | 984.28 | 28.6 | 22 |  |  |  |  |  |  |
| Complement C3 | CO3_HUMAN | 188569 | 6.03 | 5503.95 | 68.1 | 94 | 5274.17 | 65.8 | 102 | 3320.67 | 46.4 | 64 | 4155.55 | 55 | 78 | 1714.44 | 31.7 | 34 | 2138.15 | 39.1 | 49 |
| Complement C4-A | CO4A_HUMAN | 194247 | 6.66 | 2786.85 | 35.2 | 49 | 3378.44 | 47.1 | 63 |  |  |  | 3657.77 | 48.2 | 66 | 1091.08 | 18 | 22 | 1479.34 | 25.7 | 29 |
| Complement C4-B | CO4B_HUMAN | 194212 | 6.74 | 2803.3 | 35.2 | 49 |  |  |  | 2837.95 | 40.4 | 52 | 3710.43 | 48.2 | 66 |  |  |  |  |  |  |
| Complement C5 | CO5_HUMAN | 189897 | 6.11 | 1292.39 | 21.6 | 30 | 125.71 | 2.3 | 4 |  |  |  | 172.85 | 2.8 | 4 |  |  |  |  |  |  |
| Complement component C6 | CO6_HUMAN | 108367 | 6.40 | 643.42 | 18.3 | 14 |  |  |  |  |  |  | 600.77 | 16.3 | 15 |  |  |  |  |  |  |
| Complement component C7 | CO7_HUMAN | 96650 | 6.09 | 863.2 | 24.6 | 15 | 641.28 | 21.6 | 13 |  |  |  | 632.09 | 21.1 | 15 |  |  |  | 63.38 | 2.6 | 2 |
| Complement component C8 gamma chain | CO8G_HUMAN | 22435 | 8.49 |  |  |  |  |  |  |  |  |  | 197.02 | 24.3 | 4 |  |  |  |  |  |  |
| Complement component C9 | CO9_HUMAN | 64615 | 5.43 | 499.54 | 26.5 | 10 | 164.94 | 8.6 | 4 | 102.88 | 4.5 | 2 | 512.38 | 18.8 | 10 |  |  |  |  |  |  |
| Complement factor B | CFAB_HUMAN | 86847 | 6.68 | 1601.32 | 39.3 | 31 | 1619.23 | 45.3 | 28 | 568.43 | 19.4 | 12 | 1635.95 | 39.5 | 31 | 85.35 | 3.8 | 3 | 480.36 | 16.4 | 10 |
| Complement factor D* | CFAD_HUMAN | 27529 | 7.65 | 53.41 | 9.1 | 2 |  |  |  |  |  |  |  |  |  |  |  |  |  |  |  |
| Complement factor H | CFAH_HUMAN | 143680 | 6.21 | 1479.45 | 31 | 32 | 528.56 | 9.5 | 11 | 221.65 | 4.4 | 5 | 602.82 | 11.2 | 14 |  |  |  |  |  |  |
| Complement factor I | CFAI_HUMAN | 68102 | 7.72 | 519.17 | 25.9 | 12 | 361 | 13.6 | 7 | 369.83 | 12.5 | 6 | 501.77 | 18.9 | 9 | 97.55 | 5.5 | 3 |  |  |  |
| Condensin-2 complex subunit D3 | CNDD3_HUMAN | 170950 | 7.39 |  |  |  |  |  |  |  |  |  | 89.29 | 1.5 | 3 |  |  |  |  |  |  |
| Conserved oligomeric Golgi complex subunit 2 | COG2_HUMAN | 83726 | 6.20 | 52.51 | 2.3 | 2 |  |  |  |  |  |  |  |  |  |  |  |  |  |  |  |
| Constitutive coactivator of PPAR-gamma-like protein 2* | F120C_HUMAN | 121652 | 9.19 |  |  |  | 49.8 | 1.6 | 2 |  |  |  |  |  |  |  |  |  |  |  |  |
| Contactin-1 | CNTN1_HUMAN | 114104 | 5.63 | 178.32 | 3.4 | 3 |  |  |  | 237.84 | 7.4 | 5 | 775.28 | 20.4 | 16 |  |  |  |  |  |  |
| Contactin-2 | CNTN2_HUMAN | 114007 | 8.11 |  |  |  |  |  |  |  |  |  | 270.6 | 6.7 | 5 |  |  |  |  |  |  |
| COP9 signalosome complex subunit 5 | CSN5_HUMAN | 37783 | 6.10 |  |  |  |  |  |  |  |  |  | 115.67 | 7.8 | 3 |  |  |  |  |  |  |
| COP9 signalosome complex subunit 6 | CSN6_HUMAN | 36482 | 5.48 |  |  |  |  |  |  |  |  |  | 144.28 | 11.3 | 4 |  |  |  |  |  |  |
| Copine-3 | CPNE3_HUMAN | 60947 | 5.61 |  |  |  |  |  |  |  |  |  | 109.15 | 3.2 | 2 |  |  |  |  |  |  |
| Corneodesmosin* | CDSN_HUMAN | 52288 | 8.69 |  |  |  |  |  |  |  |  |  | 92.23 | 5.1 | 2 |  |  |  |  |  |  |
| Cornifin-A | SPR1A_HUMAN | 10327 | 8.85 | 58.65 | 18 | 2 |  |  |  |  |  |  | 103.8 | 33.7 | 3 |  |  |  |  |  |  |
| Corticosteroid-binding globulin | CBG_HUMAN | 45283 | 5.65 | 170.2 | 12.8 | 5 | 242.54 | 14.8 | 5 | 302.73 | 16.8 | 6 | 592.93 | 31.6 | 10 | 541.65 | 37.3 | 10 | 199.04 | 10.9 | 4 |
| Cullin-1* | CUL1_HUMAN | 90306 | 8.19 |  |  |  | 52.47 | 1.9 | 2 | 51.22 | 1.9 | 2 |  |  |  |  |  |  |  |  |  |
| Cullin-5 | CUL5_HUMAN | 91468 | 8.09 | 94.25 | 3.2 | 2 |  |  |  |  |  |  |  |  |  |  |  |  |  |  |  |
| Cullin-associated NEDD8-dissociated protein 1 | CAND1_HUMAN | 137999 | 5.53 | 472.97 | 12.9 | 12 | 198.91 | 3.7 | 3 | 191.44 | 3.7 | 3 |  |  |  |  |  |  |  |  |  |
| Cystatin-A | CYTA_HUMAN | 11000 | 5.39 |  |  |  |  |  |  | 202.36 | 37.8 | 3 | 278.19 | 68.4 | 5 |  |  |  |  |  |  |
| Cystatin-B | CYTB_HUMAN | 11190 | 6.96 |  |  |  | 89.7 | 18.4 | 2 |  |  |  |  |  |  |  |  |  |  |  |  |
| Cystatin-C | CYTC_HUMAN | 16017 | 9.00 | 415.01 | 50.7 | 7 | 603.59 | 63 | 9 | 347.59 | 41.8 | 5 | 230.49 | 19.2 | 4 | 255.83 | 29.5 | 4 | 301.93 | 62.3 | 7 |
| Cysteine-rich secretory protein 3 | CRIS3_HUMAN | 28524 | 8.09 |  |  |  |  |  |  |  |  |  | 161.8 | 13.5 | 3 |  |  |  |  |  |  |
| Cystic fibrosis transmembrane conductance regulator* | CFTR_HUMAN | 169061 | 8.91 | 49.42 | 1 | 2 |  |  |  |  |  |  |  |  |  |  |  |  |  |  |  |
| Cytidine deaminase | CDD_HUMAN | 16687 | 6.55 | 133.44 | 18.5 | 2 | 327.66 | 46.6 | 4 | 135.07 | 28.1 | 2 |  |  |  | 366.65 | 47.3 | 6 | 193.08 | 32.9 | 3 |
| Cytoplasmic aconitate hydratase | ACOC_HUMAN | 98850 | 6.23 |  |  |  |  |  |  |  |  |  | 510.02 | 17.4 | 14 |  |  |  |  |  |  |
| Cytoplasmic dynein 1 heavy chain 1 | DYHC1_HUMAN | 534809 | 6.01 | 1630.62 | 9.6 | 45 | 88.56 | 0.8 | 4 | 286.61 | 2.5 | 11 |  |  |  |  |  |  |  |  |  |
| Cytosolic non-specific dipeptidase | CNDP2_HUMAN | 53187 | 5.66 | 330.89 | 28.2 | 9 | 150.02 | 9.7 | 4 | 414.32 | 25.7 | 8 | 581.36 | 41.9 | 14 |  |  |  |  |  |  |
| Dapper homolog 2* | DACT2_HUMAN | 83276 | 9.10 |  |  |  |  |  |  |  |  |  | 51.47 | 1.8 | 2 |  |  |  |  |  |  |
| D-dopachrome decarboxylase | DOPD_HUMAN | 12818 | 6.72 | 85.48 | 22 | 2 | 229.04 | 49.2 | 5 | 210.29 | 37.3 | 4 |  |  |  |  |  |  |  |  |  |
| Deleted in malignant brain tumors 1 protein | DMBT1_HUMAN | 268039 | 5.18 |  |  |  |  |  |  |  |  |  | 219.18 | 2.1 | 4 |  |  |  |  |  |  |
| Delta-aminolevulinic acid dehydratase | HEM2_HUMAN | 36728 | 6.32 |  |  |  | 66.99 | 5.8 | 2 |  |  |  | 134.59 | 16.1 | 4 |  |  |  |  |  |  |
| DEP domain-containing protein 7* | DEPD7_HUMAN | 58615 | 7.62 |  |  |  |  |  |  |  |  |  | 53.15 | 4.7 | 2 |  |  |  |  |  |  |
| Dermcidin | DCD_HUMAN | 11391 | 6.09 | 236.4 | 22.7 | 3 | 263.57 | 35.5 | 4 | 363.41 | 53.6 | 6 | 318.28 | 46.4 | 5 | 132.34 | 35.5 | 3 |  |  |  |
| Dermokine | DMKN_HUMAN | 47282 | 6.80 |  |  |  |  |  |  | 53.53 | 3.6 | 2 | 127.63 | 7.4 | 3 |  |  |  |  |  |  |
| Desmocollin-1 | DSC1_HUMAN | 101406 | 5.26 |  |  |  |  |  |  | 203.74 | 6.9 | 5 | 379.45 | 11.5 | 9 |  |  |  |  |  |  |
| Desmocollin-2 | DSC2_HUMAN | 101324 | 5.19 |  |  |  |  |  |  |  |  |  | 97.6 | 3.4 | 3 |  |  |  |  |  |  |
| Desmoglein-1 | DSG1_HUMAN | 114702 | 4.91 | 157.43 | 4.6 | 4 | 468.53 | 13.1 | 10 | 900.46 | 16.6 | 15 | 1143.6 | 23.5 | 20 | 310.36 | 10.2 | 6 |  |  |  |
| Desmoplakin | DESP_HUMAN | 334021 | 6.44 | 473.59 | 4.4 | 14 | 1474.08 | 13.3 | 34 | 1344.19 | 11.2 | 32 | 2804.8 | 23.4 | 66 | 727.79 | 6.4 | 15 |  |  |  |
| Diacylglycerol kinase delta | DGKD_HUMAN | 136607 | 7.62 | 64.64 | 2.1 | 3 |  |  |  |  |  |  |  |  |  |  |  |  |  |  |  |
| Dickkopf-related protein 3 | DKK3_HUMAN | 39563 | 4.60 | 425.48 | 37.7 | 8 | 521.49 | 32 | 8 | 529.2 | 32 | 9 | 578.06 | 33.4 | 8 | 815.17 | 53.1 | 11 | 410.64 | 28.6 | 7 |
| Dihydropteridine reductase | DHPR_HUMAN | 26001 | 6.91 | 114.41 | 19.7 | 3 | 196.2 | 15.6 | 4 |  |  |  | 215.53 | 21.7 | 5 |  |  |  |  |  |  |
| Dipeptidyl peptidase 2 | DPP2_HUMAN | 54763 | 5.91 |  |  |  |  |  |  |  |  |  | 317.12 | 15.9 | 7 |  |  |  |  |  |  |
| Dipeptidyl peptidase 3 | DPP3_HUMAN | 82880 | 5.03 |  |  |  |  |  |  |  |  |  | 122.21 | 7.5 | 4 |  |  |  |  |  |  |
| DIS3-like exonuclease 2 | DI3L2_HUMAN | 100243 | 5.74 |  |  |  |  |  |  |  |  |  | 72.08 | 3.1 | 3 |  |  |  |  |  |  |
| DNA damage-binding protein 1 | DDB1_HUMAN | 128142 | 5.14 | 112.26 | 3.5 | 3 |  |  |  |  |  |  | 234.55 | 5.4 | 7 |  |  |  |  |  |  |
| DNA excision repair protein ERCC-6 | ERCC6_HUMAN | 169452 | 8.28 |  |  |  |  |  |  |  |  |  | 87.7 | 2.3 | 4 |  |  |  |  |  |  |
| DNA repair protein RAD50* | RAD50_HUMAN | 154823 | 6.49 | 51.74 | 1 | 2 |  |  |  | 54.17 | 1 | 2 | 78.1 | 3.5 | 3 |  |  |  |  |  |  |
| DNA replication licensing factor MCM6* | MCM6_HUMAN | 93801 | 5.30 |  |  |  |  |  |  |  |  |  | 55.16 | 1.6 | 2 |  |  |  |  |  |  |
| DNA topoisomerase 2-beta | TOP2B_HUMAN | 184122 | 8.14 | 48.87 | 0.9 | 2 | 83.44 | 1.4 | 3 | 56.54 | 0.9 | 2 | 78.1 | 1.6 | 3 |  |  |  |  |  |  |
| DNA-directed RNA polymerase III subunit RPC2* | RPC2_HUMAN | 129242 | 8.77 | 51.79 | 1.5 | 2 |  |  |  |  |  |  |  |  |  |  |  |  |  |  |  |
| Doublesex- and mab-3-related transcription factor A1* | DMRTA_HUMAN | 53890 | 9.15 |  |  |  |  |  |  |  |  |  | 51.44 | 3.8 | 2 |  |  |  |  |  |  |
| Double-strand break repair protein MRE11A* | MRE11_HUMAN | 80885 | 5.61 |  |  |  |  |  |  |  |  |  | 47.56 | 2.5 | 2 |  |  |  |  |  |  |
| Dynactin subunit 1 | DCTN1_HUMAN | 142348 | 5.62 | 66.24 | 1.4 | 2 |  |  |  |  |  |  |  |  |  |  |  |  |  |  |  |
| Dynein heavy chain 10. axonemal | DYH10_HUMAN | 517705 | 5.64 |  |  |  |  |  |  |  |  |  | 143.08 | 0.9 | 5 |  |  |  |  |  |  |
| Dynein heavy chain 11. axonemal | DYH11_HUMAN | 524873 | 6.03 |  |  |  |  |  |  |  |  |  | 122.76 | 0.8 | 5 |  |  |  |  |  |  |
| Dynein heavy chain 17. axonemal | DYH17_HUMAN | 515280 | 5.56 | 63.13 | 0.5 | 3 |  |  |  |  |  |  |  |  |  |  |  |  |  |  |  |
| Dynein heavy chain 2. axonemal | DYH2_HUMAN | 510796 | 5.98 |  |  |  |  |  |  |  |  |  | 77.42 | 0.7 | 3 |  |  |  |  |  |  |
| Dynein heavy chain 3. axonemal | DYH3_HUMAN | 473776 | 6.04 |  |  |  |  |  |  |  |  |  |  |  |  | 75.86 | 0.6 | 3 |  |  |  |
| Dynein heavy chain 5. axonemal | DYH5_HUMAN | 532504 | 5.80 | 47.08 | 0.4 | 2 |  |  |  | 69.57 | 0.4 | 2 |  |  |  |  |  |  |  |  |  |
| Dynein heavy chain 6. axonemal | DYH6_HUMAN | 479671 | 5.72 |  |  |  | 57.79 | 0.4 | 2 |  |  |  | 83.51 | 0.6 | 3 |  |  |  |  |  |  |
| Dynein heavy chain domain-containing protein 1 | DNHD1_HUMAN | 539463 | 6.25 |  |  |  |  |  |  | 63.45 | 0.3 | 2 | 85.73 | 0.5 | 3 |  |  |  |  |  |  |
| Dystonin | DYST_HUMAN | 865259 | 5.14 |  |  |  |  |  |  | 77.65 | 0.3 | 3 | 197.62 | 1 | 8 | 100.24 | 0.5 | 4 |  |  |  |
| Dystroglycan | DAG1_HUMAN | 97723 | 8.71 |  |  |  |  |  |  |  |  |  | 276.53 | 6.6 | 5 |  |  |  |  |  |  |
| Dystrophin | DMD_HUMAN | 428537 | 5.64 |  |  |  | 51.6 | 0.6 | 2 |  |  |  | 76.72 | 0.8 | 3 |  |  |  |  |  |  |
| E3 ubiquitin-protein ligase DTX3L | DTX3L_HUMAN | 84585 | 8.31 |  |  |  |  |  |  |  |  |  | 76.96 | 1.8 | 3 |  |  |  |  |  |  |
| E3 ubiquitin-protein ligase RBBP6 | RBBP6_HUMAN | 202354 | 9.66 |  |  |  |  |  |  |  |  |  | 64.96 | 1.1 | 3 |  |  |  |  |  |  |
| E3 ubiquitin-protein ligase SHPRH | SHPRH_HUMAN | 195580 | 7.30 | 82.79 | 0.8 | 3 |  |  |  | 99.82 | 1.5 | 4 | 76.33 | 1.4 | 3 |  |  |  |  |  |  |
| E3 ubiquitin-protein ligase UBR4* | UBR4_HUMAN | 580547 | 5.70 |  |  |  |  |  |  |  |  |  | 62.16 | 0.4 | 2 |  |  |  |  |  |  |
| Early endosome antigen 1 | EEA1_HUMAN | 163337 | 5.55 | 85.26 | 1.6 | 3 |  |  |  |  |  |  |  |  |  |  |  |  |  |  |  |
| Echinoderm microtubule-associated protein-like 2* | EMAL2_HUMAN | 71603 | 5.87 |  |  |  |  |  |  |  |  |  | 55.2 | 2.5 | 2 |  |  |  |  |  |  |
| Ecto-NOX disulfide-thiol exchanger 1* | ENOX1_HUMAN | 73815 | 5.41 |  |  |  |  |  |  |  |  |  | 52 | 1.2 | 2 |  |  |  |  |  |  |
| Ectonucleotide pyrophosphatase/phosphodiesterase family member 2 | ENPP2_HUMAN | 100811 | 7.14 | 1210.7 | 37.7 | 26 | 771.4 | 27.7 | 18 |  |  |  | 675.59 | 22.9 | 16 |  |  |  |  |  |  |
| EF-hand domain-containing protein C17orf57 | CQ057_HUMAN | 111143 | 6.15 | 66.89 | 2.4 | 3 |  |  |  |  |  |  |  |  |  |  |  |  |  |  |  |
| EGF-containing fibulin-like extracellular matrix protein 1 | FBLN3_HUMAN | 56885 | 4.96 | 199.33 | 10.1 | 4 |  |  |  | 110.66 | 5.3 | 3 | 499.85 | 21.3 | 8 | 542.97 | 22.9 | 10 | 77.05 | 5.7 | 2 |
| EGF-containing fibulin-like extracellular matrix protein 2 | FBLN4_HUMAN | 51767 | 4.79 |  |  |  |  |  |  |  |  |  | 104.3 | 6.3 | 3 |  |  |  |  |  |  |
| ELKS/Rab6-interacting/CAST family member 1 | RB6I2_HUMAN | 128236 | 5.72 |  |  |  |  |  |  |  |  |  | 69.98 | 3 | 3 | 52.57 | 1.7 | 2 |  |  |  |
| Elongation factor 1-alpha 1 | EF1A1_HUMAN | 50451 | 9.10 | 166.31 | 7.6 | 4 | 276.39 | 16.9 | 7 | 96.5 | 4.8 | 2 | 254.77 | 15.8 | 6 |  |  |  |  |  |  |
| Elongation factor 2 | EF2_HUMAN | 96246 | 6.42 | 330.97 | 9.4 | 8 | 283.3 | 8.9 | 8 | 52.4 | 2.1 | 2 | 207.49 | 5.8 | 6 |  |  |  | 53.27 | 1.7 | 2 |
| EMILIN-3 | EMIL3_HUMAN | 83622 | 7.84 |  |  |  |  |  |  |  |  |  | 78.73 | 2.3 | 3 |  |  |  |  |  |  |
| Endoplasmic reticulum aminopeptidase 1 | ERAP1_HUMAN | 107736 | 6.02 |  |  |  |  |  |  |  |  |  | 160.58 | 5.2 | 4 |  |  |  |  |  |  |
| Endoplasmin* | ENPL_HUMAN | 92696 | 4.76 |  |  |  | 90.32 | 2.6 | 2 |  |  |  |  |  |  |  |  |  |  |  |  |
| Engulfment and cell motility protein 1 | ELMO1_HUMAN | 84517 | 5.89 |  |  |  |  |  |  |  |  |  | 80.14 | 3.2 | 3 |  |  |  |  |  |  |
| Enhancer of mRNA-decapping protein 4 | EDC4_HUMAN | 152992 | 5.56 |  |  |  |  |  |  |  |  |  | 81.43 | 1.6 | 3 |  |  |  |  |  |  |
| Enolase-phosphatase E1 | ENOPH_HUMAN | 29086 | 4.66 |  |  |  | 82.14 | 17.6 | 3 |  |  |  | 293.01 | 28 | 5 | 154.15 | 30.7 | 5 |  |  |  |
| Epididymal secretory protein E1 | NPC2_HUMAN | 16902 | 7.57 |  |  |  |  |  |  |  |  |  |  |  |  |  |  |  | 89.3 | 17.2 | 2 |
| Epoxide hydrolase 2 | HYES_HUMAN | 63316 | 5.91 | 374.81 | 22.5 | 8 |  |  |  | 83.48 | 5.4 | 2 | 174.41 | 8.1 | 4 |  |  |  |  |  |  |
| Erythrocyte band 7 integral membrane protein | STOM_HUMAN | 31882 | 7.72 |  |  |  |  |  |  |  |  |  | 77.12 | 9 | 3 |  |  |  |  |  |  |
| Eukaryotic initiation factor 4A-I | IF4A1_HUMAN | 46353 | 5.32 |  |  |  | 176.38 | 7.6 | 3 | 99.21 | 5.7 | 2 | 292.87 | 17 | 6 | 131.22 | 7.1 | 2 | 266.55 | 15 | 5 |
| Eukaryotic initiation factor 4A-II | IF4A2_HUMAN | 46601 | 5.33 |  |  |  |  |  |  |  |  |  | 293.48 | 17.4 | 6 |  |  |  |  |  |  |
| Eukaryotic translation initiation factor 2 subunit 1 | IF2A_HUMAN | 36374 | 5.02 |  |  |  |  |  |  |  |  |  | 123.15 | 7 | 2 |  |  |  |  |  |  |
| Excitatory amino acid transporter 1* | EAA1_HUMAN | 59705 | 8.53 |  |  |  |  |  |  | 55.75 | 1.3 | 2 |  |  |  |  |  |  |  |  |  |
| Exportin-2 | XPO2_HUMAN | 111145 | 5.51 | 155.58 | 4.4 | 4 | 93.08 | 2.8 | 3 |  |  |  |  |  |  |  |  |  |  |  |  |
| Exportin-T | XPOT_HUMAN | 111148 | 5.25 | 63.17 | 2 | 2 |  |  |  |  |  |  |  |  |  |  |  |  |  |  |  |
| Extracellular matrix protein 1 | ECM1_HUMAN | 62232 | 6.25 |  |  |  |  |  |  |  |  |  | 219.31 | 9.6 | 4 |  |  |  |  |  |  |
| Extracellular superoxide dismutase [Cu-Zn] | SODE_HUMAN | 26291 | 6.14 |  |  |  | 265.73 | 21.2 | 4 | 170.93 | 14.6 | 3 | 349.52 | 28.3 | 6 |  |  |  |  |  |  |
| F-actin-capping protein subunit alpha-1 | CAZA1_HUMAN | 33073 | 5.45 | 125.57 | 15 | 3 | 130.52 | 14 | 4 |  |  |  | 460.1 | 47.9 | 9 |  |  |  |  |  |  |
| F-actin-capping protein subunit alpha-2 | CAZA2_HUMAN | 33157 | 5.58 | 228.64 | 24.5 | 5 | 183.09 | 14 | 4 |  |  |  | 366.93 | 32.9 | 7 |  |  |  |  |  |  |
| F-actin-capping protein subunit beta | CAPZB_HUMAN | 31616 | 5.37 |  |  |  |  |  |  | 197.64 | 19.1 | 4 | 464.52 | 28.2 | 8 |  |  |  |  |  |  |
| Far upstream element-binding protein 2* | FUBP2_HUMAN | 73355 | 6.85 |  |  |  |  |  |  |  |  |  | 59.78 | 2.8 | 2 |  |  |  |  |  |  |
| Farnesyl pyrophosphate synthase* | FPPS_HUMAN | 48758 | 5.84 | 50.1 | 5.5 | 2 |  |  |  |  |  |  | 71.84 | 6.4 | 2 |  |  |  |  |  |  |
| Fas-binding factor 1* | FBF1_HUMAN | 125769 | 6.62 |  |  |  | 59.14 | 1.1 | 2 |  |  |  |  |  |  |  |  |  |  |  |  |
| Fatty acid synthase | FAS_HUMAN | 275877 | 6.02 | 351.95 | 4.1 | 7 |  |  |  |  |  |  | 81.65 | 0.9 | 3 |  |  |  |  |  |  |
| Fatty acid-binding protein. epidermal | FABP5_HUMAN | 15497 | 6.60 | 630.14 | 74.8 | 12 | 501.93 | 50.4 | 10 | 462.68 | 58.5 | 9 | 390.38 | 69.6 | 9 | 222.31 | 39.3 | 5 | 448.58 | 74.1 | 10 |
| Ferritin heavy chain | FRIH_HUMAN | 21383 | 5.31 | 83.31 | 10.9 | 2 |  |  |  | 187.36 | 23.5 | 4 | 308.78 | 30.6 | 5 |  |  |  |  |  |  |
| Ferritin light chain | FRIL_HUMAN | 20064 | 5.51 | 367.36 | 50.9 | 9 | 261.4 | 38.3 | 5 | 423.16 | 40 | 7 | 590.74 | 58.9 | 11 | 238.25 | 32.6 | 5 |  |  |  |
| Fetuin-B* | FETUB_HUMAN | 42883 | 6.47 |  |  |  |  |  |  |  |  |  | 65.24 | 6.8 | 2 |  |  |  |  |  |  |
| Fibrillin-1 | FBN1_HUMAN | 332664 | 4.81 | 333.09 | 3.9 | 10 |  |  |  |  |  |  |  |  |  |  |  |  |  |  |  |
| Fibrillin-2 | FBN2_HUMAN | 335257 | 4.74 |  |  |  |  |  |  |  |  |  | 68.97 | 0.9 | 3 |  |  |  |  |  |  |
| Fibrinogen alpha chain | FIBA_HUMAN | 95656 | 5.71 | 142.63 | 3.3 | 2 |  |  |  |  |  |  | 257.56 | 7.3 | 6 |  |  |  |  |  |  |
| Fibrinogen beta chain | FIBB_HUMAN | 56577 | 8.55 | 384.85 | 20 | 8 |  |  |  |  |  |  | 253.96 | 12.8 | 5 |  |  |  |  |  |  |
| Fibrinogen gamma chain | FIBG_HUMAN | 52106 | 5.37 | 576.36 | 36.6 | 12 |  |  |  |  |  |  | 552.13 | 30.9 | 13 |  |  |  |  |  |  |
| Fibronectin | FINC_HUMAN | 266052 | 5.47 | 1723.8 | 22 | 39 | 624.73 | 5.7 | 12 | 551.59 | 5.6 | 10 | 1121.09 | 14.2 | 23 |  |  |  |  |  |  |
| Fibulin-1 | FBLN1_HUMAN | 81268 | 5.07 | 167.05 | 5.8 | 4 | 171.09 | 7.7 | 4 | 356.02 | 10.8 | 5 | 372.19 | 11.8 | 6 |  |  |  |  |  |  |
| Filaggrin | FILA_HUMAN | 435036 | 9.24 | 97.63 | 0.7 | 3 | 110.76 | 0.9 | 3 | 223.64 | 2.2 | 6 | 501.87 | 5.8 | 17 | 85.16 | 0.4 | 2 |  |  |  |
| Filaggrin-2 | FILA2_HUMAN | 249296 | 8.46 | 205.84 | 2.8 | 4 | 237.37 | 3.3 | 5 | 448.04 | 6 | 10 | 712.31 | 9.2 | 15 | 228.38 | 2.8 | 4 |  |  |  |
| Filamin-A | FLNA_HUMAN | 283301 | 5.70 | 2450.63 | 25.5 | 50 | 1262.98 | 11.7 | 29 | 1193.37 | 11.3 | 24 | 1153.46 | 15 | 31 | 160.19 | 1.7 | 4 |  |  |  |
| Filamin-B | FLNB_HUMAN | 280157 | 5.47 | 305.9 | 3.4 | 7 |  |  |  |  |  |  |  |  |  |  |  |  |  |  |  |
| Filensin | BFSP1_HUMAN | 74784 | 5.09 | 1504.91 | 44.2 | 27 | 2524.5 | 62.9 | 44 | 2010.02 | 53.4 | 34 | 3173.31 | 68.1 | 48 | 2399.24 | 71 | 41 | 1722.14 | 53.7 | 31 |
| Follistatin-related protein 1 | FSTL1_HUMAN | 36103 | 5.39 |  |  |  | 67.51 | 4.9 | 2 |  |  |  | 108.13 | 7.5 | 3 | 79.29 | 5.2 | 2 |  |  |  |
| Follistatin-related protein 4 | FSTL4_HUMAN | 94291 | 5.88 |  |  |  |  |  |  |  |  |  | 406.47 | 13.5 | 9 |  |  |  |  |  |  |
| Follistatin-related protein 5 | FSTL5_HUMAN | 97002 | 5.62 | 157.59 | 5.1 | 4 | 559.27 | 16.8 | 14 |  |  |  | 1390.92 | 36.1 | 28 |  |  |  |  |  |  |
| Four and a half LIM domains protein 1* | FHL1_HUMAN | 38006 | 9.25 |  |  |  |  |  |  |  |  |  | 79.28 | 7.1 | 2 |  |  |  |  |  |  |
| Fructose-bisphosphate aldolase A | ALDOA_HUMAN | 39851 | 8.30 | 1681.07 | 88.2 | 26 | 1541.4 | 77.7 | 23 | 1564.83 | 85.4 | 24 | 1630.68 | 79.7 | 24 | 1041.27 | 51.1 | 18 | 1020.53 | 72 | 20 |
| Fructose-bisphosphate aldolase C | ALDOC_HUMAN | 39830 | 6.42 | 1417.52 | 65.1 | 22 | 1254.53 | 66.5 | 20 | 1117.86 | 58 | 18 | 1521.99 | 70.6 | 22 | 691.62 | 41.5 | 12 | 761.91 | 43.4 | 14 |
| Fumarylacetoacetase | FAAA_HUMAN | 46743 | 6.47 | 584.89 | 37.9 | 12 | 470.83 | 27.2 | 10 | 224.2 | 11.9 | 4 | 434.59 | 26.3 | 9 | 127.15 | 7.6 | 2 | 96.6 | 8.1 | 3 |
| FYVE and coiled-coil domain-containing protein 1 | FYCO1_HUMAN | 168590 | 4.86 |  |  |  |  |  |  |  |  |  |  |  |  | 52.85 | 0.8 | 2 |  |  |  |
| G2/mitotic-specific cyclin-B3* | CCNB3_HUMAN | 159013 | 6.28 |  |  |  |  |  |  |  |  |  | 48.8 | 1.4 | 2 |  |  |  |  |  |  |
| Galectin-1 | LEG1_HUMAN | 15048 | 5.34 | 272.19 | 51.1 | 6 | 399.41 | 51.1 | 6 | 334.45 | 47.4 | 6 | 402.02 | 58.5 | 7 | 373.81 | 58.5 | 8 | 224.03 | 31.9 | 3 |
| Galectin-3-binding protein | LG3BP_HUMAN | 66202 | 5.14 | 199.41 | 15 | 6 | 270.3 | 14.5 | 8 | 370.72 | 17.4 | 9 | 829.74 | 30.4 | 17 | 270.17 | 11.1 | 5 |  |  |  |
| Galectin-7 | LEG7_HUMAN | 15123 | 7.04 |  |  |  | 143.3 | 29.4 | 4 |  |  |  |  |  |  |  |  |  |  |  |  |
| Gamma-crystallin A | CRGA_HUMAN | 21376 | 8.05 | 87.77 | 14.4 | 2 |  |  |  |  |  |  |  |  |  |  |  |  |  |  |  |
| Gamma-crystallin B | CRGB_HUMAN | 21293 | 6.88 | 762.36 | 84 | 12 | 695.75 | 78.9 | 11 | 586.2 | 70.9 | 10 | 537.44 | 54.9 | 9 | 613.99 | 62.9 | 10 | 591.47 | 74.3 | 10 |
| Gamma-crystallin C | CRGC_HUMAN | 21321 | 6.88 | 891.95 | 83.9 | 13 | 946.36 | 87.4 | 15 | 837.33 | 79.3 | 12 | 853.81 | 86.8 | 14 | 816.59 | 79.9 | 12 | 902.31 | 83.9 | 14 |
| Gamma-crystallin D | CRGD_HUMAN | 21067 | 7.00 | 990.26 | 81.6 | 13 | 1162.56 | 87.4 | 17 | 882.03 | 79.3 | 12 | 1148.37 | 85.1 | 15 | 1038.29 | 81.6 | 14 | 931.4 | 81 | 15 |
| Gamma-enolase | ENOG_HUMAN | 47581 | 4.91 | 1422.62 | 68.4 | 22 | 1015.46 | 45.6 | 14 | 1130.56 | 54.1 | 16 | 1457.11 | 73.7 | 22 | 879.31 | 48.8 | 15 | 337.85 | 23.3 | 7 |
| Gamma-glutamyl hydrolase* | GGH_HUMAN | 36340 | 6.67 |  |  |  |  |  |  |  |  |  | 100.13 | 6.9 | 2 |  |  |  |  |  |  |
| Ganglioside GM2 activator | SAP3_HUMAN | 21281 | 5.17 |  |  |  |  |  |  |  |  |  | 92.29 | 13 | 3 |  |  |  |  |  |  |
| Gap junction alpha-3 protein | CXA3_HUMAN | 47779 | 6.16 | 117.3 | 4.8 | 2 | 285.47 | 11 | 6 | 192.15 | 7.1 | 4 | 230.82 | 12.6 | 6 |  |  |  | 250.19 | 16.8 | 7 |
| Gap junction alpha-8 protein | CXA8_HUMAN | 48655 | 5.22 |  |  |  | 88.46 | 4.6 | 2 |  |  |  |  |  |  |  |  |  |  |  |  |
| Gasdermin-A | GSDMA_HUMAN | 49619 | 5.20 |  |  |  |  |  |  |  |  |  | 125.88 | 5.6 | 3 |  |  |  |  |  |  |
| Gelsolin | GELS_HUMAN | 86043 | 5.90 | 1406.16 | 38.2 | 23 | 1314.02 | 38.1 | 21 | 1435.04 | 38.9 | 23 | 1392.69 | 44.6 | 26 | 50.45 | 2.8 | 2 | 447.45 | 16.6 | 11 |
| General transcription factor IIE subunit 1 | T2EA_HUMAN | 49763 | 4.74 |  |  |  |  |  |  |  |  |  | 70.91 | 5 | 3 |  |  |  |  |  |  |
| Geranylgeranyl pyrophosphate synthase | GGPPS_HUMAN | 35020 | 5.79 |  |  |  |  |  |  |  |  |  | 81.07 | 5 | 2 |  |  |  |  |  |  |
| Geranylgeranyl transferase type-2 subunit alpha | PGTA_HUMAN | 66114 | 5.46 |  |  |  |  |  |  |  |  |  | 94.96 | 4.8 | 3 |  |  |  |  |  |  |
| Geranylgeranyl transferase type-2 subunit beta | PGTB2_HUMAN | 37585 | 4.89 |  |  |  |  |  |  |  |  |  | 66.76 | 5.7 | 2 |  |  |  |  |  |  |
| Girdin | GRDN_HUMAN | 216593 | 5.90 |  |  |  |  |  |  | 110.99 | 1.6 | 4 |  |  |  |  |  |  |  |  |  |
| Glia maturation factor beta | GMFB_HUMAN | 16874 | 5.19 |  |  |  |  |  |  | 61.9 | 22.5 | 2 |  |  |  |  |  |  |  |  |  |
| Glucosamine-6-phosphate isomerase 1 | GNPI1_HUMAN | 32819 | 6.43 |  |  |  | 172.38 | 19.4 | 4 |  |  |  | 502.36 | 50.9 | 12 |  |  |  | 87.31 | 7.6 | 2 |
| Glucosamine-6-phosphate isomerase 2 | GNPI2_HUMAN | 31293 | 6.46 |  |  |  | 125.53 | 15.9 | 3 |  |  |  | 375.48 | 48.6 | 9 |  |  |  |  |  |  |
| Glucosamine--fructose-6-phosphate aminotransferase [isomerizing] 2 | GFPT2_HUMAN | 77680 | 7.03 | 59.06 | 1 | 2 |  |  |  |  |  |  |  |  |  |  |  |  |  |  |  |
| Glucose-6-phosphate 1-dehydrogenase | G6PD_HUMAN | 59675 | 6.39 | 263.74 | 14.8 | 8 | 93.99 | 6.8 | 3 |  |  |  | 726.29 | 37.5 | 19 | 120.53 | 8.3 | 4 | 166.3 | 9.1 | 5 |
| Glucose-6-phosphate isomerase | G6PI_HUMAN | 63335 | 8.43 | 537.25 | 21.1 | 11 | 223.02 | 7.7 | 4 | 457.71 | 15.6 | 7 | 348.64 | 13.4 | 6 | 105.46 | 5 | 2 | 196.27 | 11.5 | 6 |
| Glucosidase 2 subunit beta | GLU2B_HUMAN | 60357 | 4.33 |  |  |  |  |  |  |  |  |  | 108.05 | 7.6 | 4 |  |  |  |  |  |  |
| Glutamate [NMDA] receptor subunit epsilon-2 | NMDE2_HUMAN | 167972 | 6.47 | 68.97 | 1.6 | 3 |  |  |  |  |  |  |  |  |  |  |  |  |  |  |  |
| Glutamate dehydrogenase 1. mitochondrial | DHE3_HUMAN | 61701 | 7.67 |  |  |  |  |  |  |  |  |  | 803.87 | 28 | 16 |  |  |  |  |  |  |
| Glutamate receptor 4 | GRIA4_HUMAN | 101548 | 8.32 |  |  |  |  |  |  |  |  |  | 112.98 | 3.3 | 3 |  |  |  |  |  |  |
| Glutaminyl-peptide cyclotransferase | QPCT_HUMAN | 40965 | 6.12 |  |  |  |  |  |  |  |  |  | 122.56 | 14.1 | 4 |  |  |  |  |  |  |
| Glutathione peroxidase 3 | GPX3_HUMAN | 25765 | 8.26 | 107.55 | 19.5 | 3 | 276.41 | 35 | 7 | 290.37 | 34.1 | 7 | 294.46 | 28.8 | 6 | 276.64 | 28.8 | 6 | 184.31 | 25.7 | 5 |
| Glutathione reductase. mitochondrial | GSHR_HUMAN | 56791 | 8.74 | 678.71 | 40.2 | 15 | 239.56 | 14.9 | 6 | 388.67 | 24.5 | 10 | 459.44 | 25.1 | 10 |  |  |  | 85.61 | 4.4 | 2 |
| Glutathione S-transferase Mu 2 | GSTM2_HUMAN | 25899 | 6.00 | 207.49 | 21.6 | 3 | 222.16 | 21.1 | 4 | 116.81 | 14.2 | 2 | 175.46 | 18.3 | 3 |  |  |  |  |  |  |
| Glutathione S-transferase Mu 3 | GSTM3_HUMAN | 26998 | 5.38 |  |  |  | 127.08 | 11.1 | 2 |  |  |  | 338.37 | 32 | 6 | 197.24 | 17.3 | 4 |  |  |  |
| Glutathione S-transferase P | GSTP1_HUMAN | 23569 | 5.44 |  |  |  | 85.5 | 14.8 | 2 | 89.23 | 14.8 | 2 |  |  |  |  |  |  |  |  |  |
| Glutathione synthetase | GSHB_HUMAN | 52523 | 5.67 | 1761.62 | 67.5 | 31 | 1460.15 | 67.1 | 27 | 1509.55 | 66.9 | 27 | 1726.84 | 66.9 | 30 | 1001.68 | 43 | 22 | 1029.41 | 46.6 | 20 |
| Glyceraldehyde-3-phosphate dehydrogenase | G3P_HUMAN | 36201 | 8.57 | 1474.21 | 79.7 | 20 | 1546.88 | 84.5 | 22 | 616.08 | 36.7 | 9 | 1533.5 | 86 | 23 | 1464.55 | 79.7 | 20 | 1130.93 | 73.1 | 17 |
| Glyceraldehyde-3-phosphate dehydrogenase. testis-specific | G3PT_HUMAN | 44815 | 8.38 |  |  |  | 66.6 | 4.4 | 2 |  |  |  |  |  |  |  |  |  |  |  |  |
| Glycerol-3-phosphate dehydrogenase [NAD(+)]. cytoplasmic | GPDA_HUMAN | 38171 | 5.81 | 80.65 | 9.2 | 3 | 52.09 | 11.7 | 2 |  |  |  | 336.44 | 30.7 | 10 |  |  |  |  |  |  |
| Glycerol-3-phosphate dehydrogenase 1-like protein | GPD1L_HUMAN | 39021 | 6.62 |  |  |  |  |  |  |  |  |  | 219.98 | 21.7 | 6 |  |  |  |  |  |  |
| Glycine--tRNA ligase | SYG_HUMAN | 83854 | 6.62 | 127.08 | 4.6 | 3 |  |  |  |  |  |  | 251.83 | 11.9 | 6 |  |  |  |  |  |  |
| Glycogen phosphorylase. brain form | PYGB_HUMAN | 97319 | 6.40 | 999.95 | 31.3 | 25 | 1385.72 | 39.7 | 29 | 855.15 | 26.5 | 18 | 1241.36 | 34.2 | 26 | 529.48 | 17.3 | 13 | 427.18 | 11.7 | 11 |
| Glycogen phosphorylase. muscle form | PYGM_HUMAN | 97487 | 6.58 |  |  |  | 295.41 | 10.9 | 8 |  |  |  | 1479.08 | 37.8 | 34 |  |  |  |  |  |  |
| Glyoxalase domain-containing protein 4 | GLOD4_HUMAN | 35170 | 5.40 |  |  |  |  |  |  |  |  |  | 236.96 | 16.3 | 5 |  |  |  |  |  |  |
| Glyoxylate reductase/hydroxypyruvate reductase | GRHPR_HUMAN | 36045 | 7.02 |  |  |  |  |  |  |  |  |  | 132.7 | 15.2 | 3 |  |  |  |  |  |  |
| GMP reductase 2 | GMPR2_HUMAN | 38363 | 6.80 |  |  |  |  |  |  |  |  |  | 190.19 | 13.5 | 6 |  |  |  |  |  |  |
| Golgi resident protein GCP60* | GCP60_HUMAN | 60841 | 5.02 |  |  |  |  |  |  |  |  |  | 49.75 | 3.2 | 2 |  |  |  |  |  |  |
| Golgin subfamily A member 1* | GOGA1_HUMAN | 88244 | 5.24 |  |  |  |  |  |  |  |  |  | 61.13 | 2.2 | 2 |  |  |  |  |  |  |
| Golgin subfamily A member 3 | GOGA3_HUMAN | 167765 | 5.34 | 53.91 | 0.9 | 2 |  |  |  | 82.17 | 0.5 | 2 |  |  |  |  |  |  |  |  |  |
| Golgin subfamily B member 1 | GOGB1_HUMAN | 377215 | 4.96 |  |  |  |  |  |  | 98.17 | 1.1 | 4 |  |  |  |  |  |  |  |  |  |
| Golgi-specific brefeldin A-resistance guanine nucleotide exchange factor 1* | GBF1_HUMAN | 208367 | 5.48 |  |  |  |  |  |  | 59.84 | 0.9 | 2 |  |  |  |  |  |  |  |  |  |
| GTP-binding nuclear protein Ran | RAN_HUMAN | 24579 | 7.02 | 246.2 | 31 | 6 | 151.56 | 23.6 | 4 | 230.57 | 27.8 | 6 | 249.89 | 27.8 | 6 |  |  |  | 95.28 | 14.8 | 3 |
| Guanine nucleotide-binding protein G(I)/G(S)/G(T) subunit beta-1 | GBB1_HUMAN | 38151 | 5.60 | 48.78 | 5.3 | 2 | 113.61 | 7.9 | 3 |  |  |  | 177.19 | 11.8 | 4 |  |  |  |  |  |  |
| Guanine nucleotide-binding protein G(s) subunit alpha isoforms XLas | GNAS1_HUMAN | 111697 | 4.91 |  |  |  |  |  |  |  |  |  | 93.29 | 2.8 | 3 |  |  |  |  |  |  |
| Guanine nucleotide-binding protein subunit beta-2-like 1* | GBLP_HUMAN | 35511 | 7.60 |  |  |  |  |  |  |  |  |  | 86.93 | 6.6 | 2 |  |  |  |  |  |  |
| Haloacid dehalogenase-like hydrolase domain-containing protein 2 | HDHD2_HUMAN | 28746 | 5.85 |  |  |  |  |  |  |  |  |  | 167.03 | 16.2 | 3 |  |  |  |  |  |  |
| Haptoglobin | HPT_HUMAN | 45861 | 6.14 | 1193.32 | 60.1 | 23 | 1286.75 | 56.9 | 23 | 1079.19 | 50.2 | 20 | 1290.13 | 50.5 | 21 | 920.79 | 40.1 | 16 | 1040.27 | 55.2 | 21 |
| HAUS augmin-like complex subunit 5 | HAUS5_HUMAN | 72265 | 8.83 |  |  |  |  |  |  |  |  |  | 72.84 | 4.4 | 3 |  |  |  |  |  |  |
| HEAT repeat-containing protein 1 | HEAT1_HUMAN | 244382 | 6.12 |  |  |  |  |  |  |  |  |  | 60.74 | 0.7 | 2 |  |  |  |  |  |  |
| Heat shock 70 kDa protein 4 | HSP74_HUMAN | 95127 | 5.11 | 434.69 | 11 | 8 | 90.61 | 3.5 | 3 |  |  |  | 644.04 | 18.9 | 13 |  |  |  |  |  |  |
| Heat shock cognate 71 kDa protein | HSP7C_HUMAN | 71082 | 5.38 | 1141.91 | 34.1 | 21 | 309.34 | 11.3 | 6 | 136.6 | 5.6 | 2 | 1007.66 | 34.4 | 19 | 62.5 | 2.3 | 2 |  |  |  |
| Heat shock protein beta-1 | HSPB1_HUMAN | 22826 | 5.98 | 801.33 | 87.3 | 15 | 954.47 | 90.7 | 17 | 859.07 | 87.3 | 16 | 910.32 | 86.8 | 16 | 638.42 | 82.4 | 12 | 431.09 | 73.2 | 9 |
| Heat shock protein HSP 90-alpha | HS90A_HUMAN | 85006 | 4.94 | 1306.18 | 35.7 | 28 | 1103 | 32.4 | 25 | 1254.87 | 37.7 | 27 | 1241.87 | 33.9 | 26 | 621.72 | 18 | 12 | 585.9 | 21 | 15 |
| Heat shock protein HSP 90-beta | HS90B_HUMAN | 83554 | 4.98 | 1023.34 | 31.2 | 21 | 672.22 | 20.9 | 16 | 872.11 | 25.3 | 19 | 911.31 | 26.1 | 19 | 480.05 | 12.7 | 9 |  |  |  |
| Helicase-like transcription factor | HLTF_HUMAN | 114883 | 8.83 |  |  |  |  |  |  |  |  |  | 59.04 | 1.6 | 2 |  |  |  |  |  |  |
| Heme-binding protein 2 | HEBP2_HUMAN | 22861 | 4.58 |  |  |  |  |  |  |  |  |  | 273.47 | 24.4 | 5 | 139.21 | 10.7 | 2 |  |  |  |
| Hemicentin-1 | HMCN1_HUMAN | 623265 | 6.07 |  |  |  |  |  |  | 67.47 | 0.6 | 3 | 98.08 | 0.8 | 4 |  |  |  |  |  |  |
| Hemoglobin subunit alpha | HBA_HUMAN | 15305 | 8.72 | 469.52 | 91.5 | 10 | 147.97 | 24.6 | 3 |  |  |  | 293.17 | 43 | 6 |  |  |  |  |  |  |
| Hemoglobin subunit beta | HBB_HUMAN | 16102 | 6.75 | 721.72 | 93.9 | 12 | 146.94 | 34 | 4 |  |  |  | 667.64 | 83 | 11 |  |  |  |  |  |  |
| Hemopexin | HEMO_HUMAN | 52385 | 6.55 | 1228.47 | 65.6 | 24 | 1054.58 | 55 | 19 | 1076.42 | 60 | 20 | 1223.81 | 67.7 | 23 | 716.82 | 51.7 | 16 | 943.69 | 53.9 | 19 |
| Heparin cofactor 2 | HEP2_HUMAN | 57205 | 6.41 | 203.26 | 10.2 | 6 | 234.37 | 12 | 6 | 126.98 | 4.2 | 3 | 164.46 | 8.2 | 5 |  |  |  |  |  |  |
| Hepatocyte growth factor activator | HGFA_HUMAN | 72860 | 6.99 | 121.35 | 4.9 | 2 |  |  |  |  |  |  |  |  |  |  |  |  |  |  |  |
| Histidine-rich glycoprotein | HRG_HUMAN | 60510 | 7.09 | 493.12 | 25 | 11 | 74.6 | 4 | 2 | 199.87 | 8.8 | 4 | 131.96 | 7.4 | 3 |  |  |  |  |  |  |
| Histidine--tRNA ligase. cytoplasmic* | SYHC_HUMAN | 57944 | 5.72 |  |  |  |  |  |  |  |  |  | 50.69 | 3.3 | 2 |  |  |  |  |  |  |
| Histone deacetylase 11* | HDA11_HUMAN | 39273 | 7.18 |  |  |  |  |  |  |  |  |  | 50.95 | 2 | 2 |  |  |  |  |  |  |
| Histone H2A type 1-B/E | H2A1B_HUMAN | 14127 | 11.06 |  |  |  |  |  |  |  |  |  | 117.98 | 26.9 | 3 |  |  |  |  |  |  |
| Histone H2B type 1-B | H2B1B_HUMAN | 13942 | 10.31 |  |  |  | 169.73 | 24.6 | 3 |  |  |  |  |  |  |  |  |  |  |  |  |
| Histone H2B type 1-D | H2B1D_HUMAN | 13928 | 10.31 |  |  |  |  |  |  |  |  |  | 176.68 | 33.3 | 4 |  |  |  |  |  |  |
| Histone H4 | H4_HUMAN | 11360 | 11.36 |  |  |  | 114.11 | 29.1 | 3 |  |  |  | 140.53 | 27.2 | 3 |  |  |  |  |  |  |
| Histone-lysine N-methyltransferase ASH1L | ASH1L_HUMAN | 336174 | 9.46 |  |  |  |  |  |  |  |  |  | 73.06 | 0.5 | 3 |  |  |  |  |  |  |
| Histone-lysine N-methyltransferase MLL4 | MLL4_HUMAN | 297664 | 8.59 |  |  |  |  |  |  |  |  |  | 85.98 | 1.2 | 3 |  |  |  |  |  |  |
| Homeobox protein cut-like 1* | CUX1_HUMAN | 164544 | 5.72 |  |  |  |  |  |  |  |  |  | 52.02 | 1 | 2 |  |  |  |  |  |  |
| Hornerin | HORN_HUMAN | 283140 | 10.05 | 308.81 | 4.4 | 6 | 404.03 | 3.8 | 6 | 817.35 | 8.6 | 16 | 1033.39 | 11.3 | 20 | 328.51 | 4.8 | 8 |  |  |  |
| Hyaluronan-binding protein 2 | HABP2_HUMAN | 64740 | 6.09 | 116.39 | 4.6 | 3 |  |  |  |  |  |  | 110.72 | 4.6 | 3 |  |  |  |  |  |  |
| Hydroxyacylglutathione hydrolase. mitochondrial | GLO2_HUMAN | 34240 | 8.35 | 186.01 | 22.4 | 5 | 88.42 | 5.8 | 2 | 84.63 | 5.8 | 2 | 197.33 | 14.9 | 4 |  |  |  |  |  |  |
| Iduronate 2-sulfatase | IDS_HUMAN | 62233 | 5.22 |  |  |  |  |  |  |  |  |  | 419.05 | 23.5 | 10 | 117.05 | 6.9 | 2 |  |  |  |
| Ig alpha-1 chain C region | IGHA1_HUMAN | 38486 | 6.09 | 860.77 | 54.4 | 13 | 456.59 | 28.3 | 8 | 357.24 | 22.4 | 7 | 1136.22 | 66 | 20 | 194.03 | 13.6 | 4 | 237.2 | 29.5 | 7 |
| Ig alpha-2 chain C region | IGHA2_HUMAN | 37301 | 5.72 | 614.59 | 42.1 | 10 |  |  |  |  |  |  | 780.32 | 47.6 | 14 |  |  |  |  |  |  |
| Ig gamma-1 chain C region | IGHG1_HUMAN | 36596 | 8.47 | 897.26 | 61.8 | 14 | 936.42 | 55.8 | 14 | 869.94 | 54.8 | 13 | 1083.65 | 66.7 | 17 | 934.09 | 60.3 | 14 | 700.42 | 50.9 | 11 |
| Ig gamma-2 chain C region | IGHG2_HUMAN | 36505 | 7.66 | 697.2 | 52.8 | 13 | 507.97 | 36.2 | 9 | 613.54 | 45.4 | 11 | 553.85 | 42 | 10 | 524.34 | 35.3 | 9 | 395.69 | 34.7 | 8 |
| Ig gamma-3 chain C region | IGHG3_HUMAN | 42287 | 8.24 | 776.45 | 41.9 | 13 | 732.47 | 39.5 | 13 | 578.59 | 34 | 11 | 772.84 | 41.1 | 14 | 560.57 | 26.5 | 10 | 572.46 | 38.5 | 11 |
| Ig gamma-4 chain C region | IGHG4_HUMAN | 36431 | 7.18 | 621.05 | 47.4 | 10 | 580.9 | 35.2 | 9 | 489.67 | 34.3 | 8 | 598.37 | 35.8 | 9 |  |  |  | 415.94 | 34.3 | 8 |
| Ig heavy chain V-II region WAH | HV206_HUMAN | 14222 | 8.62 |  |  |  |  |  |  |  |  |  | 85.27 | 13.2 | 2 |  |  |  |  |  |  |
| Ig heavy chain V-III region BRO | HV305_HUMAN | 13332 | 6.45 | 117.52 | 25 | 2 | 186.58 | 25 | 2 | 176.21 | 25 | 2 | 203.87 | 34.2 | 3 |  |  |  | 115.58 | 34.2 | 3 |
| Ig heavy chain V-III region BUT | HV306_HUMAN | 12485 | 9.34 |  |  |  |  |  |  |  |  |  | 231.55 | 19.1 | 4 |  |  |  |  |  |  |
| Ig heavy chain V-III region CAM | HV307_HUMAN | 13773 | 9.66 | 179.01 | 18 | 3 | 157.1 | 23.8 | 3 | 146.85 | 18 | 2 | 165.1 | 23.8 | 3 |  |  |  | 90.93 | 18 | 2 |
| Ig heavy chain V-III region GA | HV308_HUMAN | 13271 | 9.81 |  |  |  |  |  |  |  |  |  | 88.16 | 24.6 | 2 |  |  |  |  |  |  |
| Ig heavy chain V-III region TIL | HV304_HUMAN | 12462 | 9.24 | 215.21 | 26.1 | 2 | 104.27 | 26.1 | 2 |  |  |  | 185.79 | 26.1 | 2 |  |  |  |  |  |  |
| Ig heavy chain V-III region TUR | HV318_HUMAN | 12537 | 9.78 | 165.42 | 26.7 | 2 |  |  |  |  |  |  |  |  |  |  |  |  |  |  |  |
| Ig heavy chain V-III region VH26 | HV303_HUMAN | 12745 | 8.49 |  |  |  | 121.48 | 18.8 | 2 |  |  |  |  |  |  |  |  |  |  |  |  |
| Ig heavy chain V-III region WEA | HV302_HUMAN | 12363 | 8.71 | 138.87 | 19.3 | 2 |  |  |  |  |  |  | 75.15 | 26.3 | 2 |  |  |  |  |  |  |
| Ig kappa chain C region | IGKC_HUMAN | 11773 | 5.58 | 451.78 | 80.2 | 5 | 614.59 | 80.2 | 6 | 527.81 | 80.2 | 6 | 533.45 | 82.1 | 6 | 489.58 | 86.8 | 6 | 402.93 | 80.2 | 5 |
| Ig kappa chain V-I region AG | KV101_HUMAN | 12099 | 5.68 |  |  |  |  |  |  | 199.91 | 38 | 3 |  |  |  | 116.88 | 23.1 | 2 |  |  |  |
| Ig kappa chain V-I region CAR | KV104_HUMAN | 11810 | 9.48 |  |  |  |  |  |  |  |  |  | 179.05 | 27.1 | 2 |  |  |  |  |  |  |
| Ig kappa chain V-I region DEE | KV105_HUMAN | 11768 | 9.43 | 298.06 | 22.2 | 4 |  |  |  |  |  |  | 433.69 | 22.2 | 5 |  |  |  |  |  |  |
| Ig kappa chain V-I region EU | KV106_HUMAN | 11895 | 8.62 | 161.26 | 32.4 | 3 | 166.94 | 32.4 | 3 | 130.44 | 22.2 | 2 | 209.07 | 32.4 | 3 |  |  |  | 162.98 | 32.4 | 3 |
| Ig kappa chain V-I region Gal* | KV107_HUMAN | 11921 | 9.05 |  |  |  |  |  |  |  |  |  | 162.32 | 22.2 | 2 |  |  |  |  |  |  |
| Ig kappa chain V-I region Hau | KV108_HUMAN | 11778 | 8.69 |  |  |  | 149.27 | 22.2 | 2 |  |  |  |  |  |  |  |  |  | 133.1 | 22.2 | 2 |
| Ig kappa chain V-I region HK102 | KV110_HUMAN | 12931 | 6.07 | 82.74 | 20.5 | 2 |  |  |  |  |  |  |  |  |  |  |  |  |  |  |  |
| Ig kappa chain V-I region OU | KV114_HUMAN | 11884 | 9.95 |  |  |  |  |  |  | 248.23 | 22.2 | 3 |  |  |  |  |  |  |  |  |  |
| Ig kappa chain V-I region WEA | KV118_HUMAN | 11947 | 9.08 |  |  |  |  |  |  |  |  |  | 209.84 | 36.1 | 3 |  |  |  |  |  |  |
| Ig kappa chain V-II region MIL | KV203_HUMAN | 12162 | 9.39 | 159.59 | 33 | 2 | 199.26 | 17.9 | 3 | 186.6 | 17.9 | 3 | 170.99 | 17.9 | 3 |  |  |  |  |  |  |
| Ig kappa chain V-II region RPMI 6410 | KV206_HUMAN | 14811 | 9.34 |  |  |  | 114.62 | 18 | 2 |  |  |  |  |  |  |  |  |  |  |  |  |
| Ig kappa chain V-II region TEW | KV204_HUMAN | 12422 | 5.69 | 185.32 | 32.7 | 2 | 144.47 | 38.9 | 3 |  |  |  |  |  |  |  |  |  |  |  |  |
| Ig kappa chain V-III region NG9 | KV303_HUMAN | 10836 | 6.29 | 85.77 | 22 | 3 |  |  |  | 67.47 | 16 | 2 |  |  |  |  |  |  |  |  |  |
| Ig kappa chain V-III region POM | KV306_HUMAN | 12029 | 9.11 |  |  |  | 58.04 | 13.8 | 2 |  |  |  |  |  |  |  |  |  |  |  |  |
| Ig kappa chain V-III region SIE | KV302_HUMAN | 11882 | 8.70 |  |  |  | 237.92 | 51.4 | 5 |  |  |  |  |  |  |  |  |  | 178.84 | 31.2 | 2 |
| Ig kappa chain V-III region VG | KV309_HUMAN | 12681 | 4.85 | 81.46 | 13 | 2 | 63.67 | 13 | 2 |  |  |  |  |  |  |  |  |  |  |  |  |
| Ig kappa chain V-III region VH | KV310_HUMAN | 12863 | 5.63 | 65.44 | 12.9 | 2 | 52.15 | 12.9 | 2 |  |  |  | 73.98 | 12.9 | 2 |  |  |  |  |  |  |
| Ig kappa chain V-III region WOL | KV305_HUMAN | 11853 | 9.07 | 269.83 | 57.8 | 6 |  |  |  | 259.33 | 52.3 | 5 | 339.29 | 52.3 | 5 | 79.72 | 22.9 | 2 |  |  |  |
| Ig kappa chain V-IV region | KV401_HUMAN | 13486 | 5.09 |  |  |  | 145.99 | 23.1 | 3 | 54.82 | 14.9 | 2 | 151.87 | 29.8 | 3 |  |  |  | 52.99 | 13.2 | 2 |
| Ig kappa chain V-IV region Len | KV402_HUMAN | 12746 | 7.92 | 156.02 | 28.9 | 2 | 194.88 | 29.8 | 3 | 124.44 | 21.1 | 2 | 196.29 | 36.8 | 3 |  |  |  |  |  |  |
| Ig lambda chain V region 4A* | LV001_HUMAN | 12600 | 6.52 |  |  |  |  |  |  |  |  |  | 55.77 | 13.7 | 2 |  |  |  |  |  |  |
| Ig lambda chain V-I region HA | LV102_HUMAN | 12003 | 9.08 |  |  |  |  |  |  | 84.36 | 18.8 | 2 |  |  |  |  |  |  |  |  |  |
| Ig lambda chain V-I region NIG-64* | LV104_HUMAN | 11561 | 4.66 |  |  |  |  |  |  |  |  |  | 50.19 | 14.4 | 2 |  |  |  |  |  |  |
| Ig lambda chain V-I region WAH* | LV106_HUMAN | 11832 | 6.29 |  |  |  | 74.05 | 22 | 2 |  |  |  | 86.56 | 22 | 2 |  |  |  |  |  |  |
| Ig lambda chain V-III region LOI | LV302_HUMAN | 12042 | 4.95 | 166.14 | 29.7 | 3 | 127.47 | 21.6 | 2 |  |  |  | 172.26 | 29.7 | 3 |  |  |  |  |  |  |
| Ig lambda chain V-III region SH | LV301_HUMAN | 11500 | 6.02 | 146.68 | 34.3 | 3 |  |  |  |  |  |  | 62.26 | 17.6 | 2 |  |  |  |  |  |  |
| Ig lambda chain V-IV region Hil | LV403_HUMAN | 11624 | 6.04 | 98.61 | 28 | 2 |  |  |  |  |  |  |  |  |  |  |  |  |  |  |  |
| Ig lambda-1 chain C regions | LAC1_HUMAN | 11512 | 7.89 |  |  |  |  |  |  |  |  |  |  |  |  | 251.26 | 37.7 | 3 | 327.69 | 64.2 | 5 |
| Ig lambda-2 chain C regions | LAC2_HUMAN | 11458 | 6.92 | 530.05 | 93.4 | 8 | 508.77 | 88.7 | 7 | 454.34 | 88.7 | 7 | 536 | 93.4 | 8 | 350.6 | 62.3 | 5 | 407.3 | 82.1 | 6 |
| Ig mu chain C region | IGHM_HUMAN | 49960 | 6.35 |  |  |  |  |  |  |  |  |  | 284.26 | 19.5 | 7 |  |  |  |  |  |  |
| IgGFc-binding protein | FCGBP_HUMAN | 596443 | 5.15 | 1165.94 | 5.2 | 22 | 716.83 | 3.2 | 17 | 329.78 | 1.9 | 9 | 1946.79 | 7.4 | 34 | 539.32 | 3.1 | 14 |  |  |  |
| Immunoglobulin lambda-like polypeptide 5 | IGLL5_HUMAN | 23391 | 9.09 | 449.77 | 44.4 | 7 | 454.08 | 44.4 | 7 | 374.32 | 44.4 | 7 | 510.56 | 46.7 | 8 |  |  |  |  |  |  |
| Importin subunit beta-1 | IMB1_HUMAN | 98420 | 4.69 | 1187.3 | 32.1 | 23 | 1303.45 | 40.4 | 24 | 854.5 | 26 | 17 | 609.26 | 17.8 | 13 | 370.82 | 10.4 | 7 | 243.72 | 8.3 | 6 |
| Importin-5 | IPO5_HUMAN | 125032 | 4.83 | 1973.88 | 41 | 36 | 429.86 | 10.5 | 9 | 163.85 | 4.7 | 4 | 404.23 | 9.8 | 10 | 290.93 | 8.4 | 8 |  |  |  |
| Importin-7* | IPO7_HUMAN | 120751 | 4.70 | 50.51 | 2.4 | 2 |  |  |  |  |  |  |  |  |  |  |  |  |  |  |  |
| Inactive dual specificity phosphatase 27 | DUS27_HUMAN | 130666 | 5.10 | 69.48 | 2.1 | 3 |  |  |  |  |  |  | 90.82 | 2.8 | 4 |  |  |  |  |  |  |
| Inactive tyrosine-protein kinase 7 | PTK7_HUMAN | 119799 | 6.67 |  |  |  |  |  |  |  |  |  | 66.43 | 1.7 | 2 |  |  |  |  |  |  |
| InaD-like protein | INADL_HUMAN | 197046 | 4.84 | 68.25 | 0.8 | 2 |  |  |  |  |  |  |  |  |  |  |  |  |  |  |  |
| Inosine-5'-monophosphate dehydrogenase 1 | IMDH1_HUMAN | 55770 | 6.44 |  |  |  |  |  |  |  |  |  | 98.64 | 6.2 | 3 |  |  |  |  |  |  |
| Inosine-5'-monophosphate dehydrogenase 2 | IMDH2_HUMAN | 56226 | 6.44 |  |  |  |  |  |  |  |  |  | 93.2 | 6 | 3 |  |  |  |  |  |  |
| Inositol 1.4.5-trisphosphate receptor type 1 | ITPR1_HUMAN | 317167 | 5.72 |  |  |  |  |  |  |  |  |  | 100.12 | 1.2 | 4 |  |  |  |  |  |  |
| Inositol 1.4.5-trisphosphate receptor type 3 | ITPR3_HUMAN | 306820 | 6.05 |  |  |  |  |  |  |  |  |  | 69.42 | 1.2 | 3 |  |  |  |  |  |  |
| Inositol monophosphatase 1 | IMPA1_HUMAN | 30568 | 5.17 | 84.18 | 7.6 | 2 |  |  |  |  |  |  | 212.45 | 15.2 | 4 |  |  |  |  |  |  |
| Insulin-like growth factor-binding protein 7* | IBP7_HUMAN | 30138 | 8.25 |  |  |  |  |  |  |  |  |  | 52.44 | 7.4 | 2 |  |  |  |  |  |  |
| Insulin-like growth factor-binding protein complex acid labile subunit | ALS_HUMAN | 66735 | 6.34 | 301.59 | 12.1 | 5 |  |  |  |  |  |  | 203.1 | 8.6 | 4 |  |  |  |  |  |  |
| Integrator complex subunit 1 | INT1_HUMAN | 246366 | 5.77 |  |  |  |  |  |  |  |  |  | 75.51 | 1.1 | 3 |  |  |  |  |  |  |
| Integrator complex subunit 3* | INT3_HUMAN | 119533 | 5.54 |  |  |  | 66.53 | 1.2 | 2 |  |  |  |  |  |  |  |  |  |  |  |  |
| Integrator complex subunit 6* | INT6_HUMAN | 100954 | 8.79 |  |  |  |  |  |  |  |  |  | 53.15 | 2.3 | 2 |  |  |  |  |  |  |
| Integrator complex subunit 7 | INT7_HUMAN | 108021 | 8.31 |  |  |  | 72.35 | 4.1 | 3 | 54.68 | 3.2 | 2 | 104.45 | 4.8 | 4 | 74.27 | 4.1 | 3 |  |  |  |
| Inter-alpha-trypsin inhibitor heavy chain H1 | ITIH1_HUMAN | 101782 | 6.32 | 1057.43 | 24 | 16 | 750.73 | 17.2 | 13 | 687.02 | 18.2 | 12 | 419.18 | 10.9 | 8 |  |  |  |  |  |  |
| Inter-alpha-trypsin inhibitor heavy chain H2 | ITIH2_HUMAN | 106853 | 6.40 | 1258.31 | 33.8 | 25 | 986.73 | 25.6 | 19 | 485.17 | 13.1 | 11 | 785.59 | 18.2 | 16 | 214.84 | 6.1 | 4 |  |  |  |
| Inter-alpha-trypsin inhibitor heavy chain H3 | ITIH3_HUMAN | 100072 | 5.49 |  |  |  |  |  |  |  |  |  | 89.18 | 2 | 2 |  |  |  |  |  |  |
| Inter-alpha-trypsin inhibitor heavy chain H4 | ITIH4_HUMAN | 103521 | 6.51 | 1367.58 | 36.9 | 25 | 814.42 | 18.6 | 16 | 404.09 | 9.4 | 9 | 1772.79 | 44.3 | 32 |  |  |  | 49.45 | 2.2 | 2 |
| Inter-alpha-trypsin inhibitor heavy chain H5 | ITIH5_HUMAN | 105025 | 8.47 |  |  |  |  |  |  |  |  |  | 131.49 | 3.7 | 4 |  |  |  |  |  |  |
| Interleukin-6 receptor subunit beta | IL6RB_HUMAN | 104498 | 5.63 |  |  |  | 60.09 | 2.9 | 2 | 79.87 | 3.6 | 3 | 179.91 | 5.2 | 5 |  |  |  |  |  |  |
| Intersectin-1 | ITSN1_HUMAN | 196155 | 7.77 |  |  |  |  |  |  |  |  |  | 91.59 | 1.9 | 4 |  |  |  |  |  |  |
| Intersectin-2 | ITSN2_HUMAN | 194423 | 8.33 |  |  |  |  |  |  |  |  |  | 91.34 | 1.9 | 4 |  |  |  |  |  |  |
| Intraflagellar transport protein 88 homolog* | IFT88_HUMAN | 94781 | 6.21 | 47.05 | 1.6 | 2 |  |  |  |  |  |  |  |  |  |  |  |  |  |  |  |
| Intron-binding protein aquarius | AQR_HUMAN | 172270 | 5.96 |  |  |  |  |  |  |  |  |  | 55.01 | 1.3 | 2 |  |  |  |  |  |  |
| Inversin* | INVS_HUMAN | 118837 | 9.43 |  |  |  |  |  |  |  |  |  | 56.88 | 2.3 | 2 |  |  |  |  |  |  |
| IQ domain-containing protein E | IQCE_HUMAN | 77649 | 9.16 |  |  |  | 52.14 | 2.2 | 2 | 57.23 | 2.2 | 2 | 59.02 | 2.2 | 2 |  |  |  |  |  |  |
| Isoamyl acetate-hydrolyzing esterase 1 homolog | IAH1_HUMAN | 28037 | 5.13 | 106.35 | 13.3 | 3 |  |  |  |  |  |  |  |  |  |  |  |  |  |  |  |
| Isochorismatase domain-containing protein 1 | ISOC1_HUMAN | 32501 | 6.96 |  |  |  |  |  |  |  |  |  | 79.98 | 13.4 | 3 |  |  |  |  |  |  |
| Isocitrate dehydrogenase [NADP] cytoplasmic | IDHC_HUMAN | 46915 | 6.53 | 71.92 | 4.1 | 2 |  |  |  |  |  |  | 124.11 | 6.3 | 2 |  |  |  |  |  |  |
| Isoleucine--tRNA ligase. cytoplasmic | SYIC_HUMAN | 145718 | 5.82 | 55.48 | 1 | 2 | 57.03 | 1 | 2 | 54.45 | 1 | 2 |  |  |  |  |  |  |  |  |  |
| Janus kinase and microtubule-interacting protein 1 | JKIP1_HUMAN | 73506 | 5.84 |  |  |  |  |  |  |  |  |  | 92.46 | 5 | 4 |  |  |  |  |  |  |
| JmjC domain-containing protein 7 | JMJD7_HUMAN | 36308 | 5.23 | 285.14 | 25 | 6 | 143.57 | 10.8 | 3 |  |  |  | 442.26 | 30.1 | 9 | 216.49 | 25 | 4 |  |  |  |
| Junction plakoglobin | PLAK_HUMAN | 82434 | 5.75 | 244.33 | 11.1 | 7 | 557.33 | 18.7 | 11 | 610.78 | 22.1 | 14 | 1100.05 | 39.2 | 25 | 612.18 | 29.7 | 15 |  |  |  |
| Kallistatin | KAIN_HUMAN | 48682 | 7.34 | 94.26 | 9.1 | 3 | 114.86 | 8 | 3 | 88.99 | 6.6 | 2 | 264.09 | 19.2 | 8 |  |  |  |  |  |  |
| Kelch-like protein 9 | KLHL9_HUMAN | 70239 | 5.92 | 51.79 | 2.3 | 2 |  |  |  |  |  |  |  |  |  |  |  |  |  |  |  |
| Keratin. type I cuticular Ha1 | K1H1_HUMAN | 48633 | 4.84 |  |  |  |  |  |  |  |  |  | 1564.45 | 64.7 | 27 | 590.7 | 26.4 | 11 |  |  |  |
| Keratin. type I cuticular Ha2 | K1H2_HUMAN | 51793 | 4.78 |  |  |  |  |  |  |  |  |  | 475.91 | 17.2 | 9 |  |  |  |  |  |  |
| Keratin. type I cuticular Ha3-I | KT33A_HUMAN | 47166 | 4.78 |  |  |  |  |  |  |  |  |  | 1218.79 | 46.5 | 21 | 450.06 | 19.6 | 8 |  |  |  |
| Keratin. type I cuticular Ha3-II | KT33B_HUMAN | 47325 | 4.81 |  |  |  |  |  |  |  |  |  | 1421.22 | 61.9 | 23 | 426.46 | 16.8 | 8 |  |  |  |
| Keratin. type I cuticular Ha4 | KRT34_HUMAN | 50818 | 5.01 |  |  |  |  |  |  |  |  |  | 1111.96 | 44.3 | 20 |  |  |  |  |  |  |
| Keratin. type I cuticular Ha5 | KRT35_HUMAN | 51640 | 4.85 |  |  |  |  |  |  |  |  |  | 588.11 | 18.9 | 12 |  |  |  |  |  |  |
| Keratin. type I cuticular Ha6 | KRT36_HUMAN | 53354 | 4.90 |  |  |  |  |  |  |  |  |  | 646.15 | 28.7 | 14 | 193.7 | 7.1 | 4 |  |  |  |
| Keratin. type I cytoskeletal 10 | K1C10_HUMAN | 59020 | 5.13 | 2980.03 | 63.2 | 39 | 3307.44 | 62 | 41 | 3424.76 | 67.1 | 44 | 4118.15 | 69.7 | 50 | 3122.75 | 65.9 | 39 | 269.92 | 6.8 | 3 |
| Keratin. type I cytoskeletal 12 | K1C12_HUMAN | 53592 | 4.70 | 121.33 | 6.5 | 3 |  |  |  |  |  |  |  |  |  |  |  |  |  |  |  |
| Keratin. type I cytoskeletal 13 | K1C13_HUMAN | 49900 | 4.91 |  |  |  | 1186.06 | 37.6 | 23 | 925.66 | 30.3 | 18 | 1642.51 | 53.5 | 31 | 583.74 | 28.4 | 13 |  |  |  |
| Keratin. type I cytoskeletal 14 | K1C14_HUMAN | 51872 | 5.09 | 1306.41 | 46.6 | 25 | 2021.35 | 72 | 37 | 2333.88 | 78.8 | 39 | 3006.94 | 79.9 | 49 | 1817.92 | 69.5 | 32 |  |  |  |
| Keratin. type I cytoskeletal 15 | K1C15_HUMAN | 49409 | 4.72 | 458.7 | 15.6 | 10 | 892.46 | 23.5 | 18 |  |  |  | 1135.53 | 31.1 | 21 |  |  |  |  |  |  |
| Keratin. type I cytoskeletal 16 | K1C16_HUMAN | 51578 | 4.99 | 1349.64 | 58.4 | 27 | 2173.89 | 76.7 | 39 | 2344.38 | 76.7 | 40 | 2793.49 | 72.9 | 44 | 1530.58 | 64.5 | 30 |  |  |  |
| Keratin. type I cytoskeletal 17 | K1C17_HUMAN | 48361 | 4.98 |  |  |  | 1243.32 | 40.3 | 23 | 1281.31 | 47.7 | 26 | 1596.96 | 51.4 | 31 | 887.41 | 35 | 17 |  |  |  |
| Keratin. type I cytoskeletal 18 | K1C18_HUMAN | 48029 | 5.34 |  |  |  |  |  |  | 102.79 | 5.8 | 3 | 121.31 | 5.8 | 3 |  |  |  |  |  |  |
| Keratin. type I cytoskeletal 19 | K1C19_HUMAN | 44079 | 5.04 |  |  |  |  |  |  |  |  |  | 905.34 | 33.8 | 18 |  |  |  |  |  |  |
| Keratin. type I cytoskeletal 20 | K1C20_HUMAN | 48514 | 5.52 |  |  |  | 204.4 | 5.7 | 4 |  |  |  | 267.89 | 9.9 | 6 |  |  |  |  |  |  |
| Keratin. type I cytoskeletal 25 | K1C25_HUMAN | 49858 | 5.00 |  |  |  |  |  |  |  |  |  | 203.35 | 8.2 | 4 |  |  |  |  |  |  |
| Keratin. type I cytoskeletal 28 | K1C28_HUMAN | 51163 | 5.33 |  |  |  |  |  |  |  |  |  | 360.75 | 8.4 | 6 |  |  |  |  |  |  |
| Keratin. type I cytoskeletal 9 | K1C9_HUMAN | 62255 | 5.14 | 2506.43 | 59.9 | 35 | 3101.9 | 66.5 | 39 | 3158.37 | 70.5 | 41 | 3730.12 | 90.4 | 47 | 2812 | 61.5 | 38 | 225.73 | 9.1 | 4 |
| Keratin. type II cuticular Hb1 | KRT81_HUMAN | 56832 | 5.40 | 128.59 | 4.8 | 3 |  |  |  |  |  |  |  |  |  |  |  |  |  |  |  |
| Keratin. type II cuticular Hb2 | KRT82_HUMAN | 57985 | 6.40 | 74.5 | 3.3 | 2 | 162.25 | 3.9 | 3 |  |  |  | 419.78 | 15 | 8 | 336.54 | 9.4 | 6 |  |  |  |
| Keratin. type II cuticular Hb3 | KRT83_HUMAN | 55928 | 5.55 |  |  |  |  |  |  |  |  |  | 1676.92 | 51.3 | 33 | 865.49 | 29.2 | 18 |  |  |  |
| Keratin. type II cuticular Hb4 | KRT84_HUMAN | 65942 | 7.75 |  |  |  |  |  |  |  |  |  | 1014.49 | 37.7 | 22 |  |  |  |  |  |  |
| Keratin. type II cuticular Hb5 | KRT85_HUMAN | 57306 | 6.28 |  |  |  | 136.13 | 7.5 | 4 |  |  |  | 1814.53 | 64.7 | 38 | 711.17 | 26.4 | 15 |  |  |  |
| Keratin. type II cuticular Hb6 | KRT86_HUMAN | 55120 | 5.57 |  |  |  |  |  |  |  |  |  | 2112.03 | 70.6 | 38 | 881.09 | 31.7 | 18 |  |  |  |
| Keratin. type II cytoskeletal 1 | K2C1_HUMAN | 66170 | 8.15 | 2886.49 | 62 | 43 | 3984.24 | 76.7 | 58 | 4150.01 | 77.3 | 58 | 4690.39 | 78.1 | 62 | 3440.74 | 70.7 | 48 | 541.62 | 17.2 | 9 |
| Keratin. type II cytoskeletal 1b | K2C1B_HUMAN | 62149 | 5.73 |  |  |  | 481.4 | 10.2 | 8 | 814.42 | 22.7 | 15 | 1059.87 | 30.3 | 20 | 408.99 | 11.6 | 7 |  |  |  |
| Keratin. type II cytoskeletal 2 epidermal | K22E_HUMAN | 65678 | 8.08 | 3103.22 | 74 | 43 | 4372.91 | 87.3 | 61 | 4510.86 | 90.8 | 63 | 5400.39 | 94.7 | 69 | 3372.34 | 81.7 | 45 | 329.13 | 12.2 | 6 |
| Keratin. type II cytoskeletal 2 oral | K22O_HUMAN | 66370 | 8.38 |  |  |  |  |  |  |  |  |  | 1258.49 | 27.6 | 25 |  |  |  |  |  |  |
| Keratin. type II cytoskeletal 3 | K2C3_HUMAN | 64549 | 6.12 |  |  |  | 895.75 | 17.8 | 19 | 816.03 | 17 | 16 | 1694.91 | 41.6 | 32 |  |  |  |  |  |  |
| Keratin. type II cytoskeletal 4 | K2C4_HUMAN | 57649 | 6.25 |  |  |  | 1242.43 | 40.1 | 23 | 805.08 | 32.2 | 14 | 1619.07 | 56.7 | 28 |  |  |  |  |  |  |
| Keratin. type II cytoskeletal 5 | K2C5_HUMAN | 62568 | 7.59 | 1670.94 | 43.4 | 30 | 2464.55 | 53.7 | 45 | 2930.05 | 56.1 | 51 | 3477.36 | 65.1 | 57 | 1797.73 | 46.3 | 32 | 151.21 | 5.1 | 3 |
| Keratin. type II cytoskeletal 6A | K2C6A_HUMAN | 60293 | 8.10 | 1503.74 | 40.1 | 27 | 2428.45 | 57.4 | 42 | 2628.81 | 57.4 | 45 | 3149.22 | 60.5 | 50 | 1637.99 | 47.3 | 28 |  |  |  |
| Keratin. type II cytoskeletal 6B | K2C6B_HUMAN | 60315 | 8.10 | 1338.35 | 35.8 | 24 | 2372.64 | 55.9 | 41 | 2476.21 | 58.3 | 43 | 2997.36 | 60.5 | 48 | 1481.33 | 44 | 26 |  |  |  |
| Keratin. type II cytoskeletal 6C | K2C6C_HUMAN | 60273 | 8.09 |  |  |  | 2411.31 | 57.4 | 42 | 2658.98 | 58.3 | 46 | 3111.67 | 60.5 | 49 |  |  |  |  |  |  |
| Keratin. type II cytoskeletal 7 | K2C7_HUMAN | 51411 | 5.40 |  |  |  |  |  |  | 447.59 | 14.5 | 10 | 711.32 | 22.4 | 15 |  |  |  |  |  |  |
| Keratin. type II cytoskeletal 71 | K2C71_HUMAN | 57769 | 6.28 |  |  |  |  |  |  |  |  |  | 541.4 | 15.5 | 10 |  |  |  |  |  |  |
| Keratin. type II cytoskeletal 72 | K2C72_HUMAN | 56470 | 6.54 |  |  |  |  |  |  |  |  |  | 498.62 | 15.1 | 10 |  |  |  |  |  |  |
| Keratin. type II cytoskeletal 73 | K2C73_HUMAN | 59457 | 6.94 |  |  |  |  |  |  |  |  |  |  |  |  | 325.14 | 7.8 | 6 |  |  |  |
| Keratin. type II cytoskeletal 78 | K2C78_HUMAN | 57629 | 5.79 | 226.8 | 5.6 | 4 | 400.87 | 16 | 9 | 460.24 | 16.9 | 9 | 821.13 | 25.4 | 15 | 255.53 | 7.5 | 4 |  |  |  |
| Keratin. type II cytoskeletal 79 | K2C79_HUMAN | 58085 | 6.75 |  |  |  | 868.78 | 21.7 | 18 |  |  |  |  |  |  |  |  |  |  |  |  |
| Keratin. type II cytoskeletal 8 | K2C8_HUMAN | 53671 | 5.52 | 312.64 | 10.8 | 8 |  |  |  |  |  |  |  |  |  |  |  |  |  |  |  |
| Keratin. type II cytoskeletal 80 | K2C80_HUMAN | 51007 | 5.59 | 125.54 | 7.1 | 3 | 199.04 | 9.3 | 4 | 230.06 | 11.7 | 5 | 483.44 | 23 | 10 |  |  |  |  |  |  |
| Keratin-81-like protein | KT81L_HUMAN | 54972 | 6.06 |  |  |  |  |  |  |  |  |  | 1380.83 | 42.6 | 27 | 706.84 | 26.1 | 15 |  |  |  |
| Keratin-associated protein 13-1 | KR131_HUMAN | 19505 | 8.54 |  |  |  |  |  |  |  |  |  | 99.82 | 11.6 | 2 |  |  |  |  |  |  |
| Keratin-associated protein 13-2 | KR132_HUMAN | 19912 | 8.72 |  |  |  |  |  |  |  |  |  | 100.49 | 11.4 | 2 |  |  |  |  |  |  |
| Keratin-associated protein 3-1 | KRA31_HUMAN | 11558 | 6.00 |  |  |  |  |  |  |  |  |  | 88.95 | 24.5 | 2 |  |  |  |  |  |  |
| Keratinocyte proline-rich protein | KPRP_HUMAN | 67172 | 8.72 | 105.93 | 5 | 3 | 62.65 | 2.6 | 2 | 226.62 | 9.8 | 6 | 453.73 | 21.8 | 11 | 252.97 | 19 | 7 |  |  |  |
| Kinesin heavy chain isoform 5C | KIF5C_HUMAN | 109997 | 5.87 |  |  |  | 64.3 | 2.2 | 3 |  |  |  | 57.46 | 1.4 | 2 |  |  |  |  |  |  |
| Kinesin-1 heavy chain* | KINH_HUMAN | 110358 | 6.12 | 54.58 | 1.7 | 2 |  |  |  |  |  |  |  |  |  |  |  |  |  |  |  |
| Kinesin-like protein KIF1B* | KIF1B_HUMAN | 205774 | 5.42 |  |  |  |  |  |  |  |  |  | 53.23 | 0.8 | 2 |  |  |  |  |  |  |
| Kininogen-1 | KNG1_HUMAN | 72996 | 6.34 | 778.34 | 33.7 | 16 | 449.36 | 16 | 10 | 477.53 | 17.1 | 9 | 715.95 | 20.8 | 12 | 437.26 | 16.8 | 8 | 262.39 | 10.9 | 6 |
| Kynurenine--oxoglutarate transaminase 1* | KAT1_HUMAN | 48186 | 6.03 |  |  |  | 65.04 | 5.9 | 2 |  |  |  |  |  |  |  |  |  |  |  |  |
| Lactase-like protein | LCTL_HUMAN | 65560 | 8.17 |  |  |  | 86.87 | 4.4 | 3 |  |  |  | 229.38 | 10.8 | 7 | 225.53 | 6.9 | 4 |  |  |  |
| Lactotransferrin | TRFL_HUMAN | 80014 | 8.51 |  |  |  |  |  |  |  |  |  | 2171.15 | 61.4 | 35 |  |  |  |  |  |  |
| Lactoylglutathione lyase | LGUL_HUMAN | 20992 | 5.13 |  |  |  |  |  |  |  |  |  | 288.5 | 33.2 | 6 |  |  |  |  |  |  |
| Lambda-crystallin homolog | CRYL1_HUMAN | 35909 | 5.81 | 578.89 | 49.8 | 12 | 351.9 | 22.6 | 7 |  |  |  | 494.46 | 30.4 | 8 |  |  |  |  |  |  |
| Laminin subunit alpha-1 | LAMA1_HUMAN | 346332 | 5.93 |  |  |  |  |  |  |  |  |  | 75.15 | 0.8 | 3 |  |  |  |  |  |  |
| Laminin subunit alpha-2 | LAMA2_HUMAN | 352978 | 6.01 | 95.27 | 0.9 | 4 |  |  |  |  |  |  |  |  |  |  |  |  |  |  |  |
| Laminin subunit alpha-5* | LAMA5_HUMAN | 412023 | 6.66 |  |  |  | 60.51 | 0.5 | 2 | 58.36 | 0.4 | 2 | 68.68 | 0.5 | 2 |  |  |  |  |  |  |
| Laminin subunit beta-3* | LAMB3_HUMAN | 133366 | 7.15 |  |  |  |  |  |  |  |  |  | 50.9 | 2.5 | 2 |  |  |  |  |  |  |
| Laminin subunit gamma-1* | LAMC1_HUMAN | 183191 | 5.01 |  |  |  |  |  |  |  |  |  | 50.66 | 1.3 | 2 |  |  |  |  |  |  |
| Latent-transforming growth factor beta-binding protein 2 | LTBP2_HUMAN | 204046 | 5.07 | 315.14 | 4.6 | 6 |  |  |  |  |  |  |  |  |  |  |  |  |  |  |  |
| Lebercilin-like protein* | LCA5L_HUMAN | 76857 | 9.51 |  |  |  |  |  |  |  |  |  | 50.44 | 1.9 | 2 |  |  |  |  |  |  |
| Lengsin | LGSN_HUMAN | 57983 | 5.97 | 540.7 | 22.6 | 9 | 183.63 | 7.1 | 3 | 142.44 | 5.7 | 2 | 341.23 | 14.1 | 7 | 195.42 | 6.9 | 3 | 297.4 | 11 | 5 |
| Lens fiber major intrinsic protein | MIP_HUMAN | 28218 | 8.64 | 214.39 | 27 | 5 | 376.5 | 39.9 | 6 | 134.26 | 13.7 | 3 | 199.81 | 28.1 | 5 | 192.49 | 36.9 | 4 | 359.03 | 67.3 | 8 |
| Lens fiber membrane intrinsic protein | LMIP_HUMAN | 20003 | 9.66 | 204.94 | 22.5 | 4 | 188.53 | 19.1 | 3 |  |  |  |  |  |  |  |  |  | 59.97 | 10.4 | 2 |
| Leucine zipper protein 2 | LUZP2_HUMAN | 39275 | 8.90 |  |  |  |  |  |  |  |  |  | 62.68 | 3.5 | 2 |  |  |  |  |  |  |
| Leucine-rich alpha-2-glycoprotein | A2GL_HUMAN | 38382 | 6.45 | 345.55 | 30 | 9 | 181.97 | 7.5 | 3 | 236.69 | 22.2 | 6 | 581.16 | 40.3 | 11 | 491.67 | 33.1 | 8 |  |  |  |
| Leucine-rich repeat and coiled-coil domain-containing protein 1* | LRCC1_HUMAN | 120719 | 5.62 |  |  |  |  |  |  |  |  |  | 51.95 | 1.6 | 2 |  |  |  |  |  |  |
| Leucine-rich repeat and fibronectin type-III domain-containing protein 2* | LRFN2_HUMAN | 85532 | 6.28 |  |  |  |  |  |  |  |  |  | 47.61 | 1.8 | 2 |  |  |  |  |  |  |
| Leucine-rich repeat and guanylate kinase domain-containing protein* | LRGUK_HUMAN | 94415 | 6.00 |  |  |  |  |  |  | 60.54 | 1.8 | 2 |  |  |  |  |  |  |  |  |  |
| Leucine-rich repeat and IQ domain-containing protein 3* | LRIQ3_HUMAN | 74256 | 9.73 | 50.9 | 2.1 | 2 |  |  |  |  |  |  |  |  |  |  |  |  |  |  |  |
| Leucine-rich repeat serine/threonine-protein kinase 2 | LRRK2_HUMAN | 289568 | 6.36 |  |  |  |  |  |  |  |  |  | 98.19 | 1.4 | 4 |  |  |  |  |  |  |
| Leucine-rich repeat transmembrane protein FLRT3 | FLRT3_HUMAN | 73642 | 7.56 |  |  |  | 71.74 | 4.9 | 3 |  |  |  |  |  |  |  |  |  |  |  |  |
| Leucine-rich repeat-containing protein 15 | LRC15_HUMAN | 65238 | 6.24 |  |  |  |  |  |  |  |  |  | 81.48 | 5.9 | 3 |  |  |  |  |  |  |
| Leucine-rich repeat-containing protein 8C* | LRC8C_HUMAN | 93359 | 7.54 | 63.89 | 1.1 | 2 |  |  |  |  |  |  |  |  |  |  |  |  |  |  |  |
| Leukocyte elastase inhibitor | ILEU_HUMAN | 42829 | 5.90 | 318.4 | 17.4 | 6 | 179.44 | 11.9 | 4 | 254.71 | 20.6 | 7 | 514.83 | 25.9 | 9 | 59.47 | 5 | 2 |  |  |  |
| LIM and cysteine-rich domains protein 1 | LMCD1_HUMAN | 42004 | 8.28 | 77.6 | 8.8 | 2 |  |  |  |  |  |  |  |  |  |  |  |  |  |  |  |
| Limbic system-associated membrane protein | LSAMP_HUMAN | 37883 | 6.55 |  |  |  |  |  |  |  |  |  | 374.01 | 22.5 | 7 | 301.55 | 19.5 | 6 |  |  |  |
| Lipocalin-1 | LCN1_HUMAN | 19409 | 5.40 | 72.3 | 12.5 | 2 |  |  |  |  |  |  | 81.36 | 12.5 | 2 |  |  |  |  |  |  |
| Lipopolysaccharide-binding protein | LBP_HUMAN | 53521 | 6.24 |  |  |  |  |  |  |  |  |  | 84.06 | 6 | 2 |  |  |  |  |  |  |
| Liver carboxylesterase 1 | EST1_HUMAN | 62766 | 6.15 | 240.89 | 12.7 | 5 |  |  |  | 164.3 | 7.2 | 3 | 747.07 | 32.1 | 14 |  |  |  |  |  |  |
| L-lactate dehydrogenase A chain | LDHA_HUMAN | 36950 | 8.45 | 1216 | 63.3 | 21 | 944.94 | 52.7 | 18 | 882.09 | 56 | 17 | 1009.48 | 50.9 | 17 | 575.27 | 30.7 | 9 | 686.87 | 44.9 | 15 |
| L-lactate dehydrogenase B chain | LDHB_HUMAN | 36900 | 5.71 | 245.21 | 23.4 | 7 | 299.39 | 15.6 | 5 | 360.91 | 20.7 | 6 | 996.48 | 48.8 | 17 | 155.92 | 10.2 | 3 | 137.83 | 9.9 | 3 |
| L-lactate dehydrogenase C chain* | LDHC_HUMAN | 36630 | 7.09 | 89.67 | 6 | 2 |  |  |  |  |  |  | 103.72 | 6 | 2 |  |  |  |  |  |  |
| Long-chain-fatty-acid--CoA ligase 6* | ACSL6_HUMAN | 78728 | 7.21 |  |  |  |  |  |  |  |  |  | 65.66 | 1.1 | 2 |  |  |  |  |  |  |
| Low-density lipoprotein receptor-related protein 2 | LRP2_HUMAN | 540376 | 4.89 | 582.55 | 2.7 | 13 | 545.96 | 2.5 | 11 | 273.57 | 1.5 | 7 | 51.38 | 0.4 | 2 |  |  |  |  |  |  |
| Lumican | LUM_HUMAN | 38747 | 6.17 |  |  |  | 122.29 | 7.7 | 2 |  |  |  | 452.59 | 32 | 9 | 360.59 | 27.5 | 7 |  |  |  |
| Lysine-specific demethylase 5B | KDM5B_HUMAN | 178681 | 6.29 | 67.02 | 1.7 | 3 |  |  |  |  |  |  |  |  |  |  |  |  |  |  |  |
| Lysine-specific demethylase 5D | KDM5D_HUMAN | 176756 | 5.59 |  |  |  |  |  |  | 78.24 | 1 | 3 | 140.33 | 2.2 | 5 |  |  |  |  |  |  |
| Lysosomal-trafficking regulator* | LYST_HUMAN | 434169 | 6.15 |  |  |  |  |  |  |  |  |  | 49.97 | 0.5 | 2 |  |  |  |  |  |  |
| Lysosome-associated membrane glycoprotein 2* | LAMP2_HUMAN | 45503 | 5.35 |  |  |  |  |  |  |  |  |  | 56.65 | 4.9 | 2 |  |  |  |  |  |  |
| Lysozyme C | LYSC_HUMAN | 16982 | 9.39 | 293.4 | 45.9 | 5 | 179.76 | 33.1 | 3 | 166.18 | 23 | 4 | 727.05 | 73 | 15 | 114.86 | 14.2 | 2 |  |  |  |
| Macrophage colony-stimulating factor 1 receptor | CSF1R_HUMAN | 109113 | 5.94 |  |  |  | 75.34 | 2.4 | 2 |  |  |  | 130.66 | 4.1 | 4 |  |  |  |  |  |  |
| Macrophage migration inhibitory factor | MIF_HUMAN | 12639 | 7.74 |  |  |  | 165.54 | 23.5 | 3 | 174.04 | 23.5 | 3 |  |  |  |  |  |  | 138.43 | 17.4 | 2 |
| Major vault protein | MVP_HUMAN | 99551 | 5.34 | 591.99 | 18 | 13 | 245.17 | 8.4 | 8 |  |  |  | 1721.74 | 55.5 | 38 |  |  |  | 67.23 | 3.6 | 2 |
| Malate dehydrogenase. cytoplasmic | MDHC_HUMAN | 36631 | 6.92 | 895.09 | 55.4 | 17 | 630.33 | 46.7 | 12 | 380.52 | 22.8 | 6 | 686.02 | 53 | 14 |  |  |  | 284.76 | 17.7 | 5 |
| Malate dehydrogenase. mitochondrial | MDHM_HUMAN | 35937 | 8.92 |  |  |  |  |  |  |  |  |  | 322.92 | 25.7 | 7 |  |  |  |  |  |  |
| MAP/microtubule affinity-regulating kinase 3 | MARK3_HUMAN | 84949 | 9.60 |  |  |  |  |  |  |  |  |  | 70.29 | 4 | 3 |  |  |  |  |  |  |
| Mast cell carboxypeptidase A* | CBPA3_HUMAN | 48924 | 9.09 |  |  |  |  |  |  |  |  |  | 51.34 | 3.4 | 2 |  |  |  |  |  |  |
| Mediator of RNA polymerase II transcription subunit 16* | MED16_HUMAN | 98441 | 7.12 |  |  |  |  |  |  |  |  |  | 52.1 | 2.3 | 2 |  |  |  |  |  |  |
| Metalloproteinase inhibitor 1 | TIMP1_HUMAN | 23840 | 8.46 |  |  |  |  |  |  |  |  |  | 87.39 | 10.1 | 2 |  |  |  |  |  |  |
| Metalloproteinase inhibitor 2* | TIMP2_HUMAN | 25067 | 7.45 |  |  |  |  |  |  |  |  |  | 52.13 | 5.9 | 2 |  |  |  |  |  |  |
| Metastasis-associated protein MTA2 | MTA2_HUMAN | 75717 | 9.71 |  |  |  |  |  |  |  |  |  | 52.84 | 2.4 | 2 |  |  |  |  |  |  |
| Methylosome protein 50 | MEP50_HUMAN | 37442 | 5.03 |  |  |  |  |  |  |  |  |  | 170.86 | 10.5 | 3 |  |  |  |  |  |  |
| Microfibril-associated glycoprotein 4 | MFAP4_HUMAN | 28972 | 5.39 |  |  |  |  |  |  |  |  |  | 148.31 | 14.5 | 4 | 98.8 | 9.4 | 2 |  |  |  |
| Microtubule-actin cross-linking factor 1. isoforms 1/2/3/5 | MACF1_HUMAN | 843033 | 5.29 | 64.3 | 0.3 | 3 |  |  |  |  |  |  | 82.01 | 0.4 | 3 |  |  |  |  |  |  |
| Microtubule-associated protein 1A | MAP1A_HUMAN | 306781 | 4.86 | 68.89 | 0.9 | 3 |  |  |  |  |  |  |  |  |  |  |  |  |  |  |  |
| Microtubule-associated protein 1B | MAP1B_HUMAN | 271665 | 4.73 |  |  |  |  |  |  |  |  |  | 72.54 | 0.9 | 3 |  |  |  |  |  |  |
| Microtubule-associated protein RP/EB family member 1 | MARE1_HUMAN | 30151 | 5.03 |  |  |  |  |  |  |  |  |  | 77.28 | 17.9 | 3 |  |  |  |  |  |  |
| Microtubule-associated tumor suppressor candidate 2 | MTUS2_HUMAN | 150901 | 6.23 | 60.46 | 1.1 | 2 |  |  |  | 84.57 | 2.4 | 3 | 83.61 | 2 | 3 |  |  |  |  |  |  |
| Midasin | MDN1_HUMAN | 638008 | 5.46 |  |  |  |  |  |  |  |  |  | 91.29 | 0.6 | 3 |  |  |  |  |  |  |
| Mis18-binding protein 1 | M18BP_HUMAN | 129918 | 9.31 |  |  |  |  |  |  |  |  |  | 89.38 | 2.7 | 4 |  |  |  |  |  |  |
| Mitogen-activated protein kinase 3 | MK03_HUMAN | 43450 | 6.29 |  |  |  |  |  |  |  |  |  | 80.96 | 6.3 | 3 |  |  |  |  |  |  |
| Moesin | MOES_HUMAN | 67892 | 6.08 | 427.63 | 20.5 | 10 | 197.02 | 7.6 | 5 |  |  |  | 189.75 | 9.5 | 5 |  |  |  | 117.12 | 5 | 3 |
| Molybdopterin synthase sulfur carrier subunit | MOC2A_HUMAN | 9806 | 4.67 |  |  |  |  |  |  |  |  |  | 85.34 | 28.4 | 3 |  |  |  |  |  |  |
| Monocyte differentiation antigen CD14 | CD14_HUMAN | 40678 | 5.85 | 117.23 | 9.9 | 3 | 137.23 | 9.3 | 3 | 124.02 | 13.1 | 3 | 380.88 | 22.7 | 6 | 128.48 | 10.4 | 2 |  |  |  |
| Monoglyceride lipase | MGLL_HUMAN | 33468 | 6.49 |  |  |  |  |  |  |  |  |  | 67.93 | 15.2 | 3 |  |  |  |  |  |  |
| Mortality factor 4-like protein 1 | MO4L1_HUMAN | 41562 | 9.29 |  |  |  |  |  |  |  |  |  | 80.59 | 7.2 | 3 |  |  |  |  |  |  |
| Mucin-5B | MUC5B_HUMAN | 611584 | 6.20 |  |  |  |  |  |  |  |  |  | 963.87 | 3.7 | 18 |  |  |  |  |  |  |
| Mucin-7* | MUC7_HUMAN | 39420 | 8.99 |  |  |  |  |  |  |  |  |  | 53.29 | 5 | 2 |  |  |  |  |  |  |
| Multidrug resistance protein 3 | MDR3_HUMAN | 142004 | 8.66 |  |  |  |  |  |  | 63.75 | 2.3 | 3 | 64.86 | 1.8 | 3 |  |  |  |  |  |  |
| Multifunctional protein ADE2 | PUR6_HUMAN | 47790 | 6.95 |  |  |  |  |  |  |  |  |  | 185.91 | 11.5 | 5 | 64.63 | 3.1 | 2 |  |  |  |
| Multiple C2 and transmembrane domain-containing protein 2 | MCTP2_HUMAN | 100388 | 7.48 |  |  |  | 51.23 | 2.1 | 2 | 55.15 | 2.1 | 2 | 58.82 | 2.1 | 2 |  |  |  |  |  |  |
| Multiple epidermal growth factor-like domains protein 8 | MEGF8_HUMAN | 314021 | 6.46 | 140.12 | 1.5 | 3 | 166.49 | 1.3 | 3 | 209.87 | 2.1 | 4 |  |  |  |  |  |  |  |  |  |
| Myeloperoxidase | PERM_HUMAN | 84784 | 9.19 |  |  |  |  |  |  |  |  |  | 314.53 | 11 | 7 |  |  |  |  |  |  |
| Myocilin | MYOC_HUMAN | 57450 | 5.45 | 77.02 | 4 | 2 |  |  |  | 70.07 | 4 | 2 | 163.27 | 7.1 | 4 |  |  |  |  |  |  |
| Myomegalin | MYOME_HUMAN | 266343 | 5.32 |  |  |  |  |  |  |  |  |  | 76.75 | 0.8 | 3 |  |  |  |  |  |  |
| Myosin regulatory light chain 2. skeletal muscle isoform | MLRS_HUMAN | 19116 | 4.91 |  |  |  |  |  |  |  |  |  | 128.22 | 13.6 | 2 |  |  |  |  |  |  |
| Myosin-1 | MYH1_HUMAN | 223976 | 5.60 |  |  |  |  |  |  |  |  |  | 1425.14 | 17.3 | 30 |  |  |  |  |  |  |
| Myosin-10 | MYH10_HUMAN | 229827 | 5.45 | 100.61 | 1.5 | 3 | 70.59 | 1.4 | 3 |  |  |  | 67.23 | 1.3 | 3 |  |  |  |  |  |  |
| Myosin-14 | MYH14_HUMAN | 228701 | 5.53 | 118.59 | 1.8 | 3 |  |  |  |  |  |  | 51.54 | 0.7 | 2 |  |  |  |  |  |  |
| Myosin-15 | MYH15_HUMAN | 225904 | 5.67 |  |  |  |  |  |  |  |  |  | 160.53 | 2.5 | 6 |  |  |  |  |  |  |
| Myosin-2 | MYH2_HUMAN | 223932 | 5.65 |  |  |  |  |  |  |  |  |  | 1283.36 | 15.1 | 27 |  |  |  |  |  |  |
| Myosin-3 | MYH3_HUMAN | 224850 | 5.62 |  |  |  |  |  |  | 101.86 | 1.5 | 4 |  |  |  |  |  |  |  |  |  |
| Myosin-7 | MYH7_HUMAN | 223757 | 5.63 | 75.94 | 1 | 3 |  |  |  |  |  |  |  |  |  |  |  |  |  |  |  |
| Myosin-7B | MYH7B_HUMAN | 222392 | 5.78 |  |  |  |  |  |  | 55.52 | 0.7 | 2 | 117.88 | 2.2 | 5 |  |  |  |  |  |  |
| Myosin-8* | MYH8_HUMAN | 223594 | 5.60 |  |  |  |  |  |  | 59.69 | 0.9 | 2 |  |  |  |  |  |  |  |  |  |
| Myosin-9 | MYH9_HUMAN | 227646 | 5.51 | 445.85 | 5.7 | 9 |  |  |  |  |  |  |  |  |  |  |  |  |  |  |  |
| Myotrophin | MTPN_HUMAN | 13058 | 5.28 | 381.67 | 66.9 | 6 | 440.11 | 66.9 | 7 | 455.48 | 72.9 | 8 | 175.08 | 34.7 | 4 |  |  |  | 172.48 | 32.2 | 2 |
| Myotubularin-related protein 3* | MTMR3_HUMAN | 136157 | 5.52 |  |  |  |  |  |  |  |  |  |  |  |  | 54.63 | 0.7 | 2 |  |  |  |
| N(G).N(G)-dimethylarginine dimethylaminohydrolase 1 | DDAH1_HUMAN | 31444 | 5.53 | 432.68 | 36.5 | 7 | 201.18 | 18.2 | 4 |  |  |  | 580.17 | 47.4 | 12 |  |  |  | 84.24 | 9.1 | 2 |
| N(G).N(G)-dimethylarginine dimethylaminohydrolase 2 | DDAH2_HUMAN | 29911 | 5.66 |  |  |  |  |  |  |  |  |  | 122.09 | 9.1 | 2 |  |  |  |  |  |  |
| N-acetyl-D-glucosamine kinase | NAGK_HUMAN | 37694 | 5.82 |  |  |  |  |  |  |  |  |  | 219.24 | 17.7 | 4 |  |  |  |  |  |  |
| N-acetylglucosamine-6-sulfatase | GNS_HUMAN | 62840 | 8.61 |  |  |  |  |  |  |  |  |  | 321.1 | 21.9 | 8 |  |  |  |  |  |  |
| N-acetyllactosaminide beta-1.3-N-acetylglucosaminyltransferase | B3GN1_HUMAN | 47545 | 6.77 | 160.31 | 17.8 | 5 | 364.2 | 21 | 8 | 384.48 | 24.3 | 7 | 512.3 | 48 | 13 |  |  |  |  |  |  |
| N-acetylmuramoyl-L-alanine amidase | PGRP2_HUMAN | 62748 | 7.26 | 470 | 27.4 | 10 | 146.54 | 9.7 | 3 | 175.75 | 9.7 | 3 | 296.46 | 15.1 | 5 |  |  |  |  |  |  |
| NACHT. LRR and PYD domains-containing protein 6 | NALP6_HUMAN | 100188 | 8.43 |  |  |  |  |  |  |  |  |  | 72.81 | 2.1 | 3 |  |  |  |  |  |  |
| NACHT. LRR and PYD domains-containing protein 7* | NALP7_HUMAN | 113902 | 5.87 |  |  |  | 49.01 | 1.8 | 2 |  |  |  |  |  |  |  |  |  |  |  |  |
| NAD(P)H-hydrate epimerase* | AIBP_HUMAN | 31996 | 7.56 | 52.9 | 12.8 | 2 |  |  |  |  |  |  |  |  |  |  |  |  |  |  |  |
| NADH dehydrogenase [ubiquinone] 1 alpha subcomplex subunit 11 | NDUAB_HUMAN | 15071 | 8.95 |  |  |  | 145.64 | 24.1 | 2 |  |  |  | 136.06 | 24.1 | 2 |  |  |  |  |  |  |
| NADH dehydrogenase [ubiquinone] 1 alpha subcomplex subunit 13 | NDUAD_HUMAN | 16688 | 8.05 | 199.06 | 38.9 | 5 | 310.99 | 37.5 | 6 | 157.63 | 25 | 3 |  |  |  |  |  |  |  |  |  |
| NADH dehydrogenase [ubiquinone] 1 subunit C2 | NDUC2_HUMAN | 14235 | 9.05 | 97.34 | 16 | 3 | 92.21 | 16.8 | 3 |  |  |  |  |  |  |  |  |  |  |  |  |
| NADH-cytochrome b5 reductase 2 | NB5R2_HUMAN | 31495 | 8.50 | 684.43 | 74.6 | 16 | 628.65 | 64.1 | 14 | 463.66 | 46 | 11 | 686.06 | 79 | 16 | 123.8 | 32.2 | 4 | 88.69 | 14.1 | 3 |
| NADH-cytochrome b5 reductase 3 | NB5R3_HUMAN | 34441 | 7.18 |  |  |  | 104.85 | 10.3 | 3 |  |  |  | 61.03 | 10.3 | 2 |  |  |  |  |  |  |
| NADP-dependent malic enzyme | MAOX_HUMAN | 64679 | 5.80 | 1189.75 | 59.8 | 22 | 669.45 | 30.4 | 12 | 654.71 | 35.1 | 14 | 965.51 | 41.8 | 18 | 303.41 | 15.2 | 7 | 322.09 | 19.2 | 8 |
| Nebulin | NEBU_HUMAN | 775406 | 9.11 |  |  |  |  |  |  | 96.92 | 0.4 | 4 | 155 | 0.7 | 6 |  |  |  |  |  |  |
| Negative elongation factor B | NELFB_HUMAN | 66283 | 5.78 |  |  |  |  |  |  |  |  |  | 53.5 | 2.2 | 2 |  |  |  |  |  |  |
| Neogenin | NEO1_HUMAN | 160658 | 6.09 | 60.28 | 1.5 | 2 | 56.29 | 1.6 | 2 |  |  |  |  |  |  |  |  |  |  |  |  |
| Nephrocystin-3 | NPHP3_HUMAN | 151967 | 6.31 |  |  |  |  |  |  |  |  |  | 95.88 | 2 | 3 |  |  |  |  |  |  |
| Nesprin-1 | SYNE1_HUMAN | 1017069 | 5.39 | 167.9 | 0.6 | 7 | 171.62 | 0.7 | 7 | 94.24 | 0.4 | 4 | 204.26 | 1 | 9 |  |  |  |  |  |  |
| Nesprin-2 | SYNE2_HUMAN | 801817 | 5.26 |  |  |  |  |  |  | 64.25 | 0.4 | 3 | 310.22 | 1.4 | 12 |  |  |  |  |  |  |
| Nestin* | NEST_HUMAN | 177788 | 4.35 |  |  |  |  |  |  |  |  |  | 67.01 | 0.9 | 2 |  |  |  |  |  |  |
| Neural cell adhesion molecule 1 | NCAM1_HUMAN | 95370 | 4.79 |  |  |  | 282.4 | 7.8 | 5 |  |  |  | 744.9 | 21.8 | 16 | 273.14 | 8 | 5 |  |  |  |
| Neural cell adhesion molecule L1-like protein | CHL1_HUMAN | 136070 | 5.51 | 138.34 | 3.1 | 4 | 98.36 | 3.6 | 4 | 180.36 | 3.6 | 4 | 150.82 | 4.3 | 6 |  |  |  |  |  |  |
| Neurexin-3-alpha | NRX3A_HUMAN | 182651 | 5.33 |  |  |  | 212.43 | 3.7 | 5 |  |  |  | 468.44 | 8.3 | 12 | 70.38 | 0.9 | 2 |  |  |  |
| Neuroblastoma breakpoint family member 3 | NBPF3_HUMAN | 73704 | 4.40 |  |  |  |  |  |  |  |  |  | 65.02 | 2.7 | 2 |  |  |  |  |  |  |
| Neurofascin | NFASC_HUMAN | 150789 | 6.24 |  |  |  |  |  |  |  |  |  | 373.47 | 7.8 | 10 |  |  |  |  |  |  |
| Neurofilament heavy polypeptide | NFH_HUMAN | 112639 | 5.99 |  |  |  |  |  |  |  |  |  | 193.57 | 2.7 | 4 |  |  |  |  |  |  |
| Neurofilament light polypeptide | NFL_HUMAN | 61536 | 4.64 |  |  |  |  |  |  |  |  |  | 135.84 | 5.5 | 4 |  |  |  |  |  |  |
| Neuroligin-4. X-linked | NLGNX_HUMAN | 92427 | 5.78 |  |  |  |  |  |  |  |  |  | 96.5 | 3.4 | 2 |  |  |  |  |  |  |
| Neuronal cell adhesion molecule | NRCAM_HUMAN | 144655 | 5.46 | 426.83 | 10.7 | 11 | 445.25 | 9.7 | 10 | 234.37 | 4.4 | 5 | 964.26 | 20 | 21 | 454.72 | 11.3 | 10 | 90.73 | 2.4 | 2 |
| Neuroserpin | NEUS_HUMAN | 46397 | 4.85 |  |  |  | 79.56 | 7.1 | 2 |  |  |  | 414.01 | 23.9 | 8 | 213.42 | 12.9 | 4 |  |  |  |
| Neutrophil defensin 1 | DEF1_HUMAN | 10536 | 6.54 |  |  |  |  |  |  |  |  |  | 137 | 20.2 | 3 |  |  |  |  |  |  |
| Neutrophil gelatinase-associated lipocalin | NGAL_HUMAN | 22745 | 9.02 |  |  |  |  |  |  |  |  |  | 260.36 | 38.4 | 6 |  |  |  |  |  |  |
| NF-kappa-B-repressing factor | NKRF_HUMAN | 78308 | 8.94 |  |  |  |  |  |  |  |  |  | 52.51 | 2.6 | 2 |  |  |  |  |  |  |
| NIF3-like protein 1 | NIF3L_HUMAN | 42341 | 6.19 |  |  |  |  |  |  |  |  |  | 135.46 | 13.8 | 4 |  |  |  |  |  |  |
| Ninein | NIN_HUMAN | 245208 | 4.99 | 101.43 | 1.3 | 4 | 50.47 | 0.7 | 2 | 48.81 | 0.7 | 2 | 119.45 | 2.1 | 5 |  |  |  |  |  |  |
| Non-specific cytotoxic cell receptor protein 1 homolog* | NCRP1_HUMAN | 30942 | 6.16 |  |  |  |  |  |  |  |  |  | 56.82 | 5.8 | 2 |  |  |  |  |  |  |
| Nuclear mitotic apparatus protein 1 | NUMA1_HUMAN | 239199 | 5.63 |  |  |  |  |  |  | 66.36 | 1.1 | 3 | 110.1 | 1.9 | 5 |  |  |  |  |  |  |
| Nuclear receptor corepressor 2* | NCOR2_HUMAN | 275376 | 7.21 |  |  |  |  |  |  | 50.11 | 0.7 | 2 |  |  |  |  |  |  |  |  |  |
| Nuclear transport factor 2 | NTF2_HUMAN | 14640 | 5.11 |  |  |  | 67.18 | 18.9 | 2 |  |  |  |  |  |  | 60.04 | 18.9 | 2 |  |  |  |
| Nucleolar GTP-binding protein 1 | NOG1_HUMAN | 74317 | 9.53 | 55.93 | 3.2 | 2 |  |  |  |  |  |  |  |  |  |  |  |  |  |  |  |
| Nucleoside diphosphate kinase A | NDKA_HUMAN | 17309 | 5.84 | 270.98 | 52.6 | 7 | 283.37 | 46.1 | 6 | 357.59 | 57.2 | 8 | 254.64 | 40.8 | 6 | 249.18 | 31.6 | 5 | 179.13 | 46.7 | 5 |
| Nucleoside diphosphate kinase B | NDKB_HUMAN | 17401 | 8.53 | 390.33 | 70.4 | 9 | 300.67 | 53.3 | 7 | 367.5 | 57.9 | 8 | 375.08 | 60.5 | 8 | 392.99 | 53.9 | 7 | 137.55 | 31.6 | 4 |
| Nucleosome assembly protein 1-like 1 | NP1L1_HUMAN | 45631 | 4.37 | 77.23 | 5.4 | 2 |  |  |  | 55.97 | 5.4 | 2 |  |  |  | 116.51 | 10 | 3 |  |  |  |
| Nucleosome assembly protein 1-like 4 | NP1L4_HUMAN | 42968 | 4.61 | 361.63 | 24.5 | 7 | 163.14 | 9.9 | 3 | 156.76 | 9.6 | 3 | 77.26 | 6.9 | 2 | 426.61 | 26.7 | 8 |  |  |  |
| Obg-like ATPase 1 | OLA1_HUMAN | 44943 | 7.65 | 159.23 | 8.3 | 3 |  |  |  |  |  |  | 125.98 | 8.3 | 3 |  |  |  |  |  |  |
| Obscurin | OBSCN_HUMAN | 879630 | 5.70 | 59.13 | 0.2 | 2 | 47.29 | 0.2 | 2 |  |  |  | 67.91 | 0.2 | 2 |  |  |  |  |  |  |
| Oligodendrocyte-myelin glycoprotein* | OMGP_HUMAN | 50032 | 8.06 |  |  |  |  |  |  |  |  |  | 55.49 | 4.1 | 2 |  |  |  |  |  |  |
| Omega-amidase NIT2 | NIT2_HUMAN | 30988 | 6.82 |  |  |  |  |  |  |  |  |  | 138.13 | 10.9 | 3 |  |  |  |  |  |  |
| Oncostatin-M* | ONCM_HUMAN | 28751 | 10.71 |  |  |  |  |  |  |  |  |  | 50.59 | 6.3 | 2 |  |  |  |  |  |  |
| Opticin | OPT_HUMAN | 37579 | 5.40 | 483.44 | 30.7 | 7 | 587.73 | 46.1 | 11 | 467.79 | 28 | 9 | 699.09 | 46.4 | 13 | 820.13 | 37.7 | 12 | 180.11 | 12 | 3 |
| Oral-facial-digital syndrome 1 protein | OFD1_HUMAN | 117055 | 5.82 |  |  |  |  |  |  |  |  |  | 140.03 | 3.2 | 6 | 58.4 | 1.3 | 2 |  |  |  |
| Osteoclast-stimulating factor 1 | OSTF1_HUMAN | 23943 | 5.47 | 80.63 | 9.3 | 2 |  |  |  | 58.01 | 9.3 | 2 | 82.9 | 9.3 | 2 |  |  |  |  |  |  |
| Osteopontin | OSTP_HUMAN | 35572 | 4.37 | 210.81 | 14.6 | 3 | 75.72 | 7 | 2 | 67.85 | 7 | 2 | 311.95 | 25.8 | 6 | 651.04 | 55.4 | 11 | 257.94 | 24.2 | 5 |
| Out at first protein homolog | OAF_HUMAN | 31239 | 6.40 |  |  |  | 105.79 | 8.8 | 2 |  |  |  | 260.29 | 16.8 | 4 | 147.13 | 10.3 | 2 |  |  |  |
| Oxysterol-binding protein-related protein 7* | OSBL7_HUMAN | 96398 | 8.31 |  |  |  |  |  |  |  |  |  | 49.42 | 2.6 | 2 |  |  |  |  |  |  |
| Palladin* | PALLD_HUMAN | 151839 | 6.67 |  |  |  |  |  |  |  |  |  | 59.71 | 1.4 | 2 |  |  |  |  |  |  |
| Pappalysin-2 | PAPP2_HUMAN | 203486 | 5.27 | 114.7 | 2.7 | 4 | 266.86 | 3.7 | 5 | 256.69 | 6.1 | 8 |  |  |  |  |  |  |  |  |  |
| Paralemmin-1 | PALM_HUMAN | 42221 | 4.94 |  |  |  |  |  |  |  |  |  |  |  |  | 95.18 | 9.6 | 3 |  |  |  |
| Parathyroid hormone-related protein | PTHR_HUMAN | 20239 | 10.20 |  |  |  | 93.1 | 19.8 | 3 |  |  |  |  |  |  |  |  |  |  |  |  |
| PDZ domain-containing protein 2* | PDZD2_HUMAN | 303964 | 7.14 |  |  |  |  |  |  |  |  |  | 54.52 | 0.6 | 2 |  |  |  |  |  |  |
| Pecanex-like protein 2* | PCX2_HUMAN | 239978 | 6.29 |  |  |  |  |  |  |  |  |  | 46.82 | 0.7 | 2 |  |  |  |  |  |  |
| Pecanex-like protein 3* | PCX3_HUMAN | 224293 | 6.20 |  |  |  |  |  |  |  |  |  | 50.81 | 0.7 | 2 |  |  |  |  |  |  |
| Peptidase inhibitor 16 | PI16_HUMAN | 50124 | 5.24 |  |  |  |  |  |  |  |  |  |  |  |  | 59.8 | 4.3 | 2 |  |  |  |
| Peptidyl-prolyl cis-trans isomerase A | PPIA_HUMAN | 18229 | 7.69 | 565.98 | 72.7 | 11 | 405.05 | 58.8 | 7 | 340.49 | 58.8 | 8 | 375.62 | 44.8 | 7 | 414.07 | 50.9 | 8 | 316.78 | 53.9 | 6 |
| Peptidyl-prolyl cis-trans isomerase F. mitochondrial | PPIF_HUMAN | 22368 | 9.48 |  |  |  |  |  |  |  |  |  |  |  |  | 87.13 | 15.9 | 2 |  |  |  |
| Peptidyl-prolyl cis-trans isomerase FKBP5 | FKBP5_HUMAN | 51693 | 5.70 |  |  |  |  |  |  |  |  |  | 54.25 | 3.9 | 2 |  |  |  |  |  |  |
| Periaxin | PRAX_HUMAN | 155149 | 7.22 | 82.49 | 1.2 | 2 | 78.16 | 1.2 | 2 |  |  |  |  |  |  |  |  |  |  |  |  |
| Perilipin-3 | PLIN3_HUMAN | 47217 | 5.30 |  |  |  |  |  |  |  |  |  | 426.22 | 23.3 | 6 |  |  |  |  |  |  |
| Period circadian protein homolog 2* | PER2_HUMAN | 138318 | 6.05 |  |  |  | 49.55 | 1.8 | 2 |  |  |  |  |  |  |  |  |  |  |  |  |
| Periostin | POSTN_HUMAN | 93883 | 7.28 |  |  |  |  |  |  |  |  |  | 55.62 | 3 | 2 |  |  |  |  |  |  |
| Periplakin | PEPL_HUMAN | 205193 | 5.47 | 201.88 | 3.9 | 6 | 62.45 | 1.1 | 2 |  |  |  | 317.34 | 5.7 | 10 |  |  |  |  |  |  |
| Peroxiredoxin-1 | PRDX1_HUMAN | 22324 | 8.28 | 126.61 | 15.1 | 3 | 113.95 | 14.1 | 3 |  |  |  | 271.39 | 36.7 | 8 |  |  |  | 112.4 | 21.1 | 3 |
| Peroxiredoxin-2 | PRDX2_HUMAN | 22049 | 5.67 | 286.2 | 30.8 | 7 | 242.79 | 30.3 | 6 | 287.1 | 27.3 | 6 | 409.53 | 36.4 | 9 | 206.12 | 16.2 | 4 | 58.92 | 14.1 | 2 |
| Peroxiredoxin-4 | PRDX4_HUMAN | 30749 | 5.87 |  |  |  |  |  |  |  |  |  | 98.76 | 10.3 | 3 |  |  |  |  |  |  |
| Peroxiredoxin-5. mitochondrial | PRDX5_HUMAN | 22301 | 8.94 |  |  |  |  |  |  | 114.64 | 17.3 | 3 | 84.85 | 8.9 | 2 |  |  |  |  |  |  |
| Peroxiredoxin-6 | PRDX6_HUMAN | 25133 | 6.00 | 799.16 | 70.5 | 15 | 864.53 | 72.8 | 15 | 883.87 | 76.3 | 16 | 854.5 | 70.5 | 15 | 332.56 | 36.2 | 7 | 643.68 | 64.7 | 12 |
| Peroxisomal acyl-coenzyme A oxidase 2* | ACOX2_HUMAN | 77576 | 7.32 |  |  |  |  |  |  |  |  |  | 53.1 | 3.5 | 2 |  |  |  |  |  |  |
| Peroxisomal NADH pyrophosphatase NUDT12* | NUD12_HUMAN | 52784 | 6.38 | 50.49 | 4.1 | 2 |  |  |  |  |  |  |  |  |  |  |  |  |  |  |  |
| PERQ amino acid-rich with GYF domain-containing protein 1* | PERQ1_HUMAN | 115044 | 5.29 | 51.96 | 1.5 | 2 |  |  |  | 47.03 | 1.5 | 2 | 56.94 | 1.5 | 2 |  |  |  |  |  |  |
| Phakinin | BFSP2_HUMAN | 46193 | 5.41 | 1286.14 | 62.4 | 24 | 2467.24 | 83.1 | 36 | 1947.41 | 69.2 | 28 | 2572.66 | 87.7 | 37 | 2708.93 | 85.8 | 40 | 2176.23 | 84.6 | 34 |
| Phosphatidylethanolamine-binding protein 1 | PEBP1_HUMAN | 21158 | 7.02 | 625.12 | 73.3 | 9 | 566.2 | 72.7 | 10 | 531.17 | 69 | 8 | 461.3 | 58.8 | 7 | 647.24 | 77.5 | 10 | 548.97 | 75.9 | 9 |
| Phosphatidylethanolamine-binding protein 4* | PEBP4_HUMAN | 26002 | 6.08 |  |  |  |  |  |  |  |  |  | 51.96 | 9.3 | 2 |  |  |  |  |  |  |
| Phosphatidylinositol 4.5-bisphosphate 3-kinase catalytic subunit gamma isoform | PK3CG_HUMAN | 127571 | 7.24 |  |  |  |  |  |  |  |  |  | 90.38 | 4.2 | 4 |  |  |  |  |  |  |
| Phosphoglucomutase-like protein 5 | PGM5_HUMAN | 62756 | 6.82 | 183.99 | 6.7 | 3 | 127.79 | 4.1 | 2 | 298.51 | 12.5 | 6 | 313.62 | 16.8 | 7 |  |  |  |  |  |  |
| Phosphoglycerate kinase 1 | PGK1_HUMAN | 44985 | 8.30 | 1930.72 | 79.9 | 33 | 1859.49 | 79.6 | 32 | 1898.51 | 79.9 | 34 | 1958.25 | 75.8 | 33 | 1575.9 | 68.6 | 26 | 1306.95 | 75.3 | 24 |
| Phosphoglycerate kinase 2 | PGK2_HUMAN | 45166 | 8.75 | 405.46 | 27.8 | 9 | 391.98 | 19.2 | 7 |  |  |  | 442.49 | 19.2 | 7 |  |  |  |  |  |  |
| Phosphoglycerate mutase 1 | PGAM1_HUMAN | 28900 | 6.67 | 1160.09 | 77.6 | 17 | 1154.55 | 80.7 | 19 | 779.3 | 66.5 | 12 | 948.46 | 71.7 | 15 | 633.32 | 55.9 | 10 | 499.54 | 52.4 | 11 |
| Phosphoglycerate mutase 2 | PGAM2_HUMAN | 28919 | 8.99 | 421.27 | 25.7 | 7 | 371.74 | 22.1 | 7 |  |  |  | 402.79 | 32.8 | 8 |  |  |  |  |  |  |
| Phospholipid transfer protein | PLTP_HUMAN | 54933 | 6.53 |  |  |  |  |  |  |  |  |  | 724.8 | 30.6 | 13 | 271.47 | 15 | 5 |  |  |  |
| Phospholysine phosphohistidine inorganic pyrophosphate phosphatase | LHPP_HUMAN | 29432 | 5.81 |  |  |  | 82.15 | 11.1 | 2 |  |  |  | 78.64 | 21.9 | 3 |  |  |  |  |  |  |
| Phosphopantothenate--cysteine ligase | PPCS_HUMAN | 33984 | 6.26 |  |  |  |  |  |  |  |  |  | 141.34 | 9 | 4 |  |  |  |  |  |  |
| Phosphorylase b kinase regulatory subunit beta* | KPBB_HUMAN | 125946 | 6.51 |  |  |  |  |  |  | 52.14 | 1.8 | 2 |  |  |  |  |  |  |  |  |  |
| Phosphoserine phosphatase | SERB_HUMAN | 25163 | 5.54 |  |  |  |  |  |  |  |  |  | 154.26 | 12.9 | 3 |  |  |  |  |  |  |
| Pigment epithelium-derived factor | PEDF_HUMAN | 46454 | 5.98 | 1403.69 | 68.7 | 24 | 1352.33 | 59.8 | 21 | 1153.01 | 46.7 | 18 | 1529.37 | 60 | 25 | 1137.97 | 54.3 | 19 | 1005.75 | 45.2 | 17 |
| PITH domain-containing protein 1 | PITH1_HUMAN | 24391 | 5.47 | 72.61 | 14.2 | 2 |  |  |  | 105.93 | 18 | 2 | 235.5 | 32.2 | 6 | 122.32 | 19.9 | 4 |  |  |  |
| Plasma protease C1 inhibitor | IC1_HUMAN | 55347 | 6.10 | 795.39 | 27.2 | 14 | 1383.09 | 42.4 | 23 | 1087.9 | 35.4 | 18 | 1061.84 | 31.6 | 17 | 1304.61 | 36 | 20 | 576.19 | 24.6 | 11 |
| Plasma serine protease inhibitor | IPSP_HUMAN | 45760 | 9.31 | 52.66 | 5.9 | 2 |  |  |  |  |  |  | 372.18 | 18.2 | 7 |  |  |  |  |  |  |
| Plasmalemma vesicle-associated protein | PLVAP_HUMAN | 51132 | 9.01 | 64.68 | 3.4 | 2 |  |  |  |  |  |  |  |  |  |  |  |  |  |  |  |
| Plasminogen | PLMN_HUMAN | 93247 | 7.05 | 1747.28 | 58.8 | 34 | 1012.99 | 27.7 | 18 |  |  |  | 817.87 | 23.6 | 18 |  |  |  | 162.95 | 5.7 | 4 |
| Platelet-activating factor acetylhydrolase IB subunit alpha | LIS1_HUMAN | 47178 | 6.98 |  |  |  |  |  |  |  |  |  | 150.01 | 7.6 | 3 |  |  |  |  |  |  |
| Pleckstrin homology domain-containing family H member 2 | PKHH2_HUMAN | 169720 | 7.50 |  |  |  |  |  |  |  |  |  | 84.19 | 1.3 | 3 |  |  |  |  |  |  |
| Plectin | PLEC_HUMAN | 533462 | 5.75 | 2901.99 | 15.1 | 69 | 967.36 | 5.3 | 25 | 883.71 | 5.3 | 25 | 1899.07 | 10.3 | 47 | 189.16 | 1.5 | 6 | 197.29 | 1.2 | 7 |
| Plexin domain-containing protein 2 | PXDC2_HUMAN | 60116 | 5.99 |  |  |  |  |  |  |  |  |  | 447.14 | 20.4 | 10 | 177.26 | 10 | 4 |  |  |  |
| Plexin-B2 | PLXB2_HUMAN | 207734 | 5.86 |  |  |  | 122.4 | 1.9 | 4 | 50.09 | 0.8 | 2 | 560.77 | 7.3 | 12 |  |  |  |  |  |  |
| Poly(ADP-ribose) glycohydrolase ARH3 | ARHL2_HUMAN | 39264 | 4.95 |  |  |  |  |  |  |  |  |  | 153.85 | 10.5 | 4 |  |  |  |  |  |  |
| Polyadenylate-binding protein 4* | PABP4_HUMAN | 71080 | 9.31 | 50.56 | 2.3 | 2 |  |  |  |  |  |  |  |  |  |  |  |  |  |  |  |
| Polymeric immunoglobulin receptor | PIGR_HUMAN | 84429 | 5.59 |  |  |  | 150.61 | 5.6 | 4 |  |  |  | 1700.97 | 41.8 | 31 |  |  |  |  |  |  |
| PRAME family member 9/15* | PRAM9_HUMAN | 56751 | 8.83 |  |  |  |  |  |  |  |  |  | 51.47 | 2.9 | 2 |  |  |  |  |  |  |
| Pre-mRNA-splicing factor ISY1 homolog* | ISY1_HUMAN | 33029 | 5.15 | 50.49 | 4.9 | 2 |  |  |  |  |  |  |  |  |  |  |  |  |  |  |  |
| Proactivator polypeptide | SAP_HUMAN | 59899 | 5.06 |  |  |  |  |  |  |  |  |  | 111.73 | 6.1 | 3 |  |  |  |  |  |  |
| Probable ATP-dependent RNA helicase DDX23* | DDX23_HUMAN | 95866 | 9.58 |  |  |  | 46.67 | 1.8 | 2 |  |  |  | 51.21 | 1.8 | 2 |  |  |  |  |  |  |
| Probable ATP-dependent RNA helicase DDX46 | DDX46_HUMAN | 117803 | 9.34 |  |  |  |  |  |  |  |  |  | 79.6 | 2.2 | 3 |  |  |  |  |  |  |
| Probable ATP-dependent RNA helicase YTHDC2* | YTDC2_HUMAN | 161573 | 8.67 |  |  |  |  |  |  |  |  |  | 51.71 | 1.1 | 2 |  |  |  |  |  |  |
| Probable E3 ubiquitin-protein ligase MYCBP2 | MYCB2_HUMAN | 517856 | 6.63 | 76.69 | 0.4 | 3 | 73.09 | 0.6 | 3 |  |  |  |  |  |  |  |  |  |  |  |  |
| Probable JmjC domain-containing histone demethylation protein 2C | JHD2C_HUMAN | 286401 | 7.95 | 72.07 | 0.8 | 3 |  |  |  |  |  |  | 95.9 | 1 | 4 |  |  |  |  |  |  |
| Probable tRNA (uracil-O(2)-)-methyltransferase* | TRM44_HUMAN | 85944 | 6.98 |  |  |  |  |  |  |  |  |  | 74.26 | 1.6 | 2 |  |  |  |  |  |  |
| Procollagen-lysine.2-oxoglutarate 5-dioxygenase 3* | PLOD3_HUMAN | 85302 | 5.69 |  |  |  |  |  |  |  |  |  | 80.04 | 3 | 2 |  |  |  |  |  |  |
| Profilin-1 | PROF1_HUMAN | 15216 | 8.44 | 324.48 | 48.6 | 7 | 333.14 | 51.4 | 6 | 363.24 | 54.3 | 6 | 293.45 | 44.3 | 5 |  |  |  |  |  |  |
| Programmed cell death 6-interacting protein | PDC6I_HUMAN | 96590 | 6.13 |  |  |  |  |  |  |  |  |  | 541.07 | 16.6 | 14 |  |  |  |  |  |  |
| Prolactin-inducible protein | PIP_HUMAN | 16847 | 8.27 |  |  |  |  |  |  | 104.96 | 21.9 | 4 | 424.87 | 54.8 | 8 |  |  |  |  |  |  |
| Prolow-density lipoprotein receptor-related protein 1 | LRP1_HUMAN | 523150 | 5.16 |  |  |  |  |  |  | 110.06 | 0.4 | 2 | 76.45 | 0.6 | 3 |  |  |  |  |  |  |
| Prolyl endopeptidase | PPCE_HUMAN | 81560 | 5.53 | 104.34 | 4.1 | 2 |  |  |  |  |  |  | 638.26 | 22.3 | 13 |  |  |  |  |  |  |
| Prostaglandin-H2 D-isomerase | PTGDS_HUMAN | 21243 | 7.66 | 352.84 | 32.1 | 7 | 450.6 | 47.4 | 8 | 437.12 | 51.6 | 8 | 665.98 | 64.7 | 10 | 370.85 | 44.2 | 6 | 438.53 | 51.6 | 8 |
| Proteasome assembly chaperone 2* | PSMG2_HUMAN | 29776 | 6.60 |  |  |  |  |  |  |  |  |  | 53.25 | 4.5 | 2 |  |  |  |  |  |  |
| Proteasome inhibitor PI31 subunit | PSMF1_HUMAN | 29969 | 5.42 |  |  |  |  |  |  |  |  |  |  |  |  | 79.83 | 7.7 | 2 |  |  |  |
| Proteasome subunit alpha type-1 | PSA1_HUMAN | 29822 | 6.16 |  |  |  |  |  |  | 57.46 | 6.1 | 2 | 183.91 | 21.3 | 6 |  |  |  |  |  |  |
| Proteasome subunit alpha type-3* | PSA3_HUMAN | 28643 | 5.20 | 48.89 | 7.8 | 2 |  |  |  |  |  |  | 70.7 | 10.2 | 2 |  |  |  |  |  |  |
| Proteasome subunit alpha type-4 | PSA4_HUMAN | 29750 | 7.58 | 72.81 | 6.9 | 2 | 104.14 | 6.9 | 2 |  |  |  | 213.22 | 18.8 | 5 |  |  |  |  |  |  |
| Proteasome subunit alpha type-5* | PSA5_HUMAN | 26565 | 4.74 |  |  |  |  |  |  |  |  |  | 211.31 | 25.3 | 5 | 80.32 | 13.3 | 2 |  |  |  |
| Proteasome subunit alpha type-6* | PSA6_HUMAN | 27838 | 6.35 | 73.49 | 9.3 | 2 | 124.24 | 9.8 | 2 |  |  |  | 177.91 | 17.9 | 4 |  |  |  |  |  |  |
| Proteasome subunit alpha type-7 | PSA7_HUMAN | 28041 | 8.60 |  |  |  |  |  |  |  |  |  | 278.76 | 28.6 | 5 |  |  |  |  |  |  |
| Proteasome subunit beta type-5 | PSB5_HUMAN | 28633 | 6.43 |  |  |  |  |  |  |  |  |  | 86.74 | 6.8 | 2 |  |  |  | 72.82 | 8.7 | 2 |
| Proteasome subunit beta type-6 | PSB6_HUMAN | 25570 | 4.80 |  |  |  |  |  |  |  |  |  | 73.52 | 8.4 | 2 | 54.28 | 8.8 | 2 |  |  |  |
| Protein 4.1 | 41_HUMAN | 97528 | 5.42 |  |  |  | 199.67 | 5.2 | 5 |  |  |  | 54.6 | 2.7 | 2 | 101.29 | 4.4 | 3 |  |  |  |
| Protein AHNAK2* | AHNK2_HUMAN | 617383 | 5.20 |  |  |  | 49.21 | 0.3 | 2 |  |  |  |  |  |  |  |  |  |  |  |  |
| Protein AMBP | AMBP_HUMAN | 39886 | 5.96 | 438.61 | 31 | 8 | 248.14 | 16.2 | 5 | 371.92 | 32.4 | 8 | 416.32 | 19.6 | 6 | 412.15 | 26.7 | 7 |  |  |  |
| Protein CutA | CUTA_HUMAN | 19218 | 5.42 |  |  |  |  |  |  |  |  |  |  |  |  | 96.12 | 22.9 | 2 |  |  |  |
| Protein diaphanous homolog 2 | DIAP2_HUMAN | 126231 | 6.20 |  |  |  |  |  |  |  |  |  | 74.97 | 2.5 | 3 |  |  |  |  |  |  |
| Protein DJ-1 | PARK7_HUMAN | 20050 | 6.33 | 1032.9 | 90.5 | 17 | 679.83 | 68.3 | 11 | 783.51 | 75.7 | 13 | 789.37 | 83.6 | 14 | 783.47 | 75.7 | 13 | 761.89 | 77.2 | 15 |
| Protein ELYS | ELYS_HUMAN | 254223 | 6.19 |  |  |  |  |  |  |  |  |  | 72.78 | 1.6 | 3 |  |  |  |  |  |  |
| Protein FAM3C | FAM3C_HUMAN | 24950 | 8.52 |  |  |  | 319.59 | 26.9 | 5 | 164.64 | 18.9 | 4 |  |  |  |  |  |  |  |  |  |
| Protein FAM49B | FA49B_HUMAN | 37010 | 5.76 | 205.49 | 21.6 | 5 | 241.72 | 22.5 | 6 | 98.82 | 8.6 | 2 | 306.51 | 25.6 | 6 |  |  |  |  |  |  |
| Protein FAM65A* | FA65A_HUMAN | 133423 | 5.87 |  |  |  |  |  |  |  |  |  | 49.53 | 2 | 2 |  |  |  |  |  |  |
| Protein furry homolog | FRY_HUMAN | 342080 | 5.66 |  |  |  |  |  |  |  |  |  | 89.17 | 0.7 | 3 |  |  |  |  |  |  |
| Protein hid-1 homolog* | CQ028_HUMAN | 89316 | 5.69 |  |  |  |  |  |  |  |  |  | 48.93 | 1.9 | 2 |  |  |  |  |  |  |
| Protein kinase C and casein kinase substrate in neurons protein 3* | PACN3_HUMAN | 48799 | 5.84 |  |  |  | 53.38 | 3.5 | 2 |  |  |  |  |  |  |  |  |  |  |  |  |
| Protein MB21D2* | M21D2_HUMAN | 56392 | 6.58 | 49.64 | 2.9 | 2 |  |  |  |  |  |  |  |  |  |  |  |  |  |  |  |
| Protein NDRG1 | NDRG1_HUMAN | 43264 | 5.50 |  |  |  |  |  |  |  |  |  | 119.1 | 12.9 | 3 |  |  |  |  |  |  |
| Protein NPAT | NPAT_HUMAN | 155506 | 5.63 |  |  |  |  |  |  |  |  |  | 52.42 | 1.5 | 2 |  |  |  |  |  |  |
| Protein piccolo | PCLO_HUMAN | 554704 | 6.09 |  |  |  |  |  |  |  |  |  | 73.29 | 0.5 | 3 |  |  |  |  |  |  |
| Protein PML | PML_HUMAN | 99143 | 5.88 |  |  |  |  |  |  |  |  |  | 55.32 | 2.4 | 2 |  |  |  |  |  |  |
| Protein S100-A4 | S10A4_HUMAN | 11949 | 5.85 | 205.07 | 28.7 | 4 | 131.8 | 16.8 | 3 |  |  |  | 87.73 | 16.8 | 2 |  |  |  |  |  |  |
| Protein S100-A6 | S10A6_HUMAN | 10230 | 5.33 | 86.62 | 16.7 | 2 | 78.13 | 16.7 | 2 |  |  |  |  |  |  |  |  |  |  |  |  |
| Protein S100-A7 | S10A7_HUMAN | 11578 | 6.29 |  |  |  | 192.94 | 41.6 | 4 | 195.29 | 41.6 | 4 | 140.04 | 44.6 | 3 |  |  |  |  |  |  |
| Protein S100-A8 | S10A8_HUMAN | 10885 | 6.51 | 173.72 | 35.5 | 4 |  |  |  | 201.21 | 35.5 | 4 | 371.45 | 49.5 | 7 | 146.77 | 31.2 | 3 |  |  |  |
| Protein S100-A9 | S10A9_HUMAN | 13291 | 5.72 |  |  |  |  |  |  | 106.13 | 19.3 | 2 | 347.56 | 55.3 | 7 | 237.85 | 43.9 | 4 |  |  |  |
| Protein S100-B | S100B_HUMAN | 10820 | 4.56 | 196.57 | 40.2 | 3 | 157.43 | 32.6 | 2 |  |  |  |  |  |  | 210.21 | 40.2 | 3 | 104.97 | 40.2 | 2 |
| Protein Shroom3* | SHRM3_HUMAN | 218321 | 7.87 |  |  |  |  |  |  | 65.22 | 0.9 | 2 |  |  |  |  |  |  |  |  |  |
| Protein Spindly | SPDLY_HUMAN | 70755 | 5.42 |  |  |  |  |  |  |  |  |  | 56.57 | 2.5 | 2 |  |  |  |  |  |  |
| Protein unc-45 homolog A* | UN45A_HUMAN | 104266 | 5.80 |  |  |  |  |  |  |  |  |  | 56.9 | 1.7 | 2 |  |  |  |  |  |  |
| Protein XRP2 | XRP2_HUMAN | 40471 | 5.00 |  |  |  |  |  |  |  |  |  | 79.38 | 5.1 | 2 |  |  |  |  |  |  |
| Protein-glutamine gamma-glutamyltransferase 2 | TGM2_HUMAN | 78420 | 5.11 |  |  |  |  |  |  |  |  |  | 377.92 | 14.7 | 9 |  |  |  |  |  |  |
| Protein-glutamine gamma-glutamyltransferase E | TGM3_HUMAN | 76926 | 5.62 |  |  |  | 175.65 | 4.3 | 3 | 196.38 | 7.4 | 5 | 595.78 | 19 | 13 | 78.61 | 4.5 | 2 |  |  |  |
| Protein-glutamine gamma-glutamyltransferase K | TGM1_HUMAN | 90529 | 5.68 |  |  |  |  |  |  |  |  |  | 121.87 | 3.8 | 3 |  |  |  |  |  |  |
| Protein-L-isoaspartate(D-aspartate) O-methyltransferase | PIMT_HUMAN | 24792 | 6.71 | 571.21 | 66.1 | 10 | 462.09 | 62.6 | 9 | 491.02 | 62.6 | 9 | 411.59 | 51.1 | 8 | 122.84 | 28.2 | 3 | 415 | 58.1 | 8 |
| Protein-tyrosine kinase 2-beta* | FAK2_HUMAN | 117112 | 5.92 | 51.24 | 1.5 | 2 |  |  |  |  |  |  |  |  |  |  |  |  |  |  |  |
| Prothrombin | THRB_HUMAN | 71475 | 5.65 | 1032 | 38.9 | 19 | 183.61 | 9 | 5 | 225.5 | 9.2 | 6 | 423.85 | 16.7 | 9 | 255.31 | 12.5 | 5 |  |  |  |
| Protocadherin alpha-C2 | PCDC2_HUMAN | 110010 | 5.27 |  |  |  |  |  |  |  |  |  | 137.43 | 3.1 | 3 |  |  |  |  |  |  |
| Protocadherin gamma-C3 | PCDGK_HUMAN | 101301 | 5.07 |  |  |  |  |  |  |  |  |  | 73.46 | 4.3 | 3 |  |  |  |  |  |  |
| Pterin-4-alpha-carbinolamine dehydratase | PHS_HUMAN | 12049 | 6.29 | 281.91 | 49 | 7 | 237.47 | 57.7 | 6 | 172.35 | 39.4 | 4 | 206.07 | 51.9 | 6 |  |  |  | 84.25 | 28.8 | 3 |
| Purine nucleoside phosphorylase | PNPH_HUMAN | 32325 | 6.45 |  |  |  | 82.23 | 10.4 | 3 | 115.24 | 12.1 | 2 | 514.99 | 36.7 | 9 |  |  |  |  |  |  |
| Puromycin-sensitive aminopeptidase | PSA_HUMAN | 103895 | 5.50 | 1266.62 | 34.5 | 26 | 930.99 | 23.8 | 16 |  |  |  | 1560.88 | 44 | 31 | 64.3 | 2.5 | 2 |  |  |  |
| Putative adenosylhomocysteinase 2 | SAHH2_HUMAN | 59997 | 6.49 |  |  |  |  |  |  | 77.19 | 4.2 | 3 |  |  |  |  |  |  |  |  |  |
| Putative disintegrin and metalloproteinase domain-containing protein 5 | ADAM5_HUMAN | 48633 | 6.35 |  |  |  |  |  |  |  |  |  | 73.44 | 5.3 | 3 |  |  |  |  |  |  |
| Putative heat shock protein HSP 90-alpha A5 | HS905_HUMAN | 38942 | 6.15 |  |  |  |  |  |  |  |  |  | 142.89 | 13.5 | 4 |  |  |  |  |  |  |
| Putative MAGE domain-containing protein MAGEA13P | MA13P_HUMAN | 38388 | 5.99 | 55.76 | 4.1 | 2 |  |  |  |  |  |  |  |  |  |  |  |  |  |  |  |
| Putative phospholipase B-like 2* | PLBL2_HUMAN | 65886 | 6.34 |  |  |  |  |  |  |  |  |  | 62.39 | 2.7 | 2 |  |  |  |  |  |  |
| Putative protein PLEKHA9* | PKHA9_HUMAN | 44081 | 5.03 |  |  |  |  |  |  |  |  |  | 52.6 | 4.1 | 2 |  |  |  |  |  |  |
| Putative RNA-binding protein 15 | RBM15_HUMAN | 107352 | 10.09 |  |  |  |  |  |  |  |  |  | 83.02 | 1.9 | 3 |  |  |  |  |  |  |
| Putative tropomyosin alpha-3 chain-like protein | TPM3L_HUMAN | 26595 | 4.47 |  |  |  |  |  |  |  |  |  | 246.25 | 18.4 | 5 | 168.93 | 13 | 3 |  |  |  |
| Putative ubiquitin carboxyl-terminal hydrolase 17-like protein 1 | U17L1_HUMAN | 60692 | 7.82 |  |  |  | 62.43 | 2.6 | 2 |  |  |  |  |  |  |  |  |  |  |  |  |
| PX domain-containing protein 1 | PXDC1_HUMAN | 56238 | 5.49 |  |  |  |  |  |  |  |  |  | 58.73 | 3.4 | 2 | 96.63 | 7 | 3 |  |  |  |
| Pyridoxal kinase | PDXK_HUMAN | 35308 | 5.75 |  |  |  |  |  |  |  |  |  | 199.23 | 17.3 | 5 |  |  |  |  |  |  |
| Pyruvate kinase isozymes M1/M2 | KPYM_HUMAN | 58470 | 7.96 | 1755.68 | 64 | 29 | 1619.62 | 65.5 | 28 | 1532.02 | 51.6 | 24 | 1458.75 | 50.3 | 22 | 897.19 | 35.8 | 16 | 1253.43 | 53.9 | 23 |
| Pyruvate kinase isozymes R/L | KPYR_HUMAN | 62191 | 7.64 | 95.34 | 4 | 3 |  |  |  | 104.39 | 4 | 3 | 150.42 | 6.3 | 4 |  |  |  |  |  |  |
| Quinone oxidoreductase | QOR_HUMAN | 35356 | 8.56 | 557.64 | 44.4 | 12 | 752.07 | 60.5 | 14 |  |  |  | 936.11 | 76 | 18 | 57.18 | 11.6 | 2 | 195.17 | 15.2 | 4 |
| Quinone oxidoreductase PIG3 | QORX_HUMAN | 35685 | 6.67 | 849.33 | 58.4 | 15 | 830.89 | 46.7 | 14 | 100.1 | 8.7 | 3 | 917.86 | 53.6 | 16 | 260.11 | 23.2 | 5 | 153.94 | 16.3 | 4 |
| Rab GDP dissociation inhibitor alpha | GDIA_HUMAN | 51177 | 5.01 | 1296.56 | 72.9 | 23 | 726.23 | 37.6 | 12 | 1135.18 | 61.3 | 20 | 1282.17 | 53.7 | 21 | 1124.23 | 53.9 | 18 | 447.24 | 27.7 | 9 |
| Rab GDP dissociation inhibitor beta | GDIB_HUMAN | 51087 | 6.12 | 1048.21 | 50.1 | 18 | 453 | 21.3 | 8 | 489.39 | 21.6 | 8 | 999.51 | 45.2 | 15 | 407.58 | 18.7 | 8 | 248.78 | 14.8 | 6 |
| Rab GTPase-binding effector protein 1* | RABE1_HUMAN | 99629 | 4.95 |  |  |  |  |  |  | 48.62 | 1.6 | 2 |  |  |  |  |  |  |  |  |  |
| Rab11 family-interacting protein 3* | RFIP3_HUMAN | 83073 | 4.44 |  |  |  |  |  |  |  |  |  | 56.7 | 2.1 | 2 |  |  |  |  |  |  |
| Rab11 family-interacting protein 4* | RFIP4_HUMAN | 72568 | 4.78 |  |  |  |  |  |  |  |  |  | 49.8 | 3.6 | 2 |  |  |  |  |  |  |
| RAD51-associated protein 2 | R51A2_HUMAN | 135761 | 7.12 |  |  |  |  |  |  | 69.59 | 2 | 3 | 54.4 | 1.3 | 2 |  |  |  |  |  |  |
| Radixin | RADI_HUMAN | 68635 | 6.03 |  |  |  |  |  |  | 69.81 | 2.7 | 2 | 242.21 | 11.8 | 8 | 198.91 | 7.7 | 5 |  |  |  |
| Ran-binding protein 6 | RNBP6_HUMAN | 126173 | 4.89 |  |  |  |  |  |  |  |  |  | 176.33 | 4.1 | 5 |  |  |  |  |  |  |
| Rap guanine nucleotide exchange factor 4 | RPGF4_HUMAN | 116474 | 6.37 |  |  |  |  |  |  |  |  |  | 75.95 | 2.4 | 3 |  |  |  |  |  |  |
| Ras and EF-hand domain-containing protein | RASEF_HUMAN | 83569 | 4.99 |  |  |  |  |  |  |  |  |  | 53.24 | 3.1 | 2 |  |  |  |  |  |  |
| Ras GTPase-activating-like protein IQGAP1* | IQGA1_HUMAN | 189761 | 6.08 |  |  |  | 49.01 | 1.2 | 2 |  |  |  |  |  |  |  |  |  |  |  |  |
| Ras-GEF domain-containing family member 1B | RGF1B_HUMAN | 55894 | 8.19 | 55.55 | 3.4 | 2 |  |  |  |  |  |  |  |  |  |  |  |  |  |  |  |
| Ras-related C3 botulinum toxin substrate 1 | RAC1_HUMAN | 21835 | 8.78 |  |  |  | 111.93 | 16.1 | 3 |  |  |  |  |  |  |  |  |  |  |  |  |
| Ras-related protein Rab-10 | RAB10_HUMAN | 22755 | 8.59 |  |  |  |  |  |  |  |  |  | 76.93 | 11 | 2 |  |  |  |  |  |  |
| Ras-related protein Rab-13 | RAB13_HUMAN | 22988 | 9.27 |  |  |  |  |  |  |  |  |  | 68.83 | 9.4 | 2 |  |  |  |  |  |  |
| Ras-related protein Rab-15* | RAB15_HUMAN | 24660 | 5.53 |  |  |  |  |  |  |  |  |  | 63.93 | 9 | 2 |  |  |  |  |  |  |
| Ras-related protein Rab-1A | RAB1A_HUMAN | 22891 | 5.93 |  |  |  |  |  |  |  |  |  | 80.2 | 13.2 | 2 | 69.67 | 10.7 | 2 |  |  |  |
| Ras-related protein Rab-5B | RAB5B_HUMAN | 23920 | 8.30 | 62.19 | 11.6 | 2 |  |  |  |  |  |  |  |  |  |  |  |  |  |  |  |
| Ras-related protein Rab-6A | RAB6A_HUMAN | 23692 | 5.42 |  |  |  |  |  |  |  |  |  | 81.98 | 14.4 | 3 |  |  |  |  |  |  |
| Ras-related protein Rab-6B | RAB6B_HUMAN | 23561 | 5.42 |  |  |  |  |  |  | 74.36 | 16.3 | 3 | 84.03 | 15.9 | 3 |  |  |  |  |  |  |
| Ras-related protein Rap-1A | RAP1A_HUMAN | 21316 | 6.38 |  |  |  |  |  |  |  |  |  | 110.48 | 12.5 | 2 |  |  |  |  |  |  |
| Ras-related protein Rap-2a | RAP2A_HUMAN | 20830 | 4.74 |  |  |  |  |  |  |  |  |  | 244.07 | 26.8 | 5 |  |  |  |  |  |  |
| Ras-related protein Rap-2b | RAP2B_HUMAN | 20719 | 4.73 |  |  |  |  |  |  |  |  |  | 138 | 18 | 3 |  |  |  |  |  |  |
| RB1-inducible coiled-coil protein 1 | RBCC1_HUMAN | 185085 | 5.30 |  |  |  |  |  |  |  |  |  | 81.34 | 1.6 | 3 |  |  |  |  |  |  |
| Receptor-type tyrosine-protein phosphatase F | PTPRF_HUMAN | 213942 | 5.94 |  |  |  |  |  |  |  |  |  | 286.04 | 4.6 | 8 |  |  |  |  |  |  |
| Receptor-type tyrosine-protein phosphatase kappa* | PTPRK_HUMAN | 163994 | 5.58 |  |  |  |  |  |  |  |  |  | 58.13 | 0.8 | 2 |  |  |  |  |  |  |
| Receptor-type tyrosine-protein phosphatase S | PTPRS_HUMAN | 218159 | 6.07 |  |  |  |  |  |  |  |  |  | 186.58 | 3 | 4 |  |  |  |  |  |  |
| Receptor-type tyrosine-protein phosphatase zeta | PTPRZ_HUMAN | 255683 | 4.76 |  |  |  |  |  |  |  |  |  | 77.62 | 0.9 | 2 |  |  |  |  |  |  |
| Regulator of G-protein signaling 14* | RGS14_HUMAN | 61922 | 8.47 |  |  |  |  |  |  |  |  |  | 48.69 | 4.8 | 2 |  |  |  |  |  |  |
| Renin receptor | RENR_HUMAN | 38983 | 5.76 |  |  |  |  |  |  |  |  |  | 375.22 | 24 | 7 |  |  |  |  |  |  |
| Retinal dehydrogenase 1 | AL1A1_HUMAN | 55454 | 6.30 | 2081.58 | 76.4 | 31 | 1968.36 | 67.1 | 30 | 1910.38 | 68.7 | 29 | 2082.65 | 72.1 | 31 | 1827.32 | 68.7 | 29 | 1659.95 | 73.5 | 29 |
| Retinol-binding protein 1 | RET1_HUMAN | 16011 | 5.00 | 168.11 | 24.4 | 3 | 306.39 | 36.3 | 5 | 302.13 | 47.4 | 6 | 384.48 | 58.5 | 8 |  |  |  |  |  |  |
| Retinol-binding protein 3 | RET3_HUMAN | 135734 | 4.99 | 3011.92 | 64.7 | 52 | 3473.83 | 59 | 53 | 2830.16 | 57 | 48 | 2790.17 | 50.8 | 44 | 2624.83 | 62 | 43 | 1776.14 | 42.1 | 33 |
| Retinol-binding protein 4 | RET4_HUMAN | 23337 | 5.77 | 203.5 | 24.4 | 4 | 224.21 | 24.4 | 4 | 112.83 | 13.9 | 2 | 156.6 | 19.4 | 3 | 61.77 | 9 | 2 | 153.99 | 24.4 | 4 |
| Retinoschisin* | XLRS1_HUMAN | 26146 | 5.51 |  |  |  | 53.54 | 7.1 | 2 |  |  |  | 131.51 | 13.8 | 4 |  |  |  |  |  |  |
| Rho GDP-dissociation inhibitor 1 | GDIR1_HUMAN | 23250 | 5.03 | 135.45 | 18.6 | 3 | 108.43 | 13.7 | 3 | 79.17 | 10.8 | 2 | 257.81 | 27 | 6 |  |  |  |  |  |  |
| Rho GTPase-activating protein 4* | RHG04_HUMAN | 105816 | 5.97 |  |  |  |  |  |  |  |  |  | 56.18 | 1.7 | 2 |  |  |  |  |  |  |
| Rho GTPase-activating protein 8* | RHG08_HUMAN | 53735 | 9.45 | 49 | 4.1 | 2 |  |  |  |  |  |  |  |  |  |  |  |  |  |  |  |
| Rho guanine nucleotide exchange factor 15* | ARHGF_HUMAN | 92852 | 8.61 |  |  |  | 51.23 | 1.4 | 2 |  |  |  |  |  |  |  |  |  |  |  |  |
| Rho guanine nucleotide exchange factor 17* | ARHGH_HUMAN | 223645 | 5.90 |  |  |  |  |  |  |  |  |  | 51.66 | 1 | 2 |  |  |  |  |  |  |
| Rho guanine nucleotide exchange factor 18 | ARHGI_HUMAN | 131156 | 6.65 |  |  |  | 62.28 | 1.3 | 2 |  |  |  | 64.89 | 1.3 | 2 |  |  |  |  |  |  |
| Ribokinase | RBSK_HUMAN | 34577 | 4.94 |  |  |  | 65.84 | 5.6 | 2 |  |  |  | 132.56 | 8.7 | 3 |  |  |  |  |  |  |
| Ribonuclease III | CJ068_HUMAN | 159316 | 8.56 | 51.61 | 2.2 | 2 | 69.21 | 3.3 | 3 | 49.33 | 2.2 | 2 | 52.41 | 2.2 | 2 |  |  |  |  |  |  |
| Ribonuclease inhibitor | RINI_HUMAN | 51766 | 4.71 | 761.48 | 47.1 | 12 | 312.95 | 14.5 | 6 | 413.61 | 22.1 | 8 | 911.56 | 34.3 | 13 | 852.18 | 49 | 14 | 144.84 | 6.5 | 3 |
| Ribonuclease pancreatic | RNAS1_HUMAN | 18089 | 9.11 | 89.11 | 11.5 | 2 | 65.3 | 11.5 | 2 | 58.01 | 9.6 | 2 |  |  |  | 66.95 | 17.9 | 2 | 76.05 | 17.9 | 2 |
| Ribonuclease UK114 | UK114_HUMAN | 14542 | 8.74 | 321.39 | 42.3 | 5 | 247.21 | 45.3 | 4 |  |  |  |  |  |  |  |  |  |  |  |  |
| Ribose-phosphate pyrophosphokinase 1 | PRPS1_HUMAN | 35325 | 6.52 | 179.73 | 15.1 | 4 | 422.33 | 26.7 | 8 |  |  |  | 395.57 | 24.5 | 7 |  |  |  |  |  |  |
| Ribosomal L1 domain-containing protein 1* | RL1D1_HUMAN | 55167 | 10.13 |  |  |  |  |  |  |  |  |  | 49.05 | 3.1 | 2 |  |  |  |  |  |  |
| RING finger protein 207* | RN207_HUMAN | 72184 | 6.22 |  |  |  |  |  |  |  |  |  | 54.35 | 2.1 | 2 |  |  |  |  |  |  |
| RNA 3'-terminal phosphate cyclase* | RTC1_HUMAN | 39825 | 8.01 |  |  |  |  |  |  |  |  |  | 71.52 | 6.6 | 2 |  |  |  |  |  |  |
| Rootletin | CROCC_HUMAN | 228787 | 5.45 |  |  |  |  |  |  |  |  |  | 196.09 | 3 | 7 |  |  |  |  |  |  |
| Rotatin | RTTN_HUMAN | 252290 | 6.26 |  |  |  | 64.49 | 0.6 | 2 | 60.52 | 0.6 | 2 | 57.93 | 0.6 | 2 | 50.85 | 0.7 | 2 |  |  |  |
| Ryanodine receptor 3 | RYR3_HUMAN | 557790 | 5.47 |  |  |  |  |  |  |  |  |  | 72.54 | 0.4 | 3 |  |  |  |  |  |  |
| Sacsin | SACS_HUMAN | 526497 | 6.63 |  |  |  |  |  |  | 71.98 | 0.6 | 3 | 87.48 | 0.8 | 4 |  |  |  |  |  |  |
| SAGA-associated factor 29 homolog* | SGF29_HUMAN | 33445 | 8.20 | 47.6 | 4.1 | 2 |  |  |  |  |  |  |  |  |  |  |  |  |  |  |  |
| Sarcosine dehydrogenase. mitochondrial* | SARDH_HUMAN | 101942 | 6.80 | 58.96 | 2.7 | 2 |  |  |  |  |  |  |  |  |  |  |  |  | 53.26 | 2.7 | 2 |
| SCY1-like protein 2* | SCYL2_HUMAN | 104327 | 8.45 | 54.54 | 1.7 | 2 |  |  |  |  |  |  |  |  |  |  |  |  |  |  |  |
| SEC14-like protein 2* | S14L2_HUMAN | 46629 | 7.95 |  |  |  |  |  |  |  |  |  | 69.15 | 4 | 2 |  |  |  |  |  |  |
| Secernin-1 | SCRN1_HUMAN | 46980 | 4.67 | 119.95 | 8.7 | 3 |  |  |  |  |  |  | 468.69 | 18.4 | 7 | 281.23 | 12.8 | 4 |  |  |  |
| Secernin-2 | SCRN2_HUMAN | 47023 | 5.44 |  |  |  |  |  |  |  |  |  | 211.22 | 12.5 | 4 |  |  |  |  |  |  |
| Secreted frizzled-related protein 3 | SFRP3_HUMAN | 37257 | 8.81 |  |  |  |  |  |  | 55.82 | 5.8 | 2 | 67.99 | 5.8 | 2 |  |  |  |  |  |  |
| Secretoglobin family 1D member 2 | SG1D2_HUMAN | 10260 | 8.58 |  |  |  |  |  |  |  |  |  | 95.69 | 20 | 2 |  |  |  |  |  |  |
| Secretogranin-3 | SCG3_HUMAN | 52973 | 4.95 |  |  |  |  |  |  |  |  |  | 115.13 | 8.1 | 3 |  |  |  |  |  |  |
| Seizure protein 6 homolog | SEZ6_HUMAN | 108840 | 5.16 | 271.76 | 10 | 6 | 256.48 | 6.4 | 6 | 234.73 | 7.3 | 6 | 234.39 | 5.9 | 5 | 163.71 | 4 | 3 |  |  |  |
| Semaphorin-4B | SEM4B_HUMAN | 93503 | 6.51 |  |  |  |  |  |  |  |  |  | 153.04 | 6.1 | 5 |  |  |  |  |  |  |
| Semaphorin-7A | SEM7A_HUMAN | 75860 | 7.58 | 197.79 | 8.4 | 5 | 245.96 | 11.1 | 6 | 149.07 | 5 | 3 | 374.53 | 17.1 | 10 |  |  |  |  |  |  |
| Serine hydroxymethyltransferase. cytosolic | GLYC_HUMAN | 53619 | 7.61 | 117.52 | 7.2 | 3 |  |  |  |  |  |  | 147.52 | 4.3 | 2 |  |  |  |  |  |  |
| Serine/threonine-protein kinase SMG1* | SMG1_HUMAN | 414347 | 6.03 | 47.83 | 0.4 | 2 |  |  |  |  |  |  |  |  |  |  |  |  |  |  |  |
| Serine/threonine-protein kinase TAO3 | TAOK3_HUMAN | 105796 | 6.83 |  |  |  |  |  |  |  |  |  | 71.28 | 3 | 3 |  |  |  |  |  |  |
| Serine/threonine-protein kinase WNK1* | WNK1_HUMAN | 251552 | 5.94 |  |  |  |  |  |  | 46.46 | 0.6 | 2 |  |  |  |  |  |  |  |  |  |
| Serine/threonine-protein phosphatase 2A activator | PTPA_HUMAN | 41098 | 5.64 | 205.23 | 21.2 | 5 | 105.26 | 7.8 | 3 |  |  |  | 171.59 | 14.5 | 5 |  |  |  |  |  |  |
| Serine/threonine-protein phosphatase PP1-beta catalytic subunit | PP1B_HUMAN | 37961 | 5.84 |  |  |  |  |  |  |  |  |  | 194.9 | 11 | 3 |  |  |  |  |  |  |
| Serine-protein kinase ATM | ATM_HUMAN | 355564 | 6.39 |  |  |  |  |  |  |  |  |  | 82.76 | 0.8 | 3 |  |  |  |  |  |  |
| Serotransferrin | TRFE_HUMAN | 79294 | 6.81 | 3876.16 | 82.2 | 57 | 4606.46 | 77.2 | 66 | 3817.99 | 69.9 | 53 | 4238.12 | 72.5 | 59 | 3151.77 | 68.5 | 47 | 3279.79 | 71.9 | 49 |
| Serpin B12 | SPB12_HUMAN | 46646 | 5.37 |  |  |  | 95.33 | 6.2 | 2 |  |  |  | 272.74 | 16.5 | 6 | 108.75 | 7.9 | 2 |  |  |  |
| Serpin B3 | SPB3_HUMAN | 44594 | 6.35 |  |  |  | 139.85 | 11 | 3 | 165.77 | 17.2 | 5 | 671.59 | 39 | 15 | 96.78 | 5.1 | 2 |  |  |  |
| Serpin B6 | SPB6_HUMAN | 42936 | 5.19 | 1330.99 | 66.5 | 22 | 1211.96 | 60.9 | 19 | 1210.09 | 61.2 | 19 | 1638.78 | 62.8 | 23 | 1395.53 | 69.1 | 21 | 685.63 | 50.3 | 16 |
| Serpin B8 | SPB8_HUMAN | 43309 | 5.41 |  |  |  |  |  |  |  |  |  | 185.58 | 10.4 | 4 |  |  |  |  |  |  |
| Serpin B9 | SPB9_HUMAN | 43004 | 5.61 | 768.03 | 45.5 | 16 | 694.59 | 41.5 | 15 | 367.59 | 25.8 | 9 | 1183.58 | 61.7 | 22 | 393.98 | 25.8 | 8 | 331.98 | 19.7 | 7 |
| Serpin E3 | SERP3_HUMAN | 47332 | 6.70 |  |  |  |  |  |  |  |  |  | 107.99 | 8.3 | 3 |  |  |  |  |  |  |
| Serum albumin | ALBU_HUMAN | 71317 | 5.93 | 4166.37 | 83.1 | 62 | 4744.14 | 88.2 | 70 | 4163.46 | 85.6 | 63 | 4065.44 | 85.2 | 62 | 3872.84 | 82.8 | 58 | 3923.46 | 82.8 | 62 |
| Serum amyloid A-4 protein | SAA4_HUMAN | 14851 | 9.17 | 171.76 | 26.2 | 4 | 124.87 | 27.7 | 4 | 55.59 | 15.4 | 2 |  |  |  |  |  |  |  |  |  |
| Serum paraoxonase/arylesterase 1 | PON1_HUMAN | 39877 | 5.09 | 217.45 | 16.6 | 5 | 187.19 | 14.9 | 5 | 77.37 | 5.6 | 2 | 613.98 | 43.1 | 11 | 140.31 | 17.7 | 4 |  |  |  |
| SET and MYND domain-containing protein 5 | SMYD5_HUMAN | 48564 | 4.99 | 86.69 | 5 | 2 |  |  |  |  |  |  |  |  |  |  |  |  |  |  |  |
| Sex hormone-binding globulin | SHBG_HUMAN | 43980 | 6.23 |  |  |  |  |  |  | 82.91 | 5.7 | 2 | 164.15 | 11.7 | 3 |  |  |  |  |  |  |
| S-formylglutathione hydrolase | ESTD_HUMAN | 31956 | 6.54 | 386.84 | 41.8 | 8 | 611.77 | 61.3 | 13 | 682 | 59.6 | 13 | 493.55 | 45.7 | 10 |  |  |  |  |  |  |
| Sister chromatid cohesion protein PDS5 homolog B | PDS5B_HUMAN | 165818 | 8.67 | 67.11 | 0.8 | 2 | 53.05 | 0.8 | 2 | 77.39 | 0.8 | 2 | 64.6 | 0.8 | 2 |  |  |  |  |  |  |
| Skin-specific protein 32 | XP32_HUMAN | 28557 | 8.41 |  |  |  |  |  |  |  |  |  | 188.77 | 23.2 | 6 | 100.09 | 12.4 | 3 |  |  |  |
| Small proline-rich protein 2D | SPR2D_HUMAN | 8584 | 8.77 |  |  |  |  |  |  |  |  |  | 105.68 | 30.6 | 2 |  |  |  |  |  |  |
| Small proline-rich protein 3 | SPRR3_HUMAN | 18598 | 8.87 |  |  |  | 63.2 | 9.5 | 2 |  |  |  | 162.69 | 28.4 | 5 |  |  |  |  |  |  |
| S-methyl-5'-thioadenosine phosphorylase | MTAP_HUMAN | 31729 | 6.76 |  |  |  |  |  |  |  |  |  | 87.77 | 9.5 | 2 |  |  |  |  |  |  |
| Son of sevenless homolog 2 | SOS2_HUMAN | 154251 | 6.39 |  |  |  |  |  |  |  |  |  | 65.67 | 1.5 | 3 |  |  |  |  |  |  |
| Sorbitol dehydrogenase | DHSO_HUMAN | 38927 | 8.23 | 1086 | 66.9 | 19 | 1149.9 | 64.7 | 18 | 1017.56 | 58.5 | 16 | 986.19 | 53.2 | 18 | 842.69 | 47.9 | 14 | 828.75 | 54.9 | 15 |
| Sorting nexin-29 | SNX29_HUMAN | 91596 | 5.86 |  |  |  | 68.86 | 2 | 2 |  |  |  | 72.64 | 2.6 | 2 |  |  |  |  |  |  |
| SPARC-like protein 1 | SPRL1_HUMAN | 76017 | 4.71 | 562.2 | 25.3 | 11 | 325.52 | 13.4 | 7 |  |  |  | 59.13 | 3.9 | 2 | 351.49 | 16.1 | 7 |  |  |  |
| Spatacsin | SPTCS_HUMAN | 282681 | 5.63 |  |  |  |  |  |  |  |  |  | 67.46 | 1.1 | 3 |  |  |  |  |  |  |
| SPATS2-like protein* | SPS2L_HUMAN | 62204 | 9.68 |  |  |  |  |  |  |  |  |  | 48.73 | 5 | 2 |  |  |  |  |  |  |
| Spectrin alpha chain. brain | SPTA2_HUMAN | 285163 | 5.22 | 6406.29 | 52.5 | 116 | 4023 | 31.4 | 78 | 3379.36 | 29 | 65 | 6395.1 | 50 | 117 | 1529.95 | 16.7 | 32 | 981.09 | 11.3 | 23 |
| Spectrin beta chain. brain 1 | SPTB2_HUMAN | 275237 | 5.40 | 5513.93 | 52.1 | 105 | 2816.52 | 24.7 | 57 | 2904.02 | 28.6 | 59 | 2756.86 | 29.3 | 62 | 1445.9 | 16.3 | 27 | 643.9 | 8.1 | 15 |
| Spectrin beta chain. brain 2 | SPTN2_HUMAN | 272526 | 5.80 | 334.86 | 4.3 | 10 |  |  |  |  |  |  | 135.37 | 1.2 | 3 |  |  |  |  |  |  |
| Spectrin beta chain. brain 4 | SPTN4_HUMAN | 290005 | 5.72 |  |  |  |  |  |  |  |  |  | 107.65 | 1.1 | 3 |  |  |  |  |  |  |
| Spectrin beta chain. erythrocyte | SPTB1_HUMAN | 247171 | 5.15 |  |  |  | 213.68 | 2.2 | 6 |  |  |  |  |  |  |  |  |  |  |  |  |
| Spondin-1 | SPON1_HUMAN | 93537 | 5.86 | 291.4 | 12.5 | 7 | 532.5 | 19.7 | 12 | 174.94 | 7.7 | 5 | 981.66 | 29.1 | 19 |  |  |  | 75.17 | 2.7 | 2 |
| Sterol regulatory element-binding protein 1 | SRBP1_HUMAN | 122625 | 8.44 |  |  |  |  |  |  |  |  |  | 56.54 | 1.5 | 2 |  |  |  |  |  |  |
| Stress-70 protein. mitochondrial | GRP75_HUMAN | 73920 | 5.87 |  |  |  |  |  |  |  |  |  | 163.18 | 6 | 4 |  |  |  |  |  |  |
| Stress-induced-phosphoprotein 1 | STIP1_HUMAN | 63227 | 6.40 | 122.87 | 4.8 | 2 |  |  |  |  |  |  | 118.38 | 5.2 | 3 |  |  |  |  |  |  |
| Structural maintenance of chromosomes flexible hinge domain-containing protein 1 | SMHD1_HUMAN | 227942 | 6.95 |  |  |  |  |  |  |  |  |  | 67.43 | 1.2 | 3 |  |  |  |  |  |  |
| Structural maintenance of chromosomes protein 2* | SMC2_HUMAN | 136085 | 8.54 |  |  |  |  |  |  |  |  |  | 49.61 | 1.1 | 2 |  |  |  |  |  |  |
| Structural maintenance of chromosomes protein 4 | SMC4_HUMAN | 147775 | 6.37 | 96.34 | 1.6 | 3 |  |  |  |  |  |  |  |  |  |  |  |  |  |  |  |
| Sulfhydryl oxidase 1 | QSOX1_HUMAN | 83324 | 9.14 |  |  |  |  |  |  | 178.7 | 8.3 | 4 | 324.47 | 13.7 | 8 |  |  |  |  |  |  |
| Superoxide dismutase [Cu-Zn] | SODC_HUMAN | 16154 | 5.71 |  |  |  | 298.03 | 53.9 | 5 | 172.77 | 32.5 | 3 |  |  |  | 184.07 | 58.4 | 4 |  |  |  |
| Suppression of tumorigenicity 5 protein | ST5_HUMAN | 127603 | 9.36 |  |  |  |  |  |  |  |  |  | 84.34 | 2.3 | 3 |  |  |  |  |  |  |
| Suppressor of cytokine signaling 6* | SOCS6_HUMAN | 59947 | 6.80 |  |  |  |  |  |  |  |  |  | 51.22 | 2.8 | 2 |  |  |  |  |  |  |
| Synaptonemal complex protein 1 | SYCP1_HUMAN | 114748 | 5.78 |  |  |  |  |  |  |  |  |  | 75.75 | 3.3 | 3 |  |  |  |  |  |  |
| Talin-1 | TLN1_HUMAN | 271766 | 5.78 | 63.3 | 0.8 | 2 |  |  |  |  |  |  | 53.8 | 0.5 | 2 |  |  |  |  |  |  |
| Target of Nesh-SH3 | TARSH_HUMAN | 119253 | 9.48 | 260.21 | 7.8 | 6 | 274.22 | 5.5 | 6 | 225.68 | 4.7 | 5 |  |  |  |  |  |  |  |  |  |
| Taste receptor type 2 member 10* | T2R10_HUMAN | 35684 | 9.55 |  |  |  |  |  |  |  |  |  | 48.34 | 6.2 | 2 |  |  |  |  |  |  |
| TBC1 domain family member 1 | TBCD1_HUMAN | 134311 | 6.53 |  |  |  |  |  |  |  |  |  | 65.19 | 1.8 | 3 |  |  |  |  |  |  |
| TBC1 domain family member 4 | TBCD4_HUMAN | 148068 | 6.57 |  |  |  |  |  |  |  |  |  | 90.13 | 2.2 | 4 |  |  |  |  |  |  |
| T-complex protein 1 subunit alpha | TCPA_HUMAN | 60819 | 5.80 |  |  |  |  |  |  |  |  |  | 252.52 | 11 | 6 | 82.1 | 5.6 | 3 |  |  |  |
| T-complex protein 1 subunit beta | TCPB_HUMAN | 57794 | 6.01 |  |  |  | 67.15 | 3.4 | 2 |  |  |  | 426.69 | 20.7 | 9 |  |  |  |  |  |  |
| T-complex protein 1 subunit delta | TCPD_HUMAN | 58401 | 7.97 | 229.68 | 10.8 | 5 | 97.86 | 5.6 | 3 | 69.87 | 4.1 | 2 | 286.11 | 11.9 | 6 |  |  |  |  |  |  |
| T-complex protein 1 subunit epsilon | TCPE_HUMAN | 60089 | 5.45 |  |  |  |  |  |  |  |  |  | 272.18 | 10.5 | 5 | 272.5 | 12.2 | 6 |  |  |  |
| T-complex protein 1 subunit eta | TCPH_HUMAN | 59842 | 7.55 |  |  |  |  |  |  | 57.26 | 2.9 | 2 | 91.8 | 2.9 | 2 |  |  |  |  |  |  |
| T-complex protein 1 subunit gamma | TCPG_HUMAN | 61066 | 6.10 |  |  |  | 84.58 | 4 | 2 | 87.71 | 4 | 2 | 225.18 | 10.6 | 6 | 61.46 | 3.9 | 2 | 72.97 | 3.9 | 2 |
| T-complex protein 1 subunit theta | TCPQ_HUMAN | 60153 | 5.42 |  |  |  |  |  |  |  |  |  | 157.22 | 11.3 | 6 |  |  |  |  |  |  |
| T-complex protein 1 subunit zeta | TCPZ_HUMAN | 58444 | 6.23 |  |  |  |  |  |  |  |  |  | 127.76 | 8.7 | 4 |  |  |  |  |  |  |
| Telomere-associated protein RIF1 | RIF1_HUMAN | 276461 | 5.39 |  |  |  | 52.2 | 0.6 | 2 | 82.51 | 0.9 | 3 | 100.44 | 1.3 | 4 |  |  |  |  |  |  |
| Tenascin | TENA_HUMAN | 246345 | 4.80 | 131.21 | 1.4 | 3 | 98.98 | 0.9 | 2 |  |  |  |  |  |  |  |  |  |  |  |  |
| Teneurin-4 | TEN4_HUMAN | 312380 | 6.10 |  |  |  |  |  |  |  |  |  | 60.64 | 0.8 | 2 |  |  |  |  |  |  |
| Tensin-1* | TENS1_HUMAN | 186499 | 7.55 |  |  |  |  |  |  |  |  |  | 47.22 | 1.6 | 2 |  |  |  |  |  |  |
| Testican-1 | TICN1_HUMAN | 50518 | 5.74 |  |  |  | 70.29 | 4.1 | 2 |  |  |  | 112.66 | 6.4 | 3 |  |  |  |  |  |  |
| Thioredoxin | THIO_HUMAN | 12015 | 4.83 | 148.78 | 31.4 | 3 | 177.28 | 31.4 | 3 | 179.77 | 31.4 | 3 | 195.31 | 31.4 | 3 | 135.9 | 23.8 | 2 | 123.75 | 21 | 2 |
| Thioredoxin-like protein 1 | TXNL1_HUMAN | 32630 | 4.84 |  |  |  |  |  |  |  |  |  | 105.63 | 13.1 | 3 |  |  |  |  |  |  |
| Thyroid receptor-interacting protein 11 | TRIPB_HUMAN | 228131 | 5.18 |  |  |  |  |  |  |  |  |  | 54.33 | 0.8 | 2 |  |  |  |  |  |  |
| Thyroxine-binding globulin | THBG_HUMAN | 46637 | 5.88 | 162.29 | 15.2 | 4 | 213.8 | 17.1 | 6 | 336.72 | 19.3 | 8 | 878.62 | 47.2 | 16 | 108.56 | 11.1 | 3 | 186.26 | 14.9 | 4 |
| TIP41-like protein | TIPRL_HUMAN | 31652 | 5.60 |  |  |  |  |  |  |  |  |  | 173.75 | 22.1 | 5 |  |  |  |  |  |  |
| Titin | TITIN_HUMAN | 3843072 | 6.01 | 208.51 | 0.3 | 9 | 156.24 | 0.2 | 7 | 73.19 | 0.1 | 3 | 359.82 | 0.3 | 14 | 103.16 | 0.1 | 4 | 63.15 | 0.1 | 2 |
| TRAF2 and NCK-interacting protein kinase* | TNIK_HUMAN | 155361 | 6.71 |  |  |  |  |  |  |  |  |  | 51.93 | 1.1 | 2 |  |  |  |  |  |  |
| Transaldolase | TALDO_HUMAN | 37688 | 6.37 | 464.18 | 32 | 10 | 310.82 | 19 | 7 |  |  |  | 621.01 | 38.9 | 14 |  |  |  |  |  |  |
| Transcription factor HIVEP3 | ZEP3_HUMAN | 261129 | 7.85 |  |  |  |  |  |  | 50.83 | 0.7 | 2 |  |  |  |  |  |  |  |  |  |
| Transcription initiation factor TFIID subunit 1-like | TAF1L_HUMAN | 208711 | 5.26 |  |  |  |  |  |  | 56.37 | 0.9 | 2 | 78.22 | 1.3 | 3 |  |  |  |  |  |  |
| Transcriptional activator GLI3 | GLI3_HUMAN | 171410 | 7.03 |  |  |  |  |  |  |  |  |  | 51.85 | 0.9 | 2 |  |  |  |  |  |  |
| Transforming protein RhoA | RHOA_HUMAN | 22096 | 5.84 | 117.74 | 17.6 | 3 |  |  |  |  |  |  |  |  |  |  |  |  |  |  |  |
| Transitional endoplasmic reticulum ATPase | TERA_HUMAN | 89950 | 5.14 | 275.43 | 10 | 6 | 248.74 | 7.1 | 4 |  |  |  | 999.82 | 28.5 | 20 |  |  |  |  |  |  |
| Transketolase | TKT_HUMAN | 68519 | 7.58 | 1541.8 | 46.5 | 27 | 849.36 | 33.9 | 17 | 566.73 | 25.8 | 13 | 1149.06 | 48.8 | 26 | 298.5 | 16.5 | 7 | 483.06 | 22.8 | 12 |
| Translational activator GCN1* | GCN1L_HUMAN | 294967 | 7.29 |  |  |  | 56.31 | 0.6 | 2 |  |  |  |  |  |  |  |  |  |  |  |  |
| Translationally-controlled tumor protein | TCTP_HUMAN | 19697 | 4.84 |  |  |  |  |  |  |  |  |  | 108.13 | 19.8 | 3 | 109.76 | 15.7 | 2 |  |  |  |
| Translin-associated factor X-interacting protein 1* | TXIP1_HUMAN | 77010 | 4.99 |  |  |  | 50.73 | 2.3 | 2 |  |  |  |  |  |  |  |  |  |  |  |  |
| Translin-associated protein X* | TSNAX_HUMAN | 33206 | 6.11 |  |  |  |  |  |  |  |  |  | 91.24 | 9.7 | 2 |  |  |  |  |  |  |
| Transmembrane protein 132A | T132A_HUMAN | 111011 | 5.43 |  |  |  |  |  |  |  |  |  | 220.25 | 9.6 | 7 |  |  |  |  |  |  |
| Transmembrane protein 14C | TM14C_HUMAN | 11557 | 9.87 | 137.97 | 50 | 3 |  |  |  | 92.77 | 41.1 | 2 |  |  |  |  |  |  |  |  |  |
| Transportin-1* | TNPO1_HUMAN | 103771 | 4.84 | 58.39 | 2 | 2 |  |  |  |  |  |  |  |  |  |  |  |  |  |  |  |
| Transportin-2 | TNPO2_HUMAN | 102862 | 4.87 |  |  |  | 238.98 | 5.4 | 5 |  |  |  |  |  |  |  |  |  |  |  |  |
| Transthyretin | TTHY_HUMAN | 15991 | 5.52 | 802.39 | 73.5 | 11 | 1058.52 | 77.6 | 14 | 956.7 | 73.5 | 12 | 1010.2 | 73.5 | 12 | 815.54 | 69.4 | 10 | 684.59 | 73.5 | 10 |
| Triosephosphate isomerase | TPIS_HUMAN | 31057 | 5.66 | 1047.13 | 71 | 15 | 1022.48 | 66.4 | 14 | 792.5 | 68.2 | 14 | 999.93 | 67.5 | 16 | 294.21 | 25.5 | 6 | 435.59 | 42.3 | 8 |
| Tripartite motif-containing protein 29* | TRI29_HUMAN | 66478 | 6.73 |  |  |  |  |  |  |  |  |  | 51.37 | 2 | 2 |  |  |  |  |  |  |
| Tripeptidyl-peptidase 1 | TPP1_HUMAN | 61723 | 6.02 |  |  |  | 140.12 | 8 | 2 | 181.83 | 13 | 4 | 310.34 | 17.8 | 5 |  |  |  |  |  |  |
| Tropomyosin alpha-3 chain | TPM3_HUMAN | 32856 | 4.69 |  |  |  |  |  |  | 62 | 8.8 | 2 | 583.64 | 35.9 | 13 | 258.42 | 21.1 | 5 |  |  |  |
| Tropomyosin alpha-4 chain | TPM4_HUMAN | 28619 | 4.67 |  |  |  |  |  |  |  |  |  | 234.06 | 17.7 | 6 |  |  |  |  |  |  |
| Trypsin-1 | TRY1_HUMAN | 27111 | 6.08 | 92.16 | 6.5 | 2 |  |  |  |  |  |  |  |  |  |  |  |  |  |  |  |
| Trypsin-3* | TRY3_HUMAN | 33306 | 7.46 |  |  |  |  |  |  |  |  |  | 87.8 | 6.6 | 2 |  |  |  |  |  |  |
| Tubulin alpha-1A chain | TBA1A_HUMAN | 50788 | 4.95 | 600.12 | 34.6 | 11 | 663.53 | 33.9 | 14 | 786.58 | 40.6 | 14 | 925.79 | 41 | 14 | 671.8 | 34.8 | 11 | 450.57 | 31.7 | 11 |
| Tubulin alpha-1B chain | TBA1B_HUMAN | 50804 | 4.95 | 579.31 | 34.6 | 11 |  |  |  | 739.31 | 40.6 | 14 |  |  |  | 655.25 | 34.8 | 11 |  |  |  |
| Tubulin alpha-1C chain | TBA1C_HUMAN | 50548 | 4.96 |  |  |  | 637.58 | 34.1 | 14 |  |  |  | 897.48 | 41.2 | 14 | 659.37 | 35 | 11 |  |  |  |
| Tubulin alpha-8 chain | TBA8_HUMAN | 50746 | 4.94 |  |  |  |  |  |  |  |  |  | 523.22 | 27.6 | 9 |  |  |  |  |  |  |
| Tubulin beta chain | TBB5_HUMAN | 50095 | 4.78 | 251.23 | 22.1 | 6 | 288.22 | 14.4 | 5 | 289.58 | 15.5 | 5 | 660.34 | 34.9 | 12 |  |  |  |  |  |  |
| Tubulin beta-3 chain | TBB3_HUMAN | 50500 | 4.67 |  |  |  |  |  |  |  |  |  | 348.22 | 22.7 | 8 |  |  |  |  |  |  |
| Tubulin Beta-4B chain | TBB4B_HUMAN | 50255 | 4.63 |  |  |  |  |  |  |  |  |  | 513.87 | 26.9 | 10 |  |  |  |  |  |  |
| Tubulin-folding cofactor B | TBCB_HUMAN | 27594 | 5.06 |  |  |  | 86.37 | 7.8 | 2 | 70.44 | 7.8 | 2 | 290.04 | 26.6 | 6 | 148.59 | 11.1 | 3 |  |  |  |
| Tudor domain-containing protein 1* | TDRD1_HUMAN | 134277 | 5.97 | 46.78 | 1.6 | 2 |  |  |  |  |  |  |  |  |  |  |  |  |  |  |  |
| Tudor domain-containing protein 5* | TDRD5_HUMAN | 111036 | 8.31 |  |  |  |  |  |  |  |  |  | 56.14 | 1.2 | 2 |  |  |  |  |  |  |
| Ubiquitin carboxyl-terminal hydrolase 5* | UBP5_HUMAN | 96638 | 4.92 | 56.16 | 2.3 | 2 |  |  |  |  |  |  |  |  |  |  |  |  |  |  |  |
| Ubiquitin carboxyl-terminal hydrolase isozyme L1 | UCHL1_HUMAN | 25151 | 5.33 | 473.99 | 49.8 | 8 | 644.43 | 74 | 12 | 619.99 | 69.5 | 14 | 688.84 | 80.7 | 14 | 562.09 | 52.5 | 10 | 357.27 | 53.4 | 8 |
| Ubiquitin carboxyl-terminal hydrolase isozyme L3 | UCHL3_HUMAN | 26337 | 4.84 |  |  |  |  |  |  |  |  |  | 360.05 | 44.3 | 7 | 215.41 | 21.7 | 3 |  |  |  |
| Ubiquitin fusion degradation protein 1 homolog | UFD1_HUMAN | 34763 | 6.27 |  |  |  |  |  |  |  |  |  | 60.14 | 6.8 | 2 |  |  |  |  |  |  |
| Ubiquitin thioesterase OTUB1 | OTUB1_HUMAN | 31492 | 4.85 |  |  |  |  |  |  |  |  |  | 420.39 | 32.1 | 7 | 127.55 | 9.2 | 2 |  |  |  |
| Ubiquitin/ISG15-conjugating enzyme E2 L6* | UB2L6_HUMAN | 17928 | 7.71 |  |  |  |  |  |  |  |  |  | 48.93 | 5.9 | 2 |  |  |  |  |  |  |
| Ubiquitin-40S ribosomal protein S27a | RS27A_HUMAN | 18296 | 9.68 | 213.42 | 34 | 6 | 190.3 | 25.6 | 4 | 206.92 | 25.6 | 4 | 232.1 | 26.9 | 5 |  |  |  | 187.25 | 25.6 | 4 |
| Ubiquitin-conjugating enzyme E2 N | UBE2N_HUMAN | 17184 | 6.14 |  |  |  |  |  |  | 161.81 | 35.5 | 4 |  |  |  |  |  |  |  |  |  |
| Ubiquitin-conjugating enzyme E2 variant 1 | UB2V1_HUMAN | 16598 | 7.72 |  |  |  |  |  |  | 96.91 | 15.6 | 3 |  |  |  |  |  |  |  |  |  |
| Ubiquitin-like modifier-activating enzyme 1 | UBA1_HUMAN | 118858 | 5.50 | 648.3 | 18.8 | 13 | 298.3 | 7.8 | 6 |  |  |  | 1477.36 | 34.6 | 27 |  |  |  |  |  |  |
| Ubiquitin-like modifier-activating enzyme 6* | UBA6_HUMAN | 119207 | 5.76 |  |  |  |  |  |  |  |  |  | 65.52 | 1.7 | 2 |  |  |  |  |  |  |
| Ubiquitin-protein ligase E3B | UBE3B_HUMAN | 124502 | 8.51 | 67.41 | 1.2 | 2 |  |  |  |  |  |  |  |  |  |  |  |  |  |  |  |
| UDP-glucose 4-epimerase | GALE_HUMAN | 38656 | 6.27 |  |  |  |  |  |  |  |  |  | 177.47 | 14.7 | 4 |  |  |  |  |  |  |
| UDP-glucose:glycoprotein glucosyltransferase 2 | UGGG2_HUMAN | 175251 | 6.44 |  |  |  |  |  |  |  |  |  | 65.53 | 1.3 | 3 |  |  |  |  |  |  |
| Unconventional myosin-Ia* | MYO1A_HUMAN | 119238 | 9.37 |  |  |  | 52.59 | 1.2 | 2 | 56.94 | 1.2 | 2 | 55.45 | 1.2 | 2 |  |  |  |  |  |  |
| Unconventional myosin-Ie | MYO1E_HUMAN | 127552 | 9.02 |  |  |  |  |  |  |  |  |  | 70.39 | 2.3 | 3 |  |  |  |  |  |  |
| Unconventional myosin-IXb | MYO9B_HUMAN | 244846 | 8.91 |  |  |  |  |  |  |  |  |  | 78.25 | 0.9 | 3 |  |  |  |  |  |  |
| Unconventional myosin-Vb | MYO5B_HUMAN | 215135 | 6.77 |  |  |  |  |  |  |  |  |  | 95.25 | 0.9 | 3 |  |  |  |  |  |  |
| Unconventional myosin-Vc | MYO5C_HUMAN | 203994 | 7.69 | 111.38 | 1.8 | 4 |  |  |  |  |  |  | 116.03 | 1.8 | 4 |  |  |  |  |  |  |
| Unconventional myosin-XVIIIa | MY18A_HUMAN | 234168 | 5.96 |  |  |  |  |  |  | 51.44 | 0.8 | 2 | 97.43 | 1.5 | 4 |  |  |  |  |  |  |
| Unconventional myosin-XVIIIb | MY18B_HUMAN | 287175 | 6.49 | 78.45 | 0.9 | 3 |  |  |  |  |  |  |  |  |  |  |  |  |  |  |  |
| Unconventional prefoldin RPB5 interactor 1* | RMP_HUMAN | 60252 | 4.92 |  |  |  |  |  |  |  |  |  | 59.72 | 2.8 | 2 |  |  |  |  |  |  |
| UPF0553 protein C9orf64 | CI064_HUMAN | 39460 | 5.61 |  |  |  |  |  |  |  |  |  | 69.83 | 7.3 | 2 |  |  |  |  |  |  |
| UTP--glucose-1-phosphate uridylyltransferase | UGPA_HUMAN | 57076 | 8.17 | 199.46 | 11 | 5 |  |  |  |  |  |  | 359.53 | 18.7 | 8 |  |  |  |  |  |  |
| Utrophin | UTRO_HUMAN | 396444 | 5.20 |  |  |  |  |  |  |  |  |  | 76.29 | 0.8 | 3 |  |  |  |  |  |  |
| Vacuolar protein sorting-associated protein 13C | VP13C_HUMAN | 424462 | 6.39 | 89.97 | 0.8 | 4 |  |  |  |  |  |  | 89.12 | 0.8 | 4 |  |  |  |  |  |  |
| Vacuolar protein sorting-associated protein 13D* | VP13D_HUMAN | 495298 | 6.15 |  |  |  |  |  |  |  |  |  | 50 | 0.4 | 2 |  |  |  |  |  |  |
| Vasorin | VASN_HUMAN | 72751 | 7.17 |  |  |  | 168.72 | 5.6 | 3 | 304.08 | 11.9 | 6 | 395.31 | 12.9 | 7 | 259.86 | 8.3 | 4 |  |  |  |
| Versican core protein | CSPG2_HUMAN | 374585 | 4.43 | 165.19 | 1.8 | 5 |  |  |  | 264.55 | 1.8 | 5 | 437.18 | 2.3 | 7 | 476.02 | 3.5 | 9 | 49.83 | 0.7 | 2 |
| Vimentin | VIME_HUMAN | 53676 | 5.06 | 183.5 | 10.1 | 5 | 829.69 | 35.6 | 17 | 295.44 | 13.7 | 6 | 1906.02 | 69.5 | 33 | 985.5 | 40.6 | 20 | 563.67 | 25.5 | 12 |
| Vinculin | VINC_HUMAN | 124292 | 5.51 | 212.59 | 6.3 | 5 |  |  |  |  |  |  | 661.26 | 17.3 | 16 |  |  |  |  |  |  |
| Vitamin D-binding protein | VTDB_HUMAN | 54526 | 5.40 | 1627.62 | 70 | 29 | 1715.83 | 63.3 | 30 | 1706.43 | 73.4 | 29 | 1446.72 | 60.3 | 27 | 827.46 | 39.5 | 17 | 1136.13 | 62.7 | 20 |
| Vitamin K-dependent protein S | PROS_HUMAN | 77127 | 5.48 |  |  |  | 72.24 | 2.1 | 2 |  |  |  | 393.82 | 11.4 | 9 |  |  |  |  |  |  |
| Vitronectin | VTNC_HUMAN | 55069 | 5.55 | 227.91 | 13 | 4 | 240.85 | 14.6 | 6 | 306.89 | 17.8 | 6 | 333.36 | 14.9 | 7 | 428.77 | 24.3 | 8 | 121.91 | 8.4 | 3 |
| Voltage-dependent N-type calcium channel subunit alpha-1B* | CAC1B_HUMAN | 264553 | 8.78 |  |  |  |  |  |  |  |  |  | 60.47 | 0.7 | 2 |  |  |  |  |  |  |
| Voltage-gated potassium channel subunit beta-1* | KCAB1_HUMAN | 46990 | 9.10 |  |  |  |  |  |  |  |  |  | 64.09 | 2.9 | 2 |  |  |  |  |  |  |
| VPS10 domain-containing receptor SorCS1 | SORC1_HUMAN | 130694 | 7.38 |  |  |  |  |  |  |  |  |  | 313.49 | 8.4 | 9 |  |  |  |  |  |  |
| V-type proton ATPase catalytic subunit A | VATA_HUMAN | 68660 | 5.35 |  |  |  |  |  |  |  |  |  | 192.54 | 10.9 | 6 | 250.74 | 8.8 | 5 |  |  |  |
| V-type proton ATPase subunit S1 | VAS1_HUMAN | 52164 | 5.73 |  |  |  |  |  |  |  |  |  |  |  |  | 364.04 | 16.6 | 5 |  |  |  |
| WAP. kazal. immunoglobulin. kunitz and NTR domain-containing protein 2 | WFKN2_HUMAN | 66122 | 5.85 |  |  |  |  |  |  |  |  |  | 89.42 | 3.5 | 2 |  |  |  |  |  |  |
| WD repeat and HMG-box DNA-binding protein 1* | WDHD1_HUMAN | 127371 | 5.42 |  |  |  | 53.05 | 1.6 | 2 |  |  |  |  |  |  |  |  |  |  |  |  |
| WD repeat-containing protein 1 | WDR1_HUMAN | 66836 | 6.17 |  |  |  |  |  |  |  |  |  | 207.84 | 9.1 | 5 | 124.87 | 10.2 | 3 | 94.56 | 7.6 | 4 |
| WD repeat-containing protein 61 | WDR61_HUMAN | 33731 | 5.16 |  |  |  |  |  |  |  |  |  | 68.72 | 7.2 | 2 |  |  |  |  |  |  |
| WD repeat-containing protein 66* | WDR66_HUMAN | 131124 | 4.99 |  |  |  |  |  |  |  |  |  | 53.73 | 1.1 | 2 |  |  |  |  |  |  |
| WD repeat-containing protein 87 | WDR87_HUMAN | 335256 | 6.94 | 75.98 | 0.8 | 3 |  |  |  |  |  |  | 57.88 | 0.6 | 2 |  |  |  |  |  |  |
| WD repeat-containing protein 96* | WDR96_HUMAN | 193686 | 5.71 |  |  |  |  |  |  |  |  |  | 56.97 | 0.9 | 2 |  |  |  |  |  |  |
| WD repeat-containing protein KIAA1875 | K1875_HUMAN | 182416 | 7.56 |  |  |  |  |  |  |  |  |  | 80 | 1.5 | 3 |  |  |  |  |  |  |
| Wnt inhibitory factor 1 | WIF1_HUMAN | 43381 | 7.84 | 126.94 | 10.3 | 3 | 226.67 | 16.4 | 5 | 218.78 | 16.1 | 4 | 235.2 | 18.5 | 5 | 102.96 | 7.7 | 2 | 201.89 | 18.5 | 5 |
| WW domain-binding protein 2 | WBP2_HUMAN | 28182 | 5.65 |  |  |  |  |  |  |  |  |  | 80.22 | 7.7 | 2 |  |  |  |  |  |  |
| Xaa-Pro aminopeptidase 1 | XPP1_HUMAN | 70558 | 5.43 | 178.36 | 8.8 | 5 |  |  |  |  |  |  | 155.47 | 6.6 | 3 |  |  |  |  |  |  |
| Xaa-Pro dipeptidase | PEPD_HUMAN | 55311 | 5.64 | 464.69 | 24.5 | 10 | 203.26 | 11.6 | 4 | 265.48 | 16 | 7 | 367.47 | 20.1 | 9 |  |  |  |  |  |  |
| Zeta-sarcoglycan* | SGCZ_HUMAN | 33327 | 7.60 |  |  |  |  |  |  |  |  |  | 53.37 | 4.7 | 2 |  |  |  |  |  |  |
| Zinc finger and SCAN domain-containing protein 30* | ZSC30_HUMAN | 57469 | 6.24 |  |  |  |  |  |  | 52.8 | 3.4 | 2 |  |  |  |  |  |  |  |  |  |
| Zinc finger CCCH domain-containing protein 13 | ZC3HD_HUMAN | 197203 | 9.46 |  |  |  | 48 | 1.2 | 2 |  |  |  | 105.55 | 1.6 | 4 |  |  |  |  |  |  |
| Zinc finger FYVE domain-containing protein 26 | ZFY26_HUMAN | 289128 | 5.97 | 51.93 | 0.6 | 2 |  |  |  |  |  |  |  |  |  |  |  |  |  |  |  |
| Zinc finger MYM-type protein 4* | ZMYM4_HUMAN | 176155 | 6.45 |  |  |  |  |  |  |  |  |  | 51.61 | 1.2 | 2 |  |  |  |  |  |  |
| Zinc finger protein 40* | ZEP1_HUMAN | 299304 | 7.99 |  |  |  |  |  |  |  |  |  | 49.63 | 0.6 | 2 |  |  |  |  |  |  |
| Zinc finger protein 519 | ZN519_HUMAN | 64690 | 9.48 |  |  |  |  |  |  |  |  |  | 65.6 | 3.9 | 3 |  |  |  |  |  |  |
| Zinc finger protein 638 | ZN638_HUMAN | 221914 | 6.02 | 64.82 | 1.1 | 3 |  |  |  |  |  |  | 147.68 | 1.9 | 6 |  |  |  |  |  |  |
| Zinc-alpha-2-glycoprotein | ZA2G_HUMAN | 34465 | 5.71 | 415.19 | 35.2 | 8 | 310.06 | 29.5 | 6 | 302.6 | 29.2 | 6 | 643.25 | 49 | 12 | 628.37 | 48 | 12 | 86.06 | 10.4 | 2 |
| Zymogen granule protein 16 homolog B | ZG16B_HUMAN | 22725 | 6.74 |  |  |  |  |  |  |  |  |  | 427.24 | 38.5 | 8 |  |  |  |  |  |  |
| ZZ-type zinc finger-containing protein 3* | ZZZ3_HUMAN | 103328 | 5.48 | 47.36 | 1.6 | 2 |  |  |  |  |  |  |  |  |  |  |  |  |  |  |  |
| Uncharacterized protein C12orf35* | CL035_HUMAN | 196561 | 8.96 | 52.66 | 0.7 | 2 |  |  |  | 56.05 | 0.9 | 2 |  |  |  |  |  |  |  |  |  |
| **Uncharacterized** |  |  |  |  |  |  |  |  |  |  |  |  |  |  |  |  |  |  |  |  |  |
| Uncharacterized protein C20orf26* | CT026_HUMAN | 143083 | 5.76 |  |  |  |  |  |  |  |  |  | 54.01 | 1.4 | 2 |  |  |  |  |  |  |
| Uncharacterized protein KIAA0556 | K0556_HUMAN | 181946 | 5.58 |  |  |  |  |  |  |  |  |  | 54.8 | 0.7 | 2 |  |  |  |  |  |  |
| Uncharacterized protein KIAA0753 | K0753_HUMAN | 110251 | 7.61 |  |  |  |  |  |  |  |  |  | 113.47 | 3.9 | 5 |  |  |  |  |  |  |
| Uncharacterized protein KIAA0825 | K0825_HUMAN | 149778 | 6.18 |  |  |  |  |  |  | 58.79 | 1.2 | 2 | 79.35 | 1.8 | 3 |  |  |  |  |  |  |
| Uncharacterized protein KIAA0947 | K0947_HUMAN | 250759 | 5.33 |  |  |  |  |  |  |  |  |  | 97.82 | 1.2 | 4 |  |  |  |  |  |  |
| Uncharacterized protein KIAA1109 | K1109_HUMAN | 559352 | 6.12 |  |  |  | 54.29 | 0.4 | 2 |  |  |  | 82.8 | 0.7 | 3 |  |  |  |  |  |  |
|  |  |  |  |  |  |  |  |  |  |  |  |  |  |  |  |  |  |  |  |  |  |
| **Total** | | | | 463 | | | 434 | | | 372 | | | 916 | | | 284 | | | 186 | | |

Score = Mascot Score

Seq.Cov = Sequence Coverage in %

No.pep = Number of peptides
