# Supplementary material for: In-depth mass spectrometric mapping of the human vitreous proteome
Source: Proteome Sci. 2013 May 20;11:22. doi: 10.1186/1477-5956-11-22 (PMC3689628; doi:10.1186/1477-5956-11-22)
Supplement: Additional file 2: Table S2 — Proteins found in vitreous protein samples from all three patients. [file 1477-5956-11-22-S2.doc]

**Table 2: Proteins found in vitreous protein samples from all three patients**

|  | | **Functional Characterization (see legend)** | | | | | | | | | | | | | | | | | | | |
| --- | --- | --- | --- | --- | --- | --- | --- | --- | --- | --- | --- | --- | --- | --- | --- | --- | --- | --- | --- | --- | --- |
| **Protein Name** | **ACC Number** | P | S | I | II | III | IV | V | VI | VII | VIII | IX | X | XI | XII | XIII | XIV | XV | XVI | XVII | XVIII |
| 14-3-3 protein beta/alpha | 1433B_HUMAN |  |  |  |  |  |  |  |  |  |  |  |  |  |  |  |  |  | x |  |  |
| 14-3-3 protein epsilon | 1433E_HUMAN |  |  |  |  |  |  |  |  |  |  |  |  |  |  |  |  |  | x |  |  |
| 14-3-3 protein eta | 1433F_HUMAN |  |  |  |  |  |  |  |  |  |  |  |  |  |  |  |  |  | x |  |  |
| 14-3-3 protein gamma | 1433G_HUMAN |  |  |  |  |  |  |  |  |  |  |  |  |  |  |  |  |  | x |  |  |
| 14-3-3 protein theta | 1433T_HUMAN |  |  |  |  |  |  |  |  |  |  |  |  |  |  |  |  |  | x |  |  |
| 14-3-3 protein zeta/delta | 1433Z_HUMAN |  |  |  |  |  |  |  |  |  |  |  |  |  |  |  |  | x | x |  |  |
| 4-hydroxyphenylpyruvate dioxygenase | HPPD_HUMAN |  |  | x |  |  |  |  |  |  |  |  |  |  |  | x |  |  |  |  |  |
| 4-trimethylaminobutyraldehyde dehydrogenase | AL9A1_HUMAN |  |  | x |  |  |  |  |  |  |  |  |  |  |  |  |  | x |  |  |  |
| 6-phosphofructokinase, liver type | K6PL_HUMAN |  |  | x |  |  |  |  |  |  |  |  |  |  | x |  |  |  |  |  |  |
| 6-phosphogluconate dehydrogenase, decarboxylating | 6PGD_HUMAN |  |  | x |  |  |  |  |  |  |  |  |  |  | x |  |  |  |  |  |  |
| Acetyl-CoA acetyltransferase, cytosolic | THIC_HUMAN |  |  | x |  |  |  |  |  |  |  |  |  |  |  |  |  |  |  |  |  |
| Actin, alpha cardiac muscle 1 | ACTC_HUMAN |  |  |  |  |  |  |  |  |  | x |  |  |  |  |  |  | x |  |  |  |
| Actin, cytoplasmic 1 | ACTB_HUMAN | x |  |  |  |  |  |  |  |  | x |  |  |  |  |  |  | x |  |  |  |
| Acylamino-acid-releasing enzyme | ACPH_HUMAN |  |  | x |  |  |  |  |  |  |  |  |  |  |  |  |  |  |  |  |  |
| Adenosylhomocysteinase | SAHH_HUMAN |  |  | x |  |  |  |  |  |  |  |  |  |  |  |  |  |  |  |  |  |
| Adenylosuccinate lyase | PUR8_HUMAN | x |  | x |  |  |  |  |  |  |  |  |  |  |  |  |  |  |  |  |  |
| Afamin | AFAM_HUMAN | x | x |  |  |  |  |  |  |  | x |  |  |  |  |  |  |  |  |  |  |
| Agrin * | AGRIN_HUMAN | x | x |  |  |  |  |  |  |  |  |  |  |  |  |  |  |  |  | x |  |
| Aldose reductase | ALDR_HUMAN |  |  | x |  |  |  |  |  |  |  |  |  |  | x |  |  |  |  |  |  |
| Alpha-1-antichymotrypsin | AACT_HUMAN | x | x |  |  | x |  |  |  |  |  |  |  |  |  | x |  |  |  |  |  |
| Alpha-1-antitrypsin | A1AT_HUMAN | x | x |  |  | x | x |  |  |  |  |  |  |  |  | x |  |  |  |  |  |
| Alpha-1B-glycoprotein | A1BG_HUMAN | x | x |  |  |  |  |  |  |  |  | x |  |  |  |  |  |  | x |  |  |
| Alpha-2-antiplasmin | A2AP_HUMAN | x | x |  |  | x |  |  |  |  |  |  |  |  |  | x |  |  |  |  |  |
| Alpha-2-HS-glycoprotein | FETUA_HUMAN | x | x |  |  |  |  |  |  |  |  |  |  |  |  | x |  |  |  |  |  |
| Alpha-2-macroglobulin | A2MG_HUMAN | x | x |  |  | x | x |  | x |  |  |  |  |  |  | x |  |  | x |  |  |
| Alpha-actinin-1 | ACTN1_HUMAN | x |  |  |  |  |  |  |  |  |  |  |  |  |  |  |  | x |  |  |  |
| Alpha-crystallin A chain | CRYAA_HUMAN |  |  |  |  |  |  |  |  |  |  |  | x | x |  |  |  |  |  |  |  |
| Alpha-crystallin B chain | CRYAB_HUMAN |  |  |  |  |  |  |  |  |  |  |  | x | x |  |  |  | x |  |  |  |
| Alpha-enolase | ENOA_HUMAN | x |  | x |  |  |  |  |  |  |  |  |  |  |  | x |  |  |  |  |  |
| Amyloid beta A4 protein | A4_HUMAN | x |  |  |  | x |  |  |  |  | x |  |  |  |  | x |  | x |  |  |  |
| Amyloid-like protein 2 | APLP2_HUMAN |  |  |  |  | x |  |  |  |  |  |  |  |  |  | x |  |  | x |  |  |
| Angiotensinogen | ANGT_HUMAN | x | x |  |  |  |  |  |  |  |  |  |  |  |  | x |  |  |  |  |  |
| Antithrombin-III | ANT3_HUMAN | x | x |  |  | x | x |  |  |  |  |  |  |  |  |  |  |  |  |  |  |
| Ankyrin-2 | ANK2_HUMAN | x |  |  |  |  |  |  |  |  |  |  |  |  |  |  |  | x |  |  |  |
| Apolipoprotein A-I | APOA1_HUMAN | x | x |  |  |  |  |  |  |  | x |  |  |  |  |  |  |  |  |  |  |
| Apolipoprotein A-II | APOA2_HUMAN | x | x |  |  |  |  |  |  |  | x |  |  |  |  |  |  |  |  |  |  |
| Apolipoprotein A-IV | APOA4_HUMAN | x | x |  |  |  |  |  |  |  | x |  |  |  |  |  |  |  |  |  |  |
| Apolipoprotein H | APOH_HUMAN | x | x |  |  |  |  |  |  |  | x |  |  |  |  |  | x |  | x |  |  |
| Beta-actin-like protein 2 | ACTBL_HUMAN |  |  |  |  |  |  |  |  |  |  |  |  |  |  |  |  | x |  |  |  |
| Beta-crystallin A2 | CRBA2_HUMAN |  |  |  |  |  |  |  |  |  |  |  |  | x |  |  |  |  |  |  |  |
| Beta-2-microglobulin | B2MG_HUMAN |  | x |  |  |  |  |  |  |  |  |  |  |  |  |  |  |  | x |  |  |
| Beta-crystallin A3 | CRBA3_HUMAN |  |  |  |  |  |  |  |  |  |  |  |  | x |  |  |  |  |  |  |  |
| Beta-crystallin A4 | CRBA4_HUMAN |  |  |  |  |  |  |  |  |  |  |  |  | x |  |  |  |  |  |  |  |
| Beta-crystallin B1 | CRBB1_HUMAN |  |  |  |  |  |  |  |  |  |  |  | x | x |  |  |  |  |  |  |  |
| Beta-crystallin B2 | CRBB2_HUMAN |  |  |  |  |  |  |  |  |  |  |  | x | x |  |  |  |  |  |  |  |
| Beta-crystallin B3 | CRBB3_HUMAN |  |  |  |  |  |  |  |  |  |  |  | x | x |  |  |  |  |  |  |  |
| Beta-crystallin S | CRBS_HUMAN |  |  |  |  |  |  |  |  |  |  |  | x | x |  |  |  |  |  |  |  |
| Biotinidase | BTD_HUMAN | x | x | x |  |  |  |  |  |  |  |  |  |  |  |  |  |  | x |  |  |
| Bisphosphoglycerate mutase | PMGE_HUMAN |  |  | x |  |  |  |  |  |  |  |  |  |  |  |  |  |  |  |  |  |
| Calsyntenin-1 | CSTN1_HUMAN | x |  |  |  |  |  |  |  |  |  |  |  |  |  | x |  |  | x |  |  |
| Carbonic anhydrase 2 | CAH2_HUMAN | x |  | x |  |  |  |  |  |  |  |  |  |  |  | x |  |  |  |  |  |
| Carbonyl reductase [NADPH] 1 | CBR1_HUMAN |  |  | x |  |  |  |  |  |  |  |  |  | x |  |  |  |  |  |  |  |
| Caspase-14 | CASPE_HUMAN | x |  | x | x |  |  |  |  |  |  |  |  |  |  | x | x |  |  |  |  |
| Catenin beta-1 | CTNB1_HUMAN |  |  |  |  |  |  |  |  |  | x |  |  |  |  | x |  | x |  |  |  |
| Cathepsin D | CATD_HUMAN | x |  |  | x |  |  |  |  |  |  |  |  |  |  |  |  |  | x |  |  |
| Centlein | CNTLN_HUMAN |  |  |  |  |  |  |  |  |  |  |  |  |  |  |  |  | x |  |  |  |
| Centrosomal protein of 290 kDa | CE290_HUMAN | x |  |  |  |  |  |  |  |  | x |  |  |  |  |  |  | x |  |  |  |
| Ceruloplasmin | CERU_HUMAN | x | x |  |  |  |  |  |  |  | x |  |  |  |  | x |  |  |  | x |  |
| Chitinase-3-like protein 1 | CH3L1_HUMAN |  | x | x |  |  |  |  |  |  |  |  |  |  |  |  |  |  |  |  |  |
| Clathrin heavy chain 1 | CLH1_HUMAN | x |  |  |  |  |  |  |  |  | x |  |  |  |  |  |  |  | x |  |  |
| Clusterin | CLUS_HUMAN | x | x |  |  |  |  |  |  |  |  |  |  |  |  |  | x |  |  |  |  |
| Coactosin-like protein | COTL1_HUMAN |  |  |  |  |  |  |  |  |  |  |  |  |  |  |  |  | x |  |  |  |
| Collagen alpha-1(VI) chain * | CO6A1_HUMAN |  | x |  |  |  |  |  |  | x | x |  |  |  |  | x |  |  | x | x |  |
| Collagen alpha-1(XVIII) chain | COIA1_HUMAN | x | x |  |  |  |  |  |  | x | x |  |  |  |  | x |  |  | x | x |  |
| Coagulation factor XII | FA12_HUMAN | x | x |  | x |  | x |  |  |  |  |  |  |  |  | x |  |  |  |  |  |
| Complement C3 | CO3_HUMAN | x | x |  |  |  | x |  | x |  |  |  |  |  |  | x |  |  |  |  |  |
| Complement C4-B * | CO4B_HUMAN |  | x |  |  |  | x |  |  |  |  |  |  |  |  |  |  |  |  |  |  |
| Complement component C9 | CO9_HUMAN | x | x |  |  |  | x |  |  |  |  |  |  |  |  |  |  |  | x |  |  |
| Complement factor B | CFAB_HUMAN | x | x |  | x |  | x |  |  |  |  |  |  |  |  |  |  |  | x |  |  |
| Complement factor H | CFAH_HUMAN | x | x |  |  |  | x |  |  |  |  |  |  |  |  |  |  |  | x |  |  |
| Complement factor I | CFAI_HUMAN | x | x |  | x |  | x |  |  |  |  |  |  |  |  |  |  |  |  |  |  |
| Contactin-1 * | CNTN1_HUMAN |  |  |  |  |  |  |  |  |  |  | x |  |  |  |  | x |  | x |  |  |
| Corticosteroid-binding globulin | CBG_HUMAN | x | x |  |  |  |  |  |  |  |  |  |  |  |  | x |  |  |  |  |  |
| Cullin-associated NEDD8-dissociated protein 1 | CAND1_HUMAN |  |  |  |  |  |  |  |  |  |  |  |  |  |  | x |  |  |  |  |  |
| Cytidine deaminase | CDD_HUMAN |  |  | x |  |  |  |  |  |  |  |  |  |  |  |  |  |  |  |  |  |
| Cystatin-C | CYTC_HUMAN | x | x |  |  | x |  |  |  |  |  |  |  |  |  | x |  |  |  |  |  |
| Cytoplasmic dynein 1 heavy chain 1 | DYHC1_HUMAN |  |  |  |  |  |  |  |  |  | x |  |  |  |  |  |  | x |  |  |  |
| Cytosolic non-specific dipeptidase | CNDP2_HUMAN |  |  | x | x |  |  |  |  |  |  |  |  |  |  |  |  |  |  |  |  |
| Dermcidin | DCD_HUMAN | x | x |  |  |  |  |  |  |  |  |  |  |  |  |  |  |  | x |  |  |
| Desmoplakin | DESP_HUMAN | x |  |  |  |  |  |  |  |  |  |  |  |  |  | x |  | x |  |  |  |
| Desmoglein-1 | DSG1_HUMAN | x |  |  |  |  |  |  |  |  |  |  |  |  |  | x |  |  | x |  |  |
| D-dopachrome decarboxylase | DOPD_HUMAN |  |  | x |  |  |  |  | x |  |  |  |  |  |  | x |  |  |  |  |  |
| DNA topoisomerase 2-beta | TOP2B_HUMAN |  |  | x |  |  |  |  |  |  |  |  |  |  |  | x |  |  |  |  |  |
| DNA repair protein RAD50 | RAD50_HUMAN |  |  |  |  |  |  |  |  |  |  |  |  |  |  | x |  |  |  |  |  |
| Dickkopf-related protein 3 | DKK3_HUMAN |  | x |  |  |  |  |  |  |  |  |  |  |  |  |  |  |  | x |  |  |
| Dihydropteridine reductase | DHPR_HUMAN |  |  | x |  |  |  |  |  |  |  |  |  |  |  |  |  |  |  |  |  |
| Elongation factor 2 | EF2_HUMAN |  |  |  |  |  |  |  |  |  |  |  |  |  |  | x |  |  | x |  |  |
| Elongation factor 1-alpha 1 | EF1A1_HUMAN | x |  |  |  |  |  |  |  |  |  |  |  |  |  | x |  |  | x |  |  |
| EGF-containing fibulin-like extracellular matrix protein 1 | FBLN3_HUMAN | x | x |  |  |  |  |  |  |  |  |  |  | x |  | x |  |  | x | x |  |
| E3 ubiquitin-protein ligase SHPRH * | SHPRH_HUMAN |  |  | x |  |  |  |  |  |  |  |  |  |  |  | x |  |  |  |  |  |
| Epoxide hydrolase 2 * | HYES_HUMAN |  |  | x |  |  |  |  |  |  |  |  |  |  |  |  |  |  |  |  |  |
| Fatty acid-binding protein, epidermal | FABP5_HUMAN |  |  |  |  |  |  |  |  |  | x |  |  |  |  | x |  |  | x |  |  |
| Ferritin heavy chain * | FRIH_HUMAN |  |  |  |  |  |  |  |  |  | x |  |  |  |  | x |  |  |  |  |  |
| Epididymal secretory protein E1 | NPC2_HUMAN |  | x |  |  |  |  |  |  |  |  |  |  |  |  |  |  |  | x |  |  |
| Ferritin light chain | FRIL_HUMAN | x |  |  |  |  |  |  |  |  | x |  |  |  |  | x |  |  |  |  |  |
| Fibronectin | FINC_HUMAN | x | x |  |  |  |  |  |  |  | x |  |  |  |  | x |  |  | x | x |  |
| Fibulin-1 * | FBLN1_HUMAN | x | x |  |  |  |  |  |  |  |  | x |  |  |  | x |  |  |  | x |  |
| Filaggrin | FILA_HUMAN |  |  |  |  |  |  |  |  |  |  |  |  |  |  | x |  |  |  |  |  |
| Filamin-A | FLNA_HUMAN | x |  |  |  |  |  |  |  |  |  |  |  |  |  |  |  | x |  |  |  |
| Filaggrin-2 | FILA2_HUMAN |  |  |  |  |  |  |  |  |  |  |  |  |  |  | x |  |  |  |  |  |
| Filensin | BFSP1_HUMAN |  |  |  |  |  |  |  |  |  |  |  | x | x |  |  |  | x |  |  |  |
| Fructose-bisphosphate aldolase A | ALDOA_HUMAN | x |  | x |  |  |  |  |  |  |  |  |  |  |  |  | x |  |  |  |  |
| Fructose-bisphosphate aldolase C | ALDOC_HUMAN |  |  | x |  |  |  |  |  |  |  |  |  |  |  |  |  | x |  |  |  |
| Fumarylacetoacetase | FAAA_HUMAN |  |  | x |  |  |  |  |  |  |  |  |  |  |  | x |  |  |  |  |  |
| Galectin-1 | LEG1_HUMAN |  | x |  |  |  |  |  |  |  |  |  |  |  |  | x | x |  |  |  |  |
| Galectin-3-binding protein | LG3BP_HUMAN |  | x |  |  |  |  |  |  |  | x | x |  |  |  |  | x |  | x |  |  |
| Gamma-crystallin B | CRGB_HUMAN |  |  |  |  |  |  |  |  |  |  |  | x | x |  |  |  |  |  |  |  |
| Gamma-crystallin C | CRGC_HUMAN |  |  |  |  |  |  |  |  |  |  |  | x | x |  |  |  |  |  |  |  |
| Gamma-crystallin D | CRGD_HUMAN |  |  |  |  |  |  |  |  |  |  |  | x |  |  |  |  |  |  |  |  |
| Gamma-enolase | ENOG_HUMAN |  |  | x |  |  |  |  |  |  |  |  |  |  |  | x |  |  |  |  |  |
| Gap junction alpha-3 protein | CXA3_HUMAN |  |  |  |  |  |  |  |  |  |  |  |  |  | x |  |  |  | x |  |  |
| Gelsolin | GELS_HUMAN | x | x |  |  |  |  |  |  |  |  |  |  |  |  | x |  | x |  |  |  |
| Glucose-6-phosphate isomerase | G6PI_HUMAN | x | x | x |  |  |  |  |  |  |  |  |  |  |  |  |  |  |  |  |  |
| Glutathione peroxidase 3 | GPX3_HUMAN | x | x | x |  |  |  |  |  |  |  |  |  |  |  |  |  |  |  |  |  |
| Glutathione synthetase | GSHB_HUMAN | x |  | x |  |  |  |  |  |  |  |  |  |  |  | x |  |  |  |  |  |
| Glutathione reductase, mitochondrial | GSHR_HUMAN | x |  | x |  |  |  |  |  |  |  |  |  |  |  |  | x |  |  |  |  |
| Glutathione S-transferase Mu 2 | GSTM2_HUMAN |  |  | x |  |  |  |  |  |  |  |  |  |  |  |  |  |  |  |  |  |
| Glyceraldehyde-3-phosphate dehydrogenase | G3P_HUMAN | x |  | x |  |  |  |  |  |  |  |  |  |  |  |  |  |  |  |  |  |
| Glycogen phosphorylase, brain form | PYGB_HUMAN |  |  | x |  |  |  |  |  |  |  |  |  |  |  |  |  |  |  |  |  |
| GTP-binding nuclear protein Ran | RAN_HUMAN |  |  |  |  |  |  |  |  |  | x |  |  |  |  | x |  |  | x |  |  |
| Haptoglobin | HPT_HUMAN | x | x |  |  |  |  |  |  |  |  |  |  |  |  | x |  |  |  |  |  |
| Heat shock protein beta-1 | HSPB1_HUMAN |  |  |  |  |  |  |  |  |  |  |  | x | x |  |  |  | x |  |  |  |
| Heat shock protein HSP 90-beta | HS90B_HUMAN |  |  |  |  |  |  |  |  |  |  |  |  |  |  |  |  |  |  |  | x |
| Heat shock cognate 71 kDa protein | HSP7C_HUMAN | x |  |  |  |  |  |  |  |  |  |  |  |  |  | x | x |  | x |  |  |
| Heat shock protein HSP 90-alpha | HS90A_HUMAN | x |  |  |  |  |  |  |  |  |  |  |  |  |  |  |  |  |  |  | x |
| Hemopexin | HEMO_HUMAN | x | x |  |  |  |  |  |  |  | x |  |  |  |  |  |  |  |  |  |  |
| Heparin cofactor 2 | HEP2_HUMAN | x |  |  |  | x | x |  |  |  |  |  |  |  |  | x |  |  |  |  |  |
| Histidine-rich glycoprotein | HRG_HUMAN | x | x |  |  |  |  |  |  |  |  |  |  |  |  |  |  |  |  |  |  |
| Hornerin | HORN_HUMAN | x |  |  |  |  |  |  |  |  |  |  |  |  |  |  |  | x |  |  |  |
| Hydroxyacylglutathione hydrolase, mitochondrial | GLO2_HUMAN |  | x | x |  |  |  |  |  |  |  |  |  |  |  | x |  |  |  |  |  |
| Ig alpha-1 chain C region | IGHA1_HUMAN | x |  |  |  |  |  |  |  |  |  |  |  |  |  | x |  |  |  |  |  |
| Ig gamma-1 chain C region | IGHG1_HUMAN | x |  |  |  |  |  |  |  |  |  |  |  |  |  | x |  |  |  |  |  |
| Ig gamma-2 chain C region | IGHG2_HUMAN | x | x |  |  |  |  |  |  |  |  |  |  |  |  | x |  |  |  |  |  |
| Ig gamma-3 chain C region | IGHG3_HUMAN | x | x |  |  |  |  |  |  |  |  |  |  |  |  | x |  |  |  |  |  |
| Ig gamma-4 chain C region | IGHG4_HUMAN | x | x |  |  |  |  |  |  |  |  |  |  |  |  | x |  |  |  |  |  |
| Ig kappa chain C region | IGKC_HUMAN | x |  |  |  |  |  |  |  |  |  |  |  |  |  | x |  |  |  |  |  |
| Ig lambda-2 chain C regions | LAC2_HUMAN | x |  |  |  |  |  |  |  |  |  |  |  |  |  | x |  |  |  |  |  |
| Ig kappa chain V-I region EU | KV106_HUMAN | x |  |  |  |  |  |  |  |  |  |  |  |  |  | x |  |  |  |  |  |
| Ig kappa chain V-II region MIL | KV203_HUMAN | x |  |  |  |  |  |  |  |  |  |  |  |  |  | x |  |  |  |  |  |
| Ig kappa chain V-III region WOL * | KV305_HUMAN | x |  |  |  |  |  |  |  |  |  |  |  |  |  | x |  |  |  |  |  |
| Ig kappa chain V-IV region Len | KV402_HUMAN | x |  |  |  |  |  |  |  |  |  |  |  |  |  | x |  |  |  |  |  |
| Ig heavy chain V-III region BRO | HV305_HUMAN | x |  |  |  |  |  |  |  |  |  |  |  |  |  | x |  |  |  |  |  |
| Ig heavy chain V-III region CAM | HV307_HUMAN | x |  |  |  |  |  |  |  |  |  |  |  |  |  | x |  |  |  |  |  |
| IgGFc-binding protein | FCGBP_HUMAN | x | x |  |  |  |  |  |  |  |  |  |  |  |  | x |  |  |  |  |  |
| Importin subunit beta-1 | IMB1_HUMAN |  |  |  |  |  |  |  |  |  | x |  |  |  |  |  |  |  |  |  |  |
| Immunoglobulin lambda-like polypeptide 5 | IGLL5_HUMAN | x | x |  |  |  |  |  |  |  |  |  |  |  |  |  |  |  | x |  |  |
| Importin-5 | IPO5_HUMAN |  |  |  |  |  |  |  |  |  | x |  |  |  |  | x |  |  |  |  |  |
| Inter-alpha-trypsin inhibitor heavy chain H1 | ITIH1_HUMAN | x | x |  |  | x |  |  |  |  |  |  |  |  |  | x |  |  |  |  |  |
| Inter-alpha-trypsin inhibitor heavy chain H2 | ITIH2_HUMAN | x | x |  |  | x |  |  |  |  |  |  |  |  |  | x |  |  |  |  |  |
| Inter-alpha-trypsin inhibitor heavy chain H4 | ITIH4_HUMAN | x | x |  |  | x |  |  |  |  |  |  |  |  |  | x |  |  |  |  |  |
| Isoleucyl-tRNA synthetase, cytoplasmic | SYIC_HUMAN |  |  | x |  |  |  |  |  |  |  |  |  |  |  |  |  |  |  |  |  |
| Junction plakoglobin | PLAK_HUMAN | x |  |  |  |  |  |  |  |  | x |  |  |  |  | x |  | x | x |  |  |
| Kallistatin | KAIN_HUMAN | x | x |  |  | x |  |  |  |  |  |  |  |  |  | x |  |  |  |  |  |
| Keratin. type I cytoskeletal 10 | K1C10_HUMAN | x |  |  |  |  |  |  |  |  |  |  |  |  |  |  |  | x |  |  |  |
| Keratin. type I cytoskeletal 9 | K1C9_HUMAN | x |  |  |  |  |  |  |  |  |  |  |  |  |  |  |  | x |  |  |  |
| Keratin. type II cytoskeletal 2 epidermal | K22E_HUMAN | x |  |  |  |  |  |  |  |  |  |  |  |  |  |  |  | x |  |  |  |
| Keratin. type II cytoskeletal 1 | K2C1_HUMAN |  |  |  |  |  |  |  |  |  |  |  |  |  |  |  |  | x |  |  |  |
| Keratin. type II cytoskeletal 5 | K2C5_HUMAN | x |  |  |  |  |  |  |  |  |  |  |  |  |  |  |  | x |  |  |  |
| Keratin. type II cytoskeletal 78 | K2C78_HUMAN |  |  |  |  |  |  |  |  |  |  |  |  |  |  |  | x |  |  |  |  |
| Keratin. type II cytoskeletal 80 | K2C80_HUMAN |  |  |  |  |  |  |  |  |  |  |  |  |  |  |  |  | x |  |  |  |
| Keratin. type II cytoskeletal 6B | K2C6B_HUMAN | x |  |  |  |  |  |  |  |  |  |  |  |  |  |  | x |  |  |  |  |
| Keratin. type II cytoskeletal 6A | K2C6A_HUMAN |  |  |  |  |  |  |  |  |  |  |  |  |  |  |  | x |  |  |  |  |
| Keratin. type I cytoskeletal 14 | K1C14_HUMAN | x |  |  |  |  |  |  |  |  |  |  |  |  |  |  | x |  |  |  |  |
| Keratin. type I cytoskeletal 16 | K1C16_HUMAN | x |  |  |  |  |  |  |  |  |  |  |  |  |  |  | x |  |  |  |  |
| Keratinocyte proline-rich protein | KPRP_HUMAN |  |  |  |  |  |  |  |  |  |  |  |  |  |  |  |  | x |  |  |  |
| Kininogen-1 | KNG1_HUMAN | x | x |  |  | x | x |  |  |  |  |  |  |  |  | x |  |  |  |  |  |
| Lengsin | LGSN_HUMAN |  |  |  |  |  |  |  |  |  |  |  |  |  |  |  |  |  |  |  | x |
| Lens fiber major intrinsic protein | MIP_HUMAN |  |  |  |  |  |  |  |  |  | x |  |  |  |  |  |  |  |  |  |  |
| Leucine-rich alpha-2-glycoprotein | A2GL_HUMAN | x | x |  |  |  |  |  |  |  |  | x |  |  |  |  |  |  | x | x |  |
| Leukocyte elastase inhibitor | ILEU_HUMAN |  |  |  |  | x |  |  |  |  |  |  |  |  |  | x |  |  |  |  |  |
| Liver carboxylesterase 1 * | EST1_HUMAN |  |  | x |  |  |  |  |  | x |  |  |  |  |  | x |  |  | x |  |  |
| L-lactate dehydrogenase A chain | LDHA_HUMAN | x |  | x |  |  |  |  |  |  |  |  |  |  |  | x |  |  |  |  |  |
| L-lactate dehydrogenase B chain | LDHB_HUMAN | x |  | x |  |  |  |  |  |  |  |  |  |  |  |  |  |  |  |  |  |
| Low-density lipoprotein receptor-related protein 2 | LRP2_HUMAN |  |  |  |  |  |  |  |  |  |  | x |  |  |  |  |  |  |  | x |  |
| Lysozyme C | LYSC_HUMAN | x | x | x |  |  |  |  |  |  |  |  |  |  |  |  |  |  | x |  |  |
| Malate dehydrogenase, cytoplasmic | MDHC_HUMAN |  |  | x |  |  |  |  |  |  |  |  |  |  |  |  |  |  |  |  |  |
| Matrix Metalloproteinase 2 | MMP2_HUMAN | x | x | x | x |  |  |  |  |  |  |  |  |  | x |  |  |  |  | x |  |
| Microtubule-associated tumor suppressor candidate 2 * | MTUS2_HUMAN | x |  |  |  |  |  |  |  |  |  |  |  |  |  |  |  | x |  |  |  |
| Multiple epidermal growth factor-like domains protein 8 | MEGF8_HUMAN | x |  |  |  |  |  |  |  |  |  |  |  |  |  |  |  |  | x | x |  |
| Monocyte differentiation antigen CD14 | CD14_HUMAN | x |  |  |  |  |  |  |  |  |  | x |  |  |  |  |  |  |  |  |  |
| Myocilin * | MYOC_HUMAN | x | x |  |  |  |  |  |  |  | x |  | x | x |  |  |  |  | x |  |  |
| Myotrophin | MTPN_HUMAN |  |  |  |  |  |  | x |  |  |  |  |  |  |  |  |  |  |  |  |  |
| N-acetyllactosaminide beta-1,3-N-acetylglucosaminyltransferase | B3GN1_HUMAN |  |  | x |  |  |  |  |  |  |  |  |  |  |  |  |  |  |  |  |  |
| N-acetylmuramoyl-L-alanine amidase | PGRP2_HUMAN |  | x | x |  |  |  |  |  |  |  |  |  |  |  | x | x |  |  |  |  |
| NADH-cytochrome b5 reductase 2 | NB5R2_HUMAN |  |  | x |  |  |  |  |  |  |  |  |  |  |  |  |  |  |  |  |  |
| NADH dehydrogenase [ubiquinone] 1 alpha subcomplex subunit 13 | NDUAD_HUMAN |  |  | x |  |  |  |  |  |  |  |  |  |  |  |  | x |  |  |  |  |
| NADP-dependent malic enzyme | MAOX_HUMAN |  |  |  |  |  |  |  |  |  |  |  |  |  |  | x |  |  |  |  |  |
| Neuronal cell adhesion molecule | NRCAM_HUMAN |  |  |  |  |  |  |  |  |  |  | x |  |  |  |  | x |  | x |  |  |
| Neural cell adhesion molecule L1-like protein | CHL1_HUMAN | x | x |  |  |  |  |  |  |  |  | x |  |  |  |  | x |  | x |  |  |
| Nesprin-1 | SYNE1_HUMAN | x |  |  |  |  |  |  |  |  |  |  |  |  |  |  |  | x |  |  |  |
| Ninein | NIN_HUMAN |  |  |  |  |  |  |  |  |  |  |  |  |  |  | x |  | x |  |  |  |
| Nucleoside diphosphate kinase A | NDKA_HUMAN |  |  | x |  |  |  |  |  |  |  |  |  |  |  |  |  | x |  |  |  |
| Nucleoside diphosphate kinase B | NDKB_HUMAN |  |  | x |  |  |  |  |  |  |  |  |  |  |  | x |  |  |  |  |  |
| Nucleosome assembly protein 1-like 1 * | NP1L1_HUMAN | x |  |  |  |  |  |  |  |  |  |  |  |  |  | x | x |  |  |  |  |
| Nucleosome assembly protein 1-like 4 | NP1L4_HUMAN |  |  |  |  |  |  |  |  |  |  |  |  |  |  | x | x |  |  |  |  |
| Opticin | OPT_HUMAN |  | x |  |  |  |  |  |  |  |  | x |  |  |  |  |  |  | x | x |  |
| Osteopontin | OSTP_HUMAN |  | x |  |  |  |  |  | x |  |  |  |  |  |  | x |  |  |  | x |  |
| Osteoclast-stimulating factor 1 * | OSTF1_HUMAN |  |  |  |  |  |  |  |  |  |  |  |  |  |  |  |  |  |  |  | x |
| Pappalysin-2 4 | PAPP2_HUMAN | x | x |  | x |  |  |  |  |  |  |  |  |  |  |  |  |  | x |  |  |
| Peptidyl-prolyl cis-trans isomerase A | PPIA_HUMAN |  |  | x |  |  |  |  |  |  | x |  |  |  |  |  |  |  |  |  |  |
| Peroxiredoxin-2 | PRDX2_HUMAN | x |  | x |  |  |  |  |  |  |  |  |  |  |  |  |  |  |  |  |  |
| Peroxiredoxin-6 | PRDX6_HUMAN |  |  |  |  |  |  |  |  |  |  |  |  |  |  |  |  |  |  |  | x |
| Phakinin | BFSP2_HUMAN | x |  |  |  |  |  |  |  |  |  |  | x |  |  |  |  | x |  |  |  |
| Phosphatidylethanolamine-binding protein 1 | PEBP1_HUMAN |  |  |  |  | x |  |  |  |  |  |  |  |  |  | x |  |  | x |  |  |
| Phosphoglycerate kinase 1 | PGK1_HUMAN |  |  | x |  |  |  |  |  |  |  |  |  |  |  | x |  |  |  |  |  |
| Phosphoglycerate mutase 1 | PGAM1_HUMAN | x |  | x |  |  |  |  |  |  |  |  |  |  |  |  |  |  |  |  |  |
| Phosphoglucomutase-like protein 5 | PGM5_HUMAN |  |  | x |  |  |  |  |  |  |  |  |  |  |  |  |  | x |  |  |  |
| Pigment epithelium-derived factor | PEDF_HUMAN | x | x |  |  |  |  |  |  |  |  |  |  |  |  | x |  |  |  |  |  |
| PITH domain-containing protein 1 * | PITH1_HUMAN |  |  |  |  |  |  |  |  |  |  |  |  |  |  |  |  |  |  |  | x |
| Plasma protease C1 inhibitor | IC1_HUMAN | x | x | x |  | x | x |  |  |  |  |  |  |  |  | x |  |  |  |  |  |
| Plectin | PLEC_HUMAN | x |  |  |  |  |  |  |  |  |  |  |  |  |  | x |  | x |  |  |  |
| Profilin-1 | PROF1_HUMAN | x |  |  |  |  |  |  |  |  |  |  |  |  |  |  |  | x |  |  |  |
| Prothrombin | THRB_HUMAN | x | x |  | x |  | x |  |  |  |  |  |  |  |  | x |  |  |  |  |  |
| Prostaglandin-H2 D-isomerase | PTGDS_HUMAN | x | x | x |  |  |  |  |  |  | x |  |  |  |  |  |  |  | x |  |  |
| Protein DJ-1 | PARK7_HUMAN |  |  |  |  |  |  |  |  |  |  |  |  |  |  | x |  |  |  |  |  |
| Protein FAM49B | FA49B_HUMAN |  |  |  |  |  |  |  |  |  |  |  |  |  |  |  |  |  |  |  | x |
| Protein AMBP | AMBP_HUMAN | x | x |  |  | x |  |  |  |  |  |  |  |  |  | x |  |  |  |  |  |
| Protein S100-A8 * | S10A8_HUMAN | x | x |  |  |  |  |  |  |  |  |  |  |  |  | x |  |  | x |  |  |
| Protein-L-isoaspartate(D-aspartate) O-methyltransferase | PIMT_HUMAN |  |  | x |  |  |  |  |  |  |  |  |  |  |  |  |  |  |  |  |  |
| Pterin-4-alpha-carbinolamine dehydratase | PHS_HUMAN |  |  | x |  |  |  |  |  |  |  |  |  |  |  |  |  |  |  |  |  |
| Pyruvate kinase isozymes M1/M2 | KPYM_HUMAN | x |  | x |  |  |  |  |  |  |  |  |  |  |  |  |  |  |  |  |  |
| Quinone oxidoreductase PIG3 | QORX_HUMAN |  |  | x |  |  |  |  |  |  |  |  |  |  |  |  | x |  |  |  |  |
| Rab GDP dissociation inhibitor alpha | GDIA_HUMAN | x |  |  |  |  |  |  |  |  | x |  |  |  |  | x |  |  | x |  |  |
| Rab GDP dissociation inhibitor beta | GDIB_HUMAN |  |  |  |  |  |  |  |  |  | x |  |  |  |  | x |  |  | x |  |  |
| Retinal dehydrogenase 1 | AL1A1_HUMAN | x |  | x |  |  |  |  |  |  |  |  |  |  |  | x |  |  |  |  |  |
| Retinol-binding protein 4 | RET4_HUMAN | x |  |  |  |  |  |  |  |  | x |  |  |  |  | x |  |  | x |  |  |
| Retinol-binding protein 3 | RET3_HUMAN |  | x |  |  |  |  |  |  |  | x |  |  | x |  |  |  |  |  |  |  |
| Retinol-binding protein 1 | RET1_HUMAN |  |  |  |  |  |  |  |  |  | x |  |  |  |  | x |  |  | x |  |  |
| Rho GDP-dissociation inhibitor 1 | GDIR1_HUMAN |  |  |  |  |  |  |  |  |  |  |  |  |  |  | x |  | x | x |  |  |
| Ribonuclease III | CJ068_HUMAN |  |  |  |  |  |  |  |  |  |  |  |  |  |  |  |  |  |  |  | x |
| Ribonuclease inhibitor | RINI_HUMAN |  |  | x |  |  |  |  |  |  |  |  |  |  |  | x |  |  |  |  |  |
| Ribonuclease pancreatic | RNAS1_HUMAN |  | x | x |  |  |  |  |  |  |  |  |  |  |  | x |  |  |  |  |  |
| S-formylglutathione hydrolase | ESTD_HUMAN |  |  | x |  |  |  |  |  |  |  |  |  |  |  |  |  |  |  |  |  |
| Semaphorin-7A | SEM7A_HUMAN |  |  |  |  |  |  |  |  |  |  |  |  |  |  | x |  |  | x |  |  |
| Seizure protein 6 homolog | SEZ6_HUMAN |  |  |  |  |  |  |  |  |  |  |  |  |  |  |  |  |  | x |  |  |
| Serotransferrin | TRFE_HUMAN | x | x |  |  |  |  |  |  |  | x |  |  |  |  |  |  |  |  |  |  |
| Serpin B6 | SPB6_HUMAN |  |  |  |  | x |  |  |  |  |  |  |  |  |  | x |  |  |  |  |  |
| Serpin B9 | SPB9_HUMAN |  |  |  |  | x |  |  |  |  |  |  |  |  |  | x |  |  |  |  |  |
| Serum albumin | ALBU_HUMAN | x | x |  |  |  |  |  |  |  | x |  |  |  |  |  |  |  |  |  |  |
| Serum amyloid A-4 protein | SAA4_HUMAN | x | x |  |  |  |  |  |  |  |  |  |  |  |  |  |  |  |  |  |  |
| Serum paraoxonase/arylesterase 1 | PON1_HUMAN | x | x | x |  |  |  |  |  |  |  |  |  |  |  |  |  |  |  |  |  |
| Sister chromatid cohesion protein PDS5 homolog B | PDS5B_HUMAN |  |  |  |  |  |  |  |  |  |  |  |  |  |  | x |  |  |  |  |  |
| Sorbitol dehydrogenase | DHSO_HUMAN |  |  | x |  |  |  |  |  |  |  |  |  |  |  |  | x |  |  |  |  |
| Spectrin alpha chain, brain | SPTA2_HUMAN | x |  |  |  |  |  |  |  |  |  |  |  |  |  |  |  | x |  |  |  |
| Spectrin beta chain, brain 1 | SPTB2_HUMAN |  |  |  |  |  |  |  |  |  |  |  |  |  |  |  |  | x |  |  |  |
| Spondin-1 | SPON1_HUMAN |  | x |  |  |  |  |  |  |  |  |  |  |  |  |  |  |  | x |  |  |
| T-complex protein 1 subunit delta | TCPD_HUMAN |  |  |  |  |  |  |  |  |  |  |  |  |  |  |  |  |  |  |  | x |
| Target of Nesh-SH3 | TARSH_HUMAN |  | x |  |  |  |  |  |  |  |  |  |  |  |  |  |  |  | x |  |  |
| Thioredoxin | THIO_HUMAN | x |  |  |  |  |  |  |  |  |  |  |  |  |  |  |  |  | x |  |  |
| Titin | TITIN_HUMAN | x |  |  |  |  |  |  |  |  |  |  |  |  |  | x |  |  |  |  |  |
| Thyroxine-binding globulin | THBG_HUMAN | x | x |  |  |  |  |  |  |  |  |  |  |  |  | x |  |  |  |  |  |
| Transketolase | TKT_HUMAN | x |  | x |  |  |  |  |  |  |  |  |  |  |  | x |  |  |  |  |  |
| Transthyretin | TTHY_HUMAN | x | x |  |  |  |  |  |  |  | x |  |  |  |  |  |  |  | x |  |  |
| Triosephosphate isomerase | TPIS_HUMAN | x |  | x |  |  |  |  |  |  |  |  |  |  |  |  |  |  |  |  |  |
| Tubulin alpha-1A chain | TBA1A_HUMAN | x |  |  |  |  |  |  |  |  | x |  |  |  |  |  |  | x |  |  |  |
| Tubulin alpha-1B chain * | TBA1B_HUMAN | x |  |  |  |  |  |  |  |  | x |  |  |  |  |  |  | x |  |  |  |
| Tubulin beta chain | TBB5_HUMAN | x |  |  |  |  |  |  |  |  | x |  |  |  |  |  |  | x |  |  |  |
| Ubiquitin carboxyl-terminal hydrolase isozyme L1 | UCHL1_HUMAN | x |  | x | x |  |  |  |  |  |  |  |  |  |  |  |  |  |  |  |  |
| Ubiquitin-40S ribosomal protein S27a | RS27A_HUMAN |  |  |  |  |  |  |  |  |  |  |  |  |  |  | x |  |  |  |  |  |
| Ubiquitin-like modifier-activating enzyme 1 | UBA1_HUMAN |  |  | x |  |  |  |  |  |  |  |  |  |  |  | x |  |  | x |  |  |
| Versican core protein * | CSPG2_HUMAN |  | x |  |  |  |  |  |  |  |  |  |  |  |  |  |  |  | x | x |  |
| Vimentin | VIME_HUMAN |  |  |  |  |  |  |  |  |  |  |  | x |  |  |  |  | x |  |  |  |
| Vitamin D-binding protein | VTDB_HUMAN | x | x |  |  |  |  |  |  |  | x |  |  |  |  |  |  |  |  |  |  |
| Vitronectin | VTNC_HUMAN | x | x |  |  |  |  |  |  |  |  |  |  |  |  |  |  |  | x |  |  |
| Wnt inhibitory factor 1 | WIF1_HUMAN |  | x |  |  |  |  |  |  |  | x |  |  | x |  | x |  |  | x |  |  |
| Xaa-Pro dipeptidase | PEPD_HUMAN | x |  | x | x |  |  |  |  |  |  |  |  |  |  | x |  |  |  |  |  |
| Zinc-alpha-2-glycoprotein | ZA2G_HUMAN | x | x |  |  |  |  |  |  |  |  | x |  |  |  | x |  |  |  |  |  |
