# Supplementary material for: In-depth mass spectrometric mapping of the human vitreous proteome
Source: Proteome Sci. 2013 May 20;11:22. doi: 10.1186/1477-5956-11-22 (PMC3689628; doi:10.1186/1477-5956-11-22)
Supplement: Additional file 3: Table S3 — Functional annotation of vitreous proteins. Classification and functional annotation was conducted as described in Materials and Methods. P: proteins catalogued or annotated as plasma proteins; S: predicted as secreted proteins. The Roman numerals indicate the following annotations; I: Enzymes, II: Proteases, III: Protease Inhibitors, IV: Complement and coagulation system, V: Growths factors, VI: Cytokines, VII: Peptide hormones, VIII: Transport, IX: Receptors, X: Structure, XI: Visual perception, XII: Ion channel activity, XIII: Binding, XIV: Apoptosis, XV: Cytoskeleton, XVI: Signaling, XVII: Intercellular, XVIII: Miscellaneous [file 1477-5956-11-22-S3.doc]

**Supplementary Table 3: Functional annotation of vitreous proteins.**

Classification and functional annotation was conducted as described in Materials and Methods. P: proteins catalogued or annotated as plasma proteins; S: predicted as secreted proteins. The Roman numerals indicate the following annotations; I: Enzymes, II: Proteases, III: Protease Inhibitors, IV: Complement and coagulation system, V: Growths factors, VI: Cytokines, VII: Peptide hormones, VIII: Transport, IX: Receptors, X: Structure, XI: Visual perception, XII: Ion channel activity, XIII: Binding, XIV: Apoptosis, XV: Cytoskeleton, XVI: Signaling, XVII: Intercellular, XVIII: Miscellaneous

|  | | **Functional Characterization (see legend)** | | | | | | | | | | | | | | | | | | | |
| --- | --- | --- | --- | --- | --- | --- | --- | --- | --- | --- | --- | --- | --- | --- | --- | --- | --- | --- | --- | --- | --- |
| **Protein Name** | **ACC Number** | P | S | I | II | III | IV | V | VI | VII | VIII | IX | X | XI | XII | XIII | XIV | XV | XVI | XVII | XVIII |
| 14-3-3 protein beta/alpha | 1433B_HUMAN |  |  |  |  |  |  |  |  |  |  |  |  |  |  |  |  |  | x |  |  |
| 14-3-3 protein epsilon | 1433E_HUMAN |  |  |  |  |  |  |  |  |  |  |  |  |  |  |  |  |  | x |  |  |
| 14-3-3 protein eta | 1433F_HUMAN |  |  |  |  |  |  |  |  |  |  |  |  |  |  |  |  |  | x |  |  |
| 14-3-3 protein gamma | 1433G_HUMAN |  |  |  |  |  |  |  |  |  |  |  |  |  |  |  |  |  | x |  |  |
| 14-3-3 protein sigma | 1433S_HUMAN | x | x |  |  |  |  |  |  |  |  |  |  |  |  |  |  |  | x |  |  |
| 14-3-3 protein theta | 1433T_HUMAN |  |  |  |  |  |  |  |  |  |  |  |  |  |  |  |  |  | x |  |  |
| 14-3-3 protein zeta/delta | 1433Z_HUMAN |  |  |  |  |  |  |  |  |  |  |  |  |  |  |  |  | x | x |  |  |
| 1-phosphatidylinositol-4,5-bisphosphate phosphodiesterase eta-1 | PLCH1_HUMAN |  |  | x |  |  |  |  |  |  |  |  |  |  |  | x |  |  | x |  |  |
| 3-hydroxyanthranilate 3,4-dioxygenase | 3HAO_HUMAN |  |  | x |  |  |  |  |  |  |  |  |  |  |  | x |  |  |  |  |  |
| 26S protease regulatory subunit 6A | PRS6A_HUMAN |  |  | x |  |  |  |  |  |  |  |  |  |  |  |  |  |  |  |  |  |
| 26S proteasome non-ATPase regulatory subunit 11 | PSD11_HUMAN |  |  |  |  |  |  |  |  |  | x |  |  |  |  |  |  |  | x |  |  |
| 26S proteasome non-ATPase regulatory subunit 2 | PSMD2_HUMAN |  |  |  |  |  |  |  |  |  |  |  |  |  |  | x |  |  |  |  |  |
| 26S proteasome non-ATPase regulatory subunit 13 | PSD13_HUMAN |  |  |  |  |  |  |  |  |  |  |  |  |  |  |  |  |  |  |  | x |
| 40S ribosomal protein SA | RSSA_HUMAN |  |  |  |  |  |  |  |  |  |  |  |  |  |  | x |  |  |  |  |  |
| 4-hydroxyphenylpyruvate dioxygenase | HPPD_HUMAN |  |  | x |  |  |  |  |  |  |  |  |  |  |  | x |  |  |  |  |  |
| 4-trimethylaminobutyraldehyde dehydrogenase | AL9A1_HUMAN |  |  | x |  |  |  |  |  |  |  |  |  |  |  |  |  | x |  |  |  |
| 6-phosphofructokinase type C | K6PP_HUMAN |  |  | x |  |  |  |  |  |  |  |  |  |  |  |  |  |  |  |  |  |
| 6-phosphofructokinase, liver type | K6PL_HUMAN |  |  | x |  |  |  |  |  |  |  |  |  |  | x |  |  |  |  |  |  |
| 6-phosphofructokinase, muscle type | K6PF_HUMAN |  |  | x |  |  |  |  |  |  |  |  |  |  | x |  |  |  |  |  |  |
| 6-phosphogluconate dehydrogenase, decarboxylating | 6PGD_HUMAN |  |  | x |  |  |  |  |  |  |  |  |  |  | x |  |  |  |  |  |  |
| 6-phosphogluconolactonase | 6PGL_HUMAN |  |  | x |  |  |  |  |  |  |  |  |  |  | x |  |  |  |  |  |  |
| A disintegrin and metalloproteinase with thrombospondin motifs 5 | ATS5_HUMAN |  | x |  | x |  |  |  |  |  |  |  |  |  |  | x |  |  | x |  |  |
| Abnormal spindle-like microcephaly-associated protein | ASPM_HUMAN |  |  |  |  |  |  |  |  |  | x |  |  |  |  | x |  | x | x |  |  |
| Abhydrolase domain-containing protein 14B | ABHEB_HUMAN |  |  | x |  |  |  |  |  |  |  |  |  |  |  |  |  |  |  |  |  |
| Acetyl-CoA acetyltransferase, cytosolic | THIC_HUMAN |  |  | x |  |  |  |  |  |  |  |  |  |  |  |  |  |  |  |  |  |
| Acetyl-CoA carboxylase 1 | ACACA_HUMAN | x |  | x |  |  |  |  |  |  |  |  |  |  |  |  |  |  |  |  |  |
| Acid ceramidase | ASAH1_HUMAN |  |  | x |  |  |  |  |  |  |  |  |  |  |  |  |  |  | x |  |  |
| Actin, alpha cardiac muscle 1 | ACTC_HUMAN |  |  |  |  |  |  |  |  |  | x |  |  |  |  |  |  | x |  |  |  |
| Actin, cytoplasmic 1 | ACTB_HUMAN | x |  |  |  |  |  |  |  |  | x |  |  |  |  |  |  | x |  |  |  |
| Actin-related protein 2 | ARP2_HUMAN |  |  |  |  |  |  |  |  |  | x |  |  |  |  |  |  | x |  |  |  |
| Actin-related protein 2/3 complex subunit 4 | ARPC4_HUMAN |  |  |  |  |  |  |  |  |  |  |  |  |  |  |  |  | x |  |  |  |
| Actin-related protein 3 | ARP3_HUMAN |  |  |  |  |  |  |  |  |  |  |  | x |  |  |  |  |  |  | x |  |
| Actin-related protein 3B | ARP3B_HUMAN |  |  |  |  |  |  |  |  |  | x |  |  |  |  |  |  | x |  | x |  |
| Acylamino-acid-releasing enzyme | ACPH_HUMAN |  |  | x |  |  |  |  |  |  |  |  |  |  |  |  |  |  |  |  |  |
| Acylphosphatase-2 | ACYP2_HUMAN |  |  | x |  |  |  |  |  |  |  |  |  |  |  |  |  |  |  |  |  |
| Adenosylhomocysteinase | SAHH_HUMAN |  |  | x |  |  |  |  |  |  |  |  |  |  |  |  |  |  |  |  |  |
| Adenylosuccinate lyase | PUR8_HUMAN | x |  | x |  |  |  |  |  |  |  |  |  |  |  |  |  |  |  |  |  |
| Adenylyl cyclase-associated protein 1 | CAP1_HUMAN |  |  |  |  |  |  |  |  |  |  |  |  |  |  |  |  | x | x | x |  |
| Adenylyl cyclase-associated protein 2 | CAP2_HUMAN |  |  |  |  |  |  |  |  |  |  |  |  |  |  |  |  |  | x | x |  |
| ADP-ribosylation factor 1 | ARF1_HUMAN |  |  |  |  |  |  |  |  |  | x |  |  |  |  | x |  |  | x |  |  |
| ADP-ribosylation factor-like protein 3 | ARL3_HUMAN |  |  |  |  |  |  |  |  |  | x |  |  |  |  | x |  | x | x |  |  |
| ADP-sugar pyrophosphatase | NUDT5_HUMAN |  |  | x |  |  |  |  |  |  |  |  |  |  |  | x |  |  |  |  |  |
| Afamin | AFAM_HUMAN | x | x |  |  |  |  |  |  |  | x |  |  |  |  |  |  |  |  |  |  |
| Aflatoxin B1 aldehyde reductase member 2 | ARK72_HUMAN | x |  | x |  |  |  |  |  |  |  |  |  |  | x |  |  |  |  |  |  |
| Agrin | AGRIN_HUMAN | x | x |  |  |  |  |  |  |  |  |  |  |  |  |  |  |  |  | x |  |
| Alanyl-tRNA synthetase, cytoplasmic | SYAC_HUMAN |  |  | x |  |  |  |  |  |  |  |  |  |  |  | x |  |  |  |  |  |
| Alcohol dehydrogenase [NADP+] | AK1A1_HUMAN | x |  | x |  |  |  |  |  |  |  |  |  |  | x |  |  |  |  |  |  |
| Aldose 1-epimerase | GALM_HUMAN |  |  | x |  |  |  |  |  |  |  |  |  |  |  | x |  |  |  |  |  |
| Aldose reductase | ALDR_HUMAN |  |  | x |  |  |  |  |  |  |  |  |  |  | x |  |  |  |  |  |  |
| Alpha-actinin-4 | ACTN4_HUMAN |  |  |  |  |  |  |  |  |  | x |  |  |  |  | x |  |  |  |  |  |
| Alpha-1-acid glycoprotein 1 | A1AG1_HUMAN | x | x |  |  |  |  |  |  |  |  |  |  |  |  |  |  |  | x |  |  |
| Alpha-1-acid glycoprotein 2 | A1AG2_HUMAN | x | x |  |  |  |  |  |  |  |  |  |  |  |  |  |  |  | x |  |  |
| Alpha-1-antichymotrypsin | AACT_HUMAN | x | x |  |  | x |  |  |  |  |  |  |  |  |  | x |  |  |  |  |  |
| Alpha-1-antitrypsin | A1AT_HUMAN | x | x |  |  | x | x |  |  |  |  |  |  |  |  | x |  |  |  |  |  |
| Alpha-1B-glycoprotein | A1BG_HUMAN | x | x |  |  |  |  |  |  |  |  | x |  |  |  |  |  |  | x |  |  |
| Alpha-2-antiplasmin | A2AP_HUMAN | x | x |  |  | x |  |  |  |  |  |  |  |  |  | x |  |  |  |  |  |
| Alpha-2-HS-glycoprotein | FETUA_HUMAN | x | x |  |  |  |  |  |  |  |  |  |  |  |  | x |  |  |  | x |  |
| Alpha-2-macroglobulin | A2MG_HUMAN | x | x |  |  | x | x |  | x |  |  |  |  |  |  | x |  |  | x |  |  |
| Alpha-actinin-1 | ACTN1_HUMAN | x |  |  |  |  |  |  |  |  |  |  |  |  |  |  |  | x |  |  |  |
| Alpha-centractin | ACTZ_HUMAN |  |  |  |  |  |  |  |  |  | x |  |  |  |  |  |  | x |  |  |  |
| Alpha-crystallin A chain | CRYAA_HUMAN |  |  |  |  |  |  |  |  |  |  |  | x | x |  |  |  |  |  |  |  |
| Alpha-crystallin B chain | CRYAB_HUMAN |  |  |  |  |  |  |  |  |  |  |  | x | x |  |  |  | x |  |  |  |
| Alpha-enolase | ENOA_HUMAN | x |  | x |  |  |  |  |  |  |  |  |  |  |  | x |  |  |  |  |  |
| Alpha-mannosidase 2C1 | MA2C1_HUMAN |  |  | x |  |  |  |  |  |  |  |  |  |  |  | x |  |  |  |  |  |
| Alpha-soluble NSF attachment protein | SNAA_HUMAN |  |  |  |  |  |  |  |  |  | x |  |  |  |  |  |  |  |  |  |  |
| Amyloid beta A4 protein | A4_HUMAN | x |  |  |  | x |  |  |  |  | x |  |  |  |  | x |  | x |  |  |  |
| Amyloid-like protein 1 | APLP1_HUMAN |  |  |  |  |  |  |  |  |  |  |  |  |  |  | x | x |  | x |  |  |
| Amyloid-like protein 2 | APLP2_HUMAN |  |  |  |  | x |  |  |  |  |  |  |  |  |  | x |  |  | x |  |  |
| Angiotensinogen | ANGT_HUMAN | x | x |  |  |  |  |  |  |  |  |  |  |  |  | x |  |  |  |  |  |
| Ankyrin repeat and SOCS box protein 2 | ASB2_HUMAN |  |  |  |  |  |  |  |  |  |  |  |  |  |  |  |  |  |  |  | x |
| Ankyrin repeat domain-containing protein 17 | ANR17_HUMAN |  |  |  |  |  |  |  |  |  |  |  |  |  |  | x |  |  |  |  |  |
| Ankyrin repeat domain-containing protein 20A1 | A20A1_HUMAN |  |  |  |  |  |  |  |  |  |  |  |  |  |  |  |  |  |  |  | x |
| Ankyrin repeat domain-containing protein 30B | AN30B_HUMAN |  |  |  |  |  |  |  |  |  |  |  |  |  |  |  |  |  |  |  | x |
| Ankyrin repeat domain-containing protein 35 | ANR35_HUMAN |  |  |  |  |  |  |  |  |  |  |  |  |  |  |  |  |  |  |  | x |
| Ankyrin-1 | ANK1_HUMAN | x |  |  |  |  |  |  |  |  |  |  |  |  |  | x |  | x |  | x |  |
| Ankyrin-2 | ANK2_HUMAN | x |  |  |  |  |  |  |  |  |  |  |  |  |  |  |  | x |  | x |  |
| Ankyrin-3 | ANK3_HUMAN | x |  |  |  |  |  |  |  |  |  |  |  |  |  | x |  | x |  |  |  |
| Annexin A1 | ANXA1_HUMAN | x |  |  |  |  |  |  |  |  | x |  |  |  |  | x |  | x | x |  |  |
| Annexin A2 | ANXA2_HUMAN | x | x |  |  |  |  |  |  |  | x |  |  |  |  | x |  |  | x |  |  |
| Annexin A3 | ANXA3_HUMAN | x |  |  |  |  |  |  |  |  | x |  |  |  |  | x |  |  | x |  |  |
| Annexin A5 | ANXA5_HUMAN |  |  |  |  |  |  |  |  |  | x |  |  |  |  | x |  |  | x |  |  |
| Antileukoproteinase | SLPI_HUMAN |  | x | x | x |  |  |  |  |  |  |  |  |  |  | x |  |  |  |  |  |
| Antithrombin-III | ANT3_HUMAN | x | x |  |  | x | x |  |  |  |  |  |  |  |  |  |  |  |  |  |  |
| Antigen KI-67 | KI67_HUMAN |  |  |  |  |  |  |  |  |  |  |  |  |  |  | x |  |  |  |  |  |
| Apolipoprotein A-I | APOA1_HUMAN | x | x |  |  |  |  |  |  |  | x |  |  |  |  |  |  |  |  |  |  |
| Apolipoprotein A-I-binding protein | AIBP_HUMAN |  | x |  |  |  |  |  |  |  |  |  |  |  |  |  |  |  |  |  |  |
| Apolipoprotein A-II | APOA2_HUMAN | x | x |  |  |  |  |  |  |  | x |  |  |  |  |  |  |  |  |  |  |
| Apolipoprotein A-IV | APOA4_HUMAN | x | x |  |  |  |  |  |  |  | x |  |  |  |  |  |  |  |  |  |  |
| Apolipoprotein B-100 | APOB_HUMAN | x | x |  |  |  |  |  |  |  | x |  |  |  |  |  |  |  |  |  |  |
| Apolipoprotein D | APOD_HUMAN | x | x |  |  |  |  |  |  |  |  |  |  |  |  |  |  |  |  |  |  |
| Apolipoprotein E | APOE_HUMAN | x | x |  |  |  |  |  |  |  | x |  |  |  |  |  |  |  |  |  |  |
| Apolipoprotein H | APOH_HUMAN | x | x |  |  |  |  |  |  |  | x |  |  |  |  |  | x |  | x |  |  |
| Arf-GAP with Rho-GAP domain, ANK repeat and PH domain-containing protein 2 | ARAP2_HUMAN |  |  |  |  |  |  |  |  |  |  |  |  |  |  | x |  |  | x |  |  |
| Arginase-1 | ARGI1_HUMAN | x |  | x |  |  |  |  |  |  |  |  |  |  |  |  |  |  |  |  |  |
| Aspartate aminotransferase, cytoplasmic | AATC_HUMAN |  |  | x |  |  |  |  |  |  |  |  |  |  |  |  |  |  |  |  |  |
| Aspartate aminotransferase, mitochondrial | AATM_HUMAN |  |  | x |  |  |  |  |  |  | x |  |  |  |  |  |  |  |  |  |  |
| Astrocytic phosphoprotein PEA-15 | PEA15_HUMAN |  |  |  |  |  |  |  |  |  |  |  |  |  |  |  |  | x |  |  |  |
| Ataxin-2 | ATX2_HUMAN |  |  |  |  |  |  |  |  |  |  |  |  |  |  | x |  |  |  |  |  |
| ATPase family AAA domain-containing protein 3A | ATD3A_HUMAN |  |  |  |  |  |  |  |  |  |  |  |  |  |  | x |  |  |  |  |  |
| ATP-binding cassette sub-family A member 12 | ABCAC_HUMAN | x |  |  |  |  |  |  |  |  | x |  |  |  |  | x |  |  |  |  |  |
| ATP-binding cassette sub-family A member 13 | ABCAD_HUMAN |  |  |  |  |  |  |  |  |  | x |  |  |  |  | X |  |  |  |  |  |
| ATP-binding cassette sub-family B member 9 | ABCB9_HUMAN |  |  |  |  |  |  |  |  |  | x |  |  |  |  | X |  |  |  |  |  |
| ATP-binding cassette sub-family F member 1 | ABCF1_HUMAN | x |  |  |  |  |  |  |  |  | x |  |  |  |  | X |  |  |  |  |  |
| ATP-binding cassette sub-family F member 3 | ABCF3_HUMAN |  |  |  |  |  |  |  |  |  | x |  |  |  |  | X |  |  |  |  |  |
| ATP-binding cassette transporter sub-family C member 11 | ABCCB_HUMAN | x |  |  |  |  |  |  |  |  | x |  |  |  |  | X |  |  |  |  |  |
| ATP-binding cassette transporter sub-family C member 8 | ABCC8_HUMAN | x |  |  |  |  |  |  |  |  | x |  |  |  |  | X |  |  |  |  |  |
| Attractin | ATRN_HUMAN | x | x |  |  |  |  |  |  |  |  | x |  |  |  |  |  |  | x |  |  |
| Azurocidin | CAP7_HUMAN |  |  |  |  |  |  |  |  |  |  |  |  |  |  | x | x |  | x |  |  |
| BAI1-associated protein 3 | BAIP3_HUMAN |  |  |  |  |  |  |  |  |  |  |  |  |  |  | x |  |  | x |  |  |
| Baculoviral IAP repeat-containing protein 6 | BIRC6_HUMAN |  |  |  |  | x |  |  |  |  |  |  |  |  |  |  | x | x |  |  |  |
| Band 4.1-like protein 3 | E41L3_HUMAN |  |  |  |  |  |  |  |  |  |  |  |  |  |  | x |  | x |  |  |  |
| Basement membrane-specific heparan sulfate proteoglycan core protein | PGBM_HUMAN | x | x |  |  |  |  |  |  |  |  |  |  |  |  |  |  |  | x | x |  |
| Bax inhibitor 1 | BI1_HUMAN |  |  |  |  |  |  |  |  |  |  |  |  |  |  |  | x |  |  |  |  |
| Beta-1,4-galactosyltransferase 1 | B4GT1_HUMAN |  | x | x |  |  |  |  |  |  |  |  |  |  |  |  |  |  |  |  |  |
| Beta-2-microglobulin | B2MG_HUMAN |  | x |  |  |  |  |  |  |  |  |  |  |  |  |  |  |  | x |  |  |
| Beta-actin-like protein 2 | ACTBL_HUMAN |  |  |  |  |  |  |  |  |  |  |  |  |  |  |  |  | x |  |  |  |
| Beta-Ala-His dipeptidase | CNDP1_HUMAN |  | x | x | x |  |  |  |  |  |  |  |  |  |  |  |  |  |  |  |  |
| Beta-crystallin A2 | CRBA2_HUMAN |  |  |  |  |  |  |  |  |  |  |  |  | x |  |  |  |  |  |  |  |
| Beta-crystallin A3 | CRBA1_HUMAN |  |  |  |  |  |  |  |  |  |  |  | x | x |  |  |  |  |  |  |  |
| Beta-crystallin A4 | CRBA4_HUMAN |  |  |  |  |  |  |  |  |  |  |  |  | x |  |  |  |  |  |  |  |
| Beta-crystallin B1 | CRBB1_HUMAN |  |  |  |  |  |  |  |  |  |  |  | x | x |  |  |  |  |  |  |  |
| Beta-crystallin B2 | CRBB2_HUMAN |  |  |  |  |  |  |  |  |  |  |  | x | x |  |  |  |  |  |  |  |
| Beta-crystallin B3 | CRBB3_HUMAN |  |  |  |  |  |  |  |  |  |  |  | x | x |  |  |  |  |  |  |  |
| Beta-crystallin S | CRBS_HUMAN |  |  |  |  |  |  |  |  |  |  |  | x | x |  |  |  |  |  |  |  |
| Beta-enolase | ENOB_HUMAN |  |  | x |  |  |  |  |  |  |  |  |  |  |  | x |  |  |  |  |  |
| Beta-hexosaminidase subunit alpha | HEXA_HUMAN |  |  | x |  |  |  |  |  |  |  |  |  |  |  |  |  |  |  |  |  |
| Beta-hexosaminidase subunit beta | HEXB_HUMAN |  |  | x |  |  |  |  |  |  |  |  |  |  |  |  |  |  | x |  |  |
| Biotinidase | BTD_HUMAN | x | x | x |  |  |  |  |  |  |  |  |  |  |  |  |  |  | x |  |  |
| Bisphosphoglycerate mutase | PMGE_HUMAN |  |  | x |  |  |  |  |  |  |  |  |  |  |  |  |  |  |  |  |  |
| Bleomycin hydrolase | BLMH_HUMAN | x |  | x | x |  |  |  |  |  |  |  |  |  |  |  |  |  |  |  |  |
| BPI fold-containing family A member 1 | BPIA1_HUMAN |  | x |  |  |  |  |  |  |  |  |  |  |  | x |  |  | x |  |  |  |
| BPI fold-containing family B member 1 | BPIB1_HUMAN |  | x |  |  |  |  |  |  |  |  |  |  |  | x |  |  | x |  |  |  |
| BPI fold-containing family B member 2 | BPIB2_HUMAN |  | x |  |  |  |  |  |  |  |  |  |  |  | x |  |  | x |  |  |  |
| Brain acid soluble protein 1 | BASP1_HUMAN |  |  |  |  |  |  |  |  |  | x |  |  |  |  |  |  | x |  |  |  |
| Brain-specific angiogenesis inhibitor 1 | BAI1_HUMAN |  |  |  |  |  |  |  |  |  |  | x |  |  |  |  |  |  | x |  |  |
| Brefeldin A-inhibited guanine nucleotide-exchange protein 2 | BIG2_HUMAN |  |  |  |  |  |  |  |  |  | x |  |  |  |  | x |  |  |  |  |  |
| Brevican core protein | PGCB_HUMAN |  | x |  |  |  |  |  |  |  |  |  |  |  |  |  |  |  | x | x |  |
| Bromodomain adjacent to zinc finger domain protein 2B | BAZ2B_HUMAN | x |  |  |  |  |  |  |  |  |  |  |  |  |  | x |  |  |  |  |  |
| Bromodomain-containing protein 4 | BRD4_HUMAN | x |  |  |  |  |  |  |  |  |  |  |  |  |  | x |  |  |  |  |  |
| C3 and PZP-like alpha-2-macroglobulin domain-containing protein 8 | CPMD8_HUMAN |  | x |  |  | x |  |  | x |  |  |  |  |  |  | x |  |  | x |  |  |
| C-Jun-amino-terminal kinase-interacting protein 4 | JIP4_HUMAN |  |  |  |  |  |  |  |  | x |  |  |  |  |  | x |  |  |  |  |  |
| Cadherin-2 | CADH2_HUMAN |  |  |  |  |  |  |  |  |  |  | x |  | x |  | x |  |  | x |  |  |
| Cadherin-6 | CADH6_HUMAN |  |  |  |  |  |  |  |  |  |  | x |  | x |  | x |  |  | x |  |  |
| Cadherin-related family member 1 | CDHR1_HUMAN |  |  |  |  |  |  |  |  |  |  |  |  |  |  | x |  |  | x |  |  |
| Calcium-dependent secretion activator 2 | CAPS2_HUMAN |  |  |  |  |  |  |  |  |  | x |  |  |  |  | x |  |  |  |  |  |
| Calcium-regulated heat stable protein 1 | CHSP1_HUMAN |  |  |  |  |  |  |  |  |  |  |  |  |  |  | x |  |  | x |  |  |
| Calcyphosin-2 | CAYP2_HUMAN |  |  |  |  |  |  |  |  |  |  |  |  |  |  | x |  |  |  |  |  |
| Caldesmon | CALD1_HUMAN |  |  |  |  |  |  |  |  |  |  |  |  |  |  | x |  | x |  |  |  |
| Calmodulin-like protein 5 | CALL5_HUMAN |  |  |  |  |  |  |  |  |  |  |  |  |  |  | x |  |  | x |  |  |
| Calreticulin | CALR_HUMAN |  | x |  |  |  |  |  |  |  |  |  |  |  |  | x |  |  |  |  |  |
| Calsyntenin-1 | CSTN1_HUMAN | x |  |  |  |  |  |  |  |  |  |  |  |  |  | x |  |  | x |  |  |
| Calsyntenin-2 | CSTN2_HUMAN |  |  |  |  |  |  |  |  |  |  |  |  |  |  | x |  |  | x |  |  |
| CAP-Gly domain-containing linker protein 1 | CLIP1_HUMAN |  |  |  |  |  |  |  |  |  | x |  |  |  |  |  |  | x |  |  |  |
| CAP-Gly domain-containing linker protein 2 | CLIP2_HUMAN |  |  |  |  |  |  |  |  |  | x |  |  |  |  | x |  | x |  |  |  |
| cAMP-dependent protein kinase type I-alpha regulatory subunit | KAP0_HUMAN |  |  | x |  |  |  |  |  |  |  |  |  |  |  | x |  |  | x |  |  |
| Carbohydrate kinase domain-containing protein | CARKD_HUMAN |  |  | x |  |  |  |  |  |  |  |  |  |  |  |  |  |  | x |  |  |
| Carbonic anhydrase 1 | CAH1_HUMAN | x |  | x |  |  |  |  |  |  |  |  |  |  |  | x |  |  |  |  |  |
| Carbonic anhydrase 14 | CAH14_HUMAN | x |  | x |  |  |  |  |  |  |  |  |  |  |  | x |  |  |  |  |  |
| Carbonic anhydrase 2 | CAH2_HUMAN | x |  | x |  |  |  |  |  |  |  |  |  |  |  | x |  |  |  |  |  |
| Carbonic anhydrase-related protein 10 | CAH10_HUMAN |  |  | x |  |  |  |  |  |  |  |  |  |  |  | x |  |  |  |  |  |
| Carbonyl reductase [NADPH] 1 | CBR1_HUMAN |  |  | x |  |  |  |  |  |  |  |  |  | x |  |  |  |  |  |  |  |
| Carboxypeptidase B2 | CBPB2_HUMAN | x | x | x | x |  | x |  |  |  |  |  |  |  |  |  |  |  |  |  |  |
| Carboxypeptidase E | CBPE_HUMAN |  | x | x | x |  |  |  |  |  |  |  |  |  |  |  |  |  |  |  |  |
| Carboxypeptidase N subunit 2 | CPN2_HUMAN | x | x | x |  |  |  |  |  |  |  | x |  |  |  |  |  |  |  | x |  |
| Cardiomyopathy-associated protein 5 | CMYA5_HUMAN |  |  | x |  |  |  |  |  |  |  |  |  |  |  |  |  |  |  |  |  |
| Cartilage acidic protein 1 | CRAC1_HUMAN | x | x |  |  |  |  |  |  |  |  |  |  |  |  | x |  |  |  |  |  |
| Caspase-8 | CASP8_HUMAN | x |  |  | x |  |  |  |  |  |  |  |  |  |  |  | x |  |  |  |  |
| Caspase-14 | CASPE_HUMAN | x |  | x | x |  |  |  |  |  |  |  |  |  |  | x | x |  |  |  |  |
| Catalase | CATA_HUMAN | x |  | x |  |  |  |  |  |  |  |  |  |  |  | x |  |  |  |  |  |
| Catenin alpha-1 | CTNA1_HUMAN | x |  |  |  |  |  |  |  |  |  |  |  |  |  | x |  | x |  | x |  |
| Catenin alpha-2 | CTNA2_HUMAN | x |  |  |  |  |  |  |  |  |  |  |  |  |  | x |  | x |  | x |  |
| Catenin beta-1 | CTNB1_HUMAN |  |  |  |  |  |  |  |  |  | x |  |  |  |  | x |  | x |  | x |  |
| Cathepsin D | CATD_HUMAN | x |  |  | x |  |  |  |  |  |  |  |  |  |  |  |  |  | x |  |  |
| Cathepsin L1 | CATL1_HUMAN |  |  |  | x |  |  |  |  |  |  |  |  |  |  |  |  |  |  |  |  |
| Cathepsin Z | CATZ_HUMAN | x |  |  | x |  |  |  |  |  |  |  |  |  |  |  |  |  | x |  |  |
| Caveolin-1 | CAV1_HUMAN |  |  |  |  |  |  |  |  |  | x |  |  |  |  | x |  |  |  |  |  |
| Cell adhesion molecule 1 | CADM1_HUMAN |  |  |  |  |  |  |  |  |  |  | x |  |  |  |  |  |  |  |  |  |
| Cell adhesion molecule 2 | CADM2_HUMAN |  |  |  |  |  |  |  |  |  |  | x |  |  |  |  |  |  |  |  |  |
| Cell adhesion molecule-related/down-regulated by oncogenes | CDON_HUMAN |  |  |  |  |  |  |  |  |  |  | x |  |  |  |  | x |  |  |  |  |
| Cell division cycle 5-like protein | CDC5L_HUMAN |  |  |  |  |  |  |  |  |  |  |  |  |  |  | x | x |  |  |  |  |
| Centlein | CNTLN_HUMAN |  |  |  |  |  |  |  |  |  |  |  |  |  |  |  |  | x |  |  |  |
| Centriolin | CNTRL_HUMAN |  |  |  |  |  |  |  |  |  |  |  |  |  |  |  |  | x |  |  |  |
| Centrosome-associated protein 350 | CE350_HUMAN |  |  |  |  |  |  |  |  |  |  |  |  |  |  |  |  |  |  |  | x |
| Centromere protein F | CENPF_HUMAN | x |  |  |  |  |  |  |  |  |  |  |  |  |  |  |  | x |  |  |  |
| Centrosomal protein of 164 kDa | CE164_HUMAN | x |  |  |  |  |  |  |  |  |  |  |  |  |  |  |  | x |  |  |  |
| Centrosomal protein of 290 kDa | CE290_HUMAN | x |  |  |  |  |  |  |  |  | x |  |  |  |  |  |  | x |  |  |  |
| Centrosome and spindle pole-associated protein 1 | CSPP1_HUMAN |  |  |  |  |  |  |  |  |  |  |  |  |  |  |  |  | x |  |  |  |
| Centromere-associated protein E | CENPE_HUMAN | x |  |  |  |  |  |  |  |  |  |  |  |  |  |  |  | x | x |  |  |
| Ceramide kinase | CERK1_HUMAN |  |  | x |  |  |  |  |  |  |  |  |  |  |  |  |  |  |  |  |  |
| Cerebellin-1 | CBLN1_HUMAN |  | x |  |  |  |  |  |  | x |  |  |  |  |  | x |  |  |  |  |  |
| Ceruloplasmin | CERU_HUMAN | x | x |  |  |  |  |  |  |  | x |  |  |  |  | x |  |  |  | x |  |
| Chitinase-3-like protein 1 | CH3L1_HUMAN |  | x | x |  |  |  |  |  |  |  |  |  |  |  |  |  |  |  |  |  |
| Chloride intracellular channel protein 1 | CLIC1_HUMAN |  |  |  |  |  |  |  |  |  | x |  |  |  | x | x |  |  |  | x |  |
| Chloride intracellular channel protein 5 | CLIC5_HUMAN |  |  |  |  |  |  |  |  |  | x |  |  |  | x | x |  | x |  | x |  |
| Chondroitin sulfate N-acetylgalactosaminyltransferase 1 | CGAT1_HUMAN |  |  | x |  |  |  |  |  |  |  |  |  |  |  |  |  |  |  |  |  |
| Chondroitin sulfate synthase 1 | CHSS1_HUMAN |  |  | x |  |  |  |  |  |  | x |  |  |  |  | x |  |  |  |  |  |
| Chromogranin-A | CMGA_HUMAN |  | x |  |  |  |  |  |  | x |  |  |  |  |  | x |  |  |  |  |  |
| Cingulin | CING_HUMAN | x |  |  |  |  |  |  |  |  | x |  |  |  |  | x |  | x | x | x |  |
| Cingulin-like protein 1 | CGNL1_HUMAN |  |  |  |  |  |  |  |  |  |  |  |  |  |  | x |  | x |  |  |  |
| Clathrin heavy chain 1 | CLH1_HUMAN | x |  |  |  |  |  |  |  |  | x |  |  |  |  |  |  |  | x | x |  |
| Clusterin | CLUS_HUMAN | x | x |  |  |  |  |  |  |  |  |  |  |  |  |  | x |  |  |  |  |
| Clusterin-associated protein 1 | CLUA1_HUMAN |  |  |  |  |  |  |  |  |  |  |  |  |  |  |  |  |  |  |  | x |
| Coactosin-like protein | COTL1_HUMAN |  |  |  |  |  |  |  |  |  |  |  |  |  |  |  |  | x |  |  |  |
| Coagulation factor V | FA5_HUMAN | x | x |  |  |  | x |  |  |  | x |  |  |  |  | x |  |  | x | x |  |
| Coagulation factor XII | FA12_HUMAN | x | x |  | x |  | x |  |  |  |  |  |  |  |  | x |  |  |  |  |  |
| Cofilin-1 | COF1_HUMAN |  |  |  |  |  |  |  |  |  |  |  |  |  |  | x |  | x |  | x |  |
| Cofilin-2 | COF2_HUMAN |  |  |  |  |  |  |  |  |  |  |  |  |  |  |  |  |  | x |  | x |
| Coiled-coil and C2 domain-containing protein 2A | C2D2A_HUMAN | x |  |  |  |  |  |  |  |  |  |  |  |  |  |  |  | x |  |  |  |
| Coiled-coil domain-containing protein 87 | CCD87_HUMAN |  |  |  |  |  |  |  |  |  |  |  |  |  |  |  |  |  |  |  | x |
| Coiled-coil domain-containing protein 30 | CCD30_HUMAN |  |  |  |  |  |  |  |  |  |  |  |  |  |  |  |  |  |  |  | x |
| Coiled-coil domain-containing protein 88B | CC88B_HUMAN |  |  |  |  |  |  |  |  |  |  |  |  |  |  | x |  |  | x |  |  |
| Coiled-coil domain-containing protein 110 | CC110_HUMAN |  |  |  |  |  |  |  |  |  |  |  |  |  |  |  |  |  |  |  | x |
| Coiled-coil domain-containing protein 121 | CC121_HUMAN |  |  |  |  |  |  |  |  |  |  |  |  |  |  |  |  |  |  |  | x |
| Coiled-coil domain-containing protein 138 | CC138_HUMAN |  |  |  |  |  |  |  |  |  |  |  |  |  |  |  |  |  |  |  | x |
| Coiled-coil domain-containing protein 147 | CC147_HUMAN |  |  |  |  |  |  |  |  |  |  |  |  |  |  |  |  |  |  |  | x |
| Coiled-coil domain-containing protein 144A | CC144A_HUMAN |  |  |  |  |  |  |  |  |  |  |  |  |  |  |  |  |  |  |  | x |
| Coiled-coil domain-containing protein 148 | CC148_HUMAN |  |  |  |  |  |  |  |  |  |  |  |  |  |  |  |  |  |  |  | x |
| Collagen alpha-1(II) chain | CO2A1_HUMAN |  | x |  |  |  |  |  |  |  | x |  |  |  |  | x |  |  |  | x |  |
| Collagen alpha-1(IX) chain | CO9A1_HUMAN | x | x |  |  |  |  |  |  | x | x | x |  |  |  | x |  |  | x | x |  |
| Collagen alpha-1(VI) chain | CO6A1_HUMAN | x | x |  |  |  |  |  |  | x | x |  |  |  |  | x |  |  | x | x |  |
| Collagen alpha-1(XII) chain | COCA1_HUMAN |  | x |  |  |  |  |  |  | x | x |  |  |  |  | x |  |  | x | x |  |
| Collagen alpha-1(XVIII) chain | COIA1_HUMAN | x | x |  |  |  |  |  |  | x | x |  |  |  |  | x |  |  | x | x |  |
| Collagen alpha-2(IX) chain | CO9A2_HUMAN |  | x |  |  |  |  |  |  | x | x |  |  |  |  | x |  |  | x | x |  |
| COMM domain-containing protein 4 | COMD4_HUMAN |  |  |  |  |  |  |  |  |  |  |  |  |  |  |  |  |  | x |  |  |
| Complement C1q subcomponent subunit B | C1QB_HUMAN | x | x |  |  |  | x |  |  | x | x |  |  |  |  | x |  |  |  |  |  |
| Complement C1q subcomponent subunit C | C1QC_HUMAN | x | x |  |  |  | x |  |  | x | x |  |  |  |  | x |  |  |  |  |  |
| Complement C1q tumor necrosis factor-related protein 3 | C1QT3_HUMAN |  | x |  |  |  | x |  |  |  |  |  |  |  |  |  |  |  | x |  |  |
| Complement C1r subcomponent | C1R_HUMAN | x |  |  | x |  | x |  |  |  |  |  |  |  |  | x |  |  |  |  |  |
| Complement C1s subcomponent | C1S_HUMAN | x |  |  | x |  | x |  |  |  |  |  |  |  |  | x |  |  |  |  |  |
| Complement C1r subcomponent-like protein | C1RL_HUMAN |  | x |  | x |  | x |  |  |  |  |  |  |  |  |  |  |  |  |  |  |
| Complement C2 | CO2_HUMAN | x | x |  | x |  | x |  |  |  |  |  |  |  |  |  |  |  |  |  |  |
| Complement C3 | CO3_HUMAN | x | x |  |  |  | x |  | x |  |  |  |  |  |  | x |  |  |  |  |  |
| Complement C4-A | CO4A_HUMAN | x | x |  |  |  | x |  | x |  |  |  |  |  |  | x |  |  |  |  |  |
| Complement C4-B | CO4B_HUMAN |  | x |  |  |  | x |  |  |  |  |  |  |  |  |  |  |  |  |  |  |
| Complement C5 | CO5_HUMAN | x | x |  |  |  | x |  | x |  |  |  |  |  |  | x |  |  | x |  |  |
| Complement component C6 | CO6_HUMAN | x | x |  |  |  | x |  |  |  |  |  |  |  |  |  |  |  | x |  |  |
| Complement component C7 | CO7_HUMAN | x | x |  |  |  | x |  |  |  |  |  |  |  |  |  |  |  | x |  |  |
| Complement component C8 gamma chain | CO8G_HUMAN | x | x |  |  |  | x |  |  |  |  |  |  |  |  | x |  |  |  |  |  |
| Complement component C9 | CO9_HUMAN | x | x |  |  |  | x |  |  |  |  |  |  |  |  |  |  |  | x |  |  |
| Complement factor B | CFAB_HUMAN | x | x |  | x |  | x |  |  |  |  |  |  |  |  |  |  |  | x |  |  |
| Complement factor D | CFAD_HUMAN | x | x |  | x |  | x |  |  |  |  |  |  |  |  | x |  |  |  |  |  |
| Complement factor H | CFAH_HUMAN | x | x |  |  |  | x |  |  |  |  |  |  |  |  |  |  |  | x |  |  |
| Complement factor I | CFAI_HUMAN | x | x |  | x |  | x |  |  |  |  |  |  |  |  |  |  |  |  |  |  |
| Condensin-2 complex subunit D3 | CNDD3_HUMAN |  |  |  |  |  |  |  |  |  |  |  |  |  |  | x |  |  |  |  |  |
| Conserved oligomeric Golgi complex subunit 2 | COG2_HUMAN |  |  |  |  |  |  |  |  |  | x |  |  |  |  |  |  |  |  |  |  |
| Constitutive coactivator of PPAR-gamma-like protein 2 | F120C_HUMAN |  |  |  |  |  |  |  |  |  |  |  |  |  |  |  |  |  |  |  | x |
| Contactin-1 | CNTN1_HUMAN |  |  |  |  |  |  |  |  |  |  | x |  |  |  |  | x |  | x |  |  |
| Contactin-2 | CNTN2_HUMAN | x |  |  |  |  |  |  |  |  |  | x |  |  |  |  | x |  | x |  |  |
| COP9 signalosome complex subunit 5 | CSN5_HUMAN | x |  |  | x |  |  |  |  |  |  |  |  |  |  | x |  |  |  |  |  |
| COP9 signalosome complex subunit 6 | CSN6_HUMAN |  |  |  |  |  |  |  |  |  |  |  |  |  |  | x |  |  | x |  |  |
| Copine-3 | CPNE3_HUMAN |  |  |  |  |  |  |  |  |  | x |  |  |  |  |  |  |  |  |  |  |
| Corneodesmosin | CDSN_HUMAN |  | x |  |  |  |  |  |  |  |  |  |  |  |  |  |  | x |  |  |  |
| Cornifin-A | SPR1A_HUMAN |  |  |  |  |  |  |  |  |  |  |  | x |  |  |  |  |  |  |  |  |
| Cornifin-B | SPR1B_HUMAN |  |  |  |  |  |  |  |  |  |  |  | x |  |  |  |  |  |  |  |  |
| Corticosteroid-binding globulin | CBG_HUMAN | x | x |  |  |  |  |  |  |  |  |  |  |  |  | x |  |  |  |  |  |
| Cullin-1 | CUL1_HUMAN | x |  |  |  |  |  |  |  |  |  |  |  |  |  |  | x |  |  |  |  |
| Cullin-5 | CUL5_HUMAN |  |  |  |  |  |  |  |  |  |  |  |  |  | x |  | x |  |  |  |  |
| Cullin-associated NEDD8-dissociated protein 1 | CAND1_HUMAN |  |  |  |  |  |  |  |  |  |  |  |  |  |  | x |  |  |  |  |  |
| Cystatin-A | CYTA_HUMAN | x |  |  |  | x |  |  |  |  |  |  |  |  |  | x |  | x |  |  |  |
| Cystatin-B | CYTB_HUMAN | x |  |  |  | x |  |  |  |  |  |  |  |  |  | x |  |  |  |  |  |
| Cystatin-C | CYTC_HUMAN | x | x |  |  | x |  |  |  |  |  |  |  |  |  | x |  |  |  |  |  |
| Cysteine-rich secretory protein 3 | CRIS3_HUMAN | x | x |  |  |  |  |  |  |  |  |  |  |  |  |  |  |  |  |  |  |
| Cystic fibrosis transmembrane conductance regulator | CFTR_HUMAN | x |  |  |  |  |  |  |  |  | x |  |  |  | x |  |  |  |  |  |  |
| Cytidine deaminase | CDD_HUMAN |  |  | x |  |  |  |  |  |  |  |  |  |  |  |  |  |  |  |  |  |
| Cytoplasmic aconitate hydratase | ACOC_HUMAN |  |  | x |  |  |  |  |  |  |  |  |  |  |  |  |  |  |  |  |  |
| Cytoplasmic dynein 1 heavy chain 1 | DYHC1_HUMAN |  |  |  |  |  |  |  |  |  | x |  |  |  |  |  |  | x |  | x |  |
| Cytosolic non-specific dipeptidase | CNDP2_HUMAN |  |  | x | x |  |  |  |  |  |  |  |  |  |  |  |  |  |  |  |  |
| Dapper homolog 2 | DACT2_HUMAN |  |  |  |  |  |  |  |  |  |  |  |  |  |  |  |  |  |  |  | x |
| D-dopachrome decarboxylase | DOPD_HUMAN |  |  | x |  |  |  |  | x |  |  |  |  |  |  | x |  |  |  |  |  |
| Deleted in malignant brain tumors 1 protein | DMBT1_HUMAN |  | x |  |  |  |  |  |  |  | x | x |  |  |  |  | x |  | x |  |  |
| Delta-aminolevulinic acid dehydratase | HEM2_HUMAN |  |  | x |  |  |  |  |  |  |  |  |  |  |  |  |  |  |  |  |  |
| DEP domain-containing protein 7 | DEPD7_HUMAN |  |  |  |  |  |  |  |  |  |  |  |  |  |  | x |  |  | x |  |  |
| Dermcidin | DCD_HUMAN | x | x |  |  |  |  |  |  |  |  |  |  |  |  |  |  |  | x |  |  |
| Dermokine | DMKN_HUMAN |  | x |  |  |  |  |  |  |  |  |  |  |  |  |  |  |  | x |  |  |
| Desmocollin-1 | DSC1_HUMAN | x |  |  |  |  |  |  |  |  |  |  |  |  |  | x |  |  | x |  |  |
| Desmocollin-2 | DSC2_HUMAN |  |  |  |  |  |  |  |  |  |  |  |  |  |  | x |  |  | x |  |  |
| Desmoglein-1 | DSG1_HUMAN | x |  |  |  |  |  |  |  |  |  |  |  |  |  | x |  |  | x |  |  |
| Desmoplakin | DESP_HUMAN | x |  |  |  |  |  |  |  |  |  |  |  |  |  | x |  | x |  | x |  |
| Diacylglycerol kinase delta | DGKD_HUMAN |  |  |  |  |  |  |  |  |  |  |  |  |  |  |  |  |  | x |  |  |
| Dickkopf-related protein 3 | DKK3_HUMAN |  | x |  |  |  |  |  |  |  |  |  |  |  |  |  |  |  | x |  |  |
| Dihydropteridine reductase | DHPR_HUMAN |  |  | x |  |  |  |  |  |  |  |  |  |  |  |  |  |  |  |  |  |
| Dipeptidyl peptidase 2 | DPP2_HUMAN |  |  | x | x |  |  |  |  |  |  |  |  |  |  |  |  |  |  |  |  |
| Dipeptidyl peptidase 3 | DPP3_HUMAN |  |  | x | x |  |  |  |  |  |  |  |  |  |  |  |  |  |  |  |  |
| DIS3-like exonuclease 2 | DI3L2_HUMAN |  |  | x |  |  |  |  |  |  |  |  |  |  |  | x |  |  |  |  |  |
| DNA damage-binding protein 1 | DDB1_HUMAN |  |  |  |  |  |  |  |  |  |  |  |  |  |  | x |  |  |  |  |  |
| DNA-directed RNA polymerase III subunit RPC2 | RPC2_HUMAN |  |  | x |  |  |  |  |  |  |  |  |  |  |  | x |  |  |  |  |  |
| DNA excision repair protein ERCC-6 | ERCC6_HUMAN |  |  | x |  |  |  |  |  |  |  |  |  |  |  |  |  |  |  |  |  |
| DNA repair protein RAD50 | RAD50_HUMAN |  |  |  |  |  |  |  |  |  |  |  |  |  |  | x |  |  |  |  |  |
| DNA replication licensing factor MCM6 | MCM6_HUMAN |  |  |  |  |  |  |  |  |  |  |  |  |  |  | x |  |  |  |  |  |
| DNA topoisomerase 2-beta | TOP2B_HUMAN |  |  | x |  |  |  |  |  |  |  |  |  |  |  | x |  |  |  |  |  |
| Doublesex- and mab-3-related transcription factor A1 | DMRTA_HUMAN |  |  |  |  |  |  |  |  |  |  |  |  |  |  | x |  |  |  |  |  |
| Double-strand break repair protein MRE11A | MRE11_HUMAN |  |  | x |  |  |  |  |  |  |  |  |  |  |  | x |  |  |  |  |  |
| Dynactin subunit 1 | DCTN1_HUMAN | x |  |  |  |  |  |  |  |  |  |  |  |  |  | x |  | x |  |  |  |
| Dynein heavy chain 3, axonemal | DYH3_HUMAN |  |  |  |  |  |  |  |  |  |  |  |  |  |  | x |  | x |  |  |  |
| Dynein heavy chain 10, axonemal | DYH10_HUMAN |  |  |  |  |  |  |  |  |  | x |  |  |  |  |  |  | x |  | x |  |
| Dynein heavy chain 11, axonemal | DYH11_HUMAN |  |  |  |  |  |  |  |  |  | x |  |  |  |  |  |  | x |  | x |  |
| Dynein heavy chain 2, axonemal | DYH2_HUMAN |  |  |  |  |  |  |  |  |  | x |  |  |  |  |  |  | x |  | x |  |
| Dynein heavy chain 5, axonemal | DYH5_HUMAN | x |  |  |  |  |  |  |  |  | x |  |  |  |  |  |  | x |  | x |  |
| Dynein heavy chain 6, axonemal | DYH6_HUMAN | x |  |  |  |  |  |  |  |  | x |  |  |  |  |  |  | x |  | x |  |
| Dynein heavy chain 17, axonemal | DYH17_HUMAN |  |  |  |  |  |  |  |  |  |  |  |  |  |  | x |  | x |  |  |  |
| Dynein heavy chain domain-containing protein 1 | DNHD1_HUMAN |  |  |  |  |  |  |  |  |  | x |  |  |  |  |  |  | x |  | x |  |
| Dystroglycan | DAG1_HUMAN | x | x |  |  |  |  |  |  |  |  | x |  |  |  |  |  | x | x |  |  |
| Dystonin | DYST_HUMAN |  |  |  |  |  |  |  |  |  |  |  |  |  |  | x |  | x |  |  |  |
| Dystrophin | DMD_HUMAN |  |  |  |  |  |  |  |  |  |  |  |  |  |  |  |  | x | x |  |  |
| E3 ubiquitin-protein ligase DTX3L | DTX3L_HUMAN |  |  | x |  |  |  |  |  |  |  |  |  |  |  |  |  |  | x |  |  |
| E3 ubiquitin-protein ligase SHPRH | SHPRH_HUMAN |  |  | x |  |  |  |  |  |  |  |  |  |  |  | x |  |  |  |  |  |
| E3 ubiquitin-protein ligase UBR4 | UBR4_HUMAN |  |  | x |  |  |  |  |  |  |  |  |  |  |  | x |  | x |  |  |  |
| E3 ubiquitin-protein ligase RBBP6 | RBBP6_HUMAN |  |  | x |  |  |  |  |  |  |  |  |  |  |  |  |  |  |  |  |  |
| Early endosome antigen 1 | EEA1_HUMAN |  |  |  |  |  |  |  |  |  | x |  |  |  |  | x |  |  |  |  |  |
| Echinoderm microtubule-associated protein-like 2 | EMAL2_HUMAN |  |  |  |  |  |  |  |  |  |  |  |  |  |  |  |  | x |  |  |  |
| Ecto-NOX disulfide-thiol exchanger 1 | ENOX1_HUMAN |  | x |  |  |  |  |  |  |  |  |  |  |  |  | x |  |  |  |  |  |
| Ectonucleotide pyrophosphatase/phosphodiesterase family member 2 | ENPP2_HUMAN |  | x | x |  |  |  |  |  |  |  |  |  |  |  |  |  |  |  |  |  |
| EF-hand domain-containing protein C17orf57 | CQ057_HUMAN |  |  |  |  |  |  |  |  |  |  |  |  |  |  |  |  |  | x |  |  |
| EGF-containing fibulin-like extracellular matrix protein 1 | FBLN3_HUMAN | x | x |  |  |  |  |  |  |  |  |  |  | x |  | x |  |  | x | x |  |
| EGF-containing fibulin-like extracellular matrix protein 2 | FBLN4_HUMAN |  | x |  |  |  |  |  |  |  |  |  |  | x |  | x |  |  | x | x |  |
| ELKS/RAB6-interacting/CAST family member 1 | RB6I2_HUMAN | x |  |  |  |  |  |  |  |  | x |  |  |  |  | x |  |  |  |  |  |
| Elongation factor 1-alpha 1 | EF1A1_HUMAN | x |  |  |  |  |  |  |  |  |  |  |  |  |  | x |  |  | x |  |  |
| Elongation factor 2 | EF2_HUMAN |  |  |  |  |  |  |  |  |  |  |  |  |  |  | x |  |  | x |  |  |
| EMILIN-3 | EMIL3_HUMAN |  | x |  |  |  |  |  |  |  |  |  |  |  |  |  |  |  | x | x |  |
| Endoplasmin | ENPL_HUMAN |  |  |  |  |  |  |  |  |  |  |  |  |  |  | x |  |  | x |  |  |
| Endoplasmic reticulum aminopeptidase 1 | ERAP1_HUMAN |  |  | x | x |  |  |  |  |  |  |  |  |  |  |  |  |  |  |  |  |
| Engulfment and cell motility protein 1 | ELMO1_HUMAN |  |  |  |  |  |  |  |  |  |  |  |  |  |  | x |  | x |  |  |  |
| Enhancer of mRNA-decapping protein 4 | EDC4_HUMAN |  |  |  |  |  |  |  |  |  |  |  |  |  |  |  |  |  |  |  | x |
| Enolase-phosphatase E1 | ENOPH_HUMAN |  |  | x |  |  |  |  |  |  |  |  |  |  |  | x |  |  |  |  |  |
| Epididymal secretory protein E1 | NPC2_HUMAN |  | x |  |  |  |  |  |  |  |  |  |  |  |  |  |  |  | x |  |  |
| Epoxide hydrolase 2 | HYES_HUMAN |  |  | x |  |  |  |  |  |  |  |  |  |  |  |  |  |  |  |  |  |
| Erythrocyte band 7 integral membrane protein | STOM_HUMAN |  |  |  |  |  |  |  |  |  |  |  |  |  |  |  |  | x |  | x |  |
| Eukaryotic initiation factor 4A-I | IF4A1_HUMAN |  |  | x |  |  |  |  |  |  |  |  |  |  |  | x |  |  |  |  |  |
| Eukaryotic initiation factor 4A-II | IF4A2_HUMAN |  |  | x |  |  |  |  |  |  |  |  |  |  |  | x |  |  |  |  |  |
| Eukaryotic translation initiation factor 2 subunit 1 | IF2A_HUMAN |  |  |  |  |  |  |  |  |  |  |  |  |  |  | x |  |  |  |  |  |
| Excitatory amino acid transporter 1 | EAA1_HUMAN |  |  |  |  |  |  |  |  |  | x |  |  |  |  |  |  |  | x |  |  |
| Exportin-2 | XPO2_HUMAN |  |  |  |  |  |  |  |  |  | x |  |  |  |  | x |  |  |  |  |  |
| Exportin-T | XPOT_HUMAN |  |  |  |  |  |  |  |  |  |  |  |  |  |  | x |  |  |  |  |  |
| Extracellular matrix protein 1 | ECM1_HUMAN |  | x |  |  |  |  |  |  |  |  |  |  |  |  |  |  |  | x |  |  |
| Extracellular superoxide dismutase [Cu-Zn] | SODE_HUMAN |  | x | x |  |  |  |  |  |  |  |  |  |  |  |  |  |  |  |  |  |
| F-actin-capping protein subunit alpha-1 | CAZA1_HUMAN |  |  |  |  |  |  |  |  |  |  |  |  |  |  | x |  | x |  | x |  |
| F-actin-capping protein subunit alpha-2 | CAZA2_HUMAN |  |  |  |  |  |  |  |  |  |  |  |  |  |  | x |  | x |  | x |  |
| F-actin-capping protein subunit beta | CAPZB_HUMAN |  |  |  |  |  |  |  |  |  |  |  |  |  |  | x |  | x |  | x |  |
| Far upstream element-binding protein 2 | FUBP2_HUMAN |  |  |  |  |  |  |  |  |  | x |  |  |  |  |  |  |  |  |  |  |
| Farnesyl pyrophosphate synthase | FPPS_HUMAN |  |  | x |  |  |  |  |  |  |  |  |  |  |  | x |  |  |  |  |  |
| Fas-binding factor 1 | FBF1_HUMAN |  |  |  |  |  |  |  |  |  |  |  |  |  |  |  |  |  |  |  | x |
| Fatty acid synthase | FAS_HUMAN |  |  | x |  |  |  |  |  |  |  |  |  |  |  |  |  |  |  |  |  |
| Fatty acid-binding protein, epidermal | FABP5_HUMAN |  |  |  |  |  |  |  |  |  | x |  |  |  |  | x |  |  | x |  |  |
| Ferritin heavy chain | FRIH_HUMAN | x |  |  |  |  |  |  |  |  | x |  |  |  |  | x |  |  |  |  |  |
| Ferritin light chain | FRIL_HUMAN | x |  |  |  |  |  |  |  |  | x |  |  |  |  | x |  |  |  |  |  |
| Fetuin-B | FETUB_HUMAN | x | x |  |  |  |  |  |  |  |  |  |  |  |  | x |  |  |  |  |  |
| Fibrillin-1 | FBN1_HUMAN |  | x |  |  |  |  |  |  |  |  |  |  | x |  | x |  |  | x | x |  |
| Fibrillin-2 | FBN2_HUMAN |  | x |  |  |  |  |  |  |  |  |  |  | x |  | x |  |  | x | x |  |
| Fibrinogen alpha chain | FIBA_HUMAN | x | x |  |  |  | x |  |  |  | x |  |  |  |  | x |  |  | x | X |  |
| Fibrinogen beta chain | FIBB_HUMAN | x | x |  |  |  | x |  |  |  | x |  |  |  |  | x |  |  | x | X |  |
| Fibrinogen gamma chain | FIBG_HUMAN | x | x |  |  |  | x |  |  |  | x |  |  |  |  | x |  |  | x | x |  |
| Fibronectin | FINC_HUMAN | x | x |  |  |  |  |  |  |  | x |  |  |  |  | x |  |  | x | x |  |
| Fibulin-1 | FBLN1_HUMAN |  | x |  |  |  |  |  |  |  |  | x |  |  |  | x |  |  |  | x |  |
| Filaggrin | FILA_HUMAN |  |  |  |  |  |  |  |  |  |  |  |  |  |  | x |  |  |  | x |  |
| Filaggrin-2 | FILA2_HUMAN |  |  |  |  |  |  |  |  |  |  |  |  |  |  | x |  |  |  | x |  |
| Filamin-A | FLNA_HUMAN | x |  |  |  |  |  |  |  |  |  |  |  |  |  |  |  | x |  |  |  |
| Filamin-B | FLNB_HUMAN | x |  |  |  |  |  |  |  |  |  |  |  |  |  | x |  | x |  |  |  |
| Filensin | BFSP1_HUMAN |  |  |  |  |  |  |  |  |  |  |  | x | x |  |  |  | x |  | x |  |
| Follistatin-related protein 1 | FSTL1_HUMAN |  | x |  |  |  |  |  |  |  |  |  |  |  |  | x |  |  |  |  |  |
| Follistatin-related protein 4 | FSTL4_HUMAN |  | x |  |  |  |  |  |  |  |  |  |  |  |  | x |  |  |  |  |  |
| Follistatin-related protein 5 | FSTL5_HUMAN |  | x |  |  |  |  |  |  |  |  |  |  |  |  | x |  |  |  |  |  |
| Four and a half LIM domains protein 1 | FHL1_HUMAN |  |  |  |  |  |  |  |  |  |  |  |  |  |  | x |  |  |  | x |  |
| Fructose-bisphosphate aldolase A | ALDOA_HUMAN | x |  | x |  |  |  |  |  |  |  |  |  |  |  |  | x |  |  |  |  |
| Fructose-bisphosphate aldolase C | ALDOC_HUMAN |  |  | x |  |  |  |  |  |  |  |  |  |  |  |  |  | x |  |  |  |
| Fumarylacetoacetase | FAAA_HUMAN |  |  | x |  |  |  |  |  |  |  |  |  |  |  | x |  |  |  |  |  |
| FYVE and coiled-coil domain-containing protein 1 | FYCO1_HUMAN |  |  |  |  |  |  |  |  |  |  |  |  |  |  | x |  |  |  |  |  |
| G2/mitotic-specific cyclin-B3 | CCNB3_HUMAN |  |  |  |  |  |  |  |  |  |  |  |  |  |  | x |  |  |  |  |  |
| Galectin-1 | LEG1_HUMAN |  | x |  |  |  |  |  |  |  |  |  |  |  |  | x | x |  |  |  |  |
| Galectin-3-binding protein | LG3BP_HUMAN |  | x |  |  |  |  |  |  |  | x | x |  |  |  |  | x |  | x |  |  |
| Galectin-7 | LEG7_HUMAN | x | x |  |  |  |  |  |  |  |  |  |  |  |  | x | x |  |  |  |  |
| Gamma-crystallin A | CRGA_HUMAN |  |  |  |  |  |  |  |  |  |  |  |  | x |  |  |  |  |  |  |  |
| Gamma-crystallin B | CRGB_HUMAN |  |  |  |  |  |  |  |  |  |  |  | x | x |  |  |  |  |  |  |  |
| Gamma-crystallin C | CRGC_HUMAN |  |  |  |  |  |  |  |  |  |  |  | x | x |  |  |  |  |  |  |  |
| Gamma-crystallin D | CRGD_HUMAN |  |  |  |  |  |  |  |  |  |  |  | x |  |  |  |  |  |  |  |  |
| Gamma-enolase | ENOG_HUMAN |  |  | x |  |  |  |  |  |  |  |  |  |  |  | x |  |  |  |  |  |
| Gamma-glutamyl hydrolase | GGH_HUMAN | x | x | x |  |  |  |  |  |  |  |  |  |  |  |  |  |  |  |  |  |
| Ganglioside GM2 activator | SAP3_HUMAN |  |  |  |  |  |  |  |  |  |  |  |  |  |  |  |  |  | x |  |  |
| Gap junction alpha-3 protein | CXA3_HUMAN |  |  |  |  |  |  |  |  |  |  |  |  |  | x |  |  |  | x |  |  |
| Gap junction alpha-8 protein | CXA8_HUMAN |  |  |  |  |  |  |  |  |  |  |  |  |  | x |  |  |  | x |  |  |
| Gasdermin-A | GSDMA_HUMAN |  |  |  |  |  |  |  |  |  |  |  |  |  |  |  | x |  |  |  |  |
| Gelsolin | GELS_HUMAN | x | x |  |  |  |  |  |  |  |  |  |  |  |  | x |  | x |  | x |  |
| General transcription factor IIE subunit 1 | T2EA_HUMAN |  |  |  |  |  |  |  |  |  |  |  |  |  |  | x |  |  |  |  |  |
| Geranylgeranyl pyrophosphate synthase | GGPPS_HUMAN |  |  | x |  |  |  |  |  |  |  |  |  | x |  |  |  |  | x |  |  |
| Geranylgeranyl transferase type-2 subunit alpha | PGTA_HUMAN |  |  | x |  |  |  |  |  |  |  |  |  |  |  |  |  |  | x |  |  |
| Geranylgeranyl transferase type-2 subunit beta | PGTB2_HUMAN |  |  | x |  |  |  |  |  |  |  |  |  |  |  |  |  |  | x |  |  |
| Girdin | GRDN_HUMAN |  |  |  |  |  |  |  |  |  | x |  |  |  |  | x |  |  | x |  |  |
| Glia maturation factor beta | GMFB_HUMAN |  |  |  |  |  |  |  |  |  |  |  |  |  |  | x |  |  |  |  |  |
| Glucosamine-6-phosphate isomerase 1 | GNPI1_HUMAN |  |  | x |  |  |  |  |  |  |  |  |  |  |  |  |  |  |  |  |  |
| Glucosamine-6-phosphate isomerase 2 | GNPI2_HUMAN |  |  | x |  |  |  |  |  |  |  |  |  |  |  |  |  |  |  |  |  |
| Glucosamine--fructose-6-phosphate aminotransferase [isomerizing] 2 | GFPT2_HUMAN |  |  | x |  |  |  |  |  |  |  |  |  |  |  |  |  |  |  |  |  |
| Glucose-6-phosphate 1-dehydrogenase | G6PD_HUMAN |  |  | x |  |  |  |  |  |  |  |  |  |  |  |  |  | x |  |  |  |
| Glucose-6-phosphate isomerase | G6PI_HUMAN | x | x | x |  |  |  |  |  |  |  |  |  |  |  |  |  |  |  |  |  |
| Glucosidase 2 subunit beta | GLU2B_HUMAN |  |  |  |  |  |  |  |  |  |  |  |  |  |  |  |  |  | x |  |  |
| Glutamate dehydrogenase 1, mitochondrial | DHE3_HUMAN |  |  | x |  |  |  |  |  |  |  |  |  |  |  |  |  |  |  |  |  |
| Glutamate [NMDA] receptor subunit epsilon-2 | NMDE2_HUMAN |  |  |  |  |  |  |  |  |  | x |  |  |  |  |  |  |  | x |  |  |
| Glutamate receptor 4 | GRIA4_HUMAN |  |  |  |  |  |  |  |  |  | x |  |  |  | x |  |  | x | x |  |  |
| Glutaminyl-peptide cyclotransferase | QPCT_HUMAN |  |  | x |  |  |  |  |  |  |  |  |  |  |  |  |  |  |  |  |  |
| Glutathione peroxidase 3 | GPX3_HUMAN | x | x | x |  |  |  |  |  |  |  |  |  |  |  |  |  |  |  |  |  |
| Glutathione reductase, mitochondrial | GSHR_HUMAN | x |  | x |  |  |  |  |  |  |  |  |  |  |  |  | x |  |  |  |  |
| Glutathione S-transferase Mu 2 | GSTM2_HUMAN |  |  | x |  |  |  |  |  |  |  |  |  |  |  |  |  |  |  |  |  |
| Glutathione S-transferase Mu 3 | GSTM3_HUMAN | x |  | x |  |  |  |  |  |  |  |  |  |  |  |  |  |  |  |  |  |
| Glutathione S-transferase P | GSTP1_HUMAN | x |  | x |  |  |  |  |  |  |  |  |  |  |  |  |  |  |  |  |  |
| Glutathione synthetase | GSHB_HUMAN | x |  | x |  |  |  |  |  |  |  |  |  |  |  | x |  |  |  |  |  |
| Glyceraldehyde-3-phosphate dehydrogenase | G3P_HUMAN | x |  | x |  |  |  |  |  |  |  |  |  |  |  |  |  |  |  |  |  |
| Glyceraldehyde-3-phosphate dehydrogenase. testis-specific | G3PT_HUMAN | x |  | x |  |  |  |  |  |  |  |  |  |  |  |  |  |  |  |  |  |
| Glycerol-3-phosphate dehydrogenase [NAD+], cytoplasmic | GPDA_HUMAN |  |  | x |  |  |  |  |  |  |  |  |  |  |  |  |  |  |  |  |  |
| Glycerol-3-phosphate dehydrogenase 1-like protein | GPD1L_HUMAN |  |  | x |  |  |  |  |  |  |  |  |  |  |  |  |  |  |  |  |  |
| Glycogen phosphorylase, brain form | PYGB_HUMAN |  |  | x |  |  |  |  |  |  |  |  |  |  |  |  |  |  |  |  |  |
| Glycogen phosphorylase, muscle form | PYGM_HUMAN | x |  | x |  |  |  |  |  |  |  |  |  |  |  |  |  |  |  |  |  |
| Glycyl-tRNA synthetase | SYG_HUMAN |  |  | x |  |  |  |  |  |  |  |  |  |  |  |  |  |  |  |  |  |
| Glyoxalase domain-containing protein 4 | GLOD4_HUMAN |  |  |  |  |  |  |  |  |  |  |  |  |  |  |  |  |  |  |  | x |
| Glyoxylate reductase/hydroxypyruvate reductase | GRHPR_HUMAN |  |  |  |  |  |  |  |  |  |  |  |  |  |  |  |  |  |  |  | x |
| GMP reductase 2 | GMPR2_HUMAN |  |  | x |  |  |  |  |  |  |  |  |  |  |  |  |  |  | x |  |  |
| Golgi resident protein GCP60 | GCP60_HUMAN |  |  |  |  |  |  |  |  |  |  |  |  |  |  |  |  |  |  |  | X |
| Golgin subfamily A member 1 | GOGA1_HUMAN |  |  |  |  |  |  |  |  |  |  |  |  |  |  |  |  |  |  |  | X |
| Golgin subfamily A member 3 | GOGA3_HUMAN |  |  |  |  |  |  |  |  |  |  |  |  |  |  |  |  |  |  |  | x |
| Golgin subfamily B member 1 | GOGB1_HUMAN | x |  |  |  |  |  |  |  |  | x |  |  |  |  | x |  |  |  | x |  |
| Golgi-specific brefeldin A-resistance guanine nucleotide exchange factor 1 | GBF1_HUMAN |  |  |  |  |  |  |  |  |  |  |  |  |  |  | x |  |  |  |  |  |
| GTP-binding nuclear protein Ran | RAN_HUMAN |  |  |  |  |  |  |  |  |  | x |  |  |  |  | x |  |  | x |  |  |
| Guanine nucleotide-binding protein G(I)/G(S)/G(T) subunit beta-1 | GBB1_HUMAN |  |  |  |  |  |  |  |  |  |  |  |  |  |  | x |  |  | x |  |  |
| Guanine nucleotide-binding protein G(s) subunit alpha isoforms XLas | GNAS1_HUMAN |  |  |  |  |  |  |  |  |  |  |  |  |  |  | x |  |  | x |  |  |
| Guanine nucleotide-binding protein subunit beta-2-like 1 | GBLP_HUMAN |  |  |  |  |  |  |  |  |  | x |  |  |  |  |  |  |  | x |  |  |
| Haloacid dehalogenase-like hydrolase domain-containing protein 2 | HDHD2_HUMAN |  |  | x |  |  |  |  |  |  |  |  |  |  |  | x |  |  |  |  |  |
| Haptoglobin | HPT_HUMAN | x | x |  |  |  |  |  |  |  |  |  |  |  |  | x |  |  |  |  |  |
| HEAT repeat-containing protein 1 | HEAT1_HUMAN |  |  |  |  |  |  |  |  |  |  |  |  |  |  | x |  |  |  |  |  |
| HAUS augmin-like complex subunit 5 | HAUS5_HUMAN |  |  |  |  |  |  |  |  |  |  |  |  |  |  |  |  | x |  |  |  |
| Heat shock 70 kDa protein 4 | HSP74_HUMAN |  |  |  |  |  |  |  |  |  |  |  |  |  |  |  |  |  |  |  | x |
| Heat shock cognate 71 kDa protein | HSP7C_HUMAN | x |  |  |  |  |  |  |  |  |  |  |  |  |  | x | x |  | x |  |  |
| Heat shock protein beta-1 | HSPB1_HUMAN |  |  |  |  |  |  |  |  |  |  |  | x | x |  |  |  | x |  |  |  |
| Heat shock protein HSP 90-alpha | HS90A_HUMAN | x |  |  |  |  |  |  |  |  |  |  |  |  |  |  |  |  |  |  | x |
| Heat shock protein HSP 90-beta | HS90B_HUMAN |  |  |  |  |  |  |  |  |  |  |  |  |  |  |  |  |  |  |  | x |
| Helicase-like transcription factor | HLTF_HUMAN | x |  |  |  |  |  |  |  |  |  |  |  |  |  | x |  |  |  |  |  |
| Heme-binding protein 2 | HEBP2_HUMAN |  |  |  |  |  |  |  |  |  |  |  |  |  |  |  |  |  |  |  | x |
| Hemicentin-1 | HMCN1_HUMAN | x | x |  |  |  |  |  |  |  |  |  |  |  |  |  |  |  |  | x |  |
| Hemoglobin subunit alpha | HBA_HUMAN | x |  |  |  |  |  |  |  |  | x |  |  |  |  |  |  |  |  |  |  |
| Hemoglobin subunit beta | HBB_HUMAN | x |  |  |  |  |  |  |  |  |  |  |  |  |  | x |  |  |  |  |  |
| Hemopexin | HEMO_HUMAN | x | x |  |  |  |  |  |  |  | x |  |  |  |  |  |  |  |  |  |  |
| Heparin cofactor 2 | HEP2_HUMAN | x |  |  |  | x | x |  |  |  |  |  |  |  |  | x |  |  |  |  |  |
| Hepatocyte growth factor activator | HGFA_HUMAN | x | x |  | x |  |  |  |  |  |  |  |  |  |  | x |  |  |  |  |  |
| Histidine-rich glycoprotein | HRG_HUMAN | x | x |  |  |  |  |  |  |  |  |  |  |  |  |  |  |  |  |  |  |
| Histidyl-tRNA synthetase, cytoplasmic | SYHC_HUMAN |  |  | x |  |  |  |  |  |  |  |  |  |  |  | x |  |  |  |  |  |
| Histone deacetylase 11 | HDA11_HUMAN |  |  | x |  |  |  |  |  |  |  |  |  |  |  | x | x |  |  |  |  |
| Histone H2A type 1-B/E | H2A1B_HUMAN |  |  |  |  |  |  |  |  |  |  |  |  |  |  | x |  |  |  |  |  |
| Histone H2B type 1-B | H2B1B_HUMAN |  |  |  |  |  |  |  |  |  |  |  |  |  |  | x |  |  |  |  |  |
| Histone H2B type 1-D | H2B1D_HUMAN |  |  |  |  |  |  |  |  |  |  |  |  |  |  | x |  |  |  |  |  |
| Histone H4 | H4_HUMAN | x |  |  |  |  |  |  |  |  |  |  |  |  |  | x |  |  |  |  |  |
| Histone-lysine N-methyltransferase MLL4 | MLL4_HUMAN | x |  | x |  |  |  |  |  |  |  |  |  |  |  | x |  |  |  |  |  |
| Histone-lysine N-methyltransferase ASH1L | ASH1L_HUMAN |  |  |  |  |  |  |  |  |  |  |  |  |  |  | x |  |  |  |  |  |
| Homeobox protein cut-like 1 | CUX1_HUMAN |  |  |  |  |  |  |  |  |  |  |  |  |  |  | x |  |  |  |  |  |
| Hornerin | HORN_HUMAN | x |  |  |  |  |  |  |  |  |  |  |  |  |  |  |  | x |  | x |  |
| Hyaluronan-binding protein 2 | HABP2_HUMAN | x | x |  | x |  |  | x |  |  |  |  |  |  |  | x | x |  | x |  |  |
| Hydroxyacylglutathione hydrolase, mitochondrial | GLO2_HUMAN |  | x | x |  |  |  |  |  |  |  |  |  |  |  | x |  |  |  |  |  |
| Iduronate 2-sulfatase | IDS_HUMAN |  |  | x |  |  |  |  |  |  |  |  |  |  |  |  |  |  | x |  |  |
| Ig alpha-1 chain C region | IGHA1_HUMAN | x |  |  |  |  |  |  |  |  |  |  |  |  |  | x |  |  |  |  |  |
| Ig alpha-2 chain C region | IGHA2_HUMAN | x |  |  |  |  |  |  |  |  |  |  |  |  |  | x |  |  |  |  |  |
| Ig gamma-1 chain C region | IGHG1_HUMAN | x |  |  |  |  |  |  |  |  |  |  |  |  |  | x |  |  |  |  |  |
| Ig gamma-2 chain C region | IGHG2_HUMAN | x | x |  |  |  |  |  |  |  |  |  |  |  |  | x |  |  |  |  |  |
| Ig gamma-3 chain C region | IGHG3_HUMAN | x | x |  |  |  |  |  |  |  |  |  |  |  |  | x |  |  |  |  |  |
| Ig gamma-4 chain C region | IGHG4_HUMAN | x | x |  |  |  |  |  |  |  |  |  |  |  |  | x |  |  |  |  |  |
| Ig heavy chain V-II region WAH | HV206_HUMAN | x |  |  |  |  |  |  |  |  |  |  |  |  |  | x |  |  |  |  |  |
| Ig heavy chain V-III region BRO | HV305_HUMAN | x |  |  |  |  |  |  |  |  |  |  |  |  |  | x |  |  |  |  |  |
| Ig heavy chain V-III region BUT | HV306_HUMAN | x |  |  |  |  |  |  |  |  |  |  |  |  |  | x |  |  |  |  |  |
| Ig heavy chain V-III region CAM | HV307_HUMAN | x |  |  |  |  |  |  |  |  |  |  |  |  |  | x |  |  |  |  |  |
| Ig heavy chain V-III region GA | HV308_HUMAN | x |  |  |  |  |  |  |  |  |  |  |  |  |  | x |  |  |  |  |  |
| Ig heavy chain V-III region TIL | HV304_HUMAN | x |  |  |  |  |  |  |  |  |  |  |  |  |  | x |  |  |  |  |  |
| Ig heavy chain V-III region TUR | HV318_HUMAN | x |  |  |  |  |  |  |  |  |  |  |  |  |  | x |  |  |  |  |  |
| Ig heavy chain V-III region VH26 | HV303_HUMAN | x |  |  |  |  |  |  |  |  |  |  |  |  |  | x |  |  |  |  |  |
| Ig heavy chain V-III region WEA | HV302_HUMAN | x |  |  |  |  |  |  |  |  |  |  |  |  |  | x |  |  |  |  |  |
| Ig kappa chain C region | IGKC_HUMAN | x |  |  |  |  |  |  |  |  |  |  |  |  |  | x |  |  |  |  |  |
| Ig kappa chain V-I region AG | KV101_HUMAN | x |  |  |  |  |  |  |  |  |  |  |  |  |  | x |  |  |  |  |  |
| Ig kappa chain V-I region DEE | KV105_HUMAN | x |  |  |  |  |  |  |  |  |  |  |  |  |  | x |  |  |  |  |  |
| Ig kappa chain V-I region EU | KV106_HUMAN | x |  |  |  |  |  |  |  |  |  |  |  |  |  | x |  |  |  |  |  |
| Ig kappa chain V-I region HK102 | KV110_HUMAN | x |  |  |  |  |  |  |  |  |  |  |  |  |  | x |  |  | x |  |  |
| Ig kappa chain V-I region Gal | KV107_HUMAN | x |  |  |  |  |  |  |  |  |  |  |  |  |  | x |  |  |  |  |  |
| Ig kappa chain V-I region Hau | KV108_HUMAN | x |  |  |  |  |  |  |  |  |  |  |  |  |  | x |  |  |  |  |  |
| Ig kappa chain V-I region OU | KV114_HUMAN | x |  |  |  |  |  |  |  |  |  |  |  |  |  | x |  |  |  |  |  |
| Ig kappa chain V-I region WEA | KV118_HUMAN | x |  |  |  |  |  |  |  |  |  |  |  |  |  | x |  |  |  |  |  |
| Ig kappa chain V-I region CAR | KV104_HUMAN | x |  |  |  |  |  |  |  |  |  |  |  |  |  | x |  |  |  |  |  |
| Ig kappa chain V-II region MIL | KV203_HUMAN | x |  |  |  |  |  |  |  |  |  |  |  |  |  | x |  |  |  |  |  |
| Ig kappa chain V-II region TEW | KV204_HUMAN | x |  |  |  |  |  |  |  |  |  |  |  |  |  | x |  |  |  |  |  |
| Ig kappa chain V-II region RPMI 6410 | KV206_HUMAN | x |  |  |  |  |  |  |  |  |  |  |  |  |  | x |  |  | x |  |  |
| Ig kappa chain V-III region SIE | KV302_HUMAN | x |  |  |  |  |  |  |  |  |  |  |  |  |  | x |  |  |  |  |  |
| Ig kappa chain V-III region NG9 | KV303_HUMAN | x |  |  |  |  |  |  |  |  |  |  |  |  |  | x |  |  | x |  |  |
| Ig kappa chain V-III region POM | KV306_HUMAN | x |  |  |  |  |  |  |  |  |  |  |  |  |  | x |  |  |  |  |  |
| Ig kappa chain V-III region VG (Fragment) | KV309_HUMAN | x |  |  |  |  |  |  |  |  |  |  |  |  |  | x |  |  |  |  |  |
| Ig kappa chain V-III region VH (Fragment) | KV310_HUMAN | x |  |  |  |  |  |  |  |  |  |  |  |  |  | x |  |  |  |  |  |
| Ig kappa chain V-III region WOL | KV305_HUMAN | x |  |  |  |  |  |  |  |  |  |  |  |  |  | x |  |  |  |  |  |
| Ig kappa chain V-IV region (Fragment) | KV401_HUMAN | x |  |  |  |  |  |  |  |  |  |  |  |  |  | x |  |  |  |  |  |
| Ig kappa chain V-IV region Len | KV402_HUMAN | x |  |  |  |  |  |  |  |  |  |  |  |  |  | x |  |  |  |  |  |
| Ig lambda chain V region 4A | LV001_HUMAN | x |  |  |  |  |  |  |  |  |  |  |  |  |  | x |  |  |  |  |  |
| Ig lambda chain V-I region HA | LV102_HUMAN | x |  |  |  |  |  |  |  |  |  |  |  |  |  | x |  |  |  |  |  |
| Ig lambda chain V-I region NIG-64 | LV104_HUMAN | x |  |  |  |  |  |  |  |  |  |  |  |  |  | x |  |  |  |  |  |
| Ig lambda chain V-I region WAH | LV106_HUMAN | x |  |  |  |  |  |  |  |  |  |  |  |  |  | x |  |  |  |  |  |
| Ig lambda chain V-III region LOI | LV302_HUMAN | x |  |  |  |  |  |  |  |  |  |  |  |  |  | x |  |  |  |  |  |
| Ig lambda chain V-III region SH | LV301_HUMAN | x |  |  |  |  |  |  |  |  |  |  |  |  |  | x |  |  |  |  |  |
| Ig lambda chain V-IV region Hil | LV403_HUMAN | x |  |  |  |  |  |  |  |  |  |  |  |  |  | x |  |  |  |  |  |
| Ig lambda-1 chain C regions | LAC1_HUMAN | x |  |  |  |  |  |  |  |  |  |  |  |  |  | x |  |  |  |  |  |
| Ig lambda-2 chain C regions | LAC2_HUMAN | x |  |  |  |  |  |  |  |  |  |  |  |  |  | x |  |  |  |  |  |
| Ig mu chain C region | IGHM_HUMAN | x |  |  |  |  |  |  |  |  |  |  |  |  |  | x |  |  |  |  |  |
| IgGFc-binding protein | FCGBP_HUMAN | x | x |  |  |  |  |  |  |  |  |  |  |  |  | x |  |  |  |  |  |
| Immunoglobulin lambda-like polypeptide 5 | IGLL5_HUMAN | x | x |  |  |  |  |  |  |  |  |  |  |  |  |  |  |  | x |  |  |
| Importin subunit beta-1 | IMB1_HUMAN |  |  |  |  |  |  |  |  |  | x |  |  |  |  |  |  |  |  |  |  |
| Importin-5 | IPO5_HUMAN |  |  |  |  |  |  |  |  |  | x |  |  |  |  | x |  |  |  |  |  |
| Importin-7 | IPO7_HUMAN |  |  |  |  |  |  |  |  |  | x |  |  |  |  | x |  |  |  |  |  |
| Inactive dual specificity phosphatase 27 | DUS27_HUMAN |  |  | x |  |  |  |  |  |  |  |  |  |  |  |  |  |  |  |  |  |
| InaD-like protein | INADL_HUMAN | x |  |  |  |  |  |  |  |  | x |  |  |  |  |  |  |  |  | x |  |
| Inosine-5~-monophosphate dehydrogenase 1 | IMDH1_HUMAN |  |  | x |  |  |  |  |  |  |  |  |  |  |  |  |  |  |  |  |  |
| Inosine-5~-monophosphate dehydrogenase 2 | IMDH2_HUMAN |  |  | x |  |  |  |  |  |  |  |  |  |  |  |  |  |  |  |  |  |
| Inositol 1,4,5-trisphosphate receptor type 1 | ITPR1_HUMAN | x |  |  |  |  |  |  |  |  | x |  |  |  | x |  |  | x | x |  |  |
| Inositol 1.4.5-trisphosphate receptor type 3 | ITPR3_HUMAN |  |  |  |  |  |  |  |  |  | x | x |  |  |  |  |  |  |  |  |  |
| Inositol monophosphatase 1 | IMPA1_HUMAN |  |  | x |  |  |  |  |  |  |  |  |  |  |  |  |  |  |  |  |  |
| Insulin-like growth factor-binding protein 7 | IBP7_HUMAN |  | x |  |  |  |  |  |  |  |  |  |  |  |  |  |  |  | x |  |  |
| Insulin-like growth factor-binding protein complex acid labile subunit | ALS_HUMAN | x | x |  |  |  |  |  |  |  |  | x |  |  |  |  |  |  | x |  |  |
| Integrator complex subunit 1 | INT1_HUMAN |  |  |  |  |  |  |  |  |  |  |  |  |  |  |  |  |  |  |  | x |
| Integrator complex subunit 3 | INT3_HUMAN |  |  |  |  |  |  |  |  |  |  |  |  |  |  |  |  |  |  |  | x |
| Integrator complex subunit 6 | INT6_HUMAN |  |  |  |  |  |  |  |  |  |  |  |  |  |  | x |  | x |  |  |  |
| Integrator complex subunit 7 | INT7_HUMAN |  |  |  |  |  |  |  |  |  |  |  |  |  |  |  |  |  |  |  | x |
| Inter-alpha-trypsin inhibitor heavy chain H1 | ITIH1_HUMAN | x | x |  |  | x |  |  |  |  |  |  |  |  |  | x |  |  |  |  |  |
| Inter-alpha-trypsin inhibitor heavy chain H2 | ITIH2_HUMAN | x | x |  |  | x |  |  |  |  |  |  |  |  |  | x |  |  |  |  |  |
| Inter-alpha-trypsin inhibitor heavy chain H3 | ITIH3_HUMAN | x | x |  |  | x |  |  |  |  |  |  |  |  |  | x |  |  |  |  |  |
| Inter-alpha-trypsin inhibitor heavy chain H4 | ITIH4_HUMAN | x | x |  |  | x |  |  |  |  |  |  |  |  |  | x |  |  |  |  |  |
| Inter-alpha-trypsin inhibitor heavy chain H5 | ITIH5_HUMAN |  | x |  |  | x |  |  |  |  |  |  |  |  |  | x |  |  |  |  |  |
| Interleukin-6 receptor subunit beta | IL6RB_HUMAN |  | x |  |  |  |  |  |  |  |  |  |  |  |  | x |  |  | x |  |  |
| Intersectin-1 | ITSN1_HUMAN |  |  |  |  |  |  |  |  |  |  |  |  |  |  | x |  |  |  |  |  |
| Intersectin-2 | ITSN2_HUMAN |  |  |  |  |  |  |  |  |  |  |  |  |  |  | x |  |  |  |  |  |
| Intraflagellar transport protein 88 homolog | IFT88_HUMAN |  |  |  |  |  |  |  |  |  |  |  |  |  |  |  |  | x | x |  |  |
| Intron-binding protein aquarius | AQR_HUMAN |  |  |  |  |  |  |  |  |  |  |  |  |  |  | x |  |  |  |  |  |
| Inversin | INVS_HUMAN |  |  |  |  |  |  |  |  |  |  |  |  |  |  |  |  | x | x |  |  |
| IQ domain-containing protein E | IQCE_HUMAN |  |  |  |  |  |  |  |  |  |  |  |  |  |  |  |  |  |  |  | x |
| Isoamyl acetate-hydrolyzing esterase 1 homolog | IAH1_HUMAN |  |  | x |  |  |  |  |  |  |  |  |  |  |  |  |  |  |  |  |  |
| Isochorismatase domain-containing protein 1 | ISOC1_HUMAN |  |  | x |  |  |  |  |  |  |  |  |  |  |  |  |  |  |  |  |  |
| Isocitrate dehydrogenase [NADP] cytoplasmic | IDHC_HUMAN | x |  | x |  |  |  |  |  |  |  |  |  |  |  |  |  |  |  |  |  |
| Isoleucyl-tRNA synthetase, cytoplasmic | SYIC_HUMAN |  |  | x |  |  |  |  |  |  |  |  |  |  |  |  |  |  |  |  |  |
| Janus kinase and microtubule-interacting protein 1 | JKIP1_HUMAN |  |  |  |  |  |  |  |  |  |  |  |  |  |  |  |  |  |  |  | x |
| JmjC domain-containing protein 7 | JMJD7_HUMAN |  |  |  |  |  |  |  |  |  |  |  |  |  |  |  |  |  |  |  | x |
| Junction plakoglobin | PLAK_HUMAN | x |  |  |  |  |  |  |  |  | x |  |  |  |  | x |  | x | x | x |  |
| Kallistatin | KAIN_HUMAN | x | x |  |  | x |  |  |  |  |  |  |  |  |  | x |  |  |  |  |  |
| Kinesin heavy chain isoform 5C | KIF5C_HUMAN | x |  |  |  |  |  |  |  |  | x |  |  |  |  |  |  | x |  | x |  |
| Kelch-like protein 9 | KLHL9_HUMAN |  |  | x |  |  |  |  |  |  |  |  |  |  |  | x |  |  |  |  |  |
| Keratin. type I cuticular Ha1 | K1H1_HUMAN | x |  |  |  |  |  |  |  |  |  |  |  |  |  |  |  | x |  |  |  |
| Keratin. type I cuticular Ha2 | K1H2_HUMAN |  |  |  |  |  |  |  |  |  |  |  |  |  |  |  |  | x |  |  |  |
| Keratin. type I cuticular Ha3-I | KT33A_HUMAN |  |  |  |  |  |  |  |  |  |  |  |  |  |  |  |  | x |  |  |  |
| Keratin. type I cuticular Ha3-II | KT33B_HUMAN | x |  |  |  |  |  |  |  |  |  |  |  |  |  |  |  | x |  |  |  |
| Keratin. type I cuticular Ha4 | KRT34_HUMAN |  |  |  |  |  |  |  |  |  |  |  |  |  |  |  |  | x |  |  |  |
| Keratin. type I cuticular Ha5 | KRT35_HUMAN | x |  |  |  |  |  |  |  |  |  |  |  |  |  |  |  | x |  |  |  |
| Keratin. type I cuticular Ha6 | KRT36_HUMAN | x |  |  |  |  |  |  |  |  |  |  |  |  |  |  |  | x |  |  |  |
| Keratin. type I cytoskeletal 10 | K1C10_HUMAN | x |  |  |  |  |  |  |  |  |  |  |  |  |  |  |  | x |  |  |  |
| Keratin. type I cytoskeletal 12 | K1C12_HUMAN |  |  |  |  |  |  |  |  |  |  |  |  |  |  |  |  | x |  |  |  |
| Keratin. type I cytoskeletal 13 | K1C13_HUMAN | x |  |  |  |  |  |  |  |  |  |  |  |  |  |  |  | x |  |  |  |
| Keratin. type I cuticular Ha1 | K1H1_HUMAN |  |  |  |  |  |  |  |  |  |  |  |  |  |  |  |  | x |  |  |  |
| Keratin. type I cuticular Ha2 | K1H2_HUMAN |  |  |  |  |  |  |  |  |  |  |  |  |  |  |  |  | x |  |  |  |
| Keratin. type I cuticular Ha3-I | KT33A_HUMAN |  |  |  |  |  |  |  |  |  |  |  |  |  |  |  |  | x |  |  |  |
| Keratin. type I cuticular Ha3-II | KT33B_HUMAN |  |  |  |  |  |  |  |  |  |  |  |  |  |  |  |  | x |  |  |  |
| Keratin. type I cuticular Ha4 | KRT34_HUMAN |  |  |  |  |  |  |  |  |  |  |  |  |  |  |  |  | x |  |  |  |
| Keratin. type I cuticular Ha5 | KRT35_HUMAN |  |  |  |  |  |  |  |  |  |  |  |  |  |  |  |  | x |  |  |  |
| Keratin. type I cuticular Ha6 | KRT36_HUMAN |  |  |  |  |  |  |  |  |  |  |  |  |  |  |  |  | x |  |  |  |
| Keratin. type I cytoskeletal 14 | K1C14_HUMAN | x |  |  |  |  |  |  |  |  |  |  |  |  |  |  | x |  |  |  |  |
| Keratin. type I cytoskeletal 15 | K1C15_HUMAN | x |  |  |  |  |  |  |  |  |  |  |  |  |  |  | x |  |  |  |  |
| Keratin. type I cytoskeletal 16 | K1C16_HUMAN | x |  |  |  |  |  |  |  |  |  |  |  |  |  |  | x |  |  |  |  |
| Keratin. type I cytoskeletal 17 | K1C17_HUMAN |  |  |  |  |  |  |  |  |  |  |  |  |  |  |  | x |  |  |  |  |
| Keratin. type I cytoskeletal 18 | K1C18_HUMAN |  |  |  |  |  |  |  |  |  |  |  |  |  |  |  | x |  |  |  |  |
| Keratin. type I cytoskeletal 19 | K1C19_HUMAN |  |  |  |  |  |  |  |  |  |  |  |  |  |  |  | x |  |  |  |  |
| Keratin. type I cytoskeletal 20 | K1C20_HUMAN |  |  |  |  |  |  |  |  |  |  |  |  |  |  |  | x |  |  |  |  |
| Keratin. type I cytoskeletal 25 | K1C25_HUMAN |  |  |  |  |  |  |  |  |  |  |  |  |  |  |  | x |  |  |  |  |
| Keratin. type I cytoskeletal 28 | K1C28_HUMAN |  |  |  |  |  |  |  |  |  |  |  |  |  |  |  | x |  |  |  |  |
| Keratin. type I cytoskeletal 9 | K1C9_HUMAN | x |  |  |  |  |  |  |  |  |  |  |  |  |  |  | x |  |  |  |  |
| Keratin. type II cuticular Hb1 | KRT81_HUMAN |  |  |  |  |  |  |  |  |  |  |  |  |  |  |  | x |  |  |  |  |
| Keratin. type II cuticular Hb2 | KRT82_HUMAN |  |  |  |  |  |  |  |  |  |  |  |  |  |  |  | x |  |  |  |  |
| Keratin. type II cuticular Hb3 | KRT83_HUMAN |  |  |  |  |  |  |  |  |  |  |  |  |  |  |  | x |  |  |  |  |
| Keratin. type II cuticular Hb4 | KRT84_HUMAN |  |  |  |  |  |  |  |  |  |  |  |  |  |  |  | x |  |  |  |  |
| Keratin. type II cuticular Hb5 | KRT85_HUMAN |  |  |  |  |  |  |  |  |  |  |  |  |  |  |  | x |  |  |  |  |
| Keratin. type II cuticular Hb6 | KRT86_HUMAN |  |  |  |  |  |  |  |  |  |  |  |  |  |  |  | x |  |  |  |  |
| Keratin. type II cytoskeletal 1 | K2C1_HUMAN |  |  |  |  |  |  |  |  |  |  |  |  |  |  |  | x |  |  |  |  |
| Keratin. type II cytoskeletal 1b | K2C1B_HUMAN |  |  |  |  |  |  |  |  |  |  |  |  |  |  |  | x |  |  |  |  |
| Keratin. type II cytoskeletal 2 epidermal | K22E_HUMAN | x |  |  |  |  |  |  |  |  |  |  |  |  |  |  | x |  |  |  |  |
| Keratin. type II cytoskeletal 2 oral | K22O_HUMAN |  |  |  |  |  |  |  |  |  |  |  |  |  |  |  | x |  |  |  |  |
| Keratin. type II cytoskeletal 3 | K2C3_HUMAN |  |  |  |  |  |  |  |  |  |  |  |  |  |  |  | x |  |  |  |  |
| Keratin. type II cytoskeletal 4 | K2C4_HUMAN | x |  |  |  |  |  |  |  |  |  |  |  |  |  |  | x |  |  |  |  |
| Keratin. type II cytoskeletal 5 | K2C5_HUMAN | x |  |  |  |  |  |  |  |  |  |  |  |  |  |  | x |  |  |  |  |
| Keratin. type II cytoskeletal 6A | K2C6A_HUMAN |  |  |  |  |  |  |  |  |  |  |  |  |  |  |  | x |  |  |  |  |
| Keratin. type II cytoskeletal 6B | K2C6B_HUMAN |  |  |  |  |  |  |  |  |  |  |  |  |  |  |  | x |  |  |  |  |
| Keratin. type II cytoskeletal 6C | K2C6C_HUMAN |  |  |  |  |  |  |  |  |  |  |  |  |  |  |  | x |  |  |  |  |
| Keratin. type II cytoskeletal 7 | K2C7_HUMAN |  |  |  |  |  |  |  |  |  |  |  |  |  |  |  | x |  |  |  |  |
| Keratin. type II cytoskeletal 71 | K2C71_HUMAN |  |  |  |  |  |  |  |  |  |  |  |  |  |  |  | x |  |  |  |  |
| Keratin. type II cytoskeletal 72 | K2C72_HUMAN |  |  |  |  |  |  |  |  |  |  |  |  |  |  |  | x |  |  |  |  |
| Keratin. type II cytoskeletal 73 | K2C73_HUMAN |  |  |  |  |  |  |  |  |  |  |  |  |  |  |  | x |  |  |  |  |
| Keratin. type II cytoskeletal 78 | K2C78_HUMAN |  |  |  |  |  |  |  |  |  |  |  |  |  |  |  | x |  |  |  |  |
| Keratin. type II cytoskeletal 79 | K2C79_HUMAN |  |  |  |  |  |  |  |  |  |  |  |  |  |  |  | x |  |  |  |  |
| Keratin. type II cytoskeletal 8 | K2C8_HUMAN |  |  |  |  |  |  |  |  |  |  |  |  |  |  |  | x |  |  |  |  |
| Keratin. type II cytoskeletal 80 | K2C80_HUMAN |  |  |  |  |  |  |  |  |  |  |  |  |  |  |  |  | x |  |  |  |
| Keratin-81-like protein | KT81L_HUMAN |  |  |  |  |  |  |  |  |  |  |  |  |  |  |  |  | x |  |  |  |
| Keratin-associated protein 13-1 | KR131_HUMAN |  |  |  |  |  |  |  |  |  |  |  |  |  |  |  |  | x |  |  |  |
| Keratin-associated protein 13-2 | KR132_HUMAN |  |  |  |  |  |  |  |  |  |  |  |  |  |  |  |  | x |  |  |  |
| Keratin-associated protein 3-1 | KRA31_HUMAN |  |  |  |  |  |  |  |  |  |  |  |  |  |  |  |  | x |  |  |  |
| Keratinocyte proline-rich protein | KPRP_HUMAN |  |  |  |  |  |  |  |  |  |  |  |  |  |  |  |  | x |  |  |  |
| Kinesin-1 heavy chain | KINH_HUMAN | x |  |  |  |  |  |  |  |  | x |  |  |  |  |  |  | x |  | x |  |
| Kinesin-like protein KIF1B | KIF1B_HUMAN |  |  |  |  |  |  |  |  |  | x |  |  |  |  |  |  | x |  | x |  |
| Kininogen-1 | KNG1_HUMAN | x | x |  |  | x | x |  |  |  |  |  |  |  |  | x |  |  |  |  |  |
| Kynurenine--oxoglutarate transaminase 1 | KAT1_HUMAN |  |  |  |  |  |  |  |  |  |  |  |  |  |  | x |  |  |  |  |  |
| Lactase-like protein | LCTL_HUMAN |  |  | x |  |  |  |  |  |  |  |  |  |  |  | x |  |  | x |  |  |
| Lactotransferrin | TRFL_HUMAN | x | x |  | x |  |  |  |  |  | x |  |  |  |  |  |  |  |  |  |  |
| Lactoylglutathione lyase | LGUL_HUMAN |  |  | x |  |  |  |  |  |  |  |  |  |  |  |  |  |  |  |  |  |
| Lambda-crystallin homolog | CRYL1_HUMAN |  |  | x |  |  |  |  |  |  |  |  |  |  |  | x |  |  |  |  |  |
| Laminin subunit alpha-1 | LAMA1_HUMAN | x | x |  |  |  |  |  |  |  |  |  |  |  |  |  |  |  | x | X |  |
| Laminin subunit alpha-2 | LAMA2_HUMAN | x | x |  |  |  |  |  |  |  |  |  |  |  |  |  |  |  |  | X |  |
| Laminin subunit alpha-5 | LAMA5_HUMAN | x | x |  |  |  |  |  |  |  |  |  |  |  |  |  |  |  |  | x |  |
| Laminin subunit beta-3 | LAMB3_HUMAN | x | x |  |  |  |  |  |  |  |  |  |  |  |  |  |  |  | x | x |  |
| Laminin subunit gamma-1 | LAMC1_HUMAN | x | x |  |  |  |  |  |  |  |  |  |  |  |  |  |  |  | x | x |  |
| Latent-transforming growth factor beta-binding protein 2 | LTBP2_HUMAN |  | x |  |  |  |  |  |  |  |  | x |  |  |  | x |  |  | x | x |  |
| Lebercilin-like protein | LCA5L_HUMAN | x | x | x |  |  |  |  |  |  |  |  |  |  |  |  |  |  | x |  |  |
| Lengsin | LGSN_HUMAN |  |  |  |  |  |  |  |  |  |  |  |  |  |  |  |  |  |  |  | x |
| Lens fiber major intrinsic protein | MIP_HUMAN |  |  |  |  |  |  |  |  |  | x |  |  |  |  |  |  |  |  |  |  |
| Lens fiber membrane intrinsic protein | LMIP_HUMAN |  |  |  |  |  |  |  |  |  | x |  |  | x |  |  | x |  |  | x |  |
| Leucine-rich alpha-2-glycoprotein | A2GL_HUMAN | x | x |  |  |  |  |  |  |  |  | x |  |  |  |  |  |  | x | x |  |
| Leucine-rich repeat and coiled-coil domain-containing protein 1 | LRCC1_HUMAN | x |  |  |  |  |  |  |  |  |  |  |  |  |  | x |  | x |  | x |  |
| Leucine-rich repeat and fibronectin type-III domain-containing protein 2 | LRFN2_HUMAN |  |  |  |  |  |  |  |  |  |  |  |  |  |  |  |  |  | x | x |  |
| Leucine-rich repeat and guanylate kinase domain-containing protein | LRGUK_HUMAN |  |  | x |  |  |  |  |  |  |  |  |  |  |  | x |  |  |  | x |  |
| Leucine-rich repeat serine/threonine-protein kinase 2 | LRRK2_HUMAN |  |  |  |  |  |  |  |  |  |  |  |  |  |  |  |  |  |  |  | x |
| Leucine-rich repeat and IQ domain-containing protein 3 | LRIQ3_HUMAN |  |  |  |  |  |  |  |  |  |  |  |  |  |  | x |  |  |  | x |  |
| Leucine-rich repeat-containing protein 8C | LRC8C_HUMAN |  |  |  |  |  |  |  |  |  |  |  |  |  |  |  |  |  |  |  | x |
| Leucine-rich repeat-containing protein 15 | LRC15_HUMAN | x |  |  |  |  |  |  |  |  |  | x |  |  |  |  |  |  | x | x |  |
| Leucine-rich repeat transmembrane protein FLRT3 | FLRT3_HUMAN |  |  |  |  |  |  |  |  |  |  |  |  |  |  | x |  |  | x | x |  |
| Leucine zipper protein 2 | LUZP2_HUMAN |  | x |  |  |  |  |  |  |  |  |  |  |  |  |  |  |  | x |  |  |
| Leukocyte elastase inhibitor | ILEU_HUMAN |  |  |  |  | x |  |  |  |  |  |  |  |  |  | x |  |  |  |  |  |
| LIM and cysteine-rich domains protein 1 | LMCD1_HUMAN |  |  |  |  |  |  |  |  |  |  |  | x |  |  |  |  |  |  | x |  |
| Limbic system-associated membrane protein | LSAMP_HUMAN | x |  |  |  |  |  |  |  |  |  |  |  |  |  | x |  |  | x |  |  |
| Lipocalin-1 | LCN1_HUMAN |  | x |  |  |  |  |  |  |  | x |  |  |  |  |  |  |  | x |  |  |
| Lipopolysaccharide-binding protein | LBP_HUMAN | x | x |  |  |  |  |  |  |  | x |  |  |  |  |  |  |  |  |  |  |
| Liver carboxylesterase 1 | EST1_HUMAN |  |  | x |  |  |  |  |  | x |  |  |  |  |  | x |  |  | x |  |  |
| L-lactate dehydrogenase A chain | LDHA_HUMAN | x |  | x |  |  |  |  |  |  |  |  |  |  |  | x |  |  |  |  |  |
| L-lactate dehydrogenase B chain | LDHB_HUMAN | x |  | x |  |  |  |  |  |  |  |  |  |  |  |  |  |  |  |  |  |
| L-lactate dehydrogenase C chain | LDHC_HUMAN |  |  | x |  |  |  |  |  |  |  |  |  |  |  |  |  |  |  |  |  |
| Long-chain-fatty-acid--CoA ligase 6 | ACSL6_HUMAN | x |  | x |  |  |  |  |  |  |  |  |  |  |  | x |  |  |  |  |  |
| Low-density lipoprotein receptor-related protein 2 | LRP2_HUMAN |  |  |  |  |  |  |  |  |  |  | x |  |  |  |  |  |  |  | x |  |
| Lumican | LUM_HUMAN | x | x |  |  |  |  |  |  |  |  | x |  | x |  |  |  |  | x | x |  |
| Lysine-specific demethylase 5B | KDM5B_HUMAN |  |  |  |  |  |  |  |  |  |  |  |  |  |  | x |  |  |  |  |  |
| Lysine-specific demethylase 5D | KDM5D_HUMAN |  |  | x |  |  |  |  |  |  |  |  |  |  |  | x |  |  |  |  |  |
| Lysosomal-trafficking regulator | LYST_HUMAN |  |  |  |  |  |  |  |  |  | x |  |  |  |  | x |  | x | x |  |  |
| Lysosome-associated membrane glycoprotein 2 | LAMP2_HUMAN | x |  |  |  |  |  |  |  |  | x |  |  |  |  |  |  |  |  |  |  |
| Lysozyme C | LYSC_HUMAN | x | x | x |  |  |  |  |  |  |  |  |  |  |  |  |  |  | x |  |  |
| Macrophage colony-stimulating factor 1 receptor | CSF1R_HUMAN | x |  | x |  |  |  |  |  |  |  | x |  |  |  |  |  |  |  |  |  |
| Macrophage migration inhibitory factor | MIF_HUMAN |  | x |  |  |  |  |  | x |  |  |  |  |  |  | x |  |  |  |  |  |
| Major vault protein | MVP_HUMAN |  |  |  |  |  |  |  |  |  |  |  |  |  |  | x |  |  |  |  |  |
| Malate dehydrogenase, cytoplasmic | MDHC_HUMAN |  |  | x |  |  |  |  |  |  |  |  |  |  |  |  |  |  |  |  |  |
| Malate dehydrogenase, mitochondrial | MDHM_HUMAN |  |  | x |  |  |  |  |  |  |  |  |  |  |  |  |  |  |  |  |  |
| MAP/microtubule affinity-regulating kinase 3 | MARK3_HUMAN |  |  |  |  |  |  |  |  |  |  |  |  |  |  | x |  |  | x | x |  |
| Mediator of RNA polymerase II transcription subunit 16 | MED16_HUMAN |  |  |  |  |  |  |  |  |  |  |  |  |  |  |  |  |  |  |  | x |
| Mast cell carboxypeptidase A | CBPA3_HUMAN |  |  |  | x |  |  |  |  |  |  |  |  |  |  | x |  |  | x |  |  |
| Matrix Metalloproteinase 2 | MMP2_HUMAN | x | x | x | x |  |  |  |  |  |  |  |  |  | x |  |  |  |  | x |  |
| Metalloproteinase inhibitor 1 | TIMP1_HUMAN | x | x | x |  |  |  |  |  |  |  |  |  |  |  | x |  |  |  |  |  |
| Metalloproteinase inhibitor 2 | TIMP2_HUMAN |  | x | x |  |  |  |  |  |  |  |  |  |  |  | x |  |  |  |  |  |
| Metastasis-associated protein MTA2 | MTA2_HUMAN |  |  |  |  |  |  |  |  |  |  |  |  |  |  | x |  |  |  |  |  |
| Methylosome protein 50 | MEP50_HUMAN |  |  |  |  |  |  |  |  |  | x | x |  |  |  |  |  |  |  |  |  |
| Microfibril-associated glycoprotein 4 | MFAP4_HUMAN |  | x |  |  |  |  |  |  |  | x |  |  |  |  | x |  |  | x | x |  |
| Microtubule-actin cross-linking factor 1, isoforms 1/2/3/5 | MACF1_HUMAN | x |  |  |  |  |  |  |  |  |  |  |  |  |  |  |  | x |  |  |  |
| Microtubule-associated protein 1A | MAP1A_HUMAN | x |  |  |  |  |  |  |  |  |  |  |  |  |  |  |  | x |  |  |  |
| Microtubule-associated protein 1B | MAP1B_HUMAN | x |  |  |  |  |  |  |  |  |  |  | x |  |  |  |  | x |  |  |  |
| Microtubule-associated protein RP/EB family member 1 | MARE1_HUMAN |  |  |  |  |  |  |  |  |  |  |  |  |  |  | x |  | x |  | x |  |
| Microtubule-associated tumor suppressor candidate 2 | MTUS2_HUMAN |  |  |  |  |  |  |  |  |  |  |  |  |  |  |  |  | x |  |  |  |
| Midasin | MDN1_HUMAN | x |  |  |  |  |  |  |  |  |  |  |  |  |  | x |  |  |  |  |  |
| Mis18-binding protein 1 | M18BP_HUMAN |  |  |  |  |  |  |  |  |  |  |  |  |  |  |  |  |  |  |  | x |
| Mitogen-activated protein kinase 3 | MK03_HUMAN |  |  | x |  |  |  |  |  |  |  |  |  |  |  |  |  | x | x |  |  |
| Moesin | MOES_HUMAN |  |  |  |  |  |  |  |  |  |  |  |  |  |  |  |  |  |  | x |  |
| Molybdopterin synthase sulfur carrier subunit | MOC2A_HUMAN |  |  | x |  |  |  |  |  |  |  |  |  |  |  |  |  |  |  |  |  |
| Monocyte differentiation antigen CD14 | CD14_HUMAN | x |  |  |  |  |  |  |  |  |  | x |  |  |  |  |  |  |  |  |  |
| Monoglyceride lipase | MGLL_HUMAN |  |  | x |  |  |  |  |  |  |  |  |  |  |  |  |  |  |  |  |  |
| Mortality factor 4-like protein 1 | MO4L1_HUMAN |  |  |  |  |  |  |  |  |  |  |  |  |  |  | x |  |  |  |  |  |
| Mucin-5B | MUC5B_HUMAN |  | x | x |  |  |  |  |  |  |  |  |  |  |  | x |  |  |  | x |  |
| Mucin-7 | MUC7_HUMAN |  | x |  |  |  |  |  |  |  |  |  |  |  |  | x |  |  |  |  |  |
| Multidrug resistance protein 3 | MDR3_HUMAN |  |  | x |  |  |  |  |  |  | x |  |  |  |  |  |  |  |  |  |  |
| Multifunctional protein ADE2 | PUR6_HUMAN | x |  |  |  |  |  |  |  |  |  |  |  |  |  |  |  |  |  |  |  |
| Multiple C2 and transmembrane domain-containing protein 2 | MCTP2_HUMAN |  |  |  |  |  |  |  |  |  |  |  |  |  |  |  |  |  |  |  | x |
| Multiple epidermal growth factor-like domains protein 8 | MEGF8_HUMAN | x |  |  |  |  |  |  |  |  |  |  |  |  |  |  |  |  | x | x |  |
| Myeloperoxidase | PERM_HUMAN |  |  | x |  |  |  |  |  |  |  |  |  |  |  |  |  |  |  |  |  |
| Myocilin | MYOC_HUMAN | x | x |  |  |  |  |  |  |  | x |  | x | x |  |  |  |  | x |  |  |
| Myomegalin | MYOME_HUMAN |  |  |  |  |  |  |  |  |  |  |  |  |  |  |  |  | x |  |  |  |
| Myosin regulatory light chain 2, skeletal muscle isoform | MLRS_HUMAN |  |  |  |  |  |  |  |  |  |  |  | x |  |  | x |  | x |  |  |  |
| Myosin-1 | MYH1_HUMAN | x |  |  |  |  |  |  |  |  |  |  |  |  |  | x |  | x | x | x |  |
| Myosin-10 | MYH10_HUMAN | x |  |  |  |  |  |  |  |  | x |  |  |  |  | x |  | x | x | x |  |
| Myosin-15 | MYH15_HUMAN | x |  |  |  |  |  |  |  |  |  |  |  |  |  | x |  | x | x | x |  |
| Myosin-2 | MYH2_HUMAN | x |  |  |  |  |  |  |  |  | x |  |  |  |  | x |  | x | x | x |  |
| Myosin-3 | MYH3_HUMAN |  |  |  |  |  |  |  |  |  |  |  |  |  |  | x |  | x | x | x |  |
| Myosin-7 | MYH7_HUMAN | x |  |  |  |  |  |  |  |  |  |  |  |  |  | x |  | x | x | x |  |
| Myosin-7B | MYH7B_HUMAN |  |  |  |  |  |  |  |  |  | x |  |  |  |  | x |  | x | x | x |  |
| Myosin-8 | MYH8_HUMAN |  |  |  |  |  |  |  |  |  | x |  |  |  |  | x |  | x | x | x |  |
| Myosin-9 | MYH9_HUMAN | x |  |  |  |  |  |  |  |  | x |  |  |  |  | x |  | x | x | x |  |
| Myosin-14 | MYH14_HUMAN |  |  |  |  |  |  |  |  |  |  |  |  |  |  | x |  |  |  | x |  |
| Myosin-Ia | MYO1A_HUMAN | x |  |  |  |  |  |  |  |  | x |  |  |  |  | x |  | x | x | x |  |
| Myosin-Ie | MYO1E_HUMAN |  |  |  |  |  |  |  |  |  | x |  |  |  |  | x |  | x | x | x |  |
| Myosin-IXb | MYO9B_HUMAN |  |  |  |  |  |  |  |  |  | x |  |  |  |  | x |  | x | x | x |  |
| Myosin-Vb | MYO5B_HUMAN |  |  |  |  |  |  |  |  |  | x |  |  |  |  |  |  | x | x | x |  |
| Myosin-Vc | MYO5C_HUMAN | x |  |  |  |  |  |  |  |  | x |  |  |  |  | x |  | x | x | x |  |
| Myosin-XVIIIa | MY18A_HUMAN | x |  |  |  |  |  |  |  |  | x |  |  |  |  | x |  | x | x | x |  |
| Myosin-XVIIIb | MY18B_HUMAN | x |  |  |  |  |  |  |  |  | x |  |  |  |  | x |  | x | x | x |  |
| Myotrophin | MTPN_HUMAN |  |  |  |  |  |  | x |  |  |  |  |  |  |  |  |  |  |  |  |  |
| Myotubularin-related protein 3 | MTMR3_HUMAN |  |  |  |  |  |  |  |  |  | x |  |  |  |  |  |  |  |  |  |  |
| N(G),N(G)-dimethylarginine dimethylaminohydrolase 1 | DDAH1_HUMAN |  |  | x |  |  |  |  |  |  |  |  |  |  |  |  |  |  |  |  |  |
| N(G),N(G)-dimethylarginine dimethylaminohydrolase 2 | DDAH2_HUMAN |  |  | x |  |  |  |  |  |  |  |  |  |  |  |  |  |  |  |  |  |
| N-acetyl-D-glucosamine kinase | NAGK_HUMAN |  |  | x |  |  |  |  |  |  |  |  |  |  |  |  |  |  |  |  |  |
| N-acetylglucosamine-6-sulfatase | GNS_HUMAN |  |  | x |  |  |  |  |  |  |  |  |  |  |  |  |  |  |  |  |  |
| N-acetyllactosaminide beta-1,3-N-acetylglucosaminyltransferase | B3GN1_HUMAN |  |  | x |  |  |  |  |  |  |  |  |  |  |  |  |  |  |  |  |  |
| N-acetylmuramoyl-L-alanine amidase | PGRP2_HUMAN |  | x | x |  |  |  |  |  |  |  |  |  |  |  | x | x |  |  |  |  |
| NACHT, LRR and PYD domains-containing protein 7 | NALP7_HUMAN |  |  |  |  |  |  |  |  |  |  |  |  |  |  | x | x |  |  |  |  |
| NACHT. LRR and PYD domains-containing protein 6 | NALP6_HUMAN |  |  |  |  |  |  |  |  |  |  |  |  |  |  | x | x |  |  |  |  |
| NADH dehydrogenase [ubiquinone] 1 alpha subcomplex subunit 11 | NDUAB_HUMAN |  |  | x |  |  |  |  |  |  |  |  |  |  |  |  |  |  |  |  |  |
| NADH dehydrogenase [ubiquinone] 1 alpha subcomplex subunit 13 | NDUAD_HUMAN |  |  | x |  |  |  |  |  |  |  |  |  |  |  |  | x |  |  |  |  |
| NADH dehydrogenase [ubiquinone] 1 subunit C2 | NDUC2_HUMAN |  |  | x |  |  |  |  |  |  |  |  |  |  |  |  |  |  |  |  |  |
| NADH-cytochrome b5 reductase 2 | NB5R2_HUMAN |  |  | x |  |  |  |  |  |  |  |  |  |  |  |  |  |  |  |  |  |
| NADH-cytochrome b5 reductase 3 | NB5R3_HUMAN |  |  | x |  |  |  |  |  |  |  |  |  |  |  |  |  |  |  |  |  |
| NADP-dependent malic enzyme | MAOX_HUMAN |  |  |  |  |  |  |  |  |  |  |  |  |  |  | x |  |  |  |  |  |
| Nebulin | NEBU_HUMAN | x |  |  |  |  |  |  |  |  |  |  |  |  |  | x |  | x |  | x |  |
| Negative elongation factor B | NELFB_HUMAN |  |  |  |  |  |  |  |  |  |  |  |  |  |  |  |  |  |  |  | x |
| Neogenin | NEO1_HUMAN | x |  |  |  |  |  |  |  |  |  | x |  |  |  |  | x |  | x |  |  |
| Nephrocystin-3 | NPHP3_HUMAN |  |  |  |  |  |  |  |  |  | x |  |  |  |  |  |  | x |  | x |  |
| Nesprin-1 | SYNE1_HUMAN | x |  |  |  |  |  |  |  |  |  |  |  |  |  |  |  | x |  |  |  |
| Nesprin-2 | SYNE2_HUMAN | x |  |  |  |  |  |  |  |  |  |  |  |  |  |  |  | x |  |  |  |
| Neural cell adhesion molecule 1 | NCAM1_HUMAN | x | x |  |  |  |  |  |  |  |  | x |  |  |  |  | x |  | x |  |  |
| Neural cell adhesion molecule L1-like protein | CHL1_HUMAN | x | x |  |  |  |  |  |  |  |  | x |  |  |  |  | x |  | x |  |  |
| Neurexin-3-alpha | NRX3A_HUMAN | x |  |  |  |  |  |  |  |  | x |  |  |  |  | x |  |  | x | x |  |
| Neuroblastoma breakpoint family member 3 | NBPF3_HUMAN |  |  |  |  |  |  |  |  |  |  |  |  |  |  |  |  |  |  |  | x |
| Neurofascin | NFASC_HUMAN |  |  |  |  |  |  |  |  |  |  | x |  |  |  |  | x |  | x |  |  |
| Neurofilament heavy polypeptide | NFH_HUMAN |  |  |  |  |  |  |  |  |  |  |  | x |  |  |  |  | x |  |  |  |
| Neurofilament light polypeptide | NFL_HUMAN |  |  |  |  |  |  |  |  |  |  |  | x |  |  |  |  | x |  | x |  |
| Neuroligin-4, X-linked | NLGNX_HUMAN |  |  |  |  |  |  |  |  | x |  |  |  |  |  | x |  |  | x |  |  |
| Neuronal cell adhesion molecule | NRCAM_HUMAN |  |  |  |  |  |  |  |  |  |  | x |  |  |  |  | x |  | x |  |  |
| Neuroserpin | NEUS_HUMAN |  | x |  |  | x |  |  |  |  |  |  |  |  |  | x |  |  |  |  |  |
| Neutrophil defensin 1 | DEF1_HUMAN |  |  |  |  |  |  |  |  |  |  |  |  |  |  |  |  | x |  |  |  |
| Neutrophil gelatinase-associated lipocalin | NGAL_HUMAN |  | x | x |  |  |  |  |  |  | x |  |  |  |  |  |  |  | x |  |  |
| Nestin | NEST_HUMAN |  |  |  |  |  |  |  |  |  |  |  |  |  |  |  |  | x |  |  |  |
| NF-kappa-B-repressing factor | NKRF_HUMAN |  |  |  |  |  |  |  |  |  |  |  |  |  |  | x |  |  |  |  |  |
| NIF3-like protein 1 | NIF3L_HUMAN |  |  |  |  |  |  |  |  |  |  |  |  |  |  |  |  |  |  |  | x |
| Ninein | NIN_HUMAN |  |  |  |  |  |  |  |  |  |  |  |  |  |  | x |  | x |  | x |  |
| Non-specific cytotoxic cell receptor protein 1 homolog | NCRP1_HUMAN |  |  |  |  |  |  |  |  |  |  | x |  |  |  |  |  |  |  |  |  |
| Nuclear receptor corepressor 2 | NCOR2_HUMAN | x |  |  |  |  |  |  |  |  |  |  |  |  |  | x |  |  |  |  |  |
| Nuclear transport factor 2 | NTF2_HUMAN |  |  |  |  |  |  |  |  |  | x |  |  |  |  |  |  |  |  |  |  |
| Nuclear mitotic apparatus protein 1 | NUMA1_HUMAN |  |  |  |  |  |  |  |  |  |  |  |  |  |  |  |  |  |  |  | x |
| Nucleolar GTP-binding protein 1 | NOG1_HUMAN |  |  |  |  |  |  |  |  |  |  |  |  |  |  | x |  |  |  |  |  |
| Nucleoside diphosphate kinase A | NDKA_HUMAN |  |  | x |  |  |  |  |  |  |  |  |  |  |  |  |  | x |  |  |  |
| Nucleoside diphosphate kinase B | NDKB_HUMAN |  |  | x |  |  |  |  |  |  |  |  |  |  |  | x |  |  |  |  |  |
| Nucleosome assembly protein 1-like 1 | NP1L1_HUMAN | x |  |  |  |  |  |  |  |  |  |  |  |  |  | x | x |  |  |  |  |
| Nucleosome assembly protein 1-like 4 | NP1L4_HUMAN |  |  |  |  |  |  |  |  |  |  |  |  |  |  | x | x |  |  |  |  |
| Obg-like ATPase 1 | OLA1_HUMAN |  |  | x |  |  |  |  |  |  |  |  |  |  |  | x |  |  |  |  |  |
| Obscurin | OBSCN_HUMAN | x |  | x |  |  |  |  |  |  |  |  |  |  |  |  | x |  |  |  |  |
| Oligodendrocyte-myelin glycoprotein | OMGP_HUMAN |  |  |  |  |  |  |  |  |  |  | x |  |  |  |  |  |  |  | x |  |
| Omega-amidase NIT2 | NIT2_HUMAN |  |  | x |  |  |  |  |  |  |  |  |  |  |  |  |  |  |  |  |  |
| Oncostatin-M | ONCM_HUMAN |  | x |  |  |  |  |  | x |  |  |  |  |  |  | x |  |  | x |  |  |
| Opticin | OPT_HUMAN |  | x |  |  |  |  |  |  |  |  | x |  |  |  |  |  |  | x | x |  |
| Oral-facial-digital syndrome 1 protein | OFD1_HUMAN |  |  |  |  |  |  |  |  |  |  |  |  |  |  |  |  | x |  |  |  |
| Osteoclast-stimulating factor 1 | OSTF1_HUMAN |  |  |  |  |  |  |  |  |  |  |  |  |  |  |  |  |  |  |  | x |
| Osteopontin | OSTP_HUMAN |  | x |  |  |  |  |  | x |  |  |  |  |  |  | x |  |  |  | x |  |
| Out at first protein homolog | OAF_HUMAN |  |  |  |  |  |  |  |  |  |  |  |  |  |  |  |  |  | x |  |  |
| Oxysterol-binding protein-related protein 7 | OSBL7_HUMAN |  |  |  |  |  |  |  |  |  | x |  |  |  |  |  |  |  |  |  |  |
| Palladin | PALLD_HUMAN |  |  |  |  |  |  |  |  |  |  |  |  |  |  |  |  | x |  | x |  |
| Pappalysin-2 4 | PAPP2_HUMAN | x | x |  | x |  |  |  |  |  |  |  |  |  |  |  |  |  | x |  |  |
| Paralemmin | PALM_HUMAN |  |  |  |  |  |  |  |  |  |  |  |  |  |  |  |  |  | x |  |  |
| Parathyroid hormone-related protein | PTHR_HUMAN |  | x |  |  |  |  |  |  | x |  |  |  |  |  |  |  |  |  |  |  |
| PDZ domain-containing protein 2 | PDZD2_HUMAN |  | x |  |  |  |  |  | x |  |  |  |  | x |  | x |  |  |  |  |  |
| Pecanex-like protein 2 | PCX2_HUMAN |  |  |  |  |  |  |  |  |  |  |  |  |  |  |  |  |  |  |  | x |
| Pecanex-like protein 3 | PCX3_HUMAN |  |  |  |  |  |  |  |  |  |  |  |  |  |  |  |  |  |  |  | x |
| Peptidase inhibitor 16 | PI16_HUMAN |  |  |  |  | x |  |  |  |  |  |  |  |  |  |  |  |  | x |  |  |
| Peptidyl-prolyl cis-trans isomerase A | PPIA_HUMAN |  |  | x |  |  |  |  |  |  | x |  |  |  |  |  |  |  |  |  |  |
| Peptidyl-prolyl cis-trans isomerase F, mitochondrial | PPIF_HUMAN |  |  | x |  |  |  |  |  |  | x |  |  |  |  |  |  |  |  |  |  |
| Peptidyl-prolyl cis-trans isomerase FKBP5 | FKBP5_HUMAN |  |  | x |  |  |  |  |  |  |  |  |  |  |  |  | x |  |  |  |  |
| Periaxin | PRAX_HUMAN |  |  |  |  |  |  |  |  |  |  |  |  |  |  |  |  |  |  |  | x |
| Perilipin-3 | PLIN3_HUMAN |  |  |  |  |  |  |  |  |  | x |  |  |  |  |  |  |  |  |  |  |
| Period circadian protein homolog 2 | PER2_HUMAN |  |  |  |  |  |  |  |  |  |  |  |  |  |  |  |  |  | x |  |  |
| Periostin | POSTN_HUMAN |  | x |  |  |  |  |  |  |  |  |  |  | x |  | x |  |  | x |  |  |
| Periplakin | PEPL_HUMAN | x |  |  |  |  |  |  |  |  |  |  |  |  |  | x |  |  |  | x |  |
| Peroxisomal NADH pyrophosphatase NUDT12 | NUD12_HUMAN |  |  | x |  |  |  |  |  |  |  |  |  |  |  | x |  |  |  |  |  |
| Peroxiredoxin-1 | PRDX1_HUMAN |  |  |  |  |  |  |  |  |  |  |  |  |  |  |  |  |  |  |  | x |
| Peroxiredoxin-2 | PRDX2_HUMAN | x |  | x |  |  |  |  |  |  |  |  |  |  |  | x |  |  |  |  |  |
| Peroxiredoxin-4 | PRDX4_HUMAN |  | x | x |  |  |  |  |  |  |  |  |  |  |  |  |  |  | x |  |  |
| Peroxiredoxin-5, mitochondrial | PRDX5_HUMAN |  |  | x |  |  |  |  |  |  |  |  |  |  |  | x |  |  |  |  |  |
| Peroxiredoxin-6 | PRDX6_HUMAN |  |  |  |  |  |  |  |  |  |  |  |  |  |  |  |  |  |  |  | x |
| Peroxisomal acyl-coenzyme A oxidase 2 | ACOX2_HUMAN |  |  | x |  |  |  |  |  |  |  |  |  |  |  | x |  |  |  |  |  |
| PERQ amino acid-rich with GYF domain-containing protein 1 | PERQ1_HUMAN | x |  |  |  |  |  |  |  |  |  |  |  |  |  |  |  |  |  |  | x |
| Phakinin | BFSP2_HUMAN | x |  |  |  |  |  |  |  |  |  |  | x |  |  |  |  | x |  | x |  |
| Phosphatidylinositol 4.5-bisphosphate 3-kinase catalytic subunit gamma isoform | PK3CG_HUMAN |  |  | x |  |  |  |  |  |  |  |  |  |  |  | x |  |  |  |  |  |
| Phosphatidylethanolamine-binding protein 1 | PEBP1_HUMAN |  |  |  |  | x |  |  |  |  |  |  |  |  |  | x |  |  | x |  |  |
| Phosphatidylethanolamine-binding protein 4 | PEBP4_HUMAN |  |  |  |  |  |  |  |  |  |  |  |  |  |  | x |  |  | x |  |  |
| Phosphoserine phosphatase | SERB_HUMAN |  |  | x |  |  |  |  |  |  |  |  |  |  |  | x |  |  |  |  |  |
| Phosphoglucomutase-like protein 5 | PGM5_HUMAN |  |  | x |  |  |  |  |  |  |  |  |  |  |  |  |  | x |  |  |  |
| Phosphoglycerate kinase 1 | PGK1_HUMAN |  |  | x |  |  |  |  |  |  |  |  |  |  |  | x |  |  |  |  |  |
| Phosphoglycerate kinase 2 | PGK2_HUMAN |  |  | x |  |  |  |  |  |  |  |  |  |  |  | x |  |  |  |  |  |
| Phosphoglycerate mutase 1 | PGAM1_HUMAN |  |  | x |  |  |  |  |  |  |  |  |  |  |  |  |  |  |  |  |  |
| Phosphoglycerate mutase 2 | PGAM2_HUMAN |  |  | x |  |  |  |  |  |  |  |  |  |  |  |  |  |  |  |  |  |
| Phospholipid transfer protein | PLTP_HUMAN | x | x |  |  |  |  |  |  |  |  |  |  |  |  |  |  |  | x |  |  |
| Phospholysine phosphohistidine inorganic pyrophosphate phosphatase | LHPP_HUMAN |  |  | x |  |  |  |  |  |  |  |  |  |  |  | x |  |  |  |  |  |
| Phosphorylase b kinase regulatory subunit beta | KPBB_HUMAN |  |  | x |  |  |  |  |  |  |  |  |  |  |  | x |  |  |  |  |  |
| Phosphopantothenate--cysteine ligase | PPCS_HUMAN |  |  | x |  |  |  |  |  |  |  |  |  |  |  |  |  |  |  |  |  |
| Pigment epithelium-derived factor | PEDF_HUMAN | x | x |  |  |  |  |  |  |  |  |  |  |  |  | x |  |  |  |  |  |
| PITH domain-containing protein 1 | PITH1_HUMAN |  |  |  |  |  |  |  |  |  |  |  |  |  |  |  |  |  |  |  | x |
| Plasma protease C1 inhibitor | IC1_HUMAN | x | x | x |  | x | x |  |  |  |  |  |  |  |  | x |  |  |  |  |  |
| Plasma serine protease inhibitor | IPSP_HUMAN | x | x | x |  | x | x |  |  |  |  |  |  |  |  | x |  |  |  |  |  |
| Plasmalemma vesicle-associated protein | PLVAP_HUMAN | x |  |  |  |  |  |  |  |  |  |  |  |  |  |  |  |  |  |  | x |
| Plasminogen | PLMN_HUMAN | x | x |  | x |  | x |  |  | x | x |  |  |  |  | x | x |  |  |  |  |
| Platelet-activating factor acetylhydrolase IB subunit alpha | LIS1_HUMAN |  |  | x |  |  |  |  |  |  |  |  |  |  |  | x |  | x |  |  |  |
| Pleckstrin homology domain-containing family H member 2 | PKHH2_HUMAN |  |  |  |  |  |  |  |  |  |  |  |  |  |  |  |  | x |  |  |  |
| Plectin | PLEC_HUMAN | x |  |  |  |  |  |  |  |  |  |  |  |  |  | x |  | x |  |  |  |
| Plexin domain-containing protein 1 | PXDC1_HUMAN |  | x |  |  |  |  |  |  |  |  |  |  |  |  |  |  |  |  |  |  |
| Plexin domain-containing protein 2 | PXDC2_HUMAN | x |  |  |  |  |  |  |  |  |  |  |  |  |  |  |  |  | x |  |  |
| Plexin-B2 | PLXB2_HUMAN |  |  |  |  |  |  |  |  |  |  |  |  |  |  | x |  |  | x |  |  |
| Poly(ADP-ribose) glycohydrolase ARH3 | ARHL2_HUMAN |  |  | x |  |  |  |  |  |  |  |  |  |  |  |  |  |  | x |  |  |
| Polyadenylate-binding protein 4 | PABP4_HUMAN |  |  |  |  |  |  |  |  |  |  |  |  |  |  | x |  |  | x |  |  |
| Polymeric immunoglobulin receptor | PIGR_HUMAN |  | x |  |  |  |  |  |  |  | x | x |  |  |  |  |  |  |  |  |  |
| PRAME family member 9/15 | PRAM9_HUMAN |  |  |  |  |  |  |  |  |  |  |  |  |  |  |  |  |  |  |  | x |
| Pre-mRNA-splicing factor ISY1 homolog | ISY1_HUMAN |  |  |  |  |  |  |  |  |  |  |  |  |  |  |  |  |  |  |  | x |
| Proactivator polypeptide | SAP_HUMAN |  |  |  |  |  |  |  |  |  | x |  |  |  |  | x |  |  |  |  |  |
| Probable ATP-dependent RNA helicase DDX23 | DDX23_HUMAN |  |  | x |  |  |  |  |  |  |  |  |  |  |  | x |  |  |  |  |  |
| Probable JmjC domain-containing histone demethylation protein 2C | JHD2C_HUMAN |  |  |  |  |  |  |  |  |  |  |  |  |  |  | x |  |  |  |  |  |
| Probable ATP-dependent RNA helicase DDX46 | DDX46_HUMAN |  |  | x |  |  |  |  |  |  |  |  |  |  |  | x |  |  |  |  |  |
| Probable ATP-dependent RNA helicase YTDC2 | YTDC2_HUMAN |  |  | x |  |  |  |  |  |  |  |  |  |  |  | x |  |  |  |  |  |
| Probable E3 ubiquitin-protein ligase MYCBP2 | MYCB2_HUMAN |  |  | x |  |  |  |  |  |  |  |  |  |  |  |  |  | x |  |  |  |
| Probable tRNA (uracil-O(2)-)-methyltransferase | TRM44_HUMAN |  |  | x |  |  |  |  |  |  |  |  |  |  |  |  |  |  |  |  |  |
| Procollagen-lysine,2-oxoglutarate 5-dioxygenase 3 | PLOD3_HUMAN |  |  | x |  |  |  |  |  |  |  |  |  |  |  |  |  |  |  |  |  |
| Profilin-1 | PROF1_HUMAN | x |  |  |  |  |  |  |  |  |  |  |  |  |  |  |  | x |  |  |  |
| Programmed cell death 6-interacting protein | PDC6I_HUMAN |  |  |  |  |  |  |  |  |  |  |  |  |  |  |  | x | x |  |  |  |
| Prolactin-inducible protein | PIP_HUMAN |  | x |  |  |  |  |  |  |  |  |  |  |  |  |  |  |  |  |  |  |
| Prolow-density lipoprotein receptor-related protein 1 | LRP1_HUMAN |  |  |  |  |  |  |  |  |  |  | x |  |  |  |  |  |  |  | x |  |
| Prolyl endopeptidase | PPCE_HUMAN |  |  | x | x |  |  |  |  |  |  |  |  |  |  |  |  |  |  |  |  |
| Prostaglandin-H2 D-isomerase | PTGDS_HUMAN | x | x | x |  |  |  |  |  |  | x |  |  |  |  |  |  |  | x |  |  |
| Proteasome assembly chaperone 2 | PSMG2_HUMAN |  |  |  |  |  |  |  |  |  |  |  |  |  |  |  |  |  | x |  |  |
| Proteasome inhibitor PI31 subunit | PSMF1_HUMAN | x |  |  |  |  |  |  |  |  |  |  |  |  |  | x |  |  |  |  |  |
| Proteasome subunit alpha type-1 | PSA1_HUMAN |  |  |  | x |  |  |  |  |  |  |  |  |  |  |  |  |  |  |  |  |
| Proteasome subunit alpha type-3 | PSA3_HUMAN |  |  |  | x |  |  |  |  |  |  |  |  |  |  |  |  | x |  |  |  |
| Proteasome subunit alpha type-4 | PSA4_HUMAN |  |  |  | x |  |  |  |  |  |  |  |  |  |  |  |  |  |  |  |  |
| Proteasome subunit alpha type-5 | PSA5_HUMAN |  |  |  | x |  |  |  |  |  |  |  |  |  |  |  |  |  |  |  |  |
| Proteasome subunit alpha type-6 | PSA6_HUMAN |  |  |  | x |  |  |  |  |  |  |  |  |  |  |  |  |  |  |  |  |
| Proteasome subunit alpha type-7 | PSA7_HUMAN | x |  |  | x |  |  |  |  |  |  |  |  |  |  |  |  |  |  |  |  |
| Proteasome subunit beta type-5 | PSB5_HUMAN |  |  |  | x |  |  |  |  |  |  |  |  |  |  |  |  |  |  |  |  |
| Proteasome subunit beta type-6 | PSB6_HUMAN |  |  |  | x |  |  |  |  |  |  |  |  |  |  |  |  |  |  |  |  |
| Protein 4.1 | 41_HUMAN |  |  |  |  |  |  |  |  |  |  |  |  |  |  |  |  |  |  |  | x |
| Protein AHNAK2 | AHNK2_HUMAN |  |  |  |  |  |  |  |  |  |  |  |  |  |  |  |  |  |  |  | x |
| Protein AMBP | AMBP_HUMAN | x | x |  |  | x |  |  |  |  |  |  |  |  |  | x |  |  |  |  |  |
| Protein CutA | CUTA_HUMAN |  |  |  |  |  |  |  |  |  | x |  |  |  |  |  |  |  |  |  |  |
| Protein diaphanous homolog 2 | DIAP2_HUMAN |  |  |  |  |  |  |  |  |  |  |  |  |  |  | x |  |  |  | x |  |
| Protein DJ-1 | PARK7_HUMAN |  |  |  |  |  |  |  |  |  |  |  |  |  |  | x |  |  |  |  |  |
| Protein ELYS | ELYS_HUMAN |  |  |  |  |  |  |  |  |  |  |  |  |  |  | x |  |  |  |  |  |
| Protein FAM3C | FAM3C_HUMAN |  | x |  |  |  |  |  |  |  |  |  |  |  |  |  |  |  |  |  |  |
| Protein FAM49B | FA49B_HUMAN |  |  |  |  |  |  |  |  |  |  |  |  |  |  |  |  |  |  |  | x |
| Protein FAM65A | FA65A_HUMAN |  |  |  |  |  |  |  |  |  |  |  |  |  |  |  |  |  |  |  | x |
| Protein furry homolog | FRY_HUMAN |  |  |  |  |  |  |  |  |  |  |  |  |  |  |  |  |  |  |  | x |
| Protein kinase C and casein kinase substrate in neurons protein 3 | PACN3_HUMAN |  |  | x |  |  |  |  |  |  |  |  |  |  |  |  |  |  | x |  |  |
| Protein NDRG1 | NDRG1_HUMAN |  |  |  |  |  |  |  |  |  |  |  |  |  |  |  |  | x |  |  |  |
| Protein NPAT | NPAT_HUMAN |  |  |  |  |  |  |  |  |  |  |  |  |  |  |  |  |  |  |  | x |
| Protein PML | PML_HUMAN |  |  |  |  |  |  |  |  |  |  |  |  |  |  | x |  |  |  |  |  |
| Protein MB21D2 | M21D2_HUMAN |  |  |  |  |  |  |  |  |  |  |  |  |  |  |  |  |  |  |  | x |
| Protein piccolo | PCLO_HUMAN |  |  |  |  |  |  |  |  |  |  |  |  |  |  | x |  |  | x |  |  |
| Protein S100-A4 | S10A4_HUMAN |  |  |  |  |  |  | x |  |  |  |  |  |  |  | x |  |  | x |  |  |
| Protein S100-A6 | S10A6_HUMAN |  |  |  |  |  |  | x |  |  |  |  |  |  |  | x |  |  | x |  |  |
| Protein S100-A7 | S10A7_HUMAN | x | x |  |  |  |  |  |  |  |  |  |  |  |  | x |  |  | x |  |  |
| Protein S100-A8 | S10A8_HUMAN | x | x |  |  |  |  |  |  |  |  |  |  |  |  | x |  |  | x |  |  |
| Protein S100-A9 | S10A9_HUMAN | x | x |  |  |  |  |  |  |  |  |  |  |  |  | x |  |  | x |  |  |
| Protein S100-B | S100B_HUMAN |  |  |  |  |  |  |  |  |  |  |  |  |  |  | x |  |  | x |  |  |
| Protein Shroom3 | SHRM3_HUMAN | x |  |  |  |  |  |  |  |  | x |  |  |  | x | x |  | x | x | x |  |
| Protein Spindly | SPDLY_HUMAN | x |  |  |  |  |  |  |  |  |  |  |  |  |  |  |  |  | x |  |  |
| Protein-tyrosine kinase 2-beta | FAK2_HUMAN |  |  | x |  |  |  |  |  |  |  |  |  |  |  | x | x |  | x |  |  |
| Protein unc-45 homolog A | UN45A_HUMAN |  |  |  |  |  |  |  |  |  |  |  |  |  |  |  |  |  |  |  | x |
| Protein XRP2 | XRP2_HUMAN |  |  |  |  |  |  |  |  |  |  |  |  |  |  |  |  |  |  |  | x |
| Protein-glutamine gamma-glutamyltransferase 2 | TGM2_HUMAN |  |  | x |  |  |  |  |  |  |  |  |  |  |  |  |  |  |  |  |  |
| Protein-glutamine gamma-glutamyltransferase E | TGM3_HUMAN | x |  | x |  |  |  |  |  |  |  |  |  |  |  |  |  |  |  |  |  |
| Protein-glutamine gamma-glutamyltransferase K | TGM1_HUMAN |  |  | x |  |  |  |  |  |  |  |  |  |  |  |  |  | x |  |  |  |
| Protein-L-isoaspartate(D-aspartate) O-methyltransferase | PIMT_HUMAN |  |  | x |  |  |  |  |  |  |  |  |  |  |  |  |  |  |  |  |  |
| Prothrombin | THRB_HUMAN | x | x |  | x |  | x |  |  |  |  |  |  |  |  | x |  |  |  |  |  |
| Protocadherin alpha-C2 | PCDC2_HUMAN |  | x |  |  |  |  |  |  |  |  | x |  |  |  | x |  |  | x |  |  |
| Protocadherin gamma-C3 | PCDGK_HUMAN |  |  |  |  |  |  |  |  |  |  |  |  |  |  |  |  |  | x |  |  |
| Pterin-4-alpha-carbinolamine dehydratase | PHS_HUMAN |  |  | x |  |  |  |  |  |  |  |  |  |  |  |  |  |  |  |  |  |
| Purine nucleoside phosphorylase | PNPH_HUMAN |  |  | x |  |  |  |  |  |  |  |  |  |  |  |  |  | x |  |  |  |
| Puromycin-sensitive aminopeptidase | PSA_HUMAN |  |  | x |  |  |  |  |  |  |  |  |  |  |  |  |  |  |  |  |  |
| Putative adenosylhomocysteinase 2 | SAHH2_HUMAN |  |  | x |  |  |  |  |  |  |  |  |  |  |  |  |  |  |  |  |  |
| Putative disintegrin and metalloproteinase domain-containing protein 5 | ADAM5_HUMAN |  |  |  |  |  |  |  |  |  |  |  |  |  |  |  | x |  | x |  |  |
| Putative phospholipase B-like 2 | PLBL2_HUMAN |  |  | x |  |  |  |  |  |  |  |  |  |  |  |  |  |  |  |  |  |
| Putative heat shock protein HSP 90-alpha A5 | HS905_HUMAN |  |  |  |  |  |  |  |  |  |  |  |  |  |  | x |  |  |  |  |  |
| Putative MAGE domain-containing protein MAGEA13P | MA13P_HUMAN |  |  |  |  |  |  |  |  |  |  |  |  |  |  |  |  |  |  |  | x |
| Putative protein PLEKHA9 | PKHA9_HUMAN |  |  |  |  |  |  |  |  |  | x |  |  |  |  |  |  |  |  |  |  |
| Putative RNA-binding protein 15 | RBM15_HUMAN |  |  |  |  |  |  |  |  |  |  |  |  |  |  | x |  |  |  |  |  |
| Putative tropomyosin alpha-3 chain-like protein | TPM3L_HUMAN |  |  |  |  |  |  |  |  |  |  |  |  |  |  |  |  |  |  | x |  |
| Putative ubiquitin carboxyl-terminal hydrolase 17-like protein 1 | U17L1_HUMAN |  |  | x |  |  |  |  |  |  |  |  |  |  |  | x |  |  |  |  |  |
| Pyridoxal kinase | PDXK_HUMAN |  |  | x |  |  |  |  |  |  |  |  |  |  |  |  |  |  |  |  |  |
| Pyruvate kinase isozymes M1/M2 | KPYM_HUMAN | x |  | x |  |  |  |  |  |  |  |  |  |  |  |  |  |  |  |  |  |
| Quinone oxidoreductase | QOR_HUMAN |  |  | x |  |  |  |  |  |  |  |  |  |  |  |  | x |  |  |  |  |
| Quinone oxidoreductase PIG3 | QORX_HUMAN |  |  | x |  |  |  |  |  |  |  |  |  |  |  |  | x |  |  |  |  |
| Rab GDP dissociation inhibitor alpha | GDIA_HUMAN | x |  |  |  |  |  |  |  |  | x |  |  |  |  | x |  |  | x |  |  |
| Rab GDP dissociation inhibitor beta | GDIB_HUMAN |  |  |  |  |  |  |  |  |  | x |  |  |  |  | x |  |  | x |  |  |
| Rab GTPase-binding effector protein 1 | RABE1_HUMAN | x |  |  |  |  |  |  |  |  | x |  |  |  |  |  | x |  |  |  |  |
| Rab11 family-interacting protein 3 | RFIP3_HUMAN |  |  |  |  |  |  |  |  |  | x |  |  |  |  |  |  |  |  |  |  |
| Rab11 family-interacting protein 4 | RFIP4_HUMAN |  |  |  |  |  |  |  |  |  | x |  |  |  |  |  |  |  |  |  |  |
| RAD51-associated protein 2 | R51A2_HUMAN |  |  |  |  |  |  |  |  |  |  |  |  |  |  |  |  |  |  |  | x |
| Radixin | RADI_HUMAN |  |  |  |  |  |  |  |  |  |  |  |  |  |  |  |  |  |  | x |  |
| Ran-binding protein 6 | RNBP6_HUMAN | x |  |  |  |  |  |  |  |  | x |  |  |  |  | x |  |  |  |  |  |
| Rap guanine nucleotide exchange factor 4 | RPGF4_HUMAN |  |  |  |  |  |  |  |  |  |  |  |  |  |  | x |  |  | x |  |  |
| Ras and EF-hand domain-containing protein | RASEF_HUMAN |  |  |  |  |  |  |  |  |  | x |  |  |  |  | x |  |  | x |  |  |
| Ras GTPase-activating-like protein IQGAP1 | IQGA1_HUMAN |  |  |  |  |  |  |  |  |  |  |  |  |  |  |  |  |  | x |  |  |
| Ras-GEF domain-containing family member 1B | RGF1B_HUMAN |  |  |  |  |  |  |  |  |  |  |  |  |  |  | x |  |  | x |  |  |
| Ras-related C3 botulinum toxin substrate 1 | RAC1_HUMAN | x |  |  |  |  |  |  |  |  | x |  |  |  |  | x |  |  | x |  |  |
| Ras-related protein Rab-6A | RAB6A_HUMAN |  |  |  |  |  |  |  |  |  | x |  |  |  |  | x |  |  |  |  |  |
| Ras-related protein Rab-10 | RAB10_HUMAN |  |  |  |  |  |  |  |  |  |  | x |  |  |  | x |  |  |  |  |  |
| Ras-related protein Rab-13 | RAB13_HUMAN | x |  |  |  |  |  |  |  |  | x |  |  |  |  | x |  |  | x |  |  |
| Ras-related protein Rab-1A | RAB1A_HUMAN |  |  |  |  |  |  |  |  |  | x |  |  |  |  | x |  |  | x |  |  |
| Ras-related protein Rab-15 | RAB15_HUMAN |  |  |  |  |  |  |  |  |  | x |  |  |  |  | x |  |  | x |  |  |
| Ras-related protein Rab-5B | RAB5B_HUMAN |  |  |  |  |  |  |  |  |  | x |  |  |  |  | x |  |  | x |  |  |
| Ras-related protein Rab-6B | RAB6B_HUMAN |  |  |  |  |  |  |  |  |  | x |  |  |  |  | x |  |  | x |  |  |
| Ras-related protein Rap-1A | RAP1A_HUMAN |  |  |  |  |  |  |  |  |  | x |  |  |  |  | x |  |  | x |  |  |
| Ras-related protein Rap-2a | RAP2A_HUMAN |  |  |  |  |  |  |  |  |  | x |  |  |  |  | x |  |  | x |  |  |
| Ras-related protein Rap-2b | RAP2B_HUMAN |  |  |  |  |  |  |  |  |  |  |  |  |  |  | x |  |  |  |  |  |
| RB1-inducible coiled-coil protein 1 | RBCC1_HUMAN |  |  |  |  |  |  |  |  |  |  |  |  |  |  | x |  |  |  |  |  |
| Receptor-type tyrosine-protein phosphatase F | PTPRF_HUMAN | x |  | x |  |  |  |  |  |  | x | x |  |  |  |  |  |  | x |  |  |
| Receptor-type tyrosine-protein phosphatase kappa | PTPRK_HUMAN |  |  | x |  |  |  |  |  |  | x | x |  |  |  |  |  |  | x |  |  |
| Receptor-type tyrosine-protein phosphatase S | PTPRS_HUMAN |  |  | x |  |  |  |  |  |  | x | x |  |  |  |  |  |  | x |  |  |
| Receptor-type tyrosine-protein phosphatase zeta | PTPRZ_HUMAN |  |  | x |  |  |  |  |  |  | x | x |  |  |  |  |  |  | x |  |  |
| Regulator of G-protein signaling 14 | RGS14_HUMAN |  |  |  |  |  |  |  |  |  |  |  | x |  |  |  |  | x | x |  |  |
| Renin receptor | RENR_HUMAN |  |  |  |  |  |  |  |  |  |  | x |  |  |  |  |  |  |  |  |  |
| Retinal dehydrogenase 1 | AL1A1_HUMAN | x |  | x |  |  |  |  |  |  |  |  |  |  |  | x |  |  |  |  |  |
| Retinol-binding protein 1 | RET1_HUMAN |  |  |  |  |  |  |  |  |  | x |  |  |  |  | x |  |  | x |  |  |
| Retinol-binding protein 3 | RET3_HUMAN |  | x |  |  |  |  |  |  |  | x |  |  | x |  |  |  |  |  |  |  |
| Retinol-binding protein 4 | RET4_HUMAN | x | x |  |  |  |  |  |  |  | x |  |  |  |  |  |  |  |  |  |  |
| Retinoschisin | XLRS1_HUMAN |  | x |  |  |  |  |  |  |  | x |  |  | x |  | x |  |  | x | x |  |
| Rho GDP-dissociation inhibitor 1 | GDIR1_HUMAN |  |  |  |  |  |  |  |  |  |  |  |  |  |  | x |  | x | x |  |  |
| Rho GTPase-activating protein 4 | RHG04_HUMAN |  |  |  |  |  |  |  |  |  |  |  |  |  |  | x | x |  | x |  |  |
| Rho GTPase-activating protein 8 | RHG08_HUMAN | x |  |  |  |  |  |  |  |  |  |  |  |  |  | x |  |  |  |  |  |
| Rho guanine nucleotide exchange factor 15 | ARHGF_HUMAN |  |  |  |  |  |  |  |  |  |  |  |  |  |  |  |  |  |  |  | x |
| Rho guanine nucleotide exchange factor 17 | ARHGH_HUMAN |  |  |  |  |  |  |  |  |  |  |  |  |  |  |  |  |  |  |  | x |
| Rho guanine nucleotide exchange factor 18 | ARHGI_HUMAN |  |  |  |  |  |  |  |  |  | x |  |  |  |  | x |  |  | x |  |  |
| Ribokinase | RBSK_HUMAN |  |  | x |  |  |  |  |  |  |  |  |  |  |  | x |  |  |  |  |  |
| Ribonuclease III | CJ068_HUMAN |  |  | x |  |  |  |  |  |  |  |  |  |  |  |  |  |  |  |  |  |
| Ribonuclease inhibitor | RINI_HUMAN |  |  | x |  |  |  |  |  |  |  |  |  |  |  | x |  |  |  |  |  |
| Ribonuclease pancreatic | RNAS1_HUMAN |  | x | x |  |  |  |  |  |  |  |  |  |  |  | x |  |  |  |  |  |
| Ribonuclease UK114 | UK114_HUMAN |  |  | x |  |  |  |  |  |  |  |  |  |  |  |  |  |  |  |  |  |
| Ribose-phosphate pyrophosphokinase 1 | PRPS1_HUMAN |  |  | x |  |  |  |  |  |  |  |  |  |  |  | x |  |  |  |  |  |
| Ribosomal L1 domain-containing protein 1 | RL1D1_HUMAN | x |  |  |  |  |  |  |  |  |  |  |  |  |  | x |  |  |  |  |  |
| RING finger protein 207 | RN207_HUMAN |  |  |  |  |  |  |  |  |  |  |  |  |  |  | x |  |  |  |  |  |
| RNA 3~-terminal phosphate cyclase | RTC1_HUMAN |  |  | x |  |  |  |  |  |  |  |  |  |  |  | x |  |  |  |  |  |
| Rootletin | CROCC_HUMAN |  |  |  |  |  |  |  |  |  |  |  |  |  |  | x |  | x |  |  |  |
| Rotatin | RTTN_HUMAN |  |  |  |  |  |  |  |  |  |  |  |  |  |  | x |  |  |  |  |  |
| Ryanodine receptor 3 | RYR3_HUMAN |  |  |  |  |  |  |  |  |  | x |  |  |  | x |  |  |  | x |  |  |
| Sacsin | SACS_HUMAN | x |  |  |  |  |  |  |  |  |  |  |  |  |  |  | x |  |  |  |  |
| SAGA-associated factor 29 homolog | SGF29_HUMAN |  |  |  |  |  |  |  |  |  |  |  |  |  |  |  |  |  |  |  | x |
| Sarcosine dehydrogenase, mitochondrial | SARDH_HUMAN |  |  | x |  |  |  |  |  |  |  |  |  |  |  |  |  |  |  |  |  |
| SCY1-like protein 2 | SCYL2_HUMAN | x |  |  |  |  |  |  |  |  | x |  |  |  |  |  |  |  |  |  |  |
| SEC14-like protein 2 | S14L2_HUMAN |  |  |  |  |  |  |  |  |  | x |  |  |  |  |  |  |  |  |  |  |
| SET and MYND domain-containing protein 5 | SMYD5_HUMAN |  |  |  |  |  |  |  |  |  |  |  |  |  |  | x |  |  |  |  |  |
| Secernin-1 | SCRN1_HUMAN |  |  | x |  |  |  |  |  |  |  |  |  |  |  |  |  |  |  |  |  |
| Secernin-2 | SCRN2_HUMAN | x |  | x |  |  |  |  |  |  |  |  |  |  |  |  |  |  |  |  |  |
| Secreted frizzled-related protein 3 | SFRP3_HUMAN |  | x |  |  |  |  |  |  |  |  |  |  |  |  | x |  |  | x |  |  |
| Secretoglobin family 1D member 2 | SG1D2_HUMAN |  | x |  |  |  |  |  |  |  |  |  |  |  |  |  |  |  |  |  |  |
| Secretogranin-3 | SCG3_HUMAN |  | x |  |  |  |  |  |  | x |  |  |  |  |  | x |  |  |  |  |  |
| Seizure protein 6 homolog | SEZ6_HUMAN |  |  |  |  |  |  |  |  |  |  |  |  |  |  |  |  |  | x |  |  |
| Semaphorin-4B | SEM4B_HUMAN |  |  |  |  |  |  |  |  |  |  |  |  |  |  | x |  |  | x |  |  |
| Semaphorin-7A | SEM7A_HUMAN |  |  |  |  |  |  |  |  |  |  |  |  |  |  | x |  |  | x |  |  |
| Serine hydroxymethyltransferase, cytosolic | GLYC_HUMAN |  |  | x |  |  |  |  |  |  |  |  |  |  |  |  |  |  |  |  |  |
| Serine/threonine-protein kinase SMG1 | SMG1_HUMAN | x |  | x |  |  |  |  |  |  |  |  |  |  |  | x | x |  | x |  |  |
| Serine/threonine-protein kinase TAO3 | TAOK3_HUMAN |  |  | x |  |  |  |  |  |  |  |  |  |  |  |  |  |  |  |  |  |
| Serine/threonine-protein kinase WNK1 | WNK1_HUMAN |  |  | x |  |  |  |  |  |  |  |  |  |  |  | x |  |  |  |  |  |
| Serine/threonine-protein phosphatase 2A activator | PTPA_HUMAN |  |  | x |  |  |  |  |  |  |  |  |  |  |  | x |  |  |  |  |  |
| Serine/threonine-protein phosphatase PP1-beta catalytic subunit | PP1B_HUMAN | x |  | x |  |  |  |  |  |  |  |  |  |  |  | x | x |  | x |  |  |
| Serine-protein kinase ATM | ATM_HUMAN | x |  | x |  |  |  |  |  |  |  |  |  |  |  | x | x | x | x |  |  |
| Serotransferrin | TRFE_HUMAN | x | x |  |  |  |  |  |  |  | x |  |  |  |  |  |  |  |  |  |  |
| Serpin B12 | SPB12_HUMAN |  |  |  |  | x |  |  |  |  |  |  |  |  |  | x |  |  |  |  |  |
| Serpin B3 | SPB3_HUMAN | x |  |  |  | x |  |  |  |  |  |  |  |  |  | x |  |  |  |  |  |
| Serpin B6 | SPB6_HUMAN |  |  |  |  | x |  |  |  |  |  |  |  |  |  | x |  |  |  |  |  |
| Serpin B8 | SPB8_HUMAN |  |  |  |  | x |  |  |  |  |  |  |  |  |  | x |  |  |  |  |  |
| Serpin B9 | SPB9_HUMAN |  |  |  |  | x |  |  |  |  |  |  |  |  |  | x |  |  |  |  |  |
| Serpin E3 | SERP3_HUMAN |  | x |  |  | x |  |  |  |  |  |  |  |  |  | x |  |  |  |  |  |
| Serum albumin | ALBU_HUMAN | x | x |  |  |  |  |  |  |  | x |  |  |  |  |  |  |  |  |  |  |
| Serum amyloid A-4 protein | SAA4_HUMAN | x | x |  |  |  |  |  |  |  |  |  |  |  |  |  |  |  |  |  |  |
| Serum paraoxonase/arylesterase 1 | PON1_HUMAN | x | x | x |  |  |  |  |  |  |  |  |  |  |  |  |  |  |  |  |  |
| SET and MYND domain-containing protein 5 | SMD2_HUMAN |  |  |  |  |  |  |  |  |  |  |  |  |  |  |  |  |  |  |  | x |
| Sex hormone-binding globulin | SHBG_HUMAN | x | x |  |  |  |  |  |  |  |  |  |  |  |  |  |  |  |  |  |  |
| S-formylglutathione hydrolase | ESTD_HUMAN |  |  | x |  |  |  |  |  |  |  |  |  |  |  |  |  |  |  |  |  |
| Sister chromatid cohesion protein PDS5 homolog B | PDS5B_HUMAN |  |  |  |  |  |  |  |  |  |  |  |  |  |  | x |  |  |  |  |  |
| Skin-specific protein 32 | XP32_HUMAN |  |  |  |  |  |  |  |  |  |  |  | x |  |  |  |  |  |  |  |  |
| Small proline-rich protein 2D | SPR2D_HUMAN |  |  |  |  |  |  |  |  |  |  |  | x |  |  |  |  | x |  |  |  |
| Small proline-rich protein 3 | SPRR3_HUMAN |  |  |  |  |  |  |  |  |  |  |  | x |  |  |  |  |  |  |  |  |
| S-methyl-5~-thioadenosine phosphorylase | MTAP_HUMAN |  |  | x |  |  |  |  |  |  |  |  |  |  |  |  |  |  |  |  |  |
| Son of sevenless homolog 2 | SOS2_HUMAN | x |  |  |  |  |  |  |  |  |  |  |  |  |  | x |  |  | x |  |  |
| Sorbitol dehydrogenase | DHSO_HUMAN |  |  | x |  |  |  |  |  |  |  |  |  |  |  |  | x |  |  |  |  |
| Sorting nexin-29 | SNX29_HUMAN |  |  |  |  |  |  |  |  |  |  |  |  |  |  |  |  |  |  |  | x |
| SPARC-like protein 1 | SPRL1_HUMAN |  | x |  |  |  |  | x |  |  |  |  |  |  |  | x |  |  | x |  |  |
| SPATS2-like protein | SPS2L_HUMAN |  |  |  |  |  |  |  |  |  |  |  |  |  |  |  |  |  |  |  | x |
| Spatacsin | SPTCS_HUMAN |  |  |  |  |  |  |  |  |  |  |  |  |  |  |  |  |  |  |  | x |
| Spectrin alpha chain, brain | SPTA2_HUMAN | x |  |  |  |  |  |  |  |  |  |  |  |  |  |  |  | x |  |  |  |
| Spectrin beta chain, brain 1 | SPTB2_HUMAN |  |  |  |  |  |  |  |  |  |  |  |  |  |  |  |  | x |  |  |  |
| Spectrin beta chain, brain 2 | SPTN2_HUMAN |  |  |  |  |  |  |  |  |  |  |  |  |  |  |  |  | x |  |  |  |
| Spectrin beta chain, brain 4 | SPTN4_HUMAN | x |  |  |  |  |  |  |  |  |  |  |  |  |  |  |  | x |  |  |  |
| Spectrin beta chain, erythrocyte | SPTB1_HUMAN | x |  |  |  |  |  |  |  |  |  |  |  |  |  |  |  | x |  |  |  |
| Spondin-1 | SPON1_HUMAN |  | x |  |  |  |  |  |  |  |  |  |  |  |  |  |  |  | x | x |  |
| Sterol regulatory element-binding protein 1 | SRBP1_HUMAN |  |  |  |  |  |  |  |  |  |  |  |  |  |  | x |  |  |  |  |  |
| Stress-70 protein, mitochondrial | GRP75_HUMAN |  |  |  |  |  |  |  |  |  |  |  |  |  |  | x |  |  |  |  |  |
| Stress-induced-phosphoprotein 1 | STIP1_HUMAN |  |  |  |  |  |  |  |  |  |  |  |  |  |  |  |  |  |  |  | x |
| Structural maintenance of chromosomes flexible hinge domain-containing protein 1 | SMHD1_HUMAN |  |  |  |  |  |  |  |  |  |  |  |  |  |  | x |  |  |  |  |  |
| Structural maintenance of chromosomes protein 2 | SMC2_HUMAN |  |  |  |  |  |  |  |  |  |  |  |  |  |  | x |  |  |  |  |  |
| Structural maintenance of chromosomes protein 4 | SMC4_HUMAN |  |  |  |  |  |  |  |  |  |  |  |  |  |  | x |  |  |  |  |  |
| Sulfhydryl oxidase 1 | QSOX1_HUMAN | x | x | x |  |  |  |  |  |  |  |  |  |  |  |  |  |  |  |  |  |
| Superoxide dismutase [Cu-Zn] | SODC_HUMAN |  |  | x |  |  |  |  |  |  |  |  |  |  |  |  |  |  |  |  |  |
| Suppression of tumorigenicity 5 protein | ST5_HUMAN |  |  |  |  |  |  |  |  |  |  |  |  |  |  |  |  |  |  |  | x |
| Suppressor of cytokine signaling 6 | SOCS6_HUMAN |  |  |  |  |  |  |  | x |  |  |  |  |  |  | x |  |  | x |  |  |
| Synaptonemal complex protein 1 | SYCP1_HUMAN |  |  |  |  |  |  |  |  |  |  |  |  |  |  | x |  |  |  |  |  |
| Talin-1 | TLN1_HUMAN | x |  |  |  |  |  |  |  |  |  |  |  |  |  |  |  | x |  | x |  |
| Target of Nesh-SH3 | TARSH_HUMAN |  | x |  |  |  |  |  |  |  |  |  |  |  |  |  |  |  | x |  |  |
| Taste receptor type 2 member 10 | T2R10_HUMAN |  |  |  |  |  |  |  |  |  |  | x |  |  |  |  |  |  |  |  |  |
| TBC1 domain family member 1 | TBCD1_HUMAN |  |  |  |  |  |  |  |  |  |  |  |  |  |  | x |  |  |  |  |  |
| TBC1 domain family member 4 | TBCD4_HUMAN |  |  |  |  |  |  |  |  |  |  |  |  |  |  | x |  |  |  |  |  |
| T-complex protein 1 subunit alpha | TCPA_HUMAN |  |  |  |  |  |  |  |  |  |  |  |  |  |  |  |  | x |  |  |  |
| T-complex protein 1 subunit beta | TCPB_HUMAN |  |  |  |  |  |  |  |  |  |  |  |  |  |  | x |  |  |  |  |  |
| T-complex protein 1 subunit delta | TCPD_HUMAN |  |  |  |  |  |  |  |  |  |  |  |  |  |  |  |  |  |  |  | x |
| T-complex protein 1 subunit epsilon | TCPE_HUMAN |  |  |  |  |  |  |  |  |  |  |  |  |  |  |  |  |  |  |  | x |
| T-complex protein 1 subunit eta | TCPH_HUMAN |  |  |  |  |  |  |  |  |  |  |  |  |  |  | x |  |  |  |  |  |
| T-complex protein 1 subunit gamma | TCPG_HUMAN |  |  |  |  |  |  |  |  |  |  |  |  |  |  |  |  | x |  |  |  |
| T-complex protein 1 subunit theta | TCPQ_HUMAN |  |  |  |  |  |  |  |  |  |  |  |  |  |  | x |  | x |  |  |  |
| T-complex protein 1 subunit zeta | TCPZ_HUMAN |  |  |  |  |  |  |  |  |  |  |  |  |  |  | x |  |  |  |  |  |
| Telomere-associated protein RIF1 | RIF1_HUMAN |  |  |  |  |  |  |  |  |  |  |  |  |  |  | x |  | x |  |  |  |
| Tenascin | TENA_HUMAN |  | x |  |  |  |  |  |  |  | x |  |  |  |  | x |  |  | x | x |  |
| Teneurin-4 | TEN4_HUMAN |  |  |  |  |  |  |  |  |  | x |  |  |  |  | x |  |  | x |  |  |
| Tensin-1 | TENS1_HUMAN |  |  |  |  |  |  |  |  |  |  |  |  |  |  |  | x | x |  |  |  |
| Testican-1 | TICN1_HUMAN |  | x |  |  |  |  |  |  |  |  |  |  |  |  | x |  |  |  |  |  |
| Thioredoxin | THIO_HUMAN | x |  |  |  |  |  |  |  |  |  |  |  |  |  |  |  |  | x |  |  |
| Thioredoxin-like protein 1 | TXNL1_HUMAN |  |  |  |  |  |  |  |  |  |  |  |  |  |  |  | x |  | x |  |  |
| Thyroid receptor-interacting protein 11 | TRIPB_HUMAN | x |  |  |  |  |  |  |  |  |  |  |  |  |  | x |  | x |  |  |  |
| Thyroxine-binding globulin | THBG_HUMAN | x | x |  |  |  |  |  |  |  |  |  |  |  |  | x |  |  |  |  |  |
| TIP41-like protein | TIPRL_HUMAN |  |  |  |  |  |  |  |  |  |  |  |  |  |  |  |  |  |  |  | x |
| Titin | TITIN_HUMAN | x |  |  |  |  |  |  |  |  |  |  |  |  |  |  |  | x |  | x |  |
| TRAF2 and NCK-interacting protein kinase | TNIK_HUMAN |  |  | x |  |  |  |  |  |  |  |  |  |  |  |  |  |  |  |  |  |
| Transaldolase | TALDO_HUMAN |  |  | x |  |  |  |  |  |  |  |  |  |  |  |  |  |  |  |  |  |
| Transcription factor HIVEP3 | ZEP3_HUMAN |  |  |  |  |  |  |  |  |  |  |  |  |  |  | x |  |  |  |  |  |
| Transcription initiation factor TFIID subunit 1-like | TAF1L_HUMAN | x |  |  |  |  |  |  |  |  |  |  |  |  |  | x |  |  |  |  |  |
| Transforming protein RhoA | RHOA_HUMAN |  |  |  |  |  |  |  |  |  | x |  |  |  |  | x |  | x | x |  |  |
| Transitional endoplasmic reticulum ATPase | TERA_HUMAN | x |  |  |  |  |  |  |  |  | x |  |  |  |  |  |  |  |  |  |  |
| Transketolase | TKT_HUMAN | x |  | x |  |  |  |  |  |  |  |  |  |  |  | x |  |  |  |  |  |
| Translational activator GCN1 | GCN1L_HUMAN |  |  |  |  |  |  |  |  |  |  |  |  |  |  | x |  |  |  |  |  |
| Translationally-controlled tumor protein | TCTP_HUMAN |  |  |  |  |  |  |  |  |  |  |  |  |  |  | x |  | x |  |  |  |
| Translin-associated factor X-interacting protein 1 | TXIP1_HUMAN |  |  |  |  |  |  |  |  |  |  |  |  |  |  |  |  |  |  |  | x |
| Translin-associated protein X | TSNAX_HUMAN |  |  |  |  |  |  |  |  |  |  |  |  |  |  | x |  |  |  |  |  |
| Transmembrane protein 132A | T132A_HUMAN | x |  |  |  |  |  |  |  |  |  |  |  |  |  |  |  |  | x |  |  |
| Transmembrane protein 14C | TM14C_HUMAN |  |  |  |  |  |  |  |  |  |  |  |  |  |  |  |  |  |  |  | x |
| Transportin-1 | TNPO1_HUMAN |  |  |  |  |  |  |  |  |  | x |  |  |  |  |  |  |  |  |  |  |
| Transportin-2 | TNPO2_HUMAN |  |  |  |  |  |  |  |  |  | x |  |  |  |  |  |  |  |  |  |  |
| Transthyretin | TTHY_HUMAN | x | x |  |  |  |  |  |  |  | x |  |  |  |  |  |  |  | x |  |  |
| Triosephosphate isomerase | TPIS_HUMAN | x |  | x |  |  |  |  |  |  |  |  |  |  |  |  |  |  |  |  |  |
| Tripartite motif-containing protein 29 | TRI29_HUMAN |  |  |  |  |  |  |  |  |  |  |  |  |  |  | x |  |  |  |  |  |
| Tripeptidyl-peptidase 1 | TPP1_HUMAN |  |  | x | x |  |  |  |  |  |  |  |  |  |  |  |  |  |  |  |  |
| Tropomyosin alpha-3 chain | TPM3_HUMAN |  |  |  |  |  |  |  |  |  |  |  |  |  |  |  |  | x |  | x |  |
| Tropomyosin alpha-4 chain | TPM4_HUMAN |  |  |  |  |  |  |  |  |  |  |  |  |  |  |  |  |  |  | x |  |
| Trypsin-1 | TRY1_HUMAN | x | x |  | x |  |  |  |  |  |  |  |  |  |  | x |  |  | x |  |  |
| Trypsin-3 | TRY3_HUMAN | x | x |  | x |  |  |  |  |  |  |  |  |  |  | x |  |  | x |  |  |
| Tubulin alpha-1A chain | TBA1A_HUMAN | x |  |  |  |  |  |  |  |  | x |  |  |  |  |  |  | x |  | x |  |
| Tubulin alpha-1B chain | TBA1B_HUMAN | x |  |  |  |  |  |  |  |  | x |  |  |  |  |  |  | x |  | x |  |
| Tubulin alpha-1C chain | TBA1C_HUMAN |  |  |  |  |  |  |  |  |  | x |  |  |  |  |  |  | x |  | x |  |
| Tubulin alpha-8chain | TBA8_HUMAN |  |  |  |  |  |  |  |  |  |  |  |  |  |  | x |  | x |  |  |  |
| Tubulin beta 3 chain | TBB3_HUMAN |  |  |  |  |  |  |  |  |  |  |  |  |  |  | x |  | x |  |  |  |
| Tubulin Beta-4B chain | TBB4B_HUMAN |  |  |  |  |  |  |  |  |  |  |  |  |  |  | x |  | x |  |  |  |
| Tubulin beta chain | TBB5_HUMAN | x |  |  |  |  |  |  |  |  | x |  |  |  |  |  |  | x |  | x |  |
| Tubulin-folding cofactor B | TBCB_HUMAN |  |  |  |  |  |  |  |  |  | x |  |  |  |  | x |  | x |  | x |  |
| Tudor domain-containing protein 1 | TDRD1_HUMAN |  |  |  |  |  |  |  |  |  |  |  |  |  |  | x |  |  |  |  |  |
| Tudor domain-containing protein 5 | TDRD5_HUMAN |  |  |  |  |  |  |  |  |  |  |  |  |  |  | x |  |  |  |  |  |
| Tyrosine-protein kinase-like 7 | PTK7_HUMAN |  |  | x |  |  |  |  |  |  | x |  |  |  |  |  | x |  | x |  |  |
| Ubiquitin carboxyl-terminal hydrolase 5 | UBP5_HUMAN |  |  | x | x |  |  |  |  |  |  |  |  |  |  | x |  |  |  |  |  |
| Ubiquitin carboxyl-terminal hydrolase isozyme L1 | UCHL1_HUMAN | x |  | x | x |  |  |  |  |  |  |  |  |  |  |  |  |  |  |  |  |
| Ubiquitin carboxyl-terminal hydrolase isozyme L3 | UCHL3_HUMAN |  |  | x | x |  |  |  |  |  |  |  |  |  |  |  |  |  |  |  |  |
| Ubiquitin fusion degradation protein 1 homolog | UFD1_HUMAN |  |  |  |  |  |  |  |  |  |  | x |  |  |  | x |  |  |  |  |  |
| Ubiquitin thioesterase OTUB1 O | OTUB1_HUMAN |  |  | x | x |  |  |  |  |  |  |  |  |  |  |  |  |  |  |  |  |
| Ubiquitin/ISG15-conjugating enzyme E2 L6 | UB2L6_HUMAN |  |  | x |  |  |  |  |  |  |  |  |  |  |  | x | x |  |  |  |  |
| Ubiquitin-40S ribosomal protein S27a | RS27A_HUMAN |  |  |  |  |  |  |  |  |  |  |  |  |  |  | x |  |  |  |  |  |
| Ubiquitin-conjugating enzyme E2 N | UBE2N_HUMAN |  |  | x |  |  |  |  |  |  |  |  |  |  |  |  | x |  |  |  |  |
| Ubiquitin-conjugating enzyme E2 variant 1 | UB2V1_HUMAN |  |  | x |  |  |  |  |  |  |  |  |  |  |  |  |  |  |  |  |  |
| Ubiquitin-like modifier-activating enzyme 1 | UBA1_HUMAN |  |  | x |  |  |  |  |  |  |  |  |  |  |  | x |  |  | x |  |  |
| Ubiquitin-like modifier-activating enzyme 6 | UBA6_HUMAN |  |  | x |  |  |  |  |  |  |  |  |  |  |  | x |  |  | x |  |  |
| Ubiquitin-protein ligase E3B | UBE3B_HUMAN |  |  | x |  |  |  |  |  |  |  |  |  |  |  |  |  |  |  |  |  |
| UDP-glucose 4-epimerase | GALE_HUMAN |  |  | x |  |  |  |  |  |  |  |  |  |  |  | x |  |  |  |  |  |
| Unconventional prefoldin RPB5 interactor | RMP_HUMAN |  |  |  |  |  |  |  |  |  |  |  |  |  |  | x |  |  |  |  |  |
| UDP-glucose:glycoprotein glucosyltransferase 2 | UGGG2_HUMAN |  |  | x |  |  |  |  |  |  |  |  |  |  |  | x |  |  |  |  |  |
| UPF0553 protein C9orf64 | CI064_HUMAN |  |  |  |  |  |  |  |  |  |  |  |  |  |  |  |  |  |  |  | x |
| UPF0663 transmembrane protein C17orf28 | CQ028_HUMAN |  |  |  |  |  |  |  |  |  |  |  |  |  |  |  |  |  |  |  | x |
| UTP--glucose-1-phosphate uridylyltransferase | UGPA_HUMAN |  |  | x |  |  |  |  |  |  |  |  |  |  |  |  |  |  |  |  |  |
| Utrophin | UTRO_HUMAN | x |  |  |  |  |  |  |  |  |  |  |  |  |  | x |  |  |  |  |  |
| Vacuolar protein sorting-associated protein 13D | VP13D_HUMAN |  |  |  |  |  |  |  |  |  |  |  |  |  |  |  |  |  |  |  | x |
| Vacuolar protein sorting-associated protein 13C | VP13C_HUMAN |  |  |  |  |  |  |  |  |  |  |  |  |  |  |  |  |  |  |  | x |
| Vasorin | VASN_HUMAN |  | x |  |  |  |  |  |  |  |  | x |  |  |  |  |  |  | x | x |  |
| Versican core protein | CSPG2_HUMAN |  | x |  |  |  |  |  |  |  |  |  |  |  |  |  |  |  | x | x |  |
| Vimentin | VIME_HUMAN |  |  |  |  |  |  |  |  |  |  |  | x |  |  |  |  | x |  | x |  |
| Vinculin | VINC_HUMAN |  |  |  |  |  |  |  |  |  |  |  |  |  |  | x |  | x |  | x |  |
| Vitamin D-binding protein | VTDB_HUMAN | x | x |  |  |  |  |  |  |  | x |  |  |  |  |  |  |  |  |  |  |
| Vitamin K-dependent protein S | PROS_HUMAN | x | x |  |  |  | x |  |  |  |  |  |  |  |  |  |  |  |  |  |  |
| Vitronectin | VTNC_HUMAN | x | x |  |  |  |  |  |  |  |  |  |  |  |  |  |  |  | x |  |  |
| Voltage-dependent N-type calcium channel subunit alpha-1B | CAC1B_HUMAN |  |  |  |  |  |  |  |  |  | x |  |  |  | x |  |  |  | x |  |  |
| Voltage-gated potassium channel subunit beta-1 | KCAB1_HUMAN |  |  |  |  |  |  |  |  |  | x |  |  |  | x |  |  |  |  |  |  |
| VPS10 domain-containing receptor SorCS1 | SORC1_HUMAN |  |  |  |  |  |  |  |  |  | x |  |  |  |  | x |  |  | x |  |  |
| V-type proton ATPase catalytic subunit A | VATA_HUMAN |  |  |  |  |  |  |  |  |  | x |  |  |  | x | x |  |  |  | x |  |
| V-type proton ATPase subunit S1 | VAS1_HUMAN |  |  |  |  |  |  |  |  |  | x |  |  |  |  |  |  |  |  | x |  |
| WAP, kazal, immunoglobulin, kunitz and NTR domain-containing protein 2 | WFKN2_HUMAN |  | x |  |  | x |  |  |  |  |  |  |  |  |  | x |  |  |  |  |  |
| WD repeat and HMG-box DNA-binding protein 1 | WDHD1_HUMAN |  |  |  |  |  |  |  |  |  |  |  |  |  |  | x |  |  |  |  |  |
| WD repeat-containing protein 1 | WDR1_HUMAN |  |  |  |  |  |  |  |  |  |  |  |  |  |  |  |  | x |  | x |  |
| WD repeat-containing protein 61 | WDR61_HUMAN |  |  |  |  |  |  |  |  |  |  |  |  |  |  |  |  |  |  |  | x |
| WD repeat-containing protein 66 | WDR66_HUMAN |  |  |  |  |  |  |  |  |  |  |  |  |  |  | x |  |  |  |  |  |
| WD repeat-containing protein 87 | WDR87_HUMAN |  |  |  |  |  |  |  |  |  |  |  |  |  |  |  |  |  |  |  | x |
| WD repeat-containing protein 96 | WDR96_HUMAN |  |  |  |  |  |  |  |  |  |  |  |  |  |  |  |  |  |  |  | x |
| WD repeat-containing protein KIAA1875 | K1875_HUMAN |  |  |  |  |  |  |  |  |  |  |  |  |  |  | x |  |  |  |  |  |
| Wnt inhibitory factor 1 | WIF1_HUMAN |  | x |  |  |  |  |  |  |  | x |  |  | x |  | x |  |  | x |  |  |
| WW domain-binding protein 2 | WBP2_HUMAN |  |  |  |  |  |  |  |  |  |  |  |  |  |  | x |  |  |  |  |  |
| Xaa-Pro aminopeptidase 1 | XPP1_HUMAN |  |  | x | x |  |  |  |  |  |  |  |  |  |  | x |  |  |  |  |  |
| Xaa-Pro dipeptidase | PEPD_HUMAN | x |  | x | x |  |  |  |  |  |  |  |  |  |  | x |  |  |  |  |  |
| Zeta-sarcoglycan | SGCZ_HUMAN |  |  |  |  |  |  |  |  |  |  |  |  |  |  |  |  | x |  |  |  |
| Zinc finger CCCH domain-containing protein 13 | ZC3HD_HUMAN |  |  |  |  |  |  |  |  |  |  |  |  |  |  | x |  |  |  |  |  |
| Zinc finger FYVE domain-containing protein 26 | ZFY26_HUMAN |  |  |  |  |  |  |  |  |  | x |  |  |  |  | x |  |  | x |  |  |
| Zinc finger MYM-type protein 4 | ZMYM4_HUMAN |  |  |  |  |  |  |  |  |  |  |  |  |  |  | x |  |  |  |  |  |
| Zinc finger protein 40 | ZEP1_HUMAN | x |  |  |  |  |  |  |  |  |  |  |  |  |  | x |  |  |  |  |  |
| Zinc finger protein 519 | ZN519_HUMAN |  |  |  |  |  |  |  |  |  |  |  |  |  |  | x |  |  |  |  |  |
| Zinc finger protein 638 | ZN638_HUMAN |  |  |  |  |  |  |  |  |  |  |  |  |  |  | x |  |  |  |  |  |
| Zinc finger protein GLI3 | GLI3_HUMAN |  |  |  |  |  |  |  |  |  |  |  |  |  |  | x |  |  |  |  |  |
| Zinc finger and SCAN domain-containing protein 30 | ZSC30_HUMAN |  |  |  |  |  |  |  |  |  |  |  |  |  |  | x |  |  |  |  |  |
| Zinc-alpha-2-glycoprotein | ZA2G_HUMAN | x | x |  |  |  |  |  |  |  |  | x |  |  |  | x |  |  |  |  |  |
| Zymogen granule protein 16 homolog B | ZG16B_HUMAN |  | x |  |  |  |  |  |  |  |  |  |  |  |  |  |  |  |  |  |  |
| ZZ-type zinc finger-containing protein 3 | ZZZ3_HUMAN |  |  |  |  |  |  |  |  |  |  |  |  |  |  | x |  |  |  |  |  |
|  |  |  |  |  |  |  |  |  |  |  |  |  |  |  |  |  |  |  |  |  |  |
| **Uncharacterized** |  |  |  |  |  |  |  |  |  |  |  |  |  |  |  |  |  |  |  |  |  |
| Uncharacterized protein C20orf26 | CT026_HUMAN |  |  |  |  |  |  |  |  |  |  |  |  |  |  |  |  |  |  |  | x |
| Uncharacterized protein KIAA0556 | K0556_HUMAN |  |  |  |  |  |  |  |  |  |  |  |  |  |  |  |  |  |  |  | x |
| Uncharacterized protein KIAA0753 | K0753_HUMAN |  |  |  |  |  |  |  |  |  |  |  |  |  |  |  |  |  |  |  | x |
| Uncharacterized protein KIAA0825 | K0825_HUMAN |  |  |  |  |  |  |  |  |  |  |  |  |  |  |  |  |  |  |  | x |
| Uncharacterized protein KIAA0947 | K0947_HUMAN |  |  |  |  |  |  |  |  |  |  |  |  |  |  |  |  |  |  |  | x |
| Uncharacterized protein KIAA1109 | K1109_HUMAN |  |  |  |  |  |  |  |  |  |  |  |  |  |  |  |  |  |  |  | x |
| Uncharacterized protein C20orf26 | CT026_HUMAN |  |  |  |  |  |  |  |  |  |  |  |  |  |  |  |  |  |  |  | x |
